# Draft Genome Sequences of *Xanthomonas sacchari* and Two Banana-Associated Xanthomonads Reveal Insights into the *Xanthomonas* Group 1 Clade

**DOI:** 10.3390/genes2041050

**Published:** 2011-12-02

**Authors:** David J. Studholme, Arthur Wasukira, Konrad Paszkiewicz, Valente Aritua, Richard Thwaites, Julian Smith, Murray Grant

**Affiliations:** 1 Department of Biosciences, University of Exeter, Geoffrey Pope Building, Stocker Road, Exeter, EX4 4QD, UK; E-Mails: awasukira@gmail.com (A.W.); k.h.paszkiewicz@exeter.ac.uk (K.P.); m.r.grant@exeter.ac.uk (M.G.); 2 National Crops Resources Research Institute (NaCRRI), P.O. Box 7084, Kampala, Uganda; E-Mail: arituavalentine@yahoo.com; 3 The Food and Environment Research Agency, Sand Hutton, York, YO41 1LZ, UK; E-Mails: richard.thwaites@fera.gsi.gov.uk (R.T.); julian.smith@fera.gsi.gov.uk (J.S.)

**Keywords:** banana, *Xanthomonas*, genome

## Abstract

We present draft genome sequences for three strains of *Xanthomonas* species, each of which was associated with banana plants (*Musa* species) but is not closely related to the previously sequenced banana-pathogen *Xanthomonas campestris* pathovar *musacearum*. Strain NCPPB4393 had been deposited as *Xanthomonas campestris* pathovar *musacearum* but in fact falls within the species *Xanthomonas sacchari*. Strain NCPPB1132 is more distantly related to *Xanthomonas sacchari* whilst strain NCPPB 1131 grouped in a distinct species-level clade related to *X. sacchari*, along with strains from ginger, rice, cotton and sugarcane. These three newly sequenced strains share many genomic features with the previously sequenced *Xanthomonas albilineans*, for example possessing an unsual *metE* allele and lacking the Hrp type III secretion system. However, they are distinct from *Xanthomonas albilineans* in many respects, for example showing little evidence of genome reduction. They also lack the SPI-1 type III secretion system found in *Xanthomonas albilineans*. Unlike *X. albilineans*, all three strains possess a *gum* gene cluster. The data reported here provide the first genome-wide survey of non-Hrp *Xanthomonas* species other than *Xanthomonas albilineans*, which is an atypical member of this group. We hope that the availability of complete sequence data for this group of organisms is the first step towards understanding their interactions with plants and identifying potential virulence factors.

## 1. Introduction

The genus *Xanthomonas* includes some 27 species of plant-associated Gram-negative bacteria. Collectively these species, and their constituent pathovars, cause disease on several hundred plant species, including many economically important crops. Phylogenetic analyses of the genus *Xanthomonas* consistently reveal that it comprises two distinct groups [1-6]. Young and colleagues recently proposed that Group 1 and Group 2 might represent two distinct genera [6].

Complete genome sequences are available for several members of Group 2, including *X. campestris* pv. *campestris*, *X. campestris* pv. *raphani*, *X. campestris* pv. *vesicatoria*, *X. citri*, *X. fuscans* subsp. *aurantifolii, X. oryzae* pv. *oryzae* and *X. oryzae* pv. *oryzicola* [7]. Draft genome sequences are available for several further members of the Group 2 *Xanthomonas* species [7]. The genomes of all of the members of Group 2 investigated so far encode a type III secretion system (T3SS), known as the Hrp T3SS, which is required for delivery of effector proteins into the plant host in order to overcome the host's defences. The name “Hrp” is derived from “hypersensitive response and pathogenicity”.

Of the members of *Xanthomonas* Group 1, the only published genome sequence [8] is that of *X. albilineans*, a xylem-limited pathogen that causes leaf scorch in sugarcane (*Saccharum* species). Analysis of the *X. albilineans* genome sequence revealed that this species displays several interesting features that are quite distinct from those of the Group 2 species. For example, *X. albilineans* lacks the Hrp T3SS that is universally conserved and central to pathogenicity in Group 2, but it encodes an alternative non-Hrp T3SS that shares sequence similarity to the *Salmonella* SPI-1 T3SS [9]. This raises the question of whether these features of the *X. albilineans* genome are also shared with the genomes of other Group 1 *Xanthomonas* species. Furthermore, *X. albilineans* appears to have undergone significant genome reduction, perhaps as a consequence of, or adaptation to, its xylem-limited lifestyle [8]. Therefore, it would be interesting to compare its genome with those of other members of Group 2 that do not share this highly specialized lifestyle.

Until recently, the only known *X. albilineans* virulence factor was the toxin albicidin. The complete genome sequence of *X. albilineans* enabled the identification of several new candidate virulence factors via screening of a transposon mutagenesis library [10]. Many of these were not shared with the Group 2 *Xanthomonas* species and may reflect the distinctiveness of the pathogenic strategies adopted by Groups 1 and 2. Therefore, it raises the question of whether these newly discovered virulence factors are also found in Group 1 species other than *X. albilineans*.

We recently sequenced the genome of an isolate of *X. campestris* pv. *musacearum*, which is the causative agent of banana *Xanthomonas* wilt, a disease currently causing devastation to the banana crop in East Africa [11] and is a member of *Xanthomonas* Group 2. Subsequently, we performed follow-up genome-sequencing studies on additional isolates that had been deposited in the National Collection of Plant Pathogenic Bacteria (NCPPB) as *X. campestris* pv. *musacearum*. We discovered that NCPPB4393 shared little sequence similarity with the previously sequenced isolate; rather, it showed very close sequence similarity with *X. sacchari*, a member of *Xanthomonas* Group 1. On further investigation we learned that NCPPB4393 had in fact been isolated from an insect on a diseased banana plant but that there was no evidence that the strain is actually pathogenic on banana [12]. We also sequenced two additional *Xanthomonas* strains (NCPPB1131 and NCPPB1132) that had been isolated from banana plants in Eastern and Western Samoa.

## 2. Results and Discussion

### 2.1. Bacterial Strains

Bacterial strains (Table 1) were obtained from the National Collection of Plant Pathogenic Bacteria (NCPPB) in the United Kingdom. NCPPB1131 and NCPPB1132 had been deposited in 1961 by Hayward A.C. after isolation from banana plants. NCPPB4393 was one of several strains isolates deposited by one of the authors of the present study (V.A.) in 2007. It was deposited in the strain collection as *X. campestris* pv. *musacearum.* However, the results of this study suggest that it is actually a member of the species *X. sacchari*. It was originally isolated by Mgenzi Byabachwezi from an insect on a diseased banana plant (Muleba district, Kagera region, North Western Tanzania, on the shores of Lake Victoria). Although the insect was collected from diseased banana, sugarcane is commonly grown in that district of Tanzania and so it is possible that the insect acquired the bacterium from sugarcane. Pathogenicity of strain NCPPB4393 has not been tested.

**Table 1:** Bacterial strains sequenced in this study.

**Strain**	**Host plant**	**Country**	**Year**
NCPPB1131	*Musa paradisiaca*	American (Eastern) Samoa	1961
NCPPB1132	*Musa canksii* var. *samoensis*	Western Samoa	1961
NCPPB4393	*Musa* species Isolated from insect on diseased plant	Tanzania	2007

### 2.2. Genome-Wide Sequence Data

We generated genome-wide sequence data for three strains listed in Table 1 using the Illumina GA2. After removing bar-code adaptors, the sequence reads were 70 nt long. We generated 1.7 million non-paired reads for NCPPB1131. For NCPPB1132 and NCPPB4393 we generated 1.9 million and 2.1 million paired reads respectively. These Whole Genome Shotgun project data have been deposited at DDBJ/EMBL/GenBank under the accession numbers AGHY00000000, AGHZ00000000 and AGDB00000000 respectively.

### 2.3. Phylogenetic Position of the Sequenced Xanthomonas Strains

**Figure 1:**
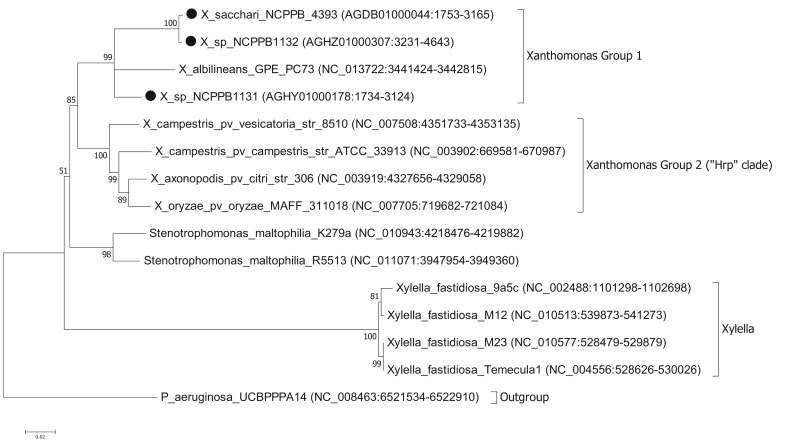
Molecular phylogenetic anaylsis of *atpD* gene of newly sequenced xanthomonads by Maximum Likelihood method. The evolutionary history was inferred by using the Maximum Likelihood method based on the Tamura-Nei model [13]. The bootstrap consensus tree inferred from 500 replicates is taken to represent the evolutionary history of the taxa analyzed. Branches corresponding to partitions reproduced in less than 50% bootstrap replicates are collapsed. The percentage of replicate trees in which the associated taxa clustered together in the bootstrap test (500 replicates) are shown next to the branches. Initial tree(s) for the heuristic search were obtained automatically as follows. When the number of common sites was <100 or less than one fourth of the total number of sites, the maximum parsimony method was used; otherwise BIONJ method with MCL distance matrix was used. The tree is drawn to scale, with branch lengths measured in the number of substitutions per site. The analysis involved 15 nucleotide sequences. Codon positions included were 1st + 2nd + 3rd + Noncoding. All positions containing gaps and missing data were eliminated. There were a total of 1,373 positions in the final dataset. Evolutionary analyses were conducted in MEGA5 [14]. The newly sequenced bacterial strains are indicated with black circles. For each nucleotide sequence, RefSeq accession numbers and coordinates are given in parentheses. The newly sequenced strains are indicated by black circles (●).

To ascertain the phylogenetic position of the three newly sequenced strains, we generated a series of phylogenetic trees based on the nucleotide sequences of house-keeping genes. We used the same set of seven genes that were used by Pieretti and colleagues [8]. For four of these genes (*atpD*, *dnaK*, *groEL* and *recA*) we were able to build trees from multiple sequence alignments using the maximum likelihood method. However, for three of the genes (*efp*, *glnA* and *gyrB*) we were unable to build valid multiple sequence alignments because of a lack of orthologues with detectable nucleotide sequence similarity. For example, *blastn* searches against the NCBI non-redundant nucleotide database, using *X. albilineans gyrB* (XALc_0004) as the query, yielded no significant matches in *Xylella* species. The phylogenetic reconstruction [13,14] based on *atpD* is shown in Figure 1. Phylogenetic trees based on *dnaK*, *groEL* and *recA* are included in the Supplementary Files. The trees for *atpD*, *dnaK* and *groEL* are all toplogically consistent with each other, though there is some variation in branch lengths and the precise position of *X. albilineans* within Group 1 is less well resolved in the *atpD* tree than in the others. However, analysis of the *recA* sequences yielded a different branching pattern in which *X. albilineans* falls within the *Xylella fastidiosa* lineage rather than within the *Xanthomonas* Group 1.

Our phylogenetic analyses clearly and consistently indicated that all three strains fell within the phylogenetic range of the genus *Xanthomonas*. Specifically, all three strains are more closely related to *X. albilineans* than to the Group 2 *Xanthomonas* species and therefore likely belong to Group 1.

We note that in our analyses based on three out of four house-keeping genes, the genus *Xanthomonas* comprises a single monophyletic clade, distinct from the related genera *Stenotrophomonas* and *Xylella*. This is consistent with previous studies [7,15] but contradicts recent claims that *X. albilineans* and *Xylella fastidiosa* form a monophyletic clade distinct from the Group 2 *Xanthomonas* species [8,10]. On the other hand, our analyses based on the *recA* gene were consistent with Pieretti's hypothesis that *X. albilineans* and *Xylella fastidiosa* form a monophyletic clade distinct from the Group 2. The incongruity between *atpD*, *groEL* and *dnaK* on the one hand and *recA* on the other implies that there has been recombination and that not all of these house-keeping genes truly reflect the vertical descent of the core genome. The most parsimonious explanation is that *recA* has undergone horizontal transfer in either the *Xylella* lineage or in the *X. albilineans* lineage. The reasons for discrepancy between our phylogenetic reconstructions for *atpD*, *groEL* and *dnaK* compared with that of Pieretti and colleagues [8] are two-fold. First, the analysis presented by Pieretti is a composite of genes displaying at least two distinct phylogenetic histories. Second, Pieretti's analysis [8] appears to be partly based on alignments of non-orthologous gene sequences (e.g., their *gyrB* sequences are not orthologous between *Xylella* and *Xanthomonas* species).

The results of our analysis are also inconsistent with those of Sharma and Patil [16]. In their study [16] they present a phylogenetic tree in which *X. albilineans* is the outgroup and *Stenotrophomonas* species fall within a monophyletic group along with the other *Xanthomonas* species. However, this discrepancy is explained by their misplacing the root of their tree. Sharma and Patil [16] do not explain how they chose the position of the root in their tree; they simply assume that *X. albilineans* is the outgroup without offering any justification for this. On the other hand, we used a phylogenetically distinct species (*P. aeruginosa*) as the outgroup in order to root our tree. If the tree of Sharma and Patil [16] is re-rooted with *Stenotrophomonas* species as the outgroup, then their tree is topologically congruent with ours. Note that Sharma and Patil do not include *Xylella fastidiosa* in their analysis.

**Figure 2:**
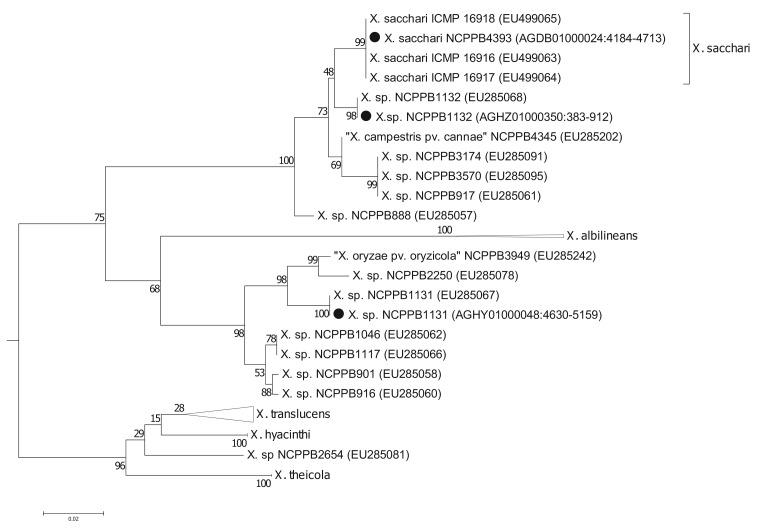
Phylogenetic positions of the three newly sequenced strains within *Xanthomonas* Group 1. The figure shows the evolutionary history of the *gyrB* gene as inferred by using the Maximum Likelihood method based on the Tamura-Nei model [13]. The tree with the highest log likelihood (−7358.2201) is shown. The percentage of trees in which the associated taxa clustered together is shown next to the branches. Initial tree(s) for the heuristic search were obtained automatically as follows. When the number of common sites was <100 or less than one fourth of the total number of sites, the maximum parsimony method was used; otherwise BIONJ method with MCL distance matrix was used. The tree is drawn to scale, with branch lengths measured in the number of substitutions per site. The analysis involved 219 nucleotide sequences, taken from the studies by Young and colleagues [5] and Parkinson and colleagues [6] as well as from the three newly sequenced strains. GenBank accession numbers are indicated for the sequences. However, for clarity, only the sub-tree corresponding to Group 1 is shown. The full length of each GenBank sequence entry was used for all of the Young [5] and Parkinson [6] sequences. For the sequences taken from our data, the coordinates of the subsequence are given in the parentheses, following the GenBank accession. All positions containing gaps and missing data were eliminated. There were a total of 517 positions in the final dataset. Evolutionary analyses were conducted in MEGA5 [14]. The newly sequenced strains are indicated by black circles (●).

To more precisely resolve the newly sequenced strains' positions within *Xanthomonas* Group 1, we performed phylogenetic analyses based on the gyrase B (*gyrB*) gene (Figure 2); partial sequences of *gyrB* are available from two studies [5,6] including many more *Xanthomonas* strains than those for which there are fully sequenced genomes. The sequences that we used are given in the Supplementary Files. We found that the *gyrB* sequence from NCPPB4393 was identical to those from strains of *X. sacchari*. Previously, *X. sacchari* was described as comprising strains isolated from diseased sugarcane [17]. Therefore, the description may need to be modified to include strains isolated from insects.

Strain NCPPB1132 is also closely related to *X. sacchari* but is more divergent than NCPPB4393 (Figure 2). It falls within a clade that included *X. sacchari sensu strictu* as well as *X.* “*campestris*” pv. *cannae* (from canna lilly, a relative of banana) and several unnamed strains isolated from sugarcane (NCPPB888 and NCPPB917), foxtail millet (NCPPB3174) and arrow leaf elephant ear (NCPPB3570).

Strain NCPPB1131 is more closely related to *X. albilineans* than to *X. sacchari*, but shows closest affinity with NCPPB2250 (Figure 2), which was originally isolated from ginger, a relative of banana. Strain NCPPB1131 is also similarly closely related to NCPPB3949, isolated from rice and erroneously deposited as *X. oryzae* pv. *oryzicola*. Parkinson and colleagues [6] describe a species-level clade (Slc 7) that included NCPPB1131, NCPPB3949 and strains isolated from ginger, cotton and sugarcane.

### 2.4. Comparison of the Three Genomes *Versus* X. albilineans

The total sizes of the genome assemblies were 3.8 Mb for NCPPB1131, 4.7 Mb for NCPPB1132 and 4.9 Mb for NCPPB4393. These can be used as estimates of genome size, but may be inaccurate since we have not closed the gaps in the draft assembly. The size for NCPPB1131 should be treated with particular caution, since the use of non-paired sequence reads yielded a very fragmented assembly. Contiguity of the assemblies can be represented by N_50_ scaffold lengths which were 1.1 Kb (NCPPB1131), 4.8 Kb (NCPPB1132) and 51.5 Kb (NCPPB4393). The numbers of scaffolds in each assembly were 4,158 (NCPPB1131), 1,652 (NCPPB1132) and 259 (NCPPB4393). Nevertheless, these estimates are congruent with sizes of previously sequenced *Xanthomonas* genomes, which range from 4.8 to 5.3 Mb for Group 2, whilst *X. albilineans* has a genome of less than 3.8 Mb.

**Figure 3:**
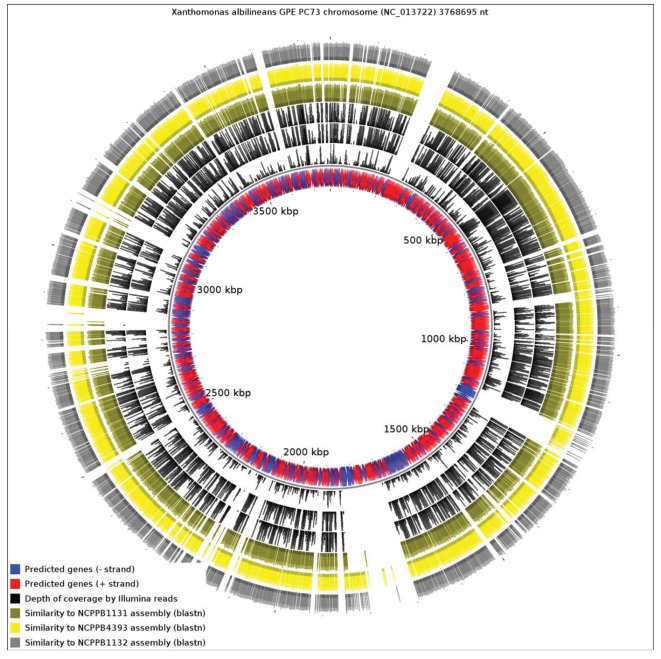
Alignment of the three *Xanthomonas* Group 1 new genome sequences against the chromosome of *X. albilineans*. The blue and red inner track represents annotated genes. The next three black tracks represent depth of coverage by Illumina sequence reads for NCPPB1131, NCPPB4393 and NCPPB1131 respectively. The four colored outer rings indicate sequence similarity to the genome assemblies of NCPPB1131 (olive), NCPPB4393 (yellow) and NCPPB1131 (grey) respectively.

We aligned the three genome assemblies against the genome sequence of *X. albilineans* species. We also aligned the reads, without performing *de novo* assembly. Figure 3 illustrates a genome-wide overview of these alignments. It should be noted that the sequencing depths obtained for the three strains ensures complete coverage over the entire breadth of the genomes. This means that by examining alignments of sequence reads against a reference sequence, independently from any *de novo* assembly, we can confidently determine the presence or absence of genes. The depths of coverage of each genome, as determined by depths of alignments of raw reads against assemblies, were 20× (NCPPB1131), 67× (NCPPB1132) and 72× (NCPPB4393).

Clearly, a significant proportion of the *X. albilineans* genome is not conserved (at the nucleotide sequence level) in the three newly sequenced Group 1 strains (Figure 3). Prominent amongst the non-conserved regions is the gene cluster encoding the *X. albilineans* SPI-1 T3SS (positions 1,703,391-1,730,688). We could find no evidence for any non-flagellar T3SS in any of the three strains. All three strains also lack the albicidin biosynthesis cluster at positions 1,740,869-1,788,517 and so they likely do not produce albicidin.

Pieretti and colleagues [8] observed that the genomes of two xylem-limited pathogens, *X. albilineans* and *Xylella fastidiosa*, both share an unusual allele of the *metE* gene, which encodes 5-methyltetrahydropterolyl-triglutamate-homocysteine methyltransferase, an enzyme required for methionine biosynthesis. They infer that the ancestor of *X. albilineans* and *X. fastidiosa* lost *metE*, while the rest of the xanthomonads retained it, and then this ancestor gained a new allele of *metE* by horizontal transfer. We reject this interpretation, since the last common ancestor of *X. albilineans* and *Xylella fastidiosa* was also the ancestor of the other xanthomonads, including *Stenotrophomonas* and all *Xanthomonas* species (Figure 1). We found that NCPPB1131, NCPPB1132 and NCPPB4393 all contain a *metE* gene that most closely resembles (at least 90% amino acid sequence identity) that of *X. albilineans* rather than those of Group 2 *Xanthomonas* species. This suggests that the *X. albilineans metE* occurs widely in the Group 1 *Xanthomonas* species and is not restricted only to xylem-limited species. The incongruence between the phylogeny of *metE* genes and the core house-keeping genes indicates that *metE* has been replaced independently in at least two distinct lineages during the evolution of the xanthomonads.

### 2.5. Genome Reduction

Genome reduction has occurred independently in two separate lineages of xanthomonads that are specialized for a xylem-limited lifestyle. That is, *Xylella fastidiosa* and *X. albilineans* have independently converged on a xylem-limited lifestyle, each having evolved from a different ancestor with a larger genome. The only other xylem-limited bacterial species for which complete genome sequence data are available is *Leifsonia xyli*. Interestingly, *L. xyli* also appears to have a reduced genome, its chromosome being 2.6 megabases long in contrast to its non-xylem-associated relative *Clavibacter michiganensis*, which has a 3.3-megabase chromosome [18]. Thus, genome reduction is associated with at least three distinct lineages of xylem-limited bacteria, suggesting that a stripped-down genome may be adaptive for this relatively stable environment. Complete genome data are not yet available for the other well-known xylem-limited species, *Ralstonia syzygii* [19], so we cannot yet be sure that this is a universal phenomenon.

**Figure 4:**
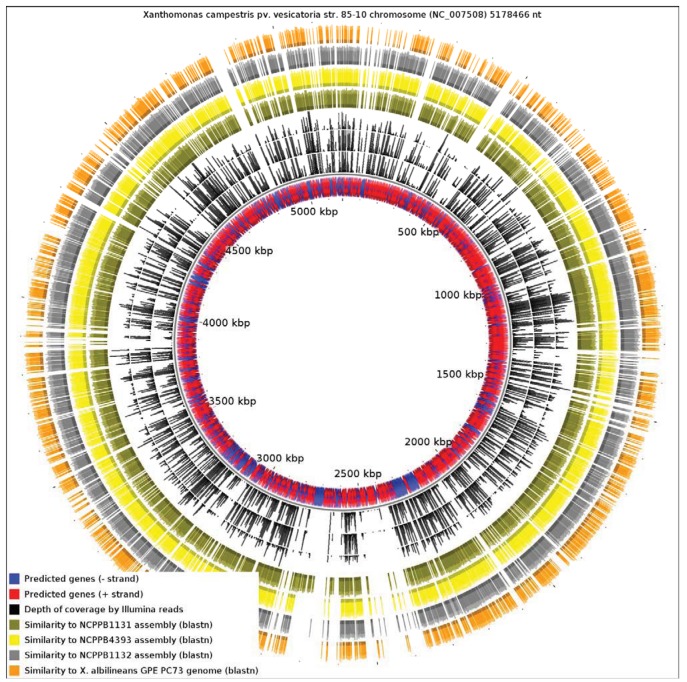
Alignment of the four *Xanthomonas* Group 1 genome sequences against the chromosome of *Xanthomonas campestris* pv. *vesicatoria*. The blue and red inner track represents annotated genes. The next three black tracks represent depth of coverage by Illumina sequence reads for NCPPB1131, NCPPB4393 and NCPPB1131 respectively. The four colored outer rings indicate sequence similarity to the genome assemblies of NCPPB1131 (olive), NCPPB4393 (yellow), NCPPB1131 (grey) and *X. albilineans* (orange) respectively.

Since the only sequenced member of *Xanthomonas* Group 1 is a specialized xylem-limited pathogen with a reduced genome, this raises the question of whether other members of Group 1 show similar evidence of genome reduction. Our results reveal that *X. albilineans* has undergone significantly more reduction than NCPPB1131, NCPPB1132 and NCPPB4393. We aligned all four available genomes from Group 1 against the reference sequence of *X. campestris* pv. *vesicatoria* (*Xcv*) 85-10 (RefSeq: NC_007508), a member of Group 2 (Figure 4). We found that only 13.82% of the *Xcv* chromosome was conserved in *X. albilineans*. Significantly larger portions of the *Xcv* were conserved in NCPPB1131, NCPPB1132 and NCPPB4393 (21.69%, 26.85% and 28.44% respectively). This is consistent with *X. albilineans* having lost more of the *Xanthomonas* genome than have the other three strains.

*X. albilineans* produces the toxin albicidin, which is a DNA-gyrase inhibitor [20]. In addition to its action on host-plant chloroplasts, it is also deleterious to most bacteria. Pieretti and colleagues [8] propose that albicidin played a key role in the erosion of the *X. albilineans* genome. Specifically, they propose that exposure to sub-lethal doses of intracellular albicidin induced recombination and mutagenesis. They note that *X. albilineans* has, in addition to its albicidin transporter (AlbF), an unusual DNA gyrase, containing a 43-amino-acid insertion, that confers resistance to albicidin [21]. They go on to propose that “*genome erosion induced by albicidin was likely arrested by the evolution of the albicidin-resistant DNA gyrase*” [8]. Interesting though it is, there is no evidence to support this conjecture. Nor is there any need to invoke such a special mechanism, since genome reduction is a common phenomenon, seen in many parasitic organisms that do not produce albicidin-like antibiotics. Furthermore, sequence insertions similar to that in *X. albilineans* gyrase A are not uncommon among the gamma-Proteobacteria (see Supplementary Files). Therefore, it is quite plausible that albicidin-insensitivity is an ancient and/or common trait that was already present in *X. albilineans* before it acquired the ability to produce albicidin.

Examination of our genome-wide sequence data revealed that the Group 1 strains NCPPB1131, NCPPB1132 and NCPPB4393 lack the gene cluster required for albicidin biosynthesis, yet all three strains encode a gyrase A resembling that of *X. albilineans*, including the 43 amino acid insertion that is supposed to be responsible for albicidin resistance. These strains do have other non-ribosomal peptide synthesis genes, so we cannot exclude the possibility that the 43 amino acid insertion confers resistance to some other unknown toxin. Nevertheless, the presence of this insert clearly does not correlate with the incidence of genome reduction. Aside from any involvement in genome reduction, albicidin-like antibiotics probably have had profound effects on the evolution and ecology of xylem-limited parasites. For example, the genome sequence of *L. xyli* encodes a close homologue of AlbF that may allow it to colonise the host simultaneously with *X. albilineans* [22].

### 2.6. Novel Genes in Xanthomonas Group 1

Until the present study, *X. albilineans* was the only member of Group 1 for which genome-wide sequence data were available. However, *X. albilineans* is an atypical example of the group, since it has undergone significant genome reduction associated with adaptation to life within the xylem. Therefore, we examined the genome sequence data from NCPPB1131, NCPPB1132 and NCPPB4393 for genomic regions that are absent from *X. albilineans* GPE PC73. Comprehensive lists of these regions are provided in the Supplementary Files and include heavy metal resistance proteins, haemolysins, haemagglutinins, citrate synthase, persistence factor HipA, sigma54-dependent activators, type-IV pili, drug efflux pumps, vancomycin B-type resistance protein VanW, xylanase, chitinase, beta-lactamase, restriction modification systems and many others.

There are, of course, some differences among the genomes of the Group I *Xanthomonas* strains. The genomes of NCPPB1132 and NCPPB4393 are alignable with each other over 88.64% and 84.33% of their respective lengths. That is, 12–16% of their genomes are variable and presumably subject to relatively recent horizontal transfer. They share 96.85% nucleotide sequence identity over the alignable conserved core portion of their genomes. Similarly they share 94.47% and 94.45% identity with NCPPB1131.

The genomes of NCPPB1131, NCPPB1132 and NCPPB4393 each encode a protein with 64% amino acid sequence identity to AvrXca (also known as AvrXccA1), a protein that confers avirulence on *Arabidopsis* in *X. campestris* pv. *raphani* [23]. Based on this avirulence activity, it has been speculated that AvrXca might be an effector. However, all three strains lack a T3SS, so it seems unlikely that it is secreted via the T3SS. Interestingly, at least two homologues of AvrXca are effectors secreted by the type-II secretion system (T2SS) [24-26], suggesting that AvrXca might also be a T2SS-dependent effector.

Each of the three newly sequenced genomes encode two cellulose-degrading enzymes that are absent from *X. albilineans*. Specifically, these include an endoglucanase that shares 61% amino acid sequence identity with XCC0028 from *X. campestris* pv. *campestris* ATCC 33913 and a cellulase (glycosyl hydrolase family 5) that shares 68% identity with XCV0358 from *X. campestris* pv. *vesicatoria* 85-10. Both enzymes are widely distributed among the Group 2 *Xanthomonas* species as well as in the three newly sequenced Group 1 strains, but absent from *X. albilineans.* It is possible that they play a role in degrading plant cell walls.

The *gum* genes, found in *Xylella fastidiosa* and all studied Group 2 *Xanthomonas* species, play a role in producing extracellular polysaccharides and forming biofilms and are implicated in pathogenicity. However, no *gum* genes have been found in *X. albilineans* [8]. We found homologues of these (*gumBGCEKDHIMJL*) conserved in all three newly sequenced strains. Therefore, it appears that the *gum* cluster was probably present in the common ancestor of *Xylella*, *Stenotrophomonas* and *Xanthomonas* and was subsequently lost by *X. albilineans* and *Stenotrophomonas* but retained in *X. sacchari* and the other Group 1 *Xanthomonas* species.

## 3. Experimental Section

Bacterial strains were obtained from the National Collection of Plant Pathogenic Bacteria (NCPPB) at FERA. Sequence alignments were performed using MAFFT [27], BWA [28], BLAST [29] and MUMMER [30]. DNA preparation and genome sequencing using the Illumina GA2 were performed as previously described [11]. We used CGview [31] to visualize whole-genome alignments and used MEGA5 for phylogenetic analyses. Literature references for previously sequenced bacterial genomes used in this study are listed cited in [10,11]. *De novo* assembly of Illumina sequence reads was performed using Velvet 1.1.03 [32]. We discarded any sequence reads that contained one or more ‘N’ prior to assembly. We used the following values for the hash-length parameter: 25 for NCPPB1131, 41 for NCPPB1132 and 49 for NCPPB4493. The coverage cut-off parameter was set to 4 in all three assemblies. For NCPPB1132 and NCPPB4393 read-pairs, we used Velvet's scaffolding step. We did not perform scaffolding on the NCPPB1131 data as the reads were not paired (*i.e*., we used single-end sequencing). The RAST server [33] was used for automated annotation of draft assemblies.

Note that in the genome-wide alignments (Figure 3 and Figure 4), the pattern of coverage by aligned raw reads does not exactly coincide with the coverage by aligned contigs/scaffolds. This inconsistency is inevitable since the two alignment methods use different criteria for assigning a match. The BWA alignment tool tolerates mismatches so long as the edit distance does not exceed two between the raw read and the reference genome sequence. On the other hand, BLAST uses an E-value threshold (1e-10 in this case) as the criterion for whether to accept a match. Furthermore, the process of assembly leads to the correction of errors by consensus of multiple sequence reads.

## 4. Conclusions

The ability to survive on banana plants has evolved more than once within the genus *Xanthomonas*, with strains isolated from banana falling within both major phylogenetic lineages: Group 1 (NCPPB1131 and NCPPB1132) and Group 2 (*X. campestris* pv. *musacearum*). Clearly their strategies are different. *Xanthomonas campestris* pv. *musacearum* encodes an apparently intact Hrp T3SS and a suite of effectors that it presumably uses to overcome the host's defences. On the other hand, the Group 1 strains, related to *X. sacchari* and *X. albilineans*, lack the T3SS and must use some other strategy to avoid triggering host defences. In the case of *X. albilineans*, the strategy appears to be one of stealth, where the pathogen restricts its colonization to the dead xylem. As a result, it has undergone substantial genome reduction, shedding genes unnecessary for this restricted niche. Very little information is available about the endophytic lifestyles of *X. sacchari* and other related members of *Xanthomonas* Group 1 and there is no evidence that they are limited to the xylem. Certainly the lack of genome reduction would be consistent with having to survive in more diverse conditions. The example strain that we sequenced here, NCPPB4393, apparently spent at least part of its life cycle associated with insects. We hope that the availability of complete sequence data for this group of organisms is the first step towards understanding their interactions with plants and identifying potential virulence factors.

## Supplementary Material

**Table:** Supplementary files

**File Name**	**File Type**	**Description**
GyraseA_alignment.pdf	PDF (Adobe Acrobat)	Multiple sequence alignment of partial GyrA proteins from *Xanthomonas albilineans* and other gamma-Proteobacteria
phylogenetic_trees.pdf	PDF (Adobe Acrobat)	Phylogenetic analysis of fully sequenced members of the genera *Xanthomonas, Xylella* and *Steonotrophomonas.* Maximum likelihood trees are based on nucleotide sequences of the house-keeping genes *dnaK*, *groEL* and *recA*
NCPPB1131-sequences- not-in-X_albilineans.html	HTML (web browser)	A list of regions in the genome of NCPPB1131 that share no detectable nucleotide sequence similarity with *X. albilineans*
NCPPB1132-sequences-not-in-X_albilineans.html	HTML (web browser)	A list of regions in the genome of NCPPB1132 that share no detectable nucleotide sequence similarity with *X. albilineans*
NCPPB4393-sequences-not-in-X_albilineans.html	HTML (web browser)	A list of regions in the genome of NCPPB4393 that share no detectable nucleotide sequence similarity with *X. albilineans*
NCPPB1131-RAST.xls	Microsoft Excel spreadsheet	Automated annotation results for NCPPB1131 draft genome sequence generated by the RAST server
NCPPB1132-RAST.xls	Microsoft Excel spreadsheet	Automated annotation results for NCPPB1132 draft genome sequence generated by the RAST server
NCPPB4393-RAST.xls	Microsoft Excel spreadsheet	Automated annotation results for NCPPB4393 draft genome sequence generated by the RAST server
NCPPB1131_genes_comparison.pdf	PDF (Adobe Acrobat)	A list of predicted genes in NCPPB1131 that are absent from NCPPB1132 and/or NCPPB4393 as judged by BWA alignments of raw Illumina sequence reads against the NCPPB1131 genome. Genomic coordinates are given on GenBank accession numbers
NCPPB1132_genes_comparison.pdf	PDF (Adobe Acrobat)	A list of predicted genes in NCPPB1132 that are absent from NCPPB1131 and/or NCPPB4393 as judged by BWA alignments of raw Illumina sequence reads against the NCPPB1132 genome. Genomic coordinates are given on GenBank accession numbers
NCPPB4393_genes_comparison.pdf	PDF (Adobe Acrobat)	A list of predicted genes in NCPPB4393 that are absent from NCPPB1131 and/or NCPPB1132 as judged by BWA alignments of raw Illumina sequence reads against the NCPPB4393 genome. Genomic coordinates are given on GenBank accession numbers
atpD_aligned_fasta.txt	Gapped FastA / plain text	Multiple sequence alignment of *atpD* used to generate the phylogenetic tree in Figure 1 in the main text
gyrB_aligned_fasta.txt	Gapped FastA / plain text	Multiple sequence alignment of *gyrB* used to generate the phylogenetic tree in Figure 1 in the main text

Supplementary File 1 ZIP-Document (ZIP, 7075 KB)
