# Correction: Studholme *et al*., Draft Genome Sequences of *Xanthomonas sacchari* and Two Banana-Associated Xanthomonads Reveal Insights into the *Xanthomonas* Group 1 clade. Genes 2011, *2*, 1050–1065

**DOI:** 10.3390/genes3010088

**Published:** 2012-01-11

**Authors:** David J Studholme, Arthur Wasukira, Konrad Paszkiewicz, Valente Aritua, Richard Thwaites, Julian Smith, Murray Grant

**Affiliations:** 1 Biosciences, University of Exeter, Geoffrey Pope Building, Stocker Road, Exeter, EX4 4QD, UK; E-Mails: awasukira@gmail.com (A.W); k.h.paszkiewicz@exeter.ac.uk (K.P.); m.r.grant@exeter.ac.uk (M.G.); 2 National Crops Resources Research Institute (NaCRRI), P.O. Box 7084, Kampala, Uganda; E-Mail: arituavalentine@yahoo.com (V.A.); 3 The Food and Environment Research Agency, Sand Hutton, York, YO41 1LZ, UK; E-Mails: richard.thwaites@fera.gsi.gov.uk (R.T.); julian.smith@fera.gsi.gov.uk (J.S.)

Following publication of our article [1], we found errors in analyses performed by the corresponding author (DJS) related to the phylogenetic relationship between *Xylella* species and the other xanthomonads. These errors do not make any difference to the main findings and conclusions reported in our paper. For example, the phylogenetic positions of NCPPB1131, NCPPB1132 and NCPPB4393 within the Group 1 *Xanthomonas* species are unaffected. However, we wish to apologize to the authors of a previous work [2] for creating any negative impression on the quality of their phylogenetic analyses and to take this opportunity to rectify the errors. The details of the errors are listed below:

(1) In section 2.3 of our paper [1], we wrote “*for three of the genes (*efp, glnA *and* gyrB) *we were unable to build valid multiple sequence alignments because of a lack of orthologues with detectable nucleotide sequence similarity. For example,* blastn *searches against the NCBI non-redundant nucleotide database, using* X. albilineans gyrB *(XALc_0004) as the query, yielded no significant matches in* Xylella *species”*. This statement was misleading as there are in fact orthologues of *efp*, *glnA* and *gyrB* in species of both *Xanthomonas* and *Xylella*.
(2) In section 2.3 of our paper [1], we wrote “*Second, Pieretti’s analysis … appears to be partly based on alignments of non-orthologous gene sequences (e.g. their* gyrB *sequences are not orthologous between* Xylella *and* Xanthomonas *species)”*. This statement was incorrect; there is no evidence to suggest that any non-orthologous sequences were aligned in the phylogenetic analyses performed by Pieretti *et al.* [2].
(3) In section 2.4 of our paper [1] we wrote “*the last common ancestor of* X. albilineans *and* Xylella fastidiosa *was also the ancestor of the other xanthomonads*”. In fact it remains unanswered whether or not *Xylella* forms a monophyletic group with X. albilineans and the other Group 1 *Xanthomonas* species. Therefore, it would be more appropriate to write that the last common ancestor of *X. albilineans* and *Xylella fastidiosa* ***may have been*** also the ancestor of the other xanthomonads.
