# Supplementary material for: Draft Genome Sequences of Xanthomonas sacchari and Two Banana-Associated Xanthomonads Reveal Insights into the Xanthomonas Group 1 Clade
Source: Genes (Basel). 2011 Dec 2;2(4):1050–65. doi: 10.3390/genes2041050 (PMC3927605; doi:10.3390/genes2041050)
Supplement: Supplementary File 1 — ZIP-Document (ZIP, 7075 KB) [file genes-02-01050-s001.zip › genes-11371-supplementary/GyraseA_alignment.pdf]

```
Xsp1132/690-821 ERDVLLFASNGKTVRFSE-----RPKDDEADDAVVDDTGADESAAADDDVAGEEGDDARGARRKSRGGVKPTGRSSRGVRGIRLAKSEYVVSLIVAEPAVGQDE--DADD-VDAAADTEADAEARNGDEAD----AYILTATEN
Xsp4393/690-821 ERDVLLFASNGKTVRFSE-----RPKDDETDDAAVDEAAADDGAVADDEAGGEEGDDARGARRKSRGGVKPTGRSSRGVRGIRLAKSEYVVSLIVAEPAVGQDE--DAED-VDAAADTEVEAEARNGDEAD----AYILTATEN
CBA16221.Xanthomonas_albilineans/690-821 ERDVLLFASNGKTVRFSE-----RPKDEEVDEVAVDEAAADDALAADDEAAGEDGDETRGARRKSRGGVKPTGRSSRGVRGIRLAKSEYVVSLIVAEPAEGQDE--DADD-ADLAQEAEADAQTRTADDAD----AYILTATEN
YP_003376211.Xanthomonas_albilineans/690-821 ERDVLLFASNGKTVRFSE-----RPKDEEVDEVAVDEAAADDALAADDEAAGEDGDETRGARRKSRGGVKPTGRSSRGVRGIRLAKSEYVVSLIVAEPAEGQDE--DADD-ADLAQEAEADAQTRTADDAD----AYILTATEN
ABY87166.Xanthomonas_albilineans/690-821 ERDVLLFASNGKTVRFSE-----RPKDEEADEVAVDEAAADDALAADDEATGEDGDETRGARRKSRGGVKPTGRSSRGVRGIRLAKSEYVVSLIVAEPAEGQDE--DADD-ADLAQEAEADAQTRTADDAD----AYILTATEN
YP_201045.Xanthomonas_oryzae_pv_oryzae_KACC10331/690-784 DRDVLLFASNGKTVRFGE------------------------------------------------STVRSMGRTATGVRGIRLTKGEEVVSLIVAERAGGADEEIEADD-VDDVVETTDGAETAVIEVADDGNVAYILTATEN
ZP_02242791.Xanthomonas_oryzae_pv_oryzicola_BLS256/690-784 DRDVLLFASNGKTVRFGE------------------------------------------------STVRSMGRTATGVRGIRLTKGEEVVSLIVAERAGGADEEIEADD-VDDVVETTDGAETAVIEVADDGNVAYILTATEN
YP_363403.Xanthomonas_campestris_pv_vesicatoria_str_85_10/690-784 DRDVLLFASNGKTVRFGE------------------------------------------------STVRSMGRTATGVRGIRLTKGEEVVSLIVAERAGGVEEEIEGEE-VDDVVEATDGGESAVIEVADDGNVAYILTATEN
ZP_06704976.Xanthomonas_fuscans_subsp_aurantifolii_str_ICPB_11122/690-784 DRDVLLFASNGKTVRFGE------------------------------------------------STVRSMGRTATGVRGIRLTKGEEVVSLIVAERAGGVEEEIEGDE-VDDVVEATDGSESAVIEVADDGNVAYILTATEN
NP_641963.Xanthomonas_axonopodis_pv_citri_str_306/690-784 DRDVLLFASNGKTVRFGE------------------------------------------------STVRSMGRTATGVRGIRLTKGEEVVSLIVAERAGGVEEEIEGDE-VDDVVEATDGGESAVIEVADDGNVAYILTATEN
ZP_06484730.Xanthomonas_campestris_pv_vasculorum_NCPPB702/690-784 DRDVLLFASNGKTVRFGE------------------------------------------------STVRSMGRTATGVRGIRLTKGEEVVSLIVAERAGGVEEEIEADD-VDDVVETTDGAEAAVIEVADDGNVAYILTATEN
NP_636945.Xanthomonas_campestris_pv_campestris_str_ATCC_33913/690-784 DRDVLLFASNGKTVRFGE------------------------------------------------STVRSMGRTATGVRGIRLAKGEEVVSLIVSERAGGVEDEVEDES-AEEVVETTDGAEPAVIDVADNGDVAYILTATEN
NP_299829.Xylella_fastidiosa_9a5c/690-784 ERDVLLFASNGKTVRFSE------------------------------------------------NTVRSMGRTATGVRGIKLTEGEEVVSLIIAEPATGVDMLEEAEETADDDIQTANTADSVHIDPTQD-DILCILTATEN
ZP_00651473.Xylella_fastidiosa_Dixon/690-784 ERDVLLFASNGKTVRFSE------------------------------------------------NTVRSMGRTATGVRGIKLTEGEEVVSLIIAEPATGVDMLEEAEETADDDIQTANTADSVHIDPTQD-DILCILTATEN
NP_780116.Xylella_fastidiosa_Temecula1/690-784 ERDVLLFASNGKTVRFSE------------------------------------------------NTVRSMGRTATGVRGIKLTEGEEVVSLIIAEPATGVDMLEEAEETADDDIQTANTAESVHIDPTQD-DILCILTATEN
ZP_00680440.Xylella_fastidiosa_Ann_1/690-784 ERDVLLFASNGKTVRFSE------------------------------------------------NTVRSMGRTATGVRGIKLTEGEEVVSLIIAEPATGVDMLEEAEETADDDIQTANTADSVHIGPTQD-DILCILTATEN
ZP_05133559.Stenotrophomonas_sp_SKA14/690-781 ERDILLFASNGKTVRFGE------------------------------------------------DKVRSMGRTATGVRGIKMPAGEEVVSLIVAESAGGIED--ENED-DNGVEEIAANGD-AVIDGADDASVQYILTATEN
YP_001972873.Stenotrophomonas_maltophilia_K279a/690-781 QRDILLFASNGKTVRFGE------------------------------------------------DKVRSMGRTATGVRGIKMPAGEEVVSLIVAESAGGIED--ENED-DSGVEEAAANGD-AVIEGADDASVQYILTATEN
YP_002028961.Stenotrophomonas_maltophilia_R551_3/690-781 ERDILLFASNGKTVRFGE------------------------------------------------DKVRSMGRTATGVRGIKMPAGEEVVSLIVAESAGGSED--ENED-DNGVEETAANGD-AVIDGADDASVQYILTATEN
ZP_04962496.Vibrio_cholerae_AM_19226/690-774 ESEIMLFSKFGKVVRFKE-----------------------SEETAVVDENGQPVLDENGQPEIKFKGVRPMGRTAAGVRGMKLADGDQVVSLIVPKTE-GD-----------------------------------VLTVTEN
ZP_01955901.Vibrio_cholerae_MZO_3/690-774 ESEIMLFSKFGKVVRFKE-----------------------SEETAVVDENGQPVLDENGQPEIKFKGVRPMGRTAAGVRGMKLADGDQVVSLIVPKTE-GD-----------------------------------VLTVTEN
ZP_04410790.Vibrio_cholerae_TM_11079_80/690-774 ESEIMLFSKFGKVVRFKE-----------------------SEETAVVDENGQPVLDENGQPEIKFKGVRPMGRTAAGVRGMKLADGDQVVSLIVPKTE-GD-----------------------------------VLTVTEN
NP_230903.Vibrio_cholerae_O1_biovar_El_Tor_str_N16961/690-774 ESEIMLFSKFGKVVRFKE-----------------------SEETAVVDENGQPVLDENGQPEIKFKGVRPMGRTAAGVRGMKLADGDQVVSLIVPKTE-GD-----------------------------------VLTVTEN
ZP_04400652.Vibrio_cholerae_B33/690-774 ESEIMLFSKFGKVVRFKE-----------------------SEETAVVDENGQPVLDENGQPEIKFKGVRPMGRTAAGVRGMKLADGDQVVSLIVPKTE-GD-----------------------------------VLTVTEN
ZP_05419603.Vibrio_cholera_CIRS_101/690-774 ESEIMLFSKFGKVVRFKE-----------------------SEETAVVDENGQPVLDENGQPEIKFKGVRPMGRTAAGVRGMKLADGDQVVSLIVPKTE-GD-----------------------------------VLTVTEN
ZP_01979964.Vibrio_cholerae_MZO_2/690-774 ESEIMLFSKFGKVVRFKE-----------------------SEETAVVDENGQPVLDENGQPEIKFKGVRPMGRTAAGVRGMKLADGDQVVSLIVPKTE-GD-----------------------------------VLTVTEN
ZP_01948050.Vibrio_cholerae_1587/690-774 ESEIMLFSKFGKVVRFKE-----------------------SEETAVVDENGQPVLDENGQPEIKFKGVRPMGRTAAGVRGMKLADGDQVVSLIVPKTE-GD-----------------------------------VLTVTEN
ZP_04405090.Vibrio_cholerae_TMA_21/690-774 ESEIMLFSKFGKVVRFKE-----------------------SEETAVVDENGQPVLDENGQPEIKFKGVRPMGRTAAGVRGMKLADGDQVVSLIVPKTE-GD-----------------------------------VLTVTEN
ZP_05715247.Vibrio_mimicus_VM573/690-774 ESEIMLFSKFGKVVRFKE-----------------------SEETAVVDENGQPVLDENGQPEIKFKGVRPMGRTAAGVRGMKLADGDQVVSLIVPKAE-GD-----------------------------------VLTVTEN
ZP_05719378.Vibrio_mimicus_VM603/690-774 ESEIMLFSKFGKVVRFKE-----------------------SEETAVVDENGQPVLDENGQPEIKFKGVRPMGRTAAGVRGMKLADGDQVVSLIVPKAE-GD-----------------------------------VLTVTEN
ZP_06078960.Vibrio_sp_RC586/690-774 ESDIMLFSKFGKVVRFKE-----------------------SEETAVVDENGQPVLDENGQPEIKFKGVRPMGRTAAGVRGMKLADGDQVVSLIVPKAE-GD-----------------------------------VLTVTEN
ZP_05925312.Vibrio_sp_RC341/690-760 ESDIMLFSKSGKVVRFNE----------------------------------KPIEDEEGNTK---GGVRPMGRTAAGVRGIKLVEGDQVVSLIVPKND-GD-----------------------------------ILTVTEN
ZP_02197384.Vibrio_sp_AND4/690-774 DSEIMLFSKAGKVVRFKE-----------------------AEEIPVLDEQGKPVLDENGQPEIKFKGVRPMGRTASGVRGMKLAENDQVVSLIVPSNE-GD-----------------------------------ILTVTQN
ZP_05881832.Vibrio_metschnikovii_CIP_69_14/690-760 ESDIMLFSKSGKVVRFNE----------------------------------KPIVDDEGNVK---GGVRPMGRTASGVRGMKLVKGDQVVSLIVPKTD-GD-----------------------------------ILTVTEN
ZP_01237041.Vibrio_angustum_S14/690-760 DCDIMLFSAFGKVVRFYE----------------------------------SPVIDAEGNAK---GGVRGMGRTAAGVRGIKLAEGDKVISLIVPHND-GD-----------------------------------VLTVTEN
ZP_01160577.Photobacterium_sp_SKA34/690-760 DCDIMLFSAFGKVVRFYE----------------------------------SPVIDAEGNAK---GGVRGMGRTAAGVRGIKLAEGDKVISLIVPHND-GD-----------------------------------VLTVTEN
YP_927606.Shewanella_amazonensis_SB2B/690-774 SSEIMLFSDAGKVVRFKE-----------------------GEEVAVVDENGNPVLDEEGKPQINFKGVRPMGRGATGVRGIALEEGQRVVSLIVPKDD-SA-----------------------------------ILTVTAN
YP_001554832.Shewanella_baltica_OS195/690-769 DDEIMLFSNEGKVVRFGE-----------------KVRDSETGEVKTDPETGEEVL-----------SLRPMGRTATGVRGIRLEEGQKVVSLIVPKGD-GA-----------------------------------ILTVTEN
YP_001050432.Shewanella_baltica_OS155/690-769 DDEIMLFSNEGKVVRFGE-----------------KVRDSETGEVKTDPETGEEVL-----------SLRPMGRTATGVRGIRLEEGQKVVSLIVPKGD-GA-----------------------------------ILTVTEN
YP_963331.Shewanella_sp_W3_18_1/690-769 DDEIMLFSNEGKVVRFGE-----------------KVRDSETGEVKTDPETGEEVL-----------SLRPMGRTATGVRGIRLEEGQKVVSLIVPKGD-GA-----------------------------------ILTVTEN
YP_734050.Shewanella_sp_MR_4/716-800 DDEIMLFSNEGKVVRFKE-----------------------GEEVSVLDENGNPVLDEEGNPQINFKGVRPMGRGATGVRGIKLEEGQKVVSLIVPKGD-GA-----------------------------------ILTVTEN
YP_738104.Shewanella_sp_MR_7/716-800 DDEIMLFSNEGKVVRFKE-----------------------GEEVAVLDENGNPVLDEEGNPQINFKGVRPMGRGATGVRGIKLEEGQKVVSLIVPKGD-GA-----------------------------------ILTVTEN
YP_869609.Shewanella_sp_ANA_3/690-774 DDEIMLFSNEGKVVRFKE-----------------------GEEVAVLDENGNAVLDEEGNPQINFKGVRPMGRGATGVRGIKLEEGQRVVSLIVPKGD-GA-----------------------------------ILTVTEN
YP_562954.Shewanella_denitrificans_OS217/690-769 SDDIMLFSNEGKVVRFNE-----------------KARDSETGEVKIDAETGEEII-----------ALRPMGRTATGVRGIKLEDGQKVVSLIVPKGD-GA-----------------------------------ILTVTEN
YP_002311810.Shewanella_piezotolerans_WP3/690-769 NDDIMLFSNEGKVVRFNE-----------------KARNAETGEVKIDPETGEEVI-----------ALRAMGRTATGVRGIKLEDGQTVVSLIVPKED-GA-----------------------------------ILTVTEN
ZP_02156186.Shewanella_benthica_KT99/690-769 KSDIMLFSDAGKVVRFNE-----------------MVRDSETGLVKLDAETNEELV-----------ALRPMGRTATGVRGIRLEDGQKVVSLIVPKAD-GA-----------------------------------ILTITEN
ZP_04716873.Alteromonas_macleodii_ATCC_27126/690-769 DDDIMLFSDAGKVVRFNE-----------------KLRDSETGEVKRDPETGEELL-----------ALRPMGRTATGVRGIRLQDGQKVVSLIVPKGD-GP-----------------------------------ILTATEN
YP_662040.Pseudoalteromonas_atlantica_T6c/690-769 SSDIMLFSDAGKVVRFNE-----------------KARDSETGAVKIDPETGEEIL-----------ALRPMGRTATGVRGIRLQDGQKVVSLIVPQGD-GA-----------------------------------ILTATEN
ZP_01614517.Alteromonadales_bacterium_TW_7/692-768 TNEIMLFTDAGKVVRFKE-----------------------AEESAVVDEN--------GNPEIRFKGVRPMGRTATGVRGIKMTDEQRVVSLIVPKTD-GA-----------------------------------ILTVTEN
YP_339939.Pseudoalteromonas_haloplanktis_TAC125/692-776 SNEIMLFTDAGKVVRFKE-----------------------AEETTVVDENGNPVLDEEGNPEIRFKGVRPMGRTATGVRGIKMPEGQRVVSLIVPKTD-GA-----------------------------------ILTVTEN
ZP_01215928.Psychromonas_sp_CNPT3/691-775 ENEIMIFSDAGKVVRFKE-----------------------AEETLVVDEQGNPVLDENGVQEIKFKGVRPMGRTATGVRGIKLKEGEKAVSLIVPNAT-GH-----------------------------------ILTCTEN
YP_856853.Aeromonas_hydrophila_subsp_hydrophila_ATCC_7966/691-788 SNEIMLFSDAGKVVRFAEGSGRADA---DATDEVDTDD----DAGNDDDGSDNGEG---TESKGTFKGVRPMGRTAAGVRGIKLGAGDKVVSLIVPRGD-GA-----------------------------------ILTATEN
YP_001141770.Aeromonas_salmonicida_subsp_salmonicida_A449/691-798 SNEIMLFSDAGKVVRFNEGSGRADAADADVSDDVDGDDESVIDAGNDDDGSDNGEGSESTESKGTFKGVRPMGRTAGGVRGIRLLNGDKVVSLIVPRGE-GA-----------------------------------ILTATEN
YP_003466665.Xenorhabdus_bovienii_SS_2004/691-763 SNEVMLFSAKGKVVRFLE-----------------------------------EDEETLEDGTIRVKGVRAMGRTAAGVRGIKLSAGDKVVSLIIPRGE-GE-----------------------------------ILTVTEN
```

Multiple sequence alignment of partial gyrase A proteins from *Xanthomonas albilineans* and other gamma-proteobacteria. The 43 amino acid insert, which was previously reported to be unique to *X. albilineans*, is highlighted in yellow.
