# Supplementary material for: Draft Genome Sequences of Xanthomonas sacchari and Two Banana-Associated Xanthomonads Reveal Insights into the Xanthomonas Group 1 Clade
Source: Genes (Basel). 2011 Dec 2;2(4):1050–65. doi: 10.3390/genes2041050 (PMC3927605; doi:10.3390/genes2041050)
Supplement: Supplementary File 1 — ZIP-Document (ZIP, 7075 KB) [file genes-02-01050-s001.zip › genes-11371-supplementary/NCPPB1131-sequences-not-in-X_albilineans.html]

Regions of the Xanthomonas species NCPPB1131 genome that show no detectable nucleotide sequence similarity with X. albilineans


### Regions of the *Xanthomonas* species NCPPB1131 genome that show no detectable nucleotide sequence similarity with *X. albilineans*

No hits against these regions were found when performing *blastn* searches against the genome of *X. albilineans* GPE PC73 (RefSeq:NC\_013722), using the NCPPB1131 draft genome assembly as the query.

| Length of region (nucleotides) | GenBank accession and coordinates of region | Predicted genes in this region (using RAST: Aziz et al. 2008 BMC Genomics 9:75) |
| --- | --- | --- |
| 15211 | AGHY01000095.1:1..15211 | FIG00856023: hypothetical protein  FIG01112043: hypothetical protein  Phosphoadenylyl-sulfate reductase [thioredoxin] (EC 1.8.4.8)  histidine kinase  hypothetical protein  hypothetical protein  hypothetical protein |
| 13766 | AGHY01000361.1:1..13766 | BFD-like (2Fe-2S)-binding region  Endoribonuclease L-PSP  NAD/NADP octopine/nopaline dehydrogenase  Opine oxidase subunit B  Programmed cell death toxin MazF like  Pyruvate/2-oxoglutarate dehydrogenase complex2C dihydrolipoamide acyltransferase (E2) component2C and related enzymes  hypothetical protein  hypothetical protein  protein of unknown function DUF62C transmembrane  putative exported protein  transcriptional regulator2C LysR family  transcriptional regulator2C LysR family |
| 12822 | AGHY01000070.1:1..12822 | Excinuclease ABC subunit B  Excinuclease ABC subunit B  FIG01211666: hypothetical protein  Forms the bulk of type IV secretion complex that spans outer membrane and periplasm (VirB9)  Inner membrane protein forms channel for type IV secretion of T-DNA complex (VirB8)  Type IV fimbrial biogenesis protein PilX  Type IV fimbrial biogenesis protein PilY1  Type IV fimbrial biogenesis protein PilY1  Type IV fimbrial biogenesis protein PilY1  Type IV pilus biogenesis protein PilE  Type IV secretion system protein VirD4  hypothetical protein  tRNA-Asn-GTT  tRNA-Val-GAC  type 4 fimbrial biogenesis protein |
| 10535 | AGHY01001281.1:1..10535 | ATPase provides energy for both assembly of type IV secretion complex and secretion of T-DNA complex (VirB4)  ATPase provides energy for both assembly of type IV secretion complex and secretion of T-DNA complex (VirB4)  FIG01213967: hypothetical protein  Inner membrane protein forms channel for type IV secretion of T-DNA complex (VirB3)  Integral inner membrane protein of type IV secretion complex (VirB6)  Major pilus subunit of type IV secretion complex2C VirB2  Maltodextrin glucosidase (EC 3.2.1.20)  TonB-dependent receptor |
| 9472 | AGHY01000042.1:1..9472 | Putative phage replication protein RstA  Zonular occludens toxin  hypothetical protein  hypothetical protein  hypothetical protein  hypothetical protein  hypothetical protein  hypothetical protein  hypothetical protein  hypothetical protein  hypothetical protein  hypothetical protein  hypothetical protein  two-component system sensor protein |
| 9163 | AGHY01000267.1:1..9163 | hypothetical protein  hypothetical protein  hypothetical protein |
| 8895 | AGHY01000103.1:1..8895 | FIG00961797: hypothetical protein  Phage tail assembly protein I  hypothetical protein  hypothetical protein  hypothetical protein  hypothetical protein  hypothetical protein  hypothetical protein  hypothetical protein  hypothetical protein  hypothetical protein  hypothetical protein  hypothetical protein  hypothetical protein  hypothetical protein  predicted transcriptional regulator |
| 8459 | AGHY01000031.1:1..8459 | Flp pilus assembly protein RcpC/CpaB  Flp pilus assembly protein TadB  Type II/IV secretion system ATP hydrolase TadA/VirB11/CpaF2C TadA subfamily  Type II/IV secretion system ATP hydrolase TadA/VirB11/CpaF2C TadA subfamily  Type II/IV secretion system ATP hydrolase TadA/VirB11/CpaF2C TadA subfamily  Type II/IV secretion system ATP hydrolase TadA/VirB11/CpaF2C TadA subfamily  Type II/IV secretion system protein TadC2C associated with Flp pilus assembly  hypothetical protein  hypothetical protein  hypothetical protein  hypothetical protein  type II and III secretion system protein |
| 8203 | AGHY01000173.1:1..8203 | 3-oxoacyl-[acyl-carrier protein] reductase (EC 1.1.1.100)  3-oxoacyl-[acyl-carrier protein] reductase (EC 1.1.1.100)  FIG01215019: hypothetical protein  Uncharacterized protein conserved in bacteria2C NMA0228-like  hypothetical protein  hypothetical protein |
| 8050 | AGHY01000157.1:1..8050 | DNA polymerase III alpha subunit (EC 2.7.7.7)  DNA polymerase III alpha subunit (EC 2.7.7.7)  DNA polymerase-like protein PA0670  Integrase  hypothetical protein  hypothetical protein |
| 7814 | AGHY01000011.1:1..7814 | Bifunctional protein: zinc-containing alcohol dehydrogenase; quinone oxidoreductase ( NADPH:quinone reductase) (EC 1.1.1.-); Similar to arginate lyase  Chemotaxis protein methyltransferase CheR (EC 2.1.1.80)  Chemotaxis protein methyltransferase CheR (EC 2.1.1.80)  FIG00452947: hypothetical protein  Glycine betaine-binding protein  Thioredoxin reductase (EC 1.8.1.9)  Transcriptional regulator2C LysR family |
| 7657 | AGHY01000045.1:1..7657 | hypothetical protein  hypothetical protein  hypothetical protein  hypothetical protein  hypothetical protein  hypothetical protein  hypothetical protein  hypothetical protein  hypothetical protein  hypothetical protein  hypothetical protein  putative DNA transposition protein  transposase A  transposase2C putative |
| 7629 | AGHY01000346.1:1..7629 | NADH-ubiquinone oxidoreductase chain H (EC 1.6.5.3)  NADH-ubiquinone oxidoreductase chain H (EC 1.6.5.3)  NADH-ubiquinone oxidoreductase chain I (EC 1.6.5.3)  NADH-ubiquinone oxidoreductase chain J (EC 1.6.5.3)  NADH-ubiquinone oxidoreductase chain K (EC 1.6.5.3)  NADH-ubiquinone oxidoreductase chain L (EC 1.6.5.3)  NADH-ubiquinone oxidoreductase chain M (EC 1.6.5.3)  NADH-ubiquinone oxidoreductase chain M (EC 1.6.5.3) |
| 7541 | AGHY01001204.1:1..7541 | Acetylornithine aminotransferase (EC 2.6.1.11)  Acetylornithine aminotransferase (EC 2.6.1.11)  Ferrichrome-iron receptor  Ferrichrome-iron receptor  Iron-uptake factor PiuC  Transcriptional regulator2C AsnC family  hypothetical protein |
| 6987 | AGHY01000220.1:1..6987 | 2-oxoglutarate dehydrogenase E1 component (EC 1.2.4.2)  Dihydrolipoamide succinyltransferase component (E2) of 2-oxoglutarate dehydrogenase complex (EC 2.3.1.61)  Metallopeptidase  hypothetical protein |
| 6949 | AGHY01000845.1:1..6949 | Carbon storage regulator  Glycogen debranching enzyme (EC 3.2.1.-)  hypothetical protein  hypothetical protein  hypothetical protein  putative; ORF located using Glimmer/Genemark  tRNA-Ser-GCT |
| 6910 | AGHY01000028.1:1..6910 | FIG01212275: hypothetical protein  O-antigen export system permease protein RfbD  Phytoene desaturase (EC 1.14.99.-)  Phytoene desaturase (EC 1.14.99.-)  Putative transmembrane oxidoreductase protein  putative; ORF located using Glimmer/Genemark |
| 6870 | AGHY01000007.1:1..6870 | Mu-like prophage FluMu protein gp29  Phage (Mu-like) virion morphogenesis protein  Phage terminase2C large subunit  hypothetical protein  hypothetical protein  hypothetical protein  hypothetical protein  protease (I) and scaffold (Z) proteins |
| 6803 | AGHY01000128.1:1..6803 | 3-oxoacyl-[acyl-carrier-protein] synthase2C KASIII (EC 2.3.1.41)  Acetyltransferase  Acetyltransferase (isoleucine patch superfamily)  Acyl carrier protein  Aminotransferase2C DegT/DnrJ/EryC1/StrS family  GbcA Glycine betaine demethylase subunit A  Oxidoreductase2C short chain dehydrogenase/reductase family  short chain dehydrogenase |
| 6754 | AGHY01000052.1:1..6754 | Inosose isomerase (EC 5.3.99.-)  Putative nucleoside transporter yegT  Putative nucleoside transporter yegT  Transcriptional (co)regulator CytR  hypothetical protein  hypothetical protein  prolyl oligopeptidase family protein |
| 6587 | AGHY01000071.1:1..6587 | Catalase (EC 1.11.1.6)  Glycine dehydrogenase [decarboxylating] (glycine cleavage system P protein) (EC 1.4.4.2)  Membrane-associated phospholipid phosphatase  TolA protein  Uncharacterized zinc-type alcohol dehydrogenase-like protein ybdR  hypothetical protein |
| 6570 | AGHY01000704.1:1..6570 | NADH ubiquinone oxidoreductase chain A (EC 1.6.5.3)  NADH-ubiquinone oxidoreductase chain B (EC 1.6.5.3)  NADH-ubiquinone oxidoreductase chain C (EC 1.6.5.3)  NADH-ubiquinone oxidoreductase chain D (EC 1.6.5.3)  NADH-ubiquinone oxidoreductase chain D (EC 1.6.5.3)  NADH-ubiquinone oxidoreductase chain E (EC 1.6.5.3)  NADH-ubiquinone oxidoreductase chain F (EC 1.6.5.3)  NADH-ubiquinone oxidoreductase chain G (EC 1.6.5.3)  tRNA-Leu-GAG |
| 6319 | AGHY01000232.1:1..6319 | 4-deoxy-L-threo-5-hexosulose-uronate ketol-isomerase (EC 5.3.1.17)  Phenylalanine-4-hydroxylase (EC 1.14.16.1)  Phenylalanine-4-hydroxylase (EC 1.14.16.1)  Probable lipoprotein  Putative lipoprotein  hypothetical protein  hypothetical protein  predicted 4-deoxy-L-threo-5-hexosulose-uronate ketol-isomerase (EC 5.3.1.17)  putative lipoprotein |
| 6315 | AGHY01000124.1:1..6315 | 2-keto-3-deoxy-D-arabino-heptulosonate-7-phosphate synthase II (EC 2.5.1.54)  FIG01210215: hypothetical protein  Membrane protein  arabinofuranosidase  hypothetical protein  hypothetical protein |
| 6247 | AGHY01000288.1:1..6247 | ATP-dependent helicase DinG/Rad3  ATP-dependent helicase DinG/Rad3  Membrane-fusion protein  colicin V secretion ABC transporter ATP-binding protein  colicin V secretion ABC transporter ATP-binding protein  hypothetical protein |
| 6093 | AGHY01000092.1:1..6093 | Alpha-12C2-mannosidase  TonB-dependent receptor  TonB-dependent receptor  hypothetical protein  hypothetical protein |
| 5997 | AGHY01000419.1:1..5997 | Agmatine deiminase (EC 3.5.3.12)  Cytidylate kinase (EC 2.7.4.14)  Integration host factor beta subunit  LSU ribosomal protein L36p  SSU ribosomal protein S1p  hypothetical protein  hypothetical protein |
| 5980 | AGHY01000090.1:1..5980 | ATP synthase A chain (EC 3.6.3.14)  ATP synthase B chain (EC 3.6.3.14)  ATP synthase C chain (EC 3.6.3.14)  ATP synthase delta chain (EC 3.6.3.14)  Dihydrolipoamide dehydrogenase of pyruvate dehydrogenase complex (EC 1.8.1.4)  FIG01210264: hypothetical protein  hypothetical protein |
| 5910 | AGHY01000663.1:1..5910 | Butyryl-CoA dehydrogenase (EC 1.3.99.2)  TonB-dependent receptor  TonB-dependent receptor  TonB-dependent receptor  Transcriptional regulator2C TetR family  alpha/beta hydrolase fold |
| 5902 | AGHY01000705.1:1..5902 | Cystathionine beta-synthase (EC 4.2.1.22)  Cystathionine beta-synthase (EC 4.2.1.22)  Cystathionine gamma-lyase (EC 4.4.1.1)  Glycosyl transferase2C family 2  Glycosyl transferase2C family 2  UptF protein  outer membrane protein |
| 5878 | AGHY01000178.1:1..5878 | ATP synthase alpha chain (EC 3.6.3.14)  ATP synthase alpha chain (EC 3.6.3.14)  ATP synthase beta chain (EC 3.6.3.14)  ATP synthase beta chain (EC 3.6.3.14)  ATP synthase epsilon chain (EC 3.6.3.14)  ATP synthase gamma chain (EC 3.6.3.14)  ATP synthase gamma chain (EC 3.6.3.14)  Periplasmic chorismate mutase I precursor (EC 5.4.99.5) |
| 5841 | AGHY01000407.1:1..5841 | DNA-directed RNA polymerase beta subunit (EC 2.7.7.6)  DNA-directed RNA polymerase beta subunit (EC 2.7.7.6)  DNA-directed RNA polymerase beta subunit (EC 2.7.7.6)  DNA-directed RNA polymerase beta subunit (EC 2.7.7.6)  hypothetical protein |
| 5837 | AGHY01000596.1:1..5837 | Lysyl endopeptidase (EC 3.4.21.50)  general stress protein  hypothetical protein  hypothetical protein  hypothetical protein  lipase2C class 3 |
| 5719 | AGHY01000034.1:1..5719 | ABC-type multidrug transport system2C ATPase component  hypothetical protein  hypothetical protein  hypothetical protein  hypothetical protein  permease  phenol hydroxylase |
| 5630 | AGHY01000020.1:1..5630 | TonB-dependent receptor  hypothetical protein  similar to mannose-6-phosphate isomerase/mannose-1-phosphate guanylyl transferase( EC:2.7.7.132CEC:5.3.1.8 ) |
| 5608 | AGHY01000369.1:1..5608 | Chaperone protein DnaJ  Chaperone protein DnaJ  Chaperone protein DnaJ  Chaperone protein DnaK  Heat shock protein GrpE  Heat-inducible transcription repressor HrcA |
| 5570 | AGHY01000284.1:1..5570 | Glutamyl-tRNA synthetase (EC 6.1.1.17)  Putative ribonucleoprotein related-protein  Transcriptional regulatory protein RtcR  Zinc uptake regulation protein ZUR |
| 5446 | AGHY01000331.1:1..5446 | Oxidoreductase  Transcriptional regulator2C TetR family  major facilitator family transporter |
| 5426 | AGHY01000166.1:1..5426 | GCN5-related N-acetyltransferase  LysR family transcriptional regulator STM3121  SAM-dependent methyltransferase BA1462 (UbiE paralog)  Transcriptional regulator2C LysR family  hypothetical protein  hypothetical protein  monooxygenase2C FAD-binding  transcriptional regulator2C MarR family |
| 5400 | AGHY01000344.1:1..5400 | DNA-directed RNA polymerase beta' subunit (EC 2.7.7.6)  SSU ribosomal protein S12p (S23e)  SSU ribosomal protein S7p (S5e) |
| 5396 | AGHY01000861.1:1..5396 | 4-hydroxybenzoyl-CoA thioesterase family active site  Holliday junction DNA helicase RuvB  MotA/TolQ/ExbB proton channel family protein  OmpA/MotB  Outer membrane lipoprotein omp16 precursor  Tol biopolymer transport system2C TolR protein  TolA protein  tolB protein precursor2C periplasmic protein involved in the tonb-independent uptake of group A colicins |
| 5384 | AGHY01000048.1:1..5384 | DNA gyrase subunit B (EC 5.99.1.3)  DNA polymerase III beta subunit (EC 2.7.7.7)  DNA recombination and repair protein RecF  DNA recombination and repair protein RecF  hypothetical protein  hypothetical protein |
| 5236 | AGHY01000356.1:1..5236 | Beta-galactosidase (EC 3.2.1.23)  Maltodextrin glucosidase (EC 3.2.1.20) |
| 5188 | AGHY01000119.1:1..5188 | Biotin carboxylase (EC 6.3.4.14)  Biotin carboxylase (EC 6.3.4.14)  Hydrolase or peptidase  Sulfate permease  hypothetical protein  hypothetical protein  hypothetical protein |
| 5154 | AGHY01001220.1:1..5154 | Ethidium bromide-methyl viologen resistance protein EmrE  Periplasmic aromatic aldehyde oxidoreductase2C iron-sulfur subunit YagT  TonB-dependent receptor  Zinc-regulated outer membrane receptor  hypothetical protein  hypothetical protein  hypothetical protein |
| 5154 | AGHY01000771.1:1..5154 | Adenylosuccinate synthetase (EC 6.3.4.4)  hypothetical protein  hypothetical protein  hypothetical protein |
| 5149 | AGHY01000013.1:1..5149 | Beta-galactosidase (EC 3.2.1.23)  hypothetical protein |
| 5146 | AGHY01000498.1:1..5146 | FIG01111510: hypothetical protein  FIG01112671: hypothetical protein  Glucose-methanol-choline (GMC) oxidoreductase:NAD binding site  Glucose-methanol-choline (GMC) oxidoreductase:NAD binding site  Glucose-methanol-choline (GMC) oxidoreductase:NAD binding site  Hydroxypyruvate isomerase (EC 5.3.1.22)  Xylose isomerase-like TIM barrel  hypothetical protein  putative multi-domain protein |
| 5141 | AGHY01000821.1:1..5141 | Cation:proton antiporter  Cation:proton antiporter  hypothetical protein  hypothetical protein  peptidase M192C renal dipeptidase  putative; ORF located using Glimmer/Genemark  transcriptional regulator |
| 5128 | AGHY01000378.1:1..5128 | Chemotaxis protein CheV (EC 2.7.3.-)  Chemotaxis protein CheV (EC 2.7.3.-)  Flagellar basal-body rod modification protein FlgD  Flagellar basal-body rod protein FlgB  Flagellar basal-body rod protein FlgC  Flagellar basal-body rod protein FlgC  Flagellar basal-body rod protein FlgF  Flagellar hook protein FlgE  Flagellar hook protein FlgE |
| 5055 | AGHY01000432.1:1..5055 | Cellulase  Similarity with glutathionylspermidine synthase (EC 6.3.1.8)2C group 1  conserved hypothetical protein |
| 4996 | AGHY01000203.1:1..4996 | N-acetylglucosamine-regulated TonB-dependent outer membrane receptor  hypothetical protein |
| 4984 | AGHY01000339.1:1..4984 | FIG01209954: hypothetical protein  Nicotinamide-nucleotide adenylyltransferase2C NadM family (EC 2.7.7.1) / ADP-ribose pyrophosphatase (EC 3.6.1.13)  arabinogalactan endo-12C4-beta-galactosidase |
| 4963 | AGHY01000054.1:1..4963 | VgrG protein  hypothetical protein  hypothetical protein |
| 4941 | AGHY01000818.1:1..4941 | Protein-disulfide isomerase  Type IV secretory pathway2C VirB4 components |
| 4918 | AGHY01000260.1:1..4918 | Protein export cytoplasm protein SecA ATPase RNA helicase (TC 3.A.5.1.1)  UDP-3-O-[3-hydroxymyristoyl] N-acetylglucosamine deacetylase (EC 3.5.1.-)  peptidase |
| 4908 | AGHY01001114.1:1..4908 | Glucan 12C4-beta-glucosidase  Glucan 12C4-beta-glucosidase  Glucan 12C4-beta-glucosidase  Xylosidase/arabinosidase  transport protein |
| 4885 | AGHY01000063.1:1..4885 | conserved hypothetical protein  hypothetical protein  hypothetical protein  hypothetical protein  hypothetical protein  hypothetical protein  transcriptional regulator2C LysR family |
| 4884 | AGHY01000320.1:1..4884 | Chromosome partitioning ATPase in PFGI-1-like cluster2C ParA-like  FIG141751: hypothetical protein in PFGI-1-like cluster  Protein with ParB-like nuclease domain in PFGI-1-like cluster  Transcriptional regulator in PFGI-1-like cluster |
| 4784 | AGHY01000152.1:1..4784 | Dihydroneopterin aldolase (EC 4.1.2.25)  Protein containing domains DUF403  Protein containing domains DUF4042C DUF407  Small-conductance mechanosensitive channel  hypothetical protein |
| 4774 | AGHY01000069.1:1..4774 | hypothetical protein  hypothetical protein  hypothetical protein |
| 4761 | AGHY01000585.1:1..4761 | FIG01209870: hypothetical protein  FIG01211750: hypothetical protein  Glycerol-3-phosphate dehydrogenase [NAD(P) ] (EC 1.1.1.94)  Transcriptional regulator2C AraC family  conserved hypothetical protein  tetR-family transcriptional regulatory protein |
| 4716 | AGHY01000900.1:1..4716 | LigA  RecA protein  RecA protein  Regulatory protein RecX  SOS-response repressor and protease LexA (EC 3.4.21.88)  hypothetical protein  hypothetical protein |
| 4705 | AGHY01001045.1:1..4705 | hypothetical protein |
| 4683 | AGHY01000018.1:1..4683 | Pass1-related protein  Pass1-related protein  Peptide transport system permease protein sapC (TC 3.A.1.5.5)  TonB-dependent receptor  Tryptophan halogenase |
| 4675 | AGHY01001427.1:1..4675 | Alpha-glucosidase (EC 3.2.1.20)  Alpha-glucosidase (EC 3.2.1.20)  TonB-dependent receptor |
| 4673 | AGHY01000833.1:1..4673 | ABC transporter ATP-binding protein USSDB6B  ABC-type transport system involved in resistance to organic solvents2C periplasmic component USSDB6C  ABC-type transport system involved in resistance to organic solvents2C permease component USSDB6A  Membrane lipoprotein lipid attachment site containing protein USSDB6D  hypothetical protein |
| 4574 | AGHY01000433.1:1..4574 | Glutamate synthase [NADPH] large chain (EC 1.4.1.13)  Glutamate synthase [NADPH] large chain (EC 1.4.1.13) |
| 4569 | AGHY01000686.1:1..4569 | Carbamoyl-phosphate synthase large chain (EC 6.3.5.5)  Carbamoyl-phosphate synthase small chain (EC 6.3.5.5)  hypothetical protein |
| 4568 | AGHY01000263.1:1..4568 | CTP synthase (EC 6.3.4.2)  CTP synthase (EC 6.3.4.2)  Topoisomerase IV subunit B (EC 5.99.1.-)  hypothetical protein |
| 4532 | AGHY01000591.1:1..4532 | Acetyl-coenzyme A synthetase (EC 6.2.1.1)  FIG01213271: hypothetical protein  FIG01213271: hypothetical protein  hypothetical protein |
| 4486 | AGHY01001260.1:1..4486 | Nucleoside diphosphate kinase (EC 2.7.4.6)  Predicted transcriptional regulator for fatty acid degradation FadQ2C TetR family  Ribosomal RNA large subunit methyltransferase N (EC 2.1.1.-)  Ribosomal RNA large subunit methyltransferase N (EC 2.1.1.-)  hypothetical protein |
| 4467 | AGHY01000029.1:1..4467 | 3-oxoacyl-[acyl-carrier-protein] synthase2C KASIII in hypothetical gene cluster  3-oxoacyl-[acyl-carrier-protein] synthase2C KASIII in hypothetical gene cluster  FIG01210420: hypothetical protein  FIG01212698: hypothetical protein  Hydrolase2C alpha/beta fold family protein2C in hypothetical gene cluster  hypothetical protein |
| 4456 | AGHY01000484.1:1..4456 | FIG01002316: hypothetical protein  FIG01211682: hypothetical protein  Glucosamine--fructose-6-phosphate aminotransferase [isomerizing] (EC 2.6.1.16)  Glucosamine--fructose-6-phosphate aminotransferase [isomerizing] (EC 2.6.1.16)  putative oxidoreductase |
| 4428 | AGHY01000149.1:1..4428 | Stringent starvation protein A  Ubiquinol--cytochrome c reductase2C cytochrome B subunit (EC 1.10.2.2)  Ubiquinol-cytochrome C reductase iron-sulfur subunit (EC 1.10.2.2)  soluble lytic murein transglycosylase  ubiquinol cytochrome C oxidoreductase2C cytochrome C1 subunit |
| 4384 | AGHY01000529.1:1..4384 | avirulence protein  avirulence protein  hypothetical protein  hypothetical protein |
| 4374 | AGHY01000471.1:1..4374 | FIG01209976: hypothetical protein  FIG01209976: hypothetical protein  L-threonine 3-dehydrogenase (EC 1.1.1.103)  hypothetical protein |
| 4354 | AGHY01000192.1:1..4354 | N-acetylglucosamine-regulated TonB-dependent outer membrane receptor  hypothetical protein |
| 4279 | AGHY01000530.1:1..4279 | FIG01211190: hypothetical protein  FIG01213552: hypothetical protein  FIG01214187: hypothetical protein  FIG076210: Hypothetical protein  FIG076676: Hypothetical protein |
| 4271 | AGHY01000116.1:1..4271 | Acetyl-CoA:acetoacetyl-CoA transferase2C beta subunit (EC 2.8.3.8)  FIG01212420: hypothetical protein  FIG01212420: hypothetical protein  Succinyl-CoA:3-ketoacid-coenzyme A transferase subunit A (EC 2.8.3.5) |
| 4244 | AGHY01000117.1:1..4244 | Extracellular ribonuclease precursor (EC 3.1.-.-)  Heat shock protein 60 family chaperone GroEL  Heat shock protein 60 family co-chaperone GroES |
| 4232 | AGHY01000274.1:1..4232 | FIG01209964: hypothetical protein  Glycerol-3-phosphate acyltransferase (EC 2.3.1.15)  Glycerol-3-phosphate acyltransferase (EC 2.3.1.15)  hypothetical protein  hypothetical protein |
| 4217 | AGHY01002616.1:1..4217 | Methyl-directed repair DNA adenine methylase (EC 2.1.1.72)  Methyl-directed repair DNA adenine methylase (EC 2.1.1.72)  hypothetical protein  hypothetical protein  hypothetical protein |
| 4196 | AGHY01000293.1:1..4196 | COG1451: Predicted metal-dependent hydrolase  lipolytic enzyme2C G-D-S-L  polyvinylalcohol dehydrogenase  tRNA pseudouridine synthase A (EC 4.2.1.70) |
| 4191 | AGHY01000329.1:1..4191 | FIG01211080: hypothetical protein  putative Glutathione-regulated potassium-efflux system protein KefB  sensor kinase  sensor kinase |
| 4172 | AGHY01000285.1:1..4172 | FKBP-type peptidyl-prolyl cis-trans isomerase FkpA precursor (EC 5.2.1.8)  FKBP-type peptidyl-prolyl cis-trans isomerase FkpA precursor (EC 5.2.1.8)  Glutathione peroxidase (EC 1.11.1.9)  Glutathione peroxidase (EC 1.11.1.9)  UDP-glucose dehydrogenase (EC 1.1.1.22)  transcriptional regulator gntR family |
| 4169 | AGHY01000112.1:1..4169 | Conjugative transfer protein TrbI  hypothetical protein  tRNA-Leu-TAA |
| 4128 | AGHY01000482.1:1..4128 | Fucose permease  Possible alternative L-fucose mutarotase  Transcriptional regulator2C IclR family  hypothetical protein  hypothetical protein |
| 4128 | AGHY01000387.1:1..4128 | FIG01209781: hypothetical protein  Iron-sulfur cluster regulator IscR  Proteins containing SET domain  ThiJ/PfpI family protein |
| 4116 | AGHY01000641.1:1..4116 | Lipoate synthase  Octanoate-[acyl-carrier-protein]-protein-N-octanoyltransferase  tail-specific protease  tail-specific protease |
| 4115 | AGHY01000397.1:1..4115 | LSU ribosomal protein L22p (L17e)  LSU ribosomal protein L23p (L23Ae)  LSU ribosomal protein L2p (L8e)  LSU ribosomal protein L2p (L8e)  LSU ribosomal protein L3p (L3e)  LSU ribosomal protein L4p (L1e)  LSU ribosomal protein L4p (L1e)  SSU ribosomal protein S10p (S20e)  SSU ribosomal protein S19p (S15e) |
| 4110 | AGHY01000449.1:1..4110 | 2-methylaconitate isomerase  2-methylcitrate dehydratase FeS dependent (EC 4.2.1.79)  2-methylcitrate dehydratase FeS dependent (EC 4.2.1.79)  Uncharacterized conserved protein  hypothetical protein |
| 4084 | AGHY01001420.1:1..4084 | FIG01212717: hypothetical protein  hypothetical protein |
| 4067 | AGHY01000724.1:1..4067 | Inorganic pyrophosphatase (EC 3.6.1.1)  Pyrophosphate-energized proton pump (EC 3.6.1.1)  hypothetical protein  response regulator  response regulator |
| 4053 | AGHY01000851.1:1..4053 | FIG016519: Putative DNA-binding protein  GTP-binding protein EngA  Mlr7403 protein  Outer membrane protein YfgL2C lipoprotein component of the protein assembly complex (forms a complex with YaeT2C YfiO2C and NlpB)  Outer membrane protein YfgL2C lipoprotein component of the protein assembly complex (forms a complex with YaeT2C YfiO2C and NlpB) |
| 4048 | AGHY01000499.1:1..4048 | Succinate dehydrogenase cytochrome b-556 subunit  Succinate dehydrogenase flavoprotein subunit (EC 1.3.99.1)  Succinate dehydrogenase hydrophobic membrane anchor protein  Succinate dehydrogenase iron-sulfur protein (EC 1.3.99.1) |
| 4042 | AGHY01000318.1:1..4042 | Autolysin sensor kinase (EC 2.7.3.-)  hypothetical protein  hypothetical protein  hypothetical protein  probable two-component response regulator transcription protein |
| 4035 | AGHY01000332.1:1..4035 | ATP-dependent Clp protease ATP-binding subunit ClpA  ATP-dependent Clp protease adaptor protein ClpS  Translation initiation factor 1  conserved hypothetical protein  hypothetical protein |
| 4024 | AGHY01000102.1:1..4024 | FIG01211168: hypothetical protein  PROBABLE LIPOPROTEIN TRANSMEMBRANE  hypothetical protein  hypothetical protein  hypothetical protein  putative; ORF located using Glimmer/Genemark |
| 4020 | AGHY01000706.1:1..4020 | Histidine kinase/response regulator hybrid protein  Phytochrome2C two-component sensor histidine kinase (EC 2.7.3.-)  Phytochrome2C two-component sensor histidine kinase (EC 2.7.3.-)  hypothetical protein  hypothetical protein  two-component system regulatory protein |
| 4009 | AGHY01001096.1:1..4009 | UDP-N-acetylglucosamine 42C6-dehydratase (EC 4.2.1.-)  UDP-N-acetylglucosamine 42C6-dehydratase (EC 4.2.1.-)  UDP-N-acetylglucosamine 42C6-dehydratase (EC 4.2.1.-)  UTP--glucose-1-phosphate uridylyltransferase (EC 2.7.7.9)  lipopolysaccharide core biosynthesis protein |
| 4001 | AGHY01000123.1:1..4001 | N-acetyl glucosamine transporter2C NagP  N-acetylglucosamine related transporter2C NagX  Predicted transcriptional regulator of N-Acetylglucosamine utilization2C GntR family |
| 3971 | AGHY01000823.1:1..3971 | Ribonucleotide reductase of class II (coenzyme B12-dependent) (EC 1.17.4.1)  Ribonucleotide reductase of class II (coenzyme B12-dependent) (EC 1.17.4.1) |
| 3968 | AGHY01000299.1:1..3968 | Outer membrane protein Imp2C required for envelope biogenesis / Organic solvent tolerance protein precursor  acetoin utilization family protein |
| 3958 | AGHY01000767.1:1..3958 | FIG01209733: hypothetical protein  FIG01210804: hypothetical protein  Glutaredoxin 3  Isocitrate dehydrogenase [NAD] (EC 1.1.1.41)  tRNA-Arg-TCT  tRNA-Pro-TGG |
| 3943 | AGHY01000286.1:1..3943 | endolysin  hypothetical protein  hypothetical protein  hypothetical protein  hypothetical protein  hypothetical protein  hypothetical protein  hypothetical protein  small terminase subunit |
| 3930 | AGHY01000380.1:1..3930 |  |
| 3917 | AGHY01000068.1:1..3917 | FIG01212167: hypothetical protein  hypothetical protein  hypothetical protein  photolyase protein family |
| 3909 | AGHY01000675.1:1..3909 | 3-hydroxydecanoyl-[acyl-carrier-protein] dehydratase (EC 4.2.1.60)  3-oxoacyl-[acyl-carrier-protein] synthase2C KASI (EC 2.3.1.41)  DNA polymerase IV (EC 2.7.7.7)  DNA polymerase IV (EC 2.7.7.7) |
| 3905 | AGHY01001441.1:1..3905 | Uncharacterized protein conserved in bacteria  Uncharacterized protein conserved in bacteria  hypothetical protein  hypothetical protein  hypothetical protein  hypothetical protein  hypothetical protein  hypothetical protein |
| 3899 | AGHY01000535.1:1..3899 | hypothetical protein  lipoprotein2C putative  lipoprotein2C putative |
| 3880 | AGHY01002520.1:1..3880 | Large exoproteins involved in heme utilization or adhesion  Large exoproteins involved in heme utilization or adhesion  filamentous haemagglutinin family outer membrane protein |
| 3804 | AGHY01000441.1:1..3804 | S-(hydroxymethyl)glutathione dehydrogenase (EC 1.1.1.284)  surface antigen gene |
| 3762 | AGHY01000420.1:1..3762 | Phosphoribosylaminoimidazole-succinocarboxamide synthase (EC 6.3.2.6)  hypothetical protein  hypothetical protein  hypothetical protein |
| 3752 | AGHY01000913.1:1..3752 | FIG01210744: hypothetical protein  TonB-dependent receptor  TonB-dependent receptor |
| 3726 | AGHY01000076.1:1..3726 | FIG00956406: hypothetical protein  FIG01210890: hypothetical protein  FIG01214923: hypothetical protein  FIG01214923: hypothetical protein |
| 3725 | AGHY01000182.1:1..3725 | Di-/tripeptide transporter  Di-/tripeptide transporter |
| 3716 | AGHY01000405.1:1..3716 | Cardiolipin synthetase (EC 2.7.8.-)  Endonuclease/exonuclease/phosphatase  PDZ domain family protein  endonuclease/exonuclease/phosphatase family protein  hypothetical protein  hypothetical protein |
| 3716 | AGHY01000214.1:1..3716 | Aspartate/tyrosine/aromatic aminotransferase  Prolyl endopeptidase (EC 3.4.21.26)  hypothetical acetyltransferase  putative prolyl oligopeptidase precursor |
| 3703 | AGHY01000699.1:1..3703 | FIG01211097: hypothetical protein  INTEGRAL MEMBRANE PROTEIN (Rhomboid family)  Long-chain fatty acid transport protein  Proline dehydrogenase (EC 1.5.99.8) (Proline oxidase) / Delta-1-pyrroline-5-carboxylate dehydrogenase (EC 1.5.1.12)  Proline dehydrogenase (EC 1.5.99.8) (Proline oxidase) / Delta-1-pyrroline-5-carboxylate dehydrogenase (EC 1.5.1.12) |
| 3699 | AGHY01000431.1:1..3699 | ATP/GTP-binding protein  FIG137360: hypothetical protein  Phosphoenolpyruvate synthase (EC 2.7.9.2) |
| 3697 | AGHY01000666.1:1..3697 | DNA-directed RNA polymerase omega subunit (EC 2.7.7.6)  GTP pyrophosphokinase (EC 2.7.6.5)2C (p)ppGpp synthetase II / Guanosine-3'2C5'-bis(diphosphate) 3'-pyrophosphohydrolase (EC 3.1.7.2)  Guanylate kinase (EC 2.7.4.8) |
| 3694 | AGHY01000229.1:1..3694 | FIG00959623: hypothetical protein  hypothetical protein |
| 3689 | AGHY01000886.1:1..3689 | FIG01111765: hypothetical protein  hypothetical protein  hypothetical protein  hypothetical protein  hypothetical protein |
| 3686 | AGHY01000212.1:1..3686 | Cell division protein FtsH (EC 3.4.24.-)  Cell division protein FtsJ / Ribosomal RNA large subunit methyltransferase E (EC 2.1.1.-)  FIG004454: RNA binding protein  hypothetical protein |
| 3683 | AGHY01000027.1:1..3683 | FIG01211164: hypothetical protein  Twin-arginine translocation protein TatC  glutamyl endopeptidase  hypothetical protein  hypothetical protein |
| 3637 | AGHY01000306.1:1..3637 | TonB-dependent receptor  hypothetical protein |
| 3633 | AGHY01001653.1:1..3633 | Ribosomal protein S6 glutaminyl transferase  Two-component system regulatory protein  two-component system sensor protein |
| 3607 | AGHY01000987.1:1..3607 | Acetyltransferase2C GNAT family (EC 2.3.1.-)  FIG01210523: hypothetical protein  Microcystin dependent protein  Microcystin dependent protein  Microcystin dependent protein |
| 3607 | AGHY01000012.1:1..3607 | Catalase (EC 1.11.1.6)  Protein yciF  hypothetical protein  hypothetical protein |
| 3602 | AGHY01000691.1:1..3602 | Threonyl-tRNA synthetase (EC 6.1.1.3)  Threonyl-tRNA synthetase (EC 6.1.1.3)  Translation initiation factor 3 |
| 3577 | AGHY01000249.1:1..3577 | Glucoamylase (EC 3.2.1.3)  Glucoamylase (EC 3.2.1.3) |
| 3564 | AGHY01001111.1:1..3564 | Decarboxylase family protein  Oar protein  TonB-dependent receptor  alginate biosynthesis protein |
| 3563 | AGHY01000477.1:1..3563 | FIG01112335: hypothetical protein  hypothetical protein  hypothetical protein  hypothetical protein  hypothetical protein  hypothetical protein |
| 3563 | AGHY01000148.1:1..3563 | Ribonuclease BN (EC 3.1.-.-)  SSU ribosomal protein S21p  Transamidase GatB domain protein |
| 3562 | AGHY01000895.1:1..3562 | Excinuclease ABC subunit A  LSU ribosomal protein L21p  LSU ribosomal protein L27p |
| 3536 | AGHY01000091.1:1..3536 | Cytochrome O ubiquinol oxidase subunit I (EC 1.10.3.-)  Cytochrome O ubiquinol oxidase subunit I (EC 1.10.3.-)  Cytochrome O ubiquinol oxidase subunit III (EC 1.10.3.-)  Cytochrome O ubiquinol oxidase subunit IV (EC 1.10.3.-)  Transcriptional regulator2C MarR family |
| 3535 | AGHY01000239.1:1..3535 | Gamma-glutamyltranspeptidase (EC 2.3.2.2)  choline/carnitine/betaine transporter  hypothetical protein |
| 3531 | AGHY01000738.1:1..3531 | Phosphohydrolase (MutT/nudix family protein)  hypothetical protein  hypothetical protein |
| 3522 | AGHY01001587.1:1..3522 | Aconitate hydratase 2 (EC 4.2.1.3)  FIG01210619: hypothetical protein  FIG01211894: hypothetical protein |
| 3517 | AGHY01000250.1:1..3517 | Histone acetyltransferase HPA2 and related acetyltransferases  Outer membrane vitamin B12 receptor BtuB  Ribosomal protein S4 and related proteins  Transcriptional regulator2C LysR family |
| 3514 | AGHY01000586.1:1..3514 | Cobalt-zinc-cadmium resistance protein CzcA; Cation efflux system protein CusA  Cobalt-zinc-cadmium resistance protein CzcA; Cation efflux system protein CusA  Cobalt-zinc-cadmium resistance protein CzcA; Cation efflux system protein CusA |
| 3496 | AGHY01000132.1:1..3496 | Cytochrome c oxidase polypeptide II (EC 1.9.3.1)  FIG01209735: hypothetical protein  Proline dehydrogenase (EC 1.5.99.8) (Proline oxidase) / Delta-1-pyrroline-5-carboxylate dehydrogenase (EC 1.5.1.12)  hypothetical protein |
| 3494 | AGHY01000646.1:1..3494 | Predicted sodium-dependent galactose transporter  diguanylate cyclase/phosphodiesterase (GGDEF |
| 3494 | AGHY01000252.1:1..3494 | Di-/tripeptide transporter  Di-/tripeptide transporter  Di-/tripeptide transporter  Tryptophan 22C3-dioxygenase (EC 1.13.11.11)  hypothetical protein |
| 3492 | AGHY01002116.1:1..3492 | Integrase regulator R  Single-stranded DNA-binding protein  hypothetical protein |
| 3486 | AGHY01000878.1:1..3486 | 3-oxoadipate CoA-transferase subunit A (EC 2.8.3.6)  3-oxoadipate CoA-transferase subunit B (EC 2.8.3.6)  hypothetical protein |
| 3474 | AGHY01001284.1:1..3474 | Topoisomerase IV subunit A (EC 5.99.1.-)  Topoisomerase IV subunit A (EC 5.99.1.-)  Topoisomerase IV subunit A (EC 5.99.1.-)  probable DNA-binding protein |
| 3472 | AGHY01000525.1:1..3472 | Cytochrome O ubiquinol oxidase subunit II (EC 1.10.3.-)  Cytochrome O ubiquinol oxidase subunit II (EC 1.10.3.-)  hypothetical protein  tRNA-Thr-CGT |
| 3460 | AGHY01000153.1:1..3460 | COG0779: clustered with transcription termination protein NusA  NADH-ubiquinone oxidoreductase chain N (EC 1.6.5.3)  Transcription termination protein NusA  tRNA-Met-CAT |
| 3439 | AGHY01001282.1:1..3439 | COG1272: Predicted membrane protein hemolysin III homolog  Membrane proteins related to metalloendopeptidases  Peptide chain release factor 3 |
| 3429 | AGHY01000038.1:1..3429 | FIG026997: Hypothetical protein  FIG034376: Hypothetical protein  FIG041301: Hypothetical protein  FIG049434: Periplasmic protein TonB2C links inner and outer membranes  hypothetical protein |
| 3421 | AGHY01000129.1:1..3421 | hypothetical protein  hypothetical protein |
| 3417 | AGHY01000903.1:1..3417 | Cytochrome c oxidase polypeptide I (EC 1.9.3.1)  Cytochrome c oxidase polypeptide III (EC 1.9.3.1)  Cytochrome oxidase biogenesis protein Cox11-CtaG2C copper delivery to Cox1 |
| 3415 | AGHY01000347.1:1..3415 | TonB-dependent receptor  TonB-dependent receptor  TonB-dependent receptor |
| 3410 | AGHY01000427.1:1..3410 | FIG01210269: hypothetical protein  Ferredoxin2C 2Fe-2S  sulfur deprivation response regulator |
| 3396 | AGHY01000557.1:1..3396 | Aerobic C4-dicarboxylate transporter for fumarate2C L-malate2C D-malate2C succunate2C aspartate  NADP-dependent malic enzyme (EC 1.1.1.40)  NADP-dependent malic enzyme (EC 1.1.1.40) |
| 3390 | AGHY01001142.1:1..3390 | B12 binding domain / kinase domain / Methylmalonyl-CoA mutase (EC 5.4.99.2)  B12 binding domain / kinase domain / Methylmalonyl-CoA mutase (EC 5.4.99.2)  B12 binding domain / kinase domain / Methylmalonyl-CoA mutase (EC 5.4.99.2) |
| 3386 | AGHY01000418.1:1..3386 | D-alanyl-D-alanine dipeptidase  FIG01210473: hypothetical protein  FIG01211609: hypothetical protein  hypothetical protein |
| 3386 | AGHY01000194.1:1..3386 | Acetylglutamate kinase (EC 2.7.2.8)  Acetylglutamate kinase (EC 2.7.2.8)  Acetylornithine deacetylase (EC 3.5.1.16)  N-acetylglutamate synthase (EC 2.3.1.1)  N-acetylglutamate synthase (EC 2.3.1.1) |
| 3370 | AGHY01001514.1:1..3370 | 2-ketoglutaric semialdehyde dehydrogenase (EC 1.2.1.26)  Aldehyde dehydrogenase B (EC 1.2.1.22)  Putative sugar ABC transport system2C periplasmic binding protein YtfQ precursor  Putative sugar ABC transport system2C periplasmic binding protein YtfQ precursor  SUGAR TRANSPORTER |
| 3370 | AGHY01000015.1:1..3370 | FIG000506: Predicted P-loop-containing kinase  HPr kinase/phosphorylase (EC 2.7.1.-) (EC 2.7.4.-)  PTS system nitrogen-specific IIA component2C PtsN  RNA polymerase sigma-54 factor RpoN  Ribosome hibernation protein YhbH  hypothetical protein |
| 3364 | AGHY01000676.1:1..3364 | Alpha-amylase (EC 3.2.1.1)  Trehalose synthase (EC 5.4.99.16) |
| 3358 | AGHY01000610.1:1..3358 | FIG01210152: hypothetical protein  hypothetical protein |
| 3358 | AGHY01000377.1:1..3358 | Catalase (EC 1.11.1.6)  hypothetical protein  hypothetical protein |
| 3352 | AGHY01000216.1:1..3352 | ATP-dependent Clp protease proteolytic subunit (EC 3.4.21.92)  Cell division trigger factor (EC 5.2.1.8) |
| 3347 | AGHY01000906.1:1..3347 | Membrane-associated zinc metalloprotease  Outer membrane protein assembly factor YaeT precursor |
| 3346 | AGHY01000248.1:1..3346 | Fumarate hydratase class I2C aerobic (EC 4.2.1.2)  Glutathione S-transferase (EC 2.5.1.18)  hypothetical protein  hypothetical protein |
| 3343 | AGHY01000470.1:1..3343 | FIG000906: Predicted Permease  FIG000906: Predicted Permease  FIG000988: Predicted permease  FIG01212138: hypothetical protein |
| 3318 | AGHY01000842.1:1..3318 | ATP-dependent protease La (EC 3.4.21.53) Type I  ATP-dependent protease La (EC 3.4.21.53) Type I  ATP-dependent protease La (EC 3.4.21.53) Type I  DNA-binding protein HU-alpha  tRNA-Val-TAC |
| 3313 | AGHY01001852.1:1..3313 | Aerotaxis sensor receptor protein  Arginine-tRNA-protein transferase (EC 2.3.2.8)  hypothetical protein |
| 3309 | AGHY01000584.1:1..3309 | FIG01111044: hypothetical protein  FIG01111044: hypothetical protein  Phosphate ABC transporter2C periplasmic phosphate-binding protein PstS (TC 3.A.1.7.1) |
| 3308 | AGHY01003061.1:1..3308 | Aminopeptidase N  FIG01149443: hypothetical protein  hypothetical protein  hypothetical protein |
| 3307 | AGHY01001280.1:1..3307 | Chemotaxis regulator - transmits chemoreceptor signals to flagelllar motor components CheY  Flagellar biosynthesis protein FlhF  Flagellar synthesis regulator FleN  RNA polymerase sigma factor for flagellar operon |
| 3304 | AGHY01000868.1:1..3304 | Potassium efflux system KefA protein / Small-conductance mechanosensitive channel  Ubiquinone biosynthesis monooxygenase UbiB |
| 3300 | AGHY01000374.1:1..3300 | FIG01209725: hypothetical protein  FIG01210483: hypothetical protein  Sensor protein PhoQ (EC 2.7.13.3) |
| 3281 | AGHY01000957.1:1..3281 | Succinyl-CoA ligase [ADP-forming] alpha chain (EC 6.2.1.5)  Succinyl-CoA ligase [ADP-forming] beta chain (EC 6.2.1.5)  hypothetical protein |
| 3278 | AGHY01000143.1:1..3278 | hypothetical protein |
| 3277 | AGHY01000131.1:1..3277 | Pectate lyase precursor (EC 4.2.2.2)  Pectate lyase precursor (EC 4.2.2.2)  probable aminopeptidase  probable aminopeptidase |
| 3274 | AGHY01000309.1:1..3274 | Acetyl-CoA hydrolase  Acetyl-CoA hydrolase  transcriptional regulator blaI family |
| 3258 | AGHY01000140.1:1..3258 | FIG01111142: hypothetical protein  Thiamin biosynthesis protein ThiC |
| 3249 | AGHY01000172.1:1..3249 | FIG01210372: hypothetical protein |
| 3248 | AGHY01000523.1:1..3248 | Methyl-accepting chemotaxis protein I (serine chemoreceptor protein)  Phosphoribosylformylglycinamidine synthase2C synthetase subunit (EC 6.3.5.3) / Phosphoribosylformylglycinamidine synthase2C glutamine amidotransferase subunit (EC 6.3.5.3)  hypothetical protein  hypothetical protein |
| 3240 | AGHY01000099.1:1..3240 | LSU ribosomal protein L15p (L27Ae)  LSU ribosomal protein L30p (L7e)  Preprotein translocase secY subunit (TC 3.A.5.1.1)  Preprotein translocase secY subunit (TC 3.A.5.1.1)  SSU ribosomal protein S5p (S2e) |
| 3239 | AGHY01000737.1:1..3239 | Flagellar hook-associated protein FlgK  hypothetical protein |
| 3232 | AGHY01000209.1:1..3232 | 3'-to-5' exoribonuclease RNase R  Gfa-like protein |
| 3231 | AGHY01001283.1:1..3231 | Aminomethyltransferase (glycine cleavage system T protein) (EC 2.1.2.10)  Glycine cleavage system H protein  Histone H1 |
| 3219 | AGHY01001044.1:1..3219 | hypothetical protein |
| 3190 | AGHY01000268.1:1..3190 | Alpha-12C2-mannosidase  Alpha-12C2-mannosidase  Alpha-12C2-mannosidase  Alpha-12C2-mannosidase |
| 3183 | AGHY01000944.1:1..3183 | Isocitrate dehydrogenase [NADP] (EC 1.1.1.42); Monomeric isocitrate dehydrogenase [NADP] (EC 1.1.1.42)  hypothetical protein |
| 3181 | AGHY01000340.1:1..3181 | glutamine synthetase family protein |
| 3178 | AGHY01000680.1:1..3178 | TonB-dependent receptor  TonB-dependent receptor |
| 3174 | AGHY01000642.1:1..3174 | N-Acetyl-D-glucosamine ABC transport system2C permease protein 2  Sugar ABC transporter2C periplasmic sugar-binding protein USSDB1B  Sugar ABC transporter2C sugar permease protein 1 USSDB1C |
| 3149 | AGHY01000097.1:1..3149 | FIG01210399: hypothetical protein  Positive regulator of CheA protein activity (CheW)  hypothetical protein  hypothetical protein |
| 3142 | AGHY01003060.1:1..3142 | Chromosome (plasmid) partitioning protein ParB / Stage 0 sporulation protein J  Dolichol-phosphate mannosyltransferase  FIG01210656: hypothetical protein  Mitomycin resistance protein  dTDP-glucose 42C6-dehydratase (EC 4.2.1.46) |
| 3138 | AGHY01000667.1:1..3138 | Amino acid permease  cationic amino acid transporter |
| 3117 | AGHY01000816.1:1..3117 | 4-hydroxybenzoate transporter  Quinate/shikimate dehydrogenase [Pyrroloquinoline-quinone] (EC 1.1.99.25)  hypothetical protein |
| 3113 | AGHY01000088.1:1..3113 | Bacterioferritin  FIG01211244: hypothetical protein  putative; ORF located using Glimmer/Genemark |
| 3106 | AGHY01000193.1:1..3106 | COG0845: Membrane-fusion protein  hypothetical protein  putative; ORF located using Glimmer/Genemark |
| 3105 | AGHY01000410.1:1..3105 | Transcriptional regulator2C AsnC family  hypothetical protein  hypothetical protein |
| 3101 | AGHY01000452.1:1..3101 | Type IV fimbrial biogenesis protein FimT  Type IV fimbrial biogenesis protein PilV  hypothetical protein  hypothetical protein  hypothetical protein |
| 3085 | AGHY01000444.1:1..3085 | Xaa-Pro aminopeptidase (EC 3.4.11.9)  Xaa-Pro dipeptidase PepQ (EC 3.4.13.9)  Xaa-Pro dipeptidase PepQ (EC 3.4.13.9)  hypothetical protein |
| 3081 | AGHY01000321.1:1..3081 | Adenosylhomocysteinase (EC 3.3.1.1)  hypothetical protein  methyltransferase |
| 3076 | AGHY01000942.1:1..3076 | FIG01210301: hypothetical protein  TPR domain protein2C putative component of TonB system  TPR domain protein2C putative component of TonB system  Zn-dependent protease with chaperone function PA4632 |
| 3066 | AGHY01002342.1:1..3066 | TonB-dependent receptor |
| 3056 | AGHY01001225.1:1..3056 | Anthranilate synthase2C amidotransferase component (EC 4.1.3.27) @ Para-aminobenzoate synthase2C amidotransferase component (EC 2.6.1.85) |
| 3049 | AGHY01000400.1:1..3049 | FIG01211910: hypothetical protein  hypothetical protein |
| 3042 | AGHY01000176.1:1..3042 | DNA-directed RNA polymerase alpha subunit (EC 2.7.7.6)  LSU ribosomal protein L17p  SSU ribosomal protein S11p (S14e)  SSU ribosomal protein S13p (S18e)  SSU ribosomal protein S4p (S9e) |
| 3021 | AGHY01000161.1:1..3021 | wall associated protein  wall associated protein |
| 3014 | AGHY01000075.1:1..3014 | FIG01211391: hypothetical protein  RNA polymerase sigma factor RpoH  Response regulator protein  Uracil-DNA glycosylase2C family 1 |
| 3002 | AGHY01000809.1:1..3002 | TonB-dependent receptor  prolyl oligopeptidase family protein  prolyl oligopeptidase family protein |
| 3000 | AGHY01000401.1:1..3000 | GumB protein  Xanthan biosynthesis chain length determinant protein GumC  hypothetical protein |
| 2996 | AGHY01000104.1:1..2996 | Acetyl-CoA synthetase (ADP-forming) alpha and beta chains2C putative  Acetyl-CoA synthetase (ADP-forming) alpha and beta chains2C putative  hypothetical protein  hypothetical protein  hypothetical protein |
| 2989 | AGHY01000714.1:1..2989 | TonB-dependent receptor |
| 2988 | AGHY01000813.1:1..2988 | Dephospho-CoA kinase (EC 2.7.1.24)  Leader peptidase (Prepilin peptidase) (EC 3.4.23.43) / N-methyltransferase (EC 2.1.1.-)  Type IV fimbrial assembly protein PilC |
| 2982 | AGHY01000224.1:1..2982 | OmpA-related protein  OmpA-related protein  Tryptophan halogenase |
| 2981 | AGHY01001216.1:1..2981 | Glycine cleavage system transcriptional antiactivator GcvR  Predicted ATPase related to phosphate starvation-inducible protein PhoH  Thiol peroxidase2C Bcp-type (EC 1.11.1.15) |
| 2971 | AGHY01000456.1:1..2971 | Homoserine O-acetyltransferase (EC 2.3.1.31)  extracellular protease( EC:3.4.21.- ) |
| 2968 | AGHY01000534.1:1..2968 | Phage integrase  hypothetical protein  tRNA-Met-CAT |
| 2966 | AGHY01001000.1:1..2966 | Catalase (EC 1.11.1.6) / Peroxidase (EC 1.11.1.7)  hypothetical protein  hypothetical protein |
| 2965 | AGHY01001214.1:1..2965 | TonB-dependent receptor  hypothetical protein |
| 2962 | AGHY01000889.1:1..2962 | Pyruvate dehydrogenase E1 component (EC 1.2.4.1)  Pyruvate dehydrogenase E1 component (EC 1.2.4.1) |
| 2955 | AGHY01000772.1:1..2955 | C-di-GMP phosphodiesterase A  Histidine kinase  Histidine kinase  hypothetical protein |
| 2950 | AGHY01000592.1:1..2950 | Radical SAM domain protein  conserved hypothetical protein  hypothetical protein |
| 2947 | AGHY01000187.1:1..2947 | Methionyl-tRNA synthetase (EC 6.1.1.10)  Methionyl-tRNA synthetase (EC 6.1.1.10)  Phosphoserine phosphatase (EC 3.1.3.3) |
| 2945 | AGHY01001277.1:1..2945 | Phosphate regulon sensor protein PhoR (SphS) (EC 2.7.13.3)  Phosphate regulon sensor protein PhoR (SphS) (EC 2.7.13.3)  Phosphate regulon transcriptional regulatory protein PhoB (SphR) |
| 2926 | AGHY01001057.1:1..2926 | TonB-dependent receptor  TonB-dependent receptor |
| 2919 | AGHY01001697.1:1..2919 | DNA gyrase subunit A (EC 5.99.1.3)  Methylthioribose-1-phosphate isomerase (EC 5.3.1.23) |
| 2916 | AGHY01000465.1:1..2916 | GTP-binding protein Era  Ribonuclease III (EC 3.1.26.3)  Signal peptidase I (EC 3.4.21.89) |
| 2913 | AGHY01000468.1:1..2913 | Lysyl-tRNA synthetase (class II) (EC 6.1.1.6)  Peptide chain release factor 2; programmed frameshift-containing |
| 2910 | AGHY01001234.1:1..2910 | FIG01211949: hypothetical protein  Glutathione S-transferase (EC 2.5.1.18)  Propionate--CoA ligase (EC 6.2.1.17)  hypothetical protein |
| 2910 | AGHY01000425.1:1..2910 | Formate dehydrogenase chain D (EC 1.2.1.2)  Putative formate dehydrogenase oxidoreductase protein  Putative formate dehydrogenase oxidoreductase protein  hypothetical protein |
| 2903 | AGHY01000618.1:1..2903 | 6-phosphofructokinase (EC 2.7.1.11)  FIG01210548: hypothetical protein  hypothetical protein |
| 2895 | AGHY01000629.1:1..2895 | Type IV pilus biogenesis protein PilM |
| 2893 | AGHY01000664.1:1..2893 | FIG01211837: hypothetical protein  Phosphoethanolamine transferase specific for the outer Kdo residue of lipopolysaccharide |
| 2891 | AGHY01000304.1:1..2891 | Thymidine kinase (EC 2.7.1.21)  Thymidine kinase (EC 2.7.1.21) |
| 2886 | AGHY01001167.1:1..2886 | Hypothetical protein USSDB1A  OmpA-related protein |
| 2885 | AGHY01000462.1:1..2885 | Asparaginyl-tRNA synthetase (EC 6.1.1.22)  Asparaginyl-tRNA synthetase (EC 6.1.1.22)  Iron binding protein SufA for iron-sulfur cluster assembly  SSU ribosomal protein S18p |
| 2869 | AGHY01000884.1:1..2869 | Membrane proteins related to metalloendopeptidases  Membrane proteins related to metalloendopeptidases  Tyrosyl-tRNA synthetase (EC 6.1.1.1)  hypothetical protein |
| 2864 | AGHY01000025.1:1..2864 | TonB-dependent receptor  TonB-dependent receptor |
| 2858 | AGHY01000254.1:1..2858 | Ribosomal large subunit pseudouridine synthase C (EC 4.2.1.70)  Ribosomal large subunit pseudouridine synthase C (EC 4.2.1.70) |
| 2852 | AGHY01001017.1:1..2852 | Basic proline-rich protein precursor  Transcriptional regulator2C AraC family  hypothetical protein |
| 2844 | AGHY01000106.1:1..2844 | FIG01210031: hypothetical protein  hypothetical protein |
| 2842 | AGHY01001278.1:1..2842 | Isocitrate dehydrogenase phosphatase (EC 2.7.11.5)/kinase (EC 3.1.3.-) |
| 2837 | AGHY01000655.1:1..2837 | FIG01211213: hypothetical protein  FIG032621: Hydrolase2C alpha/beta hydrolase fold family  Transcriptional regulator2C Cro/CI family |
| 2829 | AGHY01000308.1:1..2829 | TonB-dependent receptor  TonB-dependent receptor  hypothetical protein |
| 2827 | AGHY01000898.1:1..2827 | Acetate permease ActP (cation/acetate symporter)  FIG01111534: hypothetical protein  Putative membrane protein2C clustering with ActP |
| 2826 | AGHY01000256.1:1..2826 | FIG01211451: hypothetical protein  Methionine ABC transporter substrate-binding protein  outer membrane protein |
| 2819 | AGHY01000039.1:1..2819 | Cyclic AMP receptor protein  Quaternary ammonium compound-resistance protein sugE  S-adenosylmethionine decarboxylase proenzyme (EC 4.1.1.50)2C prokaryotic class 1A  S-adenosylmethionine decarboxylase proenzyme (EC 4.1.1.50)2C prokaryotic class 1A |
| 2818 | AGHY01001018.1:1..2818 | TonB-dependent receptor  TonB-dependent receptor |
| 2815 | AGHY01000950.1:1..2815 | FIG01210028: hypothetical protein  GCN5-related N-acetyltransferase  N-acetylglucosamine-1-phosphate uridyltransferase (EC 2.7.7.23) / Glucosamine-1-phosphate N-acetyltransferase (EC 2.3.1.157)  N-acetylglucosamine-1-phosphate uridyltransferase (EC 2.7.7.23) / Glucosamine-1-phosphate N-acetyltransferase (EC 2.3.1.157) |
| 2813 | AGHY01000548.1:1..2813 | Acriflavin resistance protein  Acriflavin resistance protein |
| 2809 | AGHY01000527.1:1..2809 | Protein-L-isoaspartate O-methyltransferase (EC 2.1.1.77)  Transcriptional regulator2C TetR family  Type I secretion outer membrane protein2C TolC precursor  hypothetical protein |
| 2801 | AGHY01000536.1:1..2801 | Cytochrome oxidase biogenesis protein Sco1/SenC/PrrC2C putative copper metallochaperone  Phosphatidylserine decarboxylase (EC 4.1.1.65)  Protein-N(5)-glutamine methyltransferase PrmB2C methylates LSU ribosomal protein L3p  hypothetical protein |
| 2800 | AGHY01001079.1:1..2800 | Transcriptional regulator lacI family  hypothetical protein  hypothetical protein |
| 2793 | AGHY01001064.1:1..2793 | Alpha/beta hydrolase fold (EC 3.8.1.5)  hypothetical protein  hypothetical protein |
| 2790 | AGHY01000661.1:1..2790 | Citrate synthase (si) (EC 2.3.3.1)  Citrate synthase (si) (EC 2.3.3.1)  FIG01209925: hypothetical protein  Inosine-uridine preferring nucleoside hydrolase (EC 3.2.2.1)  LSU ribosomal protein L31p |
| 2785 | AGHY01000929.1:1..2785 | Sugar kinase  Sugar kinase |
| 2783 | AGHY01000108.1:1..2783 | (Acyl-carrier protein) phosphodiesterase( EC:3.1.4.14 )  Phosphoglycerate mutase  Phospholipase C  hypothetical protein |
| 2774 | AGHY01000388.1:1..2774 | FIG01210644: hypothetical protein  Ferric siderophore transport system2C periplasmic binding protein TonB  Ferric siderophore transport system2C periplasmic binding protein TonB  Molybdenum transport ATP-binding protein ModC (TC 3.A.1.8.1) |
| 2773 | AGHY01001106.1:1..2773 | Aspartyl-tRNA synthetase (EC 6.1.1.12)  hypothetical protein |
| 2770 | AGHY01000177.1:1..2770 | ATP-dependent DNA helicase UvrD/PcrA  ATP-dependent DNA helicase UvrD/PcrA  ATP-dependent DNA helicase UvrD/PcrA  hypothetical protein |
| 2753 | AGHY01001577.1:1..2753 | putative exported protein |
| 2752 | AGHY01001467.1:1..2752 | FIG01210778: hypothetical protein  Fatty acid desaturase (EC 1.14.19.1); Delta-9 fatty acid desaturase (EC 1.14.19.1) |
| 2747 | AGHY01000325.1:1..2747 | Chemotaxis protein methyltransferase CheR (EC 2.1.1.80) |
| 2740 | AGHY01000415.1:1..2740 | FIG01211153: hypothetical protein  hypothetical protein |
| 2738 | AGHY01001179.1:1..2738 | COG28792C Hypothetical small protein yjiX  Carbon starvation protein A  hypothetical protein |
| 2731 | AGHY01000520.1:1..2731 |  |
| 2729 | AGHY01000395.1:1..2729 | Chaperone protein HtpG  Ureidoglycolate/malate/sulfolactate dehydrogenase family (EC 1.1.1.-) |
| 2727 | AGHY01001363.1:1..2727 | ThiJ/PfpI family protein  Transaldolase (EC 2.2.1.2)  hypothetical protein |
| 2723 | AGHY01001368.1:1..2723 | FIG01210717: hypothetical protein  FIG01211456: hypothetical protein |
| 2720 | AGHY01000234.1:1..2720 | LSU m5C1962 methyltransferase RlmI  export protein |
| 2707 | AGHY01000413.1:1..2707 | 3-oxoacyl-[acyl-carrier protein] reductase (EC 1.1.1.100)  3-oxoacyl-[acyl-carrier-protein] synthase2C KASII (EC 2.3.1.41)  Acyl carrier protein |
| 2702 | AGHY01000062.1:1..2702 | FIG01112087: hypothetical protein  Glutathione S-transferase (EC 2.5.1.18)  dipeptidyl peptidase IV |
| 2701 | AGHY01001342.1:1..2701 | Phosphate transport system permease protein PstA (TC 3.A.1.7.1)  Phosphate transport system permease protein PstA (TC 3.A.1.7.1)  Phosphate transport system permease protein PstC (TC 3.A.1.7.1) |
| 2693 | AGHY01001765.1:1..2693 | ABC transporter permease protein  ABC transporter2C substrate binding protein  ABC-transport protein2C ATP-binding protein |
| 2693 | AGHY01001135.1:1..2693 | diguanylate cyclase with GAF sensor  hypothetical protein  hypothetical protein |
| 2672 | AGHY01001412.1:1..2672 | Methylglyoxal synthase (EC 4.2.3.3)  cellulase  hypothetical protein |
| 2666 | AGHY01000489.1:1..2666 |  |
| 2665 | AGHY01000526.1:1..2665 | HflC protein  HflK protein |
| 2662 | AGHY01000409.1:1..2662 | Oar protein  putative ORF-3 protein |
| 2662 | AGHY01000001.1:1..2662 | NAD-specific glutamate dehydrogenase (EC 1.4.1.2)2C large form  NAD-specific glutamate dehydrogenase (EC 1.4.1.2)2C large form  hypothetical protein |
| 2652 | AGHY01001170.1:1..2652 | hypothetical protein  hypothetical protein |
| 2648 | AGHY01002265.1:1..2648 | D-2-hydroxyglutarate dehydrogenase |
| 2644 | AGHY01000710.1:1..2644 |  |
| 2641 | AGHY01000921.1:1..2641 | Predicted Zn-dependent peptidases |
| 2632 | AGHY01000475.1:1..2632 | Cell division protein FtsA |
| 2627 | AGHY01002029.1:1..2627 | 5-nucleotidase SurE (EC 3.1.3.5)  Protein-L-isoaspartate O-methyltransferase (EC 2.1.1.77)  Protein-L-isoaspartate O-methyltransferase (EC 2.1.1.77) |
| 2625 | AGHY01000773.1:1..2625 | FIG01209965: hypothetical protein  hypothetical protein  hypothetical protein  two-component system regulatory protein |
| 2624 | AGHY01001578.1:1..2624 | Glycerophosphoryl diester phosphodiesterase (EC 3.1.4.46)  hypothetical protein  hypothetical protein  hypothetical protein  hypothetical protein |
| 2624 | AGHY01000504.1:1..2624 | Polyphosphate kinase (EC 2.7.4.1) |
| 2614 | AGHY01001860.1:1..2614 | Holliday junction DNA helicase RuvA  Kup system potassium uptake protein |
| 2600 | AGHY01000255.1:1..2600 | Cytochrome d ubiquinol oxidase subunit II (EC 1.10.3.-)  Cytochrome d ubiquinol oxidase subunit II (EC 1.10.3.-)  HlyD family secretion protein  hypothetical protein  hypothetical protein  tRNA-Pro-CGG |
| 2599 | AGHY01001498.1:1..2599 | Cytochrome P450( EC:1.6.2.42CEC:1.14.14.1 )  Cytochrome P450( EC:1.6.2.42CEC:1.14.14.1 ) |
| 2597 | AGHY01000605.1:1..2597 | Phosphomannomutase (EC 5.4.2.8) |
| 2596 | AGHY01000134.1:1..2596 | TonB-dependent receptor  TonB-dependent receptor |
| 2590 | AGHY01000204.1:1..2590 | Arginase (EC 3.5.3.1)  Protein yjbJ  entericidin A  hypothetical protein |
| 2589 | AGHY01000690.1:1..2589 | hypothetical protein  hypothetical protein |
| 2588 | AGHY01000217.1:1..2588 | YaeQ protein  hypothetical protein  major cold shock protein |
| 2586 | AGHY01001181.1:1..2586 | Methylmalonate-semialdehyde dehydrogenase (EC 1.2.1.27)  hypothetical protein  hypothetical protein |
| 2585 | AGHY01001875.1:1..2585 | Glycerol kinase (EC 2.7.1.30)  Glycerol uptake facilitator protein  Glycerol uptake facilitator protein |
| 2585 | AGHY01000443.1:1..2585 | TonB-dependent receptor  TonB-dependent receptor |
| 2579 | AGHY01001375.1:1..2579 | FIG01211818: hypothetical protein  Sensor protein PhoQ (EC 2.7.13.3)  Two-component system regulatory protein |
| 2571 | AGHY01000362.1:1..2571 | Tetratricopeptide TPR\_2  TonB-dependent receptor |
| 2567 | AGHY01000553.1:1..2567 | Sugar transporter  sugar transporter  transcriptional regulator |
| 2565 | AGHY01001655.1:1..2565 | Protein-export membrane protein SecD (TC 3.A.5.1.1)  Protein-export membrane protein SecF (TC 3.A.5.1.1) |
| 2561 | AGHY01001127.1:1..2561 | ABC transporter ATP-binding protein  ABC transporter ATP-binding protein  ABC transporter permease |
| 2556 | AGHY01000510.1:1..2556 | Anthranilate phosphoribosyltransferase (EC 2.4.2.18)  Indole-3-glycerol phosphate synthase (EC 4.1.1.48)  Nitrilotriacetate monooxygenase component B (EC 1.14.13.-) |
| 2545 | AGHY01000429.1:1..2545 | FIG01210576: hypothetical protein |
| 2545 | AGHY01000282.1:1..2545 | ATPase provides energy for both assembly of type IV secretion complex and secretion of T-DNA complex (VirB11)  ATPase provides energy for both assembly of type IV secretion complex and secretion of T-DNA complex (VirB11)  Inner membrane protein forms channel for type IV secretion of T-DNA complex (VirB10) |
| 2544 | AGHY01000782.1:1..2544 | Polysaccharide deacetylase  RNA:NAD 2'-phosphotransferase  hypothetical protein |
| 2543 | AGHY01001439.1:1..2543 | Aldehyde dehydrogenase B (EC 1.2.1.22)  Aldehyde dehydrogenase B (EC 1.2.1.22)  tRNA-Arg-CCT |
| 2536 | AGHY01001431.1:1..2536 | Transport protein  Transport protein  transport protein |
| 2536 | AGHY01000870.1:1..2536 | Putative OMR family iron-siderophore receptor precursor  hypothetical protein  hypothetical protein |
| 2536 | AGHY01000531.1:1..2536 | Xylanase  Xylanase |
| 2531 | AGHY01000079.1:1..2531 | Manganese transport protein MntH  Manganese transport protein MntH  hypothetical protein  hypothetical protein |
| 2529 | AGHY01001177.1:1..2529 | Branched-chain alpha-keto acid dehydrogenase2C E1 component2C alpha subunit (EC 1.2.4.4)  Branched-chain alpha-keto acid dehydrogenase2C E1 component2C beta subunit (EC 1.2.4.4)  hypothetical protein |
| 2520 | AGHY01000218.1:1..2520 | L-Proline/Glycine betaine transporter ProP  L-Proline/Glycine betaine transporter ProP  L-Proline/Glycine betaine transporter ProP  L-Proline/Glycine betaine transporter ProP |
| 2515 | AGHY01000385.1:1..2515 | Probable transposase protein |
| 2512 | AGHY01000335.1:1..2512 | Dicarboxylate carrier MatC-like  Dicarboxylate carrier protein  Dicarboxylate carrier protein  Predicted regulator PutR for proline utilization2C GntR family  dicarboxylate carrier protein |
| 2509 | AGHY01001219.1:1..2509 | Pyruvate oxidase [ubiquinone2C cytochrome] (EC 1.2.2.2)  conserved hypothetical protein  hypothetical protein |
| 2506 | AGHY01001206.1:1..2506 | Phosphate-specific outer membrane porin OprP ; Pyrophosphate-specific outer membrane porin OprO  Uncharacterized transporter2C similarity to citrate transporter |
| 2501 | AGHY01000295.1:1..2501 | Sulfur carrier protein ThiS  Thiazole biosynthesis protein ThiG  hypothetical protein |
| 2498 | AGHY01000174.1:1..2498 | DNA polymerase III alpha subunit (EC 2.7.7.7)  LigA |
| 2488 | AGHY01002083.1:1..2488 | DUF378 domain-containing protein  Gluconokinase (EC 2.7.1.12)  cell division control protein CDC48 homolog  hypothetical protein  hypothetical protein |
| 2486 | AGHY01000779.1:1..2486 | FIG01211822: hypothetical protein  Hypothetical membrane protein2C possible involvement in cytochrome functioning/assembly |
| 2483 | AGHY01001145.1:1..2483 | RND efflux system2C inner membrane transporter CmeB  RND efflux system2C membrane fusion protein CmeA  RND efflux system2C membrane fusion protein CmeA |
| 2481 | AGHY01001443.1:1..2481 | 4-amino-4-deoxy-L-arabinose transferase and related glycosyltransferases of PMT family  FIG01214694: hypothetical protein  hypothetical protein |
| 2474 | AGHY01000594.1:1..2474 | CDP-diacylglycerol--serine O-phosphatidyltransferase (EC 2.7.8.8)  FIG01213248: hypothetical protein |
| 2469 | AGHY01000485.1:1..2469 | Putative large exoprotein involved in heme utilization or adhesion of ShlA/HecA/FhaA family  filamentous haemagglutinin |
| 2463 | AGHY01000109.1:1..2463 | Flagellar biosynthesis protein FliL  Flagellar motor switch protein FliM  Flagellar motor switch protein FliN |
| 2462 | AGHY01000788.1:1..2462 | 3-oxoacyl-[acyl-carrier protein] reductase (EC 1.1.1.100)  3-oxoacyl-[acyl-carrier protein] reductase (EC 1.1.1.100) |
| 2460 | AGHY01001209.1:1..2460 | L-fuconate dehydratase (EC 4.2.1.68)  hypothetical protein |
| 2456 | AGHY01001015.1:1..2456 | TonB-dependent receptor |
| 2456 | AGHY01000549.1:1..2456 | Ferric vulnibactin receptor VuuA |
| 2453 | AGHY01001562.1:1..2453 | Outer membrane receptor for ferric coprogen and ferric-rhodotorulic acid  Outer membrane receptor for ferric coprogen and ferric-rhodotorulic acid  TonB-dependent siderophore receptor |
| 2453 | AGHY01000287.1:1..2453 | Glyoxalase family protein  Transcriptional regulator2C TetR family  hypothetical protein  hypothetical protein |
| 2449 | AGHY01000658.1:1..2449 | Fructose-12C6-bisphosphatase2C type I (EC 3.1.3.11)  putative MarR family transcriptional regulator |
| 2449 | AGHY01000372.1:1..2449 | FIG01211006: hypothetical protein  Ferric uptake regulation protein FUR  Outer membrane lipoprotein SmpA2C a component of the essential YaeT outer-membrane protein assembly complex  hypothetical protein  hypothetical protein  hypothetical protein  putative dehydrogenase |
| 2446 | AGHY01000121.1:1..2446 | Uncharacterized ABC transporter2C auxiliary component YrbC  Uncharacterized ABC transporter2C periplasmic component YrbD  Uncharacterized ABC transporter2C permease component YrbE |
| 2443 | AGHY01000732.1:1..2443 | TonB-dependent receptor |
| 2437 | AGHY01001248.1:1..2437 | Leucyl-tRNA synthetase (EC 6.1.1.4)  Leucyl-tRNA synthetase (EC 6.1.1.4) |
| 2433 | AGHY01002052.1:1..2433 | TonB-dependent receptor  TonB-dependent receptor  TonB-dependent receptor  TonB-dependent receptor |
| 2430 | AGHY01000236.1:1..2430 | FIG01209836: hypothetical protein  hypothetical protein |
| 2424 | AGHY01000981.1:1..2424 | Probable FERULIC acid hydratase protein |
| 2419 | AGHY01000843.1:1..2419 | ADP-ribose 1"-phosphate phophatase related protein  L-sorbosone dehydrogenase  hypothetical protein |
| 2417 | AGHY01000837.1:1..2417 | LigA  probable two-component response regulator |
| 2411 | AGHY01001344.1:1..2411 | ABC-type multidrug transport system2C ATPase component |
| 2405 | AGHY01001378.1:1..2405 | Na /H antiporter  Na /H antiporter  hypothetical protein |
| 2401 | AGHY01000352.1:1..2401 | Guanosine polyphosphate pyrophosphohydrolases/synthetases  hypothetical protein |
| 2389 | AGHY01001469.1:1..2389 | Transcriptional regulator2C AraC family  Transcriptional regulator2C LysR family  hypothetical protein  tRNA-Ser-TGA |
| 2387 | AGHY01000480.1:1..2387 | Transcriptional regulator2C TetR family  hypothetical protein  hypothetical protein  hypothetical protein  hypothetical protein  putative cytochrome b561  putative cytochrome b561 |
| 2384 | AGHY01000668.1:1..2384 | FIG023873: Plasmid related protein  hypothetical protein |
| 2380 | AGHY01000137.1:1..2380 | TonB-dependent receptor |
| 2375 | AGHY01001385.1:1..2375 | Putative hemolysin  Ser/Thr protein phosphatase family protein2C UDP-22C3-diacylglucosamine hydrolase (EC 3.6.1.-) homolog  hypothetical protein |
| 2375 | AGHY01000073.1:1..2375 | Ribonucleotide reductase of class Ia (aerobic)2C alpha subunit (EC 1.17.4.1)  Ribonucleotide reductase of class Ia (aerobic)2C beta subunit (EC 1.17.4.1) |
| 2370 | AGHY01000414.1:1..2370 | Hemolysin |
| 2369 | AGHY01000089.1:1..2369 | Periplasmic aromatic aldehyde oxidoreductase2C molybdenum binding subunit YagR @ 4-hydroxybenzoyl-CoA reductase2C alpha subunit (EC 1.3.99.20) |
| 2368 | AGHY01000463.1:1..2368 | Phosphoribosylglycinamide formyltransferase 2 (EC 2.1.2.-)  ring hydroxylating dioxygenase alpha-subunit |
| 2363 | AGHY01003059.1:1..2363 | ABC-type multidrug transport system2C ATPase component  ABC-type multidrug transport system2C permease component  FIG01210488: hypothetical protein  FIG01211407: hypothetical protein |
| 2363 | AGHY01001478.1:1..2363 | LSU ribosomal protein L25p  Peptidyl-tRNA hydrolase (EC 3.1.1.29) |
| 2363 | AGHY01000575.1:1..2363 | FIG01209811: hypothetical protein  Riboflavin synthase alpha chain (EC 2.5.1.9)  hypothetical protein |
| 2361 | AGHY01000961.1:1..2361 | LSU m3Psi1915 methyltransferase RlmH  TonB-dependent receptor |
| 2361 | AGHY01000179.1:1..2361 | Translation elongation factor Ts |
| 2357 | AGHY01000198.1:1..2357 | Ribonucleotide reductase of class II (coenzyme B12-dependent)2C alpha subunit (EC 1.17.4.1)  Ribonucleotide reductase of class II (coenzyme B12-dependent)2C alpha subunit (EC 1.17.4.1)  hypothetical protein |
| 2351 | AGHY01000422.1:1..2351 | Protease II (EC 3.4.21.83)  Protease II (EC 3.4.21.83) |
| 2346 | AGHY01001401.1:1..2346 | Type IV pilus biogenesis protein PilQ |
| 2342 | AGHY01000058.1:1..2342 | hypothetical protein |
| 2340 | AGHY01000215.1:1..2340 | VirB6 protein  hypothetical protein |
| 2337 | AGHY01000805.1:1..2337 | TonB-dependent hemin 2C ferrichrome receptor  hypothetical protein |
| 2331 | AGHY01000078.1:1..2331 | FIG01211880: hypothetical protein  hypothetical protein |
| 2329 | AGHY01001224.1:1..2329 | Phospholipase D-like protein  putative; ORF located using Glimmer/Genemark |
| 2327 | AGHY01000488.1:1..2327 | LigA  Predicted maltose transporter MalT |
| 2325 | AGHY01000125.1:1..2325 | Glutathione-dependent formaldehyde-activating enzyme (EC 4.4.1.22)  S-(hydroxymethyl)glutathione dehydrogenase (EC 1.1.1.284)  S-(hydroxymethyl)glutathione dehydrogenase (EC 1.1.1.284) |
| 2323 | AGHY01000603.1:1..2323 | AmpG permease  Exodeoxyribonuclease III (EC 3.1.11.2) |
| 2320 | AGHY01001004.1:1..2320 | Manganese transport protein MntH  hypothetical protein  tRNA-Cys-GCA |
| 2313 | AGHY01001702.1:1..2313 | Chromosome partition protein smc  LSU ribosomal protein L9p  hypothetical protein |
| 2309 | AGHY01001771.1:1..2309 | Zinc-regulated outer membrane receptor  colicin I receptor |
| 2306 | AGHY01001097.1:1..2306 | LSU ribosomal protein L10p (P0)  LSU ribosomal protein L1p (L10Ae)  LSU ribosomal protein L7/L12 (P1/P2) |
| 2302 | AGHY01000496.1:1..2302 | FIG01212272: hypothetical protein  hypothetical protein  hypothetical protein  hypothetical protein  hypothetical protein |
| 2298 | AGHY01001700.1:1..2298 | FIG00509706: hypothetical protein |
| 2292 | AGHY01000744.1:1..2292 | FIG01211029: hypothetical protein  Probable component of the lipoprotein assembly complex (forms a complex with YaeT2C YfgL2C and NlpB) |
| 2278 | AGHY01000740.1:1..2278 | Putative permease  Putative regulatory protein |
| 2277 | AGHY01000628.1:1..2277 | TonB-dependent receptor |
| 2270 | AGHY01002243.1:1..2270 | L-sorbosone dehydrogenase  L-sorbosone dehydrogenase  Sensor histidine kinase  hypothetical protein |
| 2269 | AGHY01000142.1:1..2269 | DNA polymerase III alpha subunit (EC 2.7.7.7)  DNA polymerase III alpha subunit (EC 2.7.7.7) |
| 2267 | AGHY01000110.1:1..2267 | hypothetical protein  hypothetical protein  hypothetical protein  hypothetical protein |
| 2261 | AGHY01001637.1:1..2261 | histidine kinase/response regulator hybrid protein  hypothetical protein  tRNA-Leu-CAA |
| 2261 | AGHY01001415.1:1..2261 | hypothetical protein |
| 2259 | AGHY01000059.1:1..2259 | Acetyl-coenzyme A carboxyl transferase alpha chain (EC 6.4.1.2)  DNA polymerase III alpha subunit (EC 2.7.7.7)  hypothetical protein |
| 2257 | AGHY01000774.1:1..2257 | hypothetical protein  hypothetical protein |
| 2254 | AGHY01000455.1:1..2254 | Pectate lyase precursor (EC 4.2.2.2)  Ribosomal-protein-S18p-alanine acetyltransferase (EC 2.3.1.-) |
| 2249 | AGHY01001047.1:1..2249 | N-acetylglucosamine-regulated TonB-dependent outer membrane receptor  N-acetylglucosamine-regulated TonB-dependent outer membrane receptor  N-acetylglucosamine-regulated TonB-dependent outer membrane receptor |
| 2247 | AGHY01000533.1:1..2247 | response regulator |
| 2246 | AGHY01001148.1:1..2246 | Methionine aminotransferase2C PLP-dependent  Possible hydrolase  hypothetical protein |
| 2239 | AGHY01001666.1:1..2239 | Putative oxidoreductase  Replicative DNA helicase (EC 3.6.1.-)  hypothetical protein  hypothetical protein |
| 2231 | AGHY01001422.1:1..2231 | Esterase/lipase  Staphylolytic protease preproenzyme LasA  hypothetical protein |
| 2227 | AGHY01000188.1:1..2227 | FIG01210050: hypothetical protein  Glutaminyl-tRNA synthetase (EC 6.1.1.18) |
| 2226 | AGHY01000359.1:1..2226 | twitching motility protein PilG  twitching motility protein PilH  type IV pili signal transduction protein PilI |
| 2220 | AGHY01001549.1:1..2220 | hypothetical protein  hypothetical protein  hypothetical protein |
| 2220 | AGHY01000787.1:1..2220 | LigA |
| 2219 | AGHY01001602.1:1..2219 | FIG01212910: hypothetical protein  FIG01213330: hypothetical protein  Gluconolactonase |
| 2217 | AGHY01000560.1:1..2217 | hypothetical protein  hypothetical protein |
| 2217 | AGHY01000351.1:1..2217 | DNA primase (EC 2.7.7.-)  hypothetical protein |
| 2205 | AGHY01000518.1:1..2205 | Nicotinate phosphoribosyltransferase (EC 2.4.2.11)  hypothetical protein |
| 2202 | AGHY01000542.1:1..2202 | Multicopper oxidase  Multicopper oxidase  hypothetical protein  hypothetical protein |
| 2200 | AGHY01000189.1:1..2200 | hypothetical protein  hypothetical protein |
| 2199 | AGHY01000044.1:1..2199 | GMP synthase [glutamine-hydrolyzing] (EC 6.3.5.2)  Hypothetical transmembrane protein coupled to NADH-ubiquinone oxidoreductase chain 5 homolog |
| 2190 | AGHY01000144.1:1..2190 | Biosynthetic arginine decarboxylase (EC 4.1.1.19) |
| 2186 | AGHY01000581.1:1..2186 | Transketolase (EC 2.2.1.1) |
| 2186 | AGHY01000493.1:1..2186 | Gamma-glutamyltranspeptidase (EC 2.3.2.2) |
| 2185 | AGHY01000276.1:1..2185 | NAD-dependent protein deacetylase of SIR2 family |
| 2184 | AGHY01001430.1:1..2184 | Beta-galactosidase (EC 3.2.1.23) |
| 2178 | AGHY01001982.1:1..2178 | Beta-galactosidase (EC 3.2.1.23)  Beta-galactosidase (EC 3.2.1.23) |
| 2172 | AGHY01001732.1:1..2172 | Signal transduction histidine kinase CheA (EC 2.7.3.-)  methyl-accepting chemotaxis protein |
| 2171 | AGHY01000672.1:1..2171 | hypothetical protein  hypothetical protein |
| 2169 | AGHY01000991.1:1..2169 | DNA-binding response regulator  periplasmic sensor signal transduction histidine kinase |
| 2167 | AGHY01001195.1:1..2167 | potassium efflux system protein  putative Glutathione-regulated potassium-efflux system protein KefB |
| 2167 | AGHY01000303.1:1..2167 |  |
| 2161 | AGHY01001871.1:1..2161 | Oligopeptide transporter  Oligopeptide transporter  hypothetical protein |
| 2161 | AGHY01001341.1:1..2161 | Membrane fusion component of tripartite multidrug resistance system |
| 2161 | AGHY01001259.1:1..2161 | DNA polymerase I (EC 2.7.7.7)  Nitroreductase  Nitroreductase  hypothetical protein |
| 2157 | AGHY01000412.1:1..2157 | PDZ domain family protein  PDZ domain family protein |
| 2156 | AGHY01000160.1:1..2156 | Dipeptidyl peptidase IV |
| 2151 | AGHY01001333.1:1..2151 | 22C3-dihydroxy-22C3-dihydro-phenylpropionate dehydrogenase (EC 1.3.1.-)  hypothetical protein |
| 2133 | AGHY01001477.1:1..2133 | Alkylated DNA repair protein AlkB  Electron transfer flavoprotein-ubiquinone oxidoreductase (EC 1.5.5.1) |
| 2132 | AGHY01000692.1:1..2132 | FIG01211502: hypothetical protein  FIG01211598: hypothetical protein  Oligopeptide transporter |
| 2127 | AGHY01002011.1:1..2127 | Hemolysin activator protein precursor  filamentous haemagglutinin |
| 2122 | AGHY01000438.1:1..2122 | Twitching motility protein PilT  Twitching motility protein PilT  hypothetical protein |
| 2119 | AGHY01001744.1:1..2119 | General secretion pathway protein E / Type II secretion cytoplasmic ATP binding protein (PulE2C ATPase)  General secretion pathway protein F |
| 2113 | AGHY01001297.1:1..2113 | FIG01111726: hypothetical protein  Uncharacterized protein conserved in bacteria |
| 2111 | AGHY01000612.1:1..2111 | POSSIBLE LINOLEOYL-CoA DESATURASE (DELTA(6)-DESATURASE)  hypothetical protein  protein of unknown function DUF1452 |
| 2111 | AGHY01000200.1:1..2111 | Integral membrane protein  TonB-dependent receptor  TonB-dependent receptor  hypothetical protein |
| 2109 | AGHY01002979.1:1..2109 | TPR repeat-containing protein  hypothetical protein  hypothetical protein  hypothetical protein |
| 2103 | AGHY01001242.1:1..2103 | hypothetical protein |
| 2101 | AGHY01000914.1:1..2101 | Permeases of the major facilitator superfamily  hypothetical protein |
| 2101 | AGHY01000587.1:1..2101 | Xanthan biosynthesis oligosaccharidyl-lipid flippase GumJ |
| 2100 | AGHY01000850.1:1..2100 | Type IV secretory pathway2C VirD4 components  Type IV secretory pathway2C VirD4 components |
| 2099 | AGHY01001635.1:1..2099 | amino acid transporter  amino acid transporter |
| 2099 | AGHY01000392.1:1..2099 | Lipoprotein releasing system ATP-binding protein LolD  Lipoprotein releasing system transmembrane protein LolC |
| 2097 | AGHY01000712.1:1..2097 | FIG01210064: hypothetical protein |
| 2091 | AGHY01001806.1:1..2091 | hemolysin activator protein  hypothetical protein |
| 2090 | AGHY01000766.1:1..2090 | FIG01211374: hypothetical protein  acyl-CoA dehydrogenase  hypothetical protein |
| 2087 | AGHY01000037.1:1..2087 | FIG01210126: hypothetical protein |
| 2085 | AGHY01000262.1:1..2085 | LigA |
| 2083 | AGHY01000882.1:1..2083 | Valyl-tRNA synthetase (EC 6.1.1.9) |
| 2081 | AGHY01001317.1:1..2081 | Alcohol dehydrogenase (EC 1.1.1.1)  Alcohol dehydrogenase (EC 1.1.1.1)  Transcriptional regulator2C HxlR family |
| 2074 | AGHY01001519.1:1..2074 | Long-chain-fatty-acid--CoA ligase (EC 6.2.1.3)  Long-chain-fatty-acid--CoA ligase (EC 6.2.1.3) |
| 2074 | AGHY01000219.1:1..2074 | accessory protein  hypothetical protein  phospholipase A1 |
| 2071 | AGHY01000439.1:1..2071 |  |
| 2070 | AGHY01001390.1:1..2070 | Probable signal peptide protein  Uncharacterized glutathione S-transferase-like protein  hypothetical protein |
| 2069 | AGHY01001795.1:1..2069 | FIG01210913: hypothetical protein |
| 2065 | AGHY01000783.1:1..2065 | BatD  Proton/glutamate symport protein @ Sodium/glutamate symport protein  hypothetical protein |
| 2064 | AGHY01000583.1:1..2064 | Glutathione-regulated potassium-efflux system protein KefB |
| 2060 | AGHY01001129.1:1..2060 | FIG01210434: hypothetical protein  hypothetical protein  hypothetical protein  hypothetical protein |
| 2051 | AGHY01001008.1:1..2051 | FIG000859: hypothetical protein  hypothetical protein |
| 2050 | AGHY01000754.1:1..2050 | PilL protein  hypothetical protein |
| 2037 | AGHY01001073.1:1..2037 | ABC transporter ATP-binding protein  ABC transporter ATP-binding protein |
| 2036 | AGHY01001013.1:1..2036 |  |
| 2035 | AGHY01000083.1:1..2035 | Aldehyde dehydrogenase (EC 1.2.1.3) |
| 2032 | AGHY01000994.1:1..2032 | beta-lactamase |
| 2031 | AGHY01000795.1:1..2031 | Outer membrane protein |
| 2031 | AGHY01000571.1:1..2031 | Gamma-glutamyltranspeptidase (EC 2.3.2.2)  Ketol-acid reductoisomerase (EC 1.1.1.86)  Ketol-acid reductoisomerase (EC 1.1.1.86) |
| 2024 | AGHY01000547.1:1..2024 | Chemotaxis protein methyltransferase CheR (EC 2.1.1.80)  diguanylate cyclase/phosphodiesterase (GGDEF  hypothetical protein |
| 2021 | AGHY01000846.1:1..2021 | COG2833: uncharacterized protein  COG2833: uncharacterized protein  peptidyl-Asp metalloendopeptidase |
| 2020 | AGHY01001807.1:1..2020 | hypothetical protein  tRNA dihydrouridine synthase B (EC 1.-.-.-) |
| 2020 | AGHY01001322.1:1..2020 | LSU ribosomal protein L14p (L23e)  LSU ribosomal protein L16p (L10e)  LSU ribosomal protein L29p (L35e)  LSU ribosomal protein L5p (L11e)  SSU ribosomal protein S17p (S11e) |
| 2019 | AGHY01001667.1:1..2019 | Acetyltransferase  hypothetical protein |
| 2006 | AGHY01002048.1:1..2006 | Flagellar biosynthesis protein FlhB |
| 2006 | AGHY01000640.1:1..2006 | Transcriptional regulator2C MarR family  hypothetical protein |
| 2004 | AGHY01001146.1:1..2004 | hypothetical protein |
| 2001 | AGHY01000948.1:1..2001 | Flagellar regulatory protein FleQ  RNA polymerase sigma-54 factor RpoN  response regulator |
| 2001 | AGHY01000243.1:1..2001 | TonB-dependent receptor |
| 1996 | AGHY01001221.1:1..1996 | EAL domain protein  hypothetical protein  hypothetical protein |
| 1996 | AGHY01000910.1:1..1996 | INTEGRAL MEMBRANE PROTEIN (Rhomboid family)  Transcription termination factor Rho |
| 1995 | AGHY01000163.1:1..1995 | Putative large exoprotein involved in heme utilization or adhesion of ShlA/HecA/FhaA family |
| 1987 | AGHY01001511.1:1..1987 | hypothetical protein  hypothetical protein  putative secreted protein |
| 1987 | AGHY01001098.1:1..1987 | Taurine transport system permease protein TauC  hypothetical protein |
| 1987 | AGHY01000208.1:1..1987 | probable outer membrane protein precursor |
| 1979 | AGHY01000519.1:1..1979 | 3-oxoacyl-[acyl-carrier-protein] synthase2C KASIII (EC 2.3.1.41)  3-oxoacyl-[acyl-carrier-protein] synthase2C KASIII (EC 2.3.1.41)  COG1399 protein2C clustered with ribosomal protein L32p  LSU ribosomal protein L32p |
| 1978 | AGHY01000616.1:1..1978 |  |
| 1976 | AGHY01001091.1:1..1976 | Alpha-12C2-mannosidase  Alpha-12C2-mannosidase |
| 1975 | AGHY01000637.1:1..1975 | FIG01211069: hypothetical protein |
| 1974 | AGHY01001325.1:1..1974 | Bacterioferritin  Bacterioferritin  Peroxiredoxin  low molecular weight heat shock protein |
| 1973 | AGHY01001006.1:1..1973 | FIG01211946: hypothetical protein  cytidine and deoxycytidylate deaminase family protein |
| 1971 | AGHY01001987.1:1..1971 | Exodeoxyribonuclease III (EC 3.1.11.2) |
| 1968 | AGHY01000313.1:1..1968 | NAD-specific glutamate dehydrogenase (EC 1.4.1.2)2C large form |
| 1966 | AGHY01001503.1:1..1966 | LigA  RND efflux system2C inner membrane transporter CmeB |
| 1961 | AGHY01000030.1:1..1961 |  |
| 1959 | AGHY01000096.1:1..1959 | S-adenosylmethionine synthetase (EC 2.5.1.6) |
| 1953 | AGHY01000778.1:1..1953 | hypothetical protein |
| 1952 | AGHY01000613.1:1..1952 |  |
| 1951 | AGHY01000827.1:1..1951 | HNH endonuclease family protein |
| 1950 | AGHY01000516.1:1..1950 | FIG00537023: hypothetical protein  Vibrioferrin decarboxylase protein PvsE |
| 1947 | AGHY01001202.1:1..1947 | hypothetical protein |
| 1946 | AGHY01001130.1:1..1946 |  |
| 1946 | AGHY01000053.1:1..1946 | FIG01211497: hypothetical protein  Glyoxalase/Bleomycin resistance protein/Dioxygenase family protein  hypothetical protein |
| 1945 | AGHY01000365.1:1..1945 | Alcohol dehydrogenase (EC 1.1.1.1)  hypothetical protein |
| 1945 | AGHY01000101.1:1..1945 | PE\_PGRS family protein |
| 1940 | AGHY01000300.1:1..1940 |  |
| 1939 | AGHY01000810.1:1..1939 | Transporter2C LysE family  hypothetical protein  hypothetical protein |
| 1937 | AGHY01000017.1:1..1937 | LSU ribosomal protein L6p (L9e)  SSU ribosomal protein S14p (S29e)  SSU ribosomal protein S8p (S15Ae)  hypothetical protein |
| 1933 | AGHY01001419.1:1..1933 | DNA recombination-dependent growth factor C  tRNA(Cytosine32)-2-thiocytidine synthetase |
| 1933 | AGHY01000631.1:1..1933 | Chemotaxis protein methyltransferase CheR (EC 2.1.1.80) |
| 1932 | AGHY01000226.1:1..1932 | hypothetical protein  putative AtsE |
| 1925 | AGHY01001392.1:1..1925 | FIG01211261: hypothetical protein  Methylcrotonyl-CoA carboxylase biotin-containing subunit (EC 6.4.1.4)  cold-shock DNA-binding domain protein |
| 1924 | AGHY01000615.1:1..1924 | Ferrichrome-iron receptor  hypothetical protein |
| 1924 | AGHY01000550.1:1..1924 | Diacylglycerol kinase (EC 2.7.1.107)  Integral membrane protein TerC |
| 1923 | AGHY01000085.1:1..1923 | hypothetical protein |
| 1919 | AGHY01001263.1:1..1919 | 33 kDa chaperonin (Heat shock protein 33) (HSP33)  FIG01211173: hypothetical protein |
| 1915 | AGHY01001268.1:1..1915 | Acetyl-coenzyme A carboxyl transferase beta chain (EC 6.4.1.2)  Phosphoglucosamine mutase (EC 5.4.2.10) |
| 1910 | AGHY01001550.1:1..1910 | FIG01210424: hypothetical protein  transferase2C putative |
| 1910 | AGHY01001118.1:1..1910 | Two-component sensor PilS |
| 1909 | AGHY01001331.1:1..1909 | Glucans biosynthesis protein D precursor  hypothetical protein |
| 1907 | AGHY01001424.1:1..1907 | ABC transporter ATP-binding protein |
| 1904 | AGHY01001866.1:1..1904 | DNA polymerase I (EC 2.7.7.7) |
| 1901 | AGHY01000881.1:1..1901 | Hypothetical ABC transport system2C periplasmic component  hypothetical protein  hypothetical protein |
| 1897 | AGHY01001479.1:1..1897 | 5-methyltetrahydrofolate--homocysteine methyltransferase (EC 2.1.1.13)  5-methyltetrahydrofolate--homocysteine methyltransferase (EC 2.1.1.13)  hypothetical protein |
| 1897 | AGHY01001155.1:1..1897 | Transcriptional regulator lysR family  transcriptional regulator uid family |
| 1895 | AGHY01000049.1:1..1895 | histidine kinase-response regulator hybrid protein  hypothetical protein  single-domain response regulator |
| 1894 | AGHY01001628.1:1..1894 | Hemolysin III |
| 1894 | AGHY01000009.1:1..1894 | hypothetical protein |
| 1893 | AGHY01000461.1:1..1893 | Methylcrotonyl-CoA carboxylase carboxyl transferase subunit (EC 6.4.1.4) |
| 1891 | AGHY01001123.1:1..1891 | Proton/glutamate symport protein @ Sodium/glutamate symport protein  Proton/glutamate symport protein @ Sodium/glutamate symport protein  Proton/glutamate symport protein @ Sodium/glutamate symport protein |
| 1890 | AGHY01002647.1:1..1890 | LigA  hypothetical protein |
| 1890 | AGHY01000943.1:1..1890 | (3R)-hydroxymyristoyl-[acyl carrier protein] dehydratase (EC 4.2.1.-)  Acyl-[acyl-carrier-protein]--UDP-N-acetylglucosamine O-acyltransferase (EC 2.3.1.129)  UDP-3-O-[3-hydroxymyristoyl] glucosamine N-acyltransferase (EC 2.3.1.-) |
| 1889 | AGHY01000590.1:1..1889 | NAD-dependent glyceraldehyde-3-phosphate dehydrogenase (EC 1.2.1.12)  Outer membrane protein W precursor |
| 1888 | AGHY01000355.1:1..1888 | Preprotein translocase subunit SecE (TC 3.A.5.1.1)  Transcription antitermination protein NusG  tRNA-Trp-CCA |
| 1886 | AGHY01000855.1:1..1886 | transcriptional regulator marR family |
| 1878 | AGHY01000233.1:1..1878 |  |
| 1874 | AGHY01000257.1:1..1874 | Translation elongation factor LepA |
| 1873 | AGHY01000386.1:1..1873 |  |
| 1872 | AGHY01001056.1:1..1872 | Aminopeptidase  aminopeptidase  transcriptional regulator lacI family |
| 1872 | AGHY01000765.1:1..1872 | Glucose-1-phosphate thymidylyltransferase (EC 2.7.7.24)  dTDP-4-dehydrorhamnose 32C5-epimerase (EC 5.1.3.13)  dTDP-glucose 42C6-dehydratase (EC 4.2.1.46) |
| 1870 | AGHY01000578.1:1..1870 | peptidase M28 |
| 1869 | AGHY01000022.1:1..1869 | hypothetical protein |
| 1862 | AGHY01000989.1:1..1862 | two-component system sensor protein |
| 1862 | AGHY01000696.1:1..1862 | alanyl dipeptidyl peptidase |
| 1861 | AGHY01000627.1:1..1861 | Proposed peptidoglycan lipid II flippase MurJ  SSU ribosomal protein S20p |
| 1856 | AGHY01000072.1:1..1856 |  |
| 1854 | AGHY01000935.1:1..1854 | 2-methylcitrate synthase (EC 2.3.3.5)  Acid-resistant locus arl7 (Fragment) |
| 1849 | AGHY01001619.1:1..1849 | putative tetratricopeptide repeat family protein |
| 1847 | AGHY01000291.1:1..1847 |  |
| 1846 | AGHY01000196.1:1..1846 |  |
| 1845 | AGHY01001288.1:1..1845 | 1-hydroxy-2-methyl-2-(E)-butenyl 4-diphosphate synthase (EC 1.17.7.1)  1-hydroxy-2-methyl-2-(E)-butenyl 4-diphosphate synthase (EC 1.17.7.1)  phosphatidylglycerophosphatase B-related protein |
| 1844 | AGHY01000026.1:1..1844 | D-serine/D-alanine/glycine transporter  hypothetical protein |
| 1843 | AGHY01001571.1:1..1843 | FIG01212093: hypothetical protein  hypothetical protein  hypothetical protein |
| 1842 | AGHY01000261.1:1..1842 | Pca regulon regulatory protein PcaR  Transporter2C MFS superfamily |
| 1838 | AGHY01001547.1:1..1838 | 4-amino-4-deoxy-L-arabinose transferase and related glycosyltransferases of PMT family |
| 1835 | AGHY01001623.1:1..1835 | FIG01211108: hypothetical protein  Translation elongation factor P Lys34:lysine transferase |
| 1834 | AGHY01001030.1:1..1834 | Beta-xylosidase (EC 3.2.1.37)  sialic acid-specific 9-O-acetylesterase |
| 1834 | AGHY01000298.1:1..1834 | dehydrogenase |
| 1834 | AGHY01000238.1:1..1834 | 4-hydroxyphenylpyruvate dioxygenase (EC 1.13.11.27) |
| 1833 | AGHY01000722.1:1..1833 | FIG01210164: hypothetical protein |
| 1833 | AGHY01000440.1:1..1833 |  |
| 1827 | AGHY01000988.1:1..1827 | FIG01210443: hypothetical protein  FIG01211151: hypothetical protein  hypothetical protein  hypothetical protein |
| 1822 | AGHY01002030.1:1..1822 | N-formylglutamate deformylase (EC 3.5.1.68)  Urocanate hydratase (EC 4.2.1.49) |
| 1820 | AGHY01000679.1:1..1820 | ABC-type Na efflux pump2C permease component  ABC-type Na transport system2C ATPase component |
| 1816 | AGHY01000689.1:1..1816 | hypothetical protein  two-component system sensor protein |
| 1815 | AGHY01002076.1:1..1815 |  |
| 1814 | AGHY01001641.1:1..1814 | N-ethylmaleimide reductase (EC 1.-.-.-) |
| 1814 | AGHY01001413.1:1..1814 | lipopolysaccharide biosynthesis protein  lipopolysaccharide biosynthesis protein |
| 1813 | AGHY01001543.1:1..1813 | FIG027190: Putative transmembrane protein  Putative stomatin/prohibitin-family membrane protease subunit YbbK |
| 1811 | AGHY01001235.1:1..1811 | putative ABC transporter ATP-binding protein |
| 1810 | AGHY01000122.1:1..1810 | FIG01212086: hypothetical protein  transcriptional regulator |
| 1809 | AGHY01001660.1:1..1809 | Outer membrane protein  hypothetical protein |
| 1808 | AGHY01000709.1:1..1808 | ATPase  GDP-mannose pyrophosphatase YffH  hypothetical protein |
| 1806 | AGHY01001356.1:1..1806 | FIG143263: Glycosyl transferase @ Dolichyl-phosphate mannose synthase related protein |
| 1806 | AGHY01000770.1:1..1806 | hypothetical protein  hypothetical protein |
| 1800 | AGHY01001790.1:1..1800 | Integrase |
| 1799 | AGHY01001853.1:1..1799 | CoA tranferase  CoA tranferase |
| 1799 | AGHY01000246.1:1..1799 | hypothetical protein  putative phosphatidylserine decarboxylase( EC:4.1.1.65 ) |
| 1797 | AGHY01000382.1:1..1797 | Translation initiation factor 2 |
| 1795 | AGHY01001622.1:1..1795 |  |
| 1795 | AGHY01001372.1:1..1795 | hypothetical protein  nuclease |
| 1792 | AGHY01001027.1:1..1792 | Ferrochelatase2C protoheme ferro-lyase (EC 4.99.1.1)  Ferrochelatase2C protoheme ferro-lyase (EC 4.99.1.1)  hypothetical protein |
| 1791 | AGHY01000936.1:1..1791 | Biotin synthase related domain containing protein  Putative cytoplasmic protein  hypothetical protein |
| 1788 | AGHY01001180.1:1..1788 | Alpha-amylase |
| 1788 | AGHY01000945.1:1..1788 | Histone acetyltransferase HPA2 and related acetyltransferases |
| 1777 | AGHY01001474.1:1..1777 | cytochrome P450  possible transcriptional regulator2C TetR family |
| 1768 | AGHY01001589.1:1..1768 |  |
| 1767 | AGHY01001552.1:1..1767 | Taurine-binding periplasmic protein TauA |
| 1767 | AGHY01000406.1:1..1767 | hypothetical protein |
| 1767 | AGHY01000133.1:1..1767 | Transport protein |
| 1765 | AGHY01001265.1:1..1765 | Glyoxalase/bleomycin resistance protein/dioxygenase  hypothetical protein |
| 1765 | AGHY01001151.1:1..1765 | Polyribonucleotide nucleotidyltransferase (EC 2.7.7.8)  SSU ribosomal protein S15p (S13e)  tRNA pseudouridine synthase B (EC 4.2.1.70) |
| 1764 | AGHY01000703.1:1..1764 |  |
| 1762 | AGHY01000863.1:1..1762 | ApaG protein  Dimethyladenosine transferase (EC 2.1.1.-) |
| 1762 | AGHY01000701.1:1..1762 | TRAP-type C4-dicarboxylate transport system2C periplasmic component  TRAP-type transport system2C small permease component2C predicted N-acetylneuraminate transporter  TRAP-type transport system2C small permease component2C predicted N-acetylneuraminate transporter |
| 1760 | AGHY01000093.1:1..1760 | FIG01210448: hypothetical protein  Protein sirB1  hypothetical protein  hypothetical protein |
| 1757 | AGHY01001683.1:1..1757 | Short-chain alcohol dehydrogenase family |
| 1757 | AGHY01001320.1:1..1757 | ABC transporter ATP-binding protein  hypothetical protein |
| 1752 | AGHY01000479.1:1..1752 |  |
| 1745 | AGHY01000404.1:1..1745 | Putrescine transport system permease protein PotH (TC 3.A.1.11.2)  Putrescine transport system permease protein PotI (TC 3.A.1.11.2) |
| 1743 | AGHY01002140.1:1..1743 | FIG01211650: hypothetical protein  UDP-N-acetylglucosamine 1-carboxyvinyltransferase (EC 2.5.1.7)  hypothetical protein |
| 1743 | AGHY01000010.1:1..1743 | Alkyl hydroperoxide reductase protein C (EC 1.6.4.-) |
| 1742 | AGHY01000885.1:1..1742 | DNA polymerase III delta prime subunit (EC 2.7.7.7)  Type IV pilus biogenesis protein PilZ  YoeB toxin protein  tRNA-Val-CAC |
| 1739 | AGHY01002091.1:1..1739 | 3'-to-5' oligoribonuclease (orn)  Potassium efflux system KefA protein / Small-conductance mechanosensitive channel  hypothetical protein |
| 1739 | AGHY01001425.1:1..1739 | Biopolymer transport protein ExbD/TolR  Lipid A export ATP-binding/permease protein MsbA (EC 3.6.3.25) |
| 1739 | AGHY01000876.1:1..1739 | 12C4-alpha-glucan branching enzyme (EC 2.4.1.18)  Methyltransferase type 11  hypothetical protein  hypothetical protein |
| 1738 | AGHY01002310.1:1..1738 | hypothetical protein |
| 1736 | AGHY01001556.1:1..1736 | 2-amino-4-hydroxy-6-hydroxymethyldihydropteridine pyrophosphokinase (EC 2.7.6.3)  3-methyl-2-oxobutanoate hydroxymethyltransferase (EC 2.1.2.11)  3-methyl-2-oxobutanoate hydroxymethyltransferase (EC 2.1.2.11) |
| 1735 | AGHY01000857.1:1..1735 | two-component system sensor protein |
| 1735 | AGHY01000227.1:1..1735 |  |
| 1734 | AGHY01001510.1:1..1734 | Pirin-related protein  hypothetical protein |
| 1733 | AGHY01000458.1:1..1733 | Arsenate reductase (EC 1.20.4.1) |
| 1732 | AGHY01000154.1:1..1732 | Isocitrate lyase (EC 4.1.3.1) |
| 1730 | AGHY01001370.1:1..1730 | Alanine dehydrogenase (EC 1.4.1.1)  Omega-amino acid--pyruvate aminotransferase (EC 2.6.1.18) |
| 1729 | AGHY01000602.1:1..1729 | FIG01111128: hypothetical protein  NADPH dependent preQ0 reductase |
| 1727 | AGHY01001763.1:1..1727 | Probable Co/Zn/Cd efflux system membrane fusion protein |
| 1725 | AGHY01001286.1:1..1725 | FIG071884: Hypothetical protein  Integral membrane protein CcmA involved in cell shape determination  probable iron binding protein from the HesB\_IscA\_SufA family |
| 1725 | AGHY01001244.1:1..1725 | hypothetical protein  hypothetical protein  inner membrane protein  putative lipoprotein |
| 1724 | AGHY01001020.1:1..1724 | BarA-associated response regulator UvrY ( GacA SirA) |
| 1723 | AGHY01001523.1:1..1723 | Histidine kinase  hypothetical protein  hypothetical protein |
| 1723 | AGHY01000066.1:1..1723 | Two-component response regulator |
| 1721 | AGHY01000819.1:1..1721 | Gluconate transporter family protein |
| 1718 | AGHY01000775.1:1..1718 | FIG01209855: hypothetical protein |
| 1718 | AGHY01000634.1:1..1718 | FIG01210375: hypothetical protein  probably aromatic ring hydroxylating enzyme2C evidenced by COGnitor; PaaD-like protein (DUF59) involved in Fe-S cluster assembly |
| 1718 | AGHY01000138.1:1..1718 | Electron transfer flavoprotein2C beta subunit  dTDP-glucose 42C6-dehydratase (EC 4.2.1.46) |
| 1717 | AGHY01000980.1:1..1717 | Alpha-L-fucosidase (EC 3.2.1.51) |
| 1715 | AGHY01000393.1:1..1715 |  |
| 1713 | AGHY01001927.1:1..1713 | Beta-lactamase (EC 3.5.2.6) |
| 1708 | AGHY01000552.1:1..1708 |  |
| 1707 | AGHY01001072.1:1..1707 | dicarboxylate transport protein |
| 1703 | AGHY01002239.1:1..1703 | FIG01111779: hypothetical protein |
| 1703 | AGHY01001107.1:1..1703 | ABC transporter permease  ABC-type antimicrobial peptide transport system2C permease component  hypothetical protein |
| 1703 | AGHY01000169.1:1..1703 | 1-acyl-sn-glycerol-3-phosphate acyltransferase (EC 2.3.1.51)  Phosphatidate cytidylyltransferase (EC 2.7.7.41)  Phosphatidate cytidylyltransferase (EC 2.7.7.41) |
| 1702 | AGHY01001600.1:1..1702 | NAD(P) transhydrogenase subunit beta (EC 1.6.1.2) |
| 1700 | AGHY01002077.1:1..1700 | hypothetical protein |
| 1699 | AGHY01002027.1:1..1699 | Putative outer membrane or secreted lipoprotein |
| 1698 | AGHY01000491.1:1..1698 | FIG01211701: hypothetical protein |
| 1697 | AGHY01001682.1:1..1697 |  |
| 1694 | AGHY01001290.1:1..1694 | Ferrichrome-iron receptor |
| 1694 | AGHY01001035.1:1..1694 | Phosphoenolpyruvate-protein phosphotransferase of PTS system (EC 2.7.3.9)  Phosphoenolpyruvate-protein phosphotransferase of PTS system (EC 2.7.3.9) |
| 1694 | AGHY01000366.1:1..1694 | 5'-methylthioadenosine phosphorylase (EC 2.4.2.28)  Beta N-acetyl-glucosaminidase (EC 3.2.1.52)  Hypoxanthine-guanine phosphoribosyltransferase (EC 2.4.2.8) |
| 1693 | AGHY01000826.1:1..1693 | LigA |
| 1691 | AGHY01001120.1:1..1691 | Glucose dehydrogenase2C PQQ-dependent (EC 1.1.5.2) |
| 1691 | AGHY01000210.1:1..1691 | LSU ribosomal protein L28p  LSU ribosomal protein L33p  Transcriptional regulator2C LysR family |
| 1690 | AGHY01001574.1:1..1690 | Putative exported protein precursor  hypothetical protein  tRNA-Gly-GCC |
| 1684 | AGHY01000684.1:1..1684 |  |
| 1682 | AGHY01001154.1:1..1682 |  |
| 1680 | AGHY01000924.1:1..1680 | D-tyrosyl-tRNA(Tyr) deacylase  RNA polymerase sigma factor RpoD |
| 1679 | AGHY01001009.1:1..1679 | FIG01111895: hypothetical protein |
| 1678 | AGHY01000731.1:1..1678 | FIG138056: a glutathione-dependent thiol reductase  hypothetical protein |
| 1677 | AGHY01000541.1:1..1677 | Acetyltransferase  Ferredoxin2C 2Fe-2S |
| 1673 | AGHY01000956.1:1..1673 | Dipeptidyl carboxypeptidase Dcp (EC 3.4.15.5)  Signal peptidase I (EC 3.4.21.89) |
| 1672 | AGHY01002043.1:1..1672 | tRNA-i(6)A37 methylthiotransferase |
| 1671 | AGHY01001437.1:1..1671 | hypothetical protein |
| 1669 | AGHY01001595.1:1..1669 | FIG01209666: hypothetical protein  hypothetical protein  hypothetical protein  tRNA-Ser-CGA |
| 1669 | AGHY01001226.1:1..1669 | Xylulose kinase (EC 2.7.1.17)  Xylulose kinase (EC 2.7.1.17) |
| 1667 | AGHY01001766.1:1..1667 | 2-keto-3-deoxy-L-fuconate dehydrogenase  2-keto-3-deoxy-L-fuconate dehydrogenase  22C4-diketo-3-deoxy-L-fuconate hydrolase |
| 1662 | AGHY01000259.1:1..1662 | Multimodular transpeptidase-transglycosylase (EC 2.4.1.129) (EC 3.4.-.-) |
| 1658 | AGHY01000804.1:1..1658 | Beta-mannosidase (EC 3.2.1.25) |
| 1658 | AGHY01000348.1:1..1658 |  |
| 1655 | AGHY01002203.1:1..1655 | 23S rRNA (guanosine-2'-O-) -methyltransferase rlmB (EC 2.1.1.-) |
| 1655 | AGHY01000500.1:1..1655 | hypothetical protein  hypothetical protein  hypothetical protein |
| 1654 | AGHY01000568.1:1..1654 | Organic hydroperoxide resistance protein  putative exported protein |
| 1652 | AGHY01001292.1:1..1652 | LysR family transcriptional regulator YbhD |
| 1651 | AGHY01000572.1:1..1651 | ClpB protein  ClpB protein |
| 1651 | AGHY01000360.1:1..1651 |  |
| 1650 | AGHY01000130.1:1..1650 |  |
| 1649 | AGHY01000589.1:1..1649 | Isoleucyl-tRNA synthetase (EC 6.1.1.5) |
| 1648 | AGHY01001773.1:1..1648 | TonB-dependent receptor  ferric enterobactin receptor |
| 1648 | AGHY01000894.1:1..1648 | Signal recognition particle2C subunit Ffh SRP54 (TC 3.A.5.1.1) |
| 1648 | AGHY01000450.1:1..1648 | Methionine aminopeptidase (EC 3.4.11.18)  Sigma-fimbriae tip adhesin |
| 1645 | AGHY01001185.1:1..1645 | hypothetical protein |
| 1644 | AGHY01000454.1:1..1644 | Dipeptidyl peptidase IV in 4-hydroxyproline catabolic gene cluster  Dipeptidyl peptidase IV in 4-hydroxyproline catabolic gene cluster |
| 1639 | AGHY01001191.1:1..1639 | Inosine-5'-monophosphate dehydrogenase (EC 1.1.1.205)  Inosine-5'-monophosphate dehydrogenase (EC 1.1.1.205) |
| 1637 | AGHY01000966.1:1..1637 | 3-ketoacyl-CoA thiolase (EC 2.3.1.16) @ Acetyl-CoA acetyltransferase (EC 2.3.1.9) |
| 1636 | AGHY01002121.1:1..1636 | tRNA pseudouridine synthase A (EC 4.2.1.70) |
| 1635 | AGHY01000175.1:1..1635 | periplasmic sensor signal transduction histidine kinase |
| 1632 | AGHY01001174.1:1..1632 | Probable low-affinity inorganic phosphate transporter  hypothetical protein |
| 1631 | AGHY01000919.1:1..1631 | Na( ) H( ) antiporter subunit A; Na( ) H( ) antiporter subunit B  Na( ) H( ) antiporter subunit C |
| 1629 | AGHY01000036.1:1..1629 | Zinc-regulated outer membrane receptor |
| 1628 | AGHY01002314.1:1..1628 | Two-component system regulatory protein |
| 1628 | AGHY01000730.1:1..1628 | Inner membrane protein translocase component YidC2C long form |
| 1623 | AGHY01001093.1:1..1623 | Cysteine synthase (EC 2.5.1.47)  Cysteine synthase (EC 2.5.1.47) |
| 1621 | AGHY01000749.1:1..1621 | Transcriptional regulator  Transcriptional regulator  hypothetical protein  hypothetical protein |
| 1617 | AGHY01000278.1:1..1617 | Isovaleryl-CoA dehydrogenase (EC 1.3.99.10) |
| 1614 | AGHY01001316.1:1..1614 | hypothetical protein  hypothetical protein |
| 1614 | AGHY01000305.1:1..1614 | Flagellar biosynthesis protein FliS  Flagellar hook-associated protein FliD |
| 1611 | AGHY01002467.1:1..1611 | TonB-dependent receptor  TonB-dependent receptor |
| 1611 | AGHY01001169.1:1..1611 | ADP compounds hydrolase NudE (EC 3.6.1.-) |
| 1608 | AGHY01000985.1:1..1608 | Type IV fimbrial assembly2C ATPase PilB |
| 1608 | AGHY01000931.1:1..1608 | Enolase (EC 4.2.1.11) |
| 1608 | AGHY01000927.1:1..1608 | Alpha-12C2-mannosidase  hypothetical protein |
| 1606 | AGHY01001855.1:1..1606 | Galactonate dehydratase (EC 4.2.1.6)  Galactonate dehydratase (EC 4.2.1.6) |
| 1604 | AGHY01000139.1:1..1604 |  |
| 1603 | AGHY01001251.1:1..1603 | C4-type zinc finger protein2C DksA/TraR family  putative secreted protein |
| 1603 | AGHY01000678.1:1..1603 | hypothetical protein  hypothetical protein |
| 1603 | AGHY01000588.1:1..1603 | FIG01112033: hypothetical protein  hydrolase2C haloacid dehalogenase-like family  hypothetical protein |
| 1602 | AGHY01001936.1:1..1602 |  |
| 1601 | AGHY01002108.1:1..1601 |  |
| 1600 | AGHY01001404.1:1..1600 | Putative preQ0 transporter  Putative preQ0 transporter  Transporter |
| 1600 | AGHY01001328.1:1..1600 | 4-hydroxy-3-methylbut-2-enyl diphosphate reductase (EC 1.17.1.2)  Lipoprotein signal peptidase (EC 3.4.23.36) |
| 1599 | AGHY01001954.1:1..1599 | L-serine dehydratase (EC 4.3.1.17) |
| 1594 | AGHY01000790.1:1..1594 | hypothetical protein  hypothetical protein |
| 1591 | AGHY01001581.1:1..1591 | hypothetical protein  hypothetical protein  putative secreted protein |
| 1591 | AGHY01001352.1:1..1591 | hypothetical protein |
| 1589 | AGHY01002282.1:1..1589 | Bifunctional protein: zinc-containing alcohol dehydrogenase; quinone oxidoreductase ( NADPH:quinone reductase) (EC 1.1.1.-); Similar to arginate lyase  Oxidoreductase  hypothetical protein |
| 1586 | AGHY01002229.1:1..1586 | Adenosylmethionine-8-amino-7-oxononanoate aminotransferase (EC 2.6.1.62)  Putrescine ABC transporter putrescine-binding protein PotF (TC 3.A.1.11.2)  Putrescine ABC transporter putrescine-binding protein PotF (TC 3.A.1.11.2) |
| 1586 | AGHY01000756.1:1..1586 | Heme O synthase2C protoheme IX farnesyltransferase (EC 2.5.1.-) COX10-CtaB |
| 1586 | AGHY01000158.1:1..1586 | putative glyoxalase/bleomycin resistance protein/dioxygenase superfamily protein |
| 1586 | AGHY01000004.1:1..1586 | 2-amino-4-hydroxy-6-hydroxymethyldihydropteridine pyrophosphokinase (EC 2.7.6.3)  FIG01111359: hypothetical protein |
| 1585 | AGHY01002514.1:1..1585 | O-acetylhomoserine sulfhydrylase (EC 2.5.1.49) / O-succinylhomoserine sulfhydrylase (EC 2.5.1.48)  O-acetylhomoserine sulfhydrylase (EC 2.5.1.49) / O-succinylhomoserine sulfhydrylase (EC 2.5.1.48) |
| 1585 | AGHY01001272.1:1..1585 | hypothetical protein  putative membrane protein |
| 1583 | AGHY01001021.1:1..1583 |  |
| 1583 | AGHY01000490.1:1..1583 |  |
| 1578 | AGHY01001112.1:1..1578 | Malate dehydrogenase (EC 1.1.1.37)  Malate dehydrogenase (EC 1.1.1.37) |
| 1578 | AGHY01001076.1:1..1578 | 3-deoxy-D-manno-octulosonate 8-phosphate phosphatase (EC 3.1.3.45)  Uncharacterized protein YrbK clustered with lipopolysaccharide transporters |
| 1578 | AGHY01000799.1:1..1578 | hypothetical protein |
| 1577 | AGHY01000334.1:1..1577 | ABC transporter2C ATP-binding protein |
| 1575 | AGHY01001159.1:1..1575 | Prolipoprotein diacylglyceryl transferase (EC 2.4.99.-)  Thymidylate synthase (EC 2.1.1.45) |
| 1575 | AGHY01000780.1:1..1575 | Integration host factor alpha subunit  Phenylalanyl-tRNA synthetase beta chain (EC 6.1.1.20)  Transcriptional regulator2C MerR family  tRNA-Pro-GGG |
| 1574 | AGHY01001125.1:1..1574 | Carboxyl-terminal protease (EC 3.4.21.102)  Carboxyl-terminal protease (EC 3.4.21.102)  Cell wall endopeptidase2C family M23/M37 |
| 1573 | AGHY01001319.1:1..1573 | Aromatic-amino-acid aminotransferase (EC 2.6.1.57)  hypothetical protein |
| 1571 | AGHY01001759.1:1..1571 | Fatty acid desaturase (EC 1.14.19.1); Delta-9 fatty acid desaturase (EC 1.14.19.1) |
| 1571 | AGHY01001458.1:1..1571 | Ubiquinone biosynthesis monooxygenase UbiB |
| 1566 | AGHY01000638.1:1..1566 | 3-beta hydroxysteroid dehydrogenase/isomerase family protein in hypothetical gene cluster  hypothetical protein  hypothetical protein |
| 1565 | AGHY01001800.1:1..1565 | Dihydrolipoamide dehydrogenase of 2-oxoglutarate dehydrogenase (EC 1.8.1.4)  Dihydrolipoamide dehydrogenase of 2-oxoglutarate dehydrogenase (EC 1.8.1.4) |
| 1565 | AGHY01001271.1:1..1565 | ATP-dependent hsl protease ATP-binding subunit HslU  ATP-dependent hsl protease ATP-binding subunit HslU |
| 1564 | AGHY01000916.1:1..1564 | hypothetical protein |
| 1562 | AGHY01000858.1:1..1562 |  |
| 1561 | AGHY01000145.1:1..1561 | Dipeptidyl peptidase IV |
| 1561 | AGHY01000067.1:1..1561 |  |
| 1559 | AGHY01001309.1:1..1559 | hypothetical protein  hypothetical protein  oxidoreductase |
| 1558 | AGHY01000554.1:1..1558 | Dethiobiotin synthetase (EC 6.3.3.3)  FIG01210979: hypothetical protein  Queuosine biosynthesis QueD2C PTPS-I |
| 1556 | AGHY01001824.1:1..1556 | FIG01210445: hypothetical protein  Hypothetical Zinc-finger containing protein  Small-conductance mechanosensitive channel  nucleoprotein/polynucleotide-associated enzyme |
| 1554 | AGHY01001841.1:1..1554 | 4-hydroxythreonine-4-phosphate dehydrogenase (EC 1.1.1.262)  Dimethyladenosine transferase (EC 2.1.1.-) |
| 1554 | AGHY01001141.1:1..1554 | hypothetical protein |
| 1553 | AGHY01001462.1:1..1553 |  |
| 1553 | AGHY01000451.1:1..1553 | Dihydrodipicolinate synthase (EC 4.2.1.52)  Transporter2C MFS superfamily |
| 1552 | AGHY01001124.1:1..1552 | Acriflavin resistance protein  Acriflavin resistance protein  hypothetical protein |
| 1550 | AGHY01002220.1:1..1550 |  |
| 1548 | AGHY01000574.1:1..1548 | Homogentisate 12C2-dioxygenase (EC 1.13.11.5)  Homogentisate 12C2-dioxygenase (EC 1.13.11.5) |
| 1545 | AGHY01000595.1:1..1545 | DNA-binding protein H-NS  Prolyl-tRNA synthetase (EC 6.1.1.15)2C bacterial type  hypothetical protein |
| 1541 | AGHY01001964.1:1..1541 | MFS transporter  hypothetical protein |
| 1541 | AGHY01001493.1:1..1541 | sensory box histidine kinase/response regulator |
| 1541 | AGHY01001321.1:1..1541 | GCN5-related N-acetyltransferase  Transcription regulator [contains diacylglycerol kinase catalytic domain] |
| 1541 | AGHY01000997.1:1..1541 | Phospho-N-acetylmuramoyl-pentapeptide-transferase (EC 2.7.8.13) |
| 1540 | AGHY01000567.1:1..1540 | Chemotaxis regulator - transmits chemoreceptor signals to flagelllar motor components CheY  FIG01209829: hypothetical protein  Positive regulator of CheA protein activity (CheW) |
| 1538 | AGHY01000597.1:1..1538 | tRNA-Ser-GGA |
| 1536 | AGHY01000474.1:1..1536 | putative cytoplasmic protein |
| 1532 | AGHY01001339.1:1..1532 | RNA polymerase sigma factor RpoE  RNA polymerase sigma factor RpoE |
| 1526 | AGHY01001977.1:1..1526 | Carboxypeptidase C (cathepsin A) |
| 1522 | AGHY01001451.1:1..1522 | hypothetical protein |
| 1517 | AGHY01000403.1:1..1517 | Glycosyl transferase2C family 2  GumO  putative acyltransferase (PhnO) |
| 1517 | AGHY01000363.1:1..1517 | Serine hydroxymethyltransferase (EC 2.1.2.1) |
| 1516 | AGHY01001074.1:1..1516 | Methyl-accepting chemotaxis protein I (serine chemoreceptor protein) |
| 1514 | AGHY01000008.1:1..1514 | Sensory/regulatory protein rpfC (EC 2.7.3.-)  hypothetical protein |
| 1512 | AGHY01001149.1:1..1512 | N-acetylglucosamine-6-phosphate deacetylase (EC 3.5.1.25)  hypothetical protein |
| 1512 | AGHY01000830.1:1..1512 | Glutamate--cysteine ligase (EC 6.3.2.2) |
| 1511 | AGHY01000671.1:1..1511 |  |
| 1505 | AGHY01001137.1:1..1505 | 3-oxoacyl-[acyl-carrier protein] reductase (EC 1.1.1.100)  Exodeoxyribonuclease V alpha chain (EC 3.1.11.5) |
| 1503 | AGHY01001063.1:1..1503 | FIG01214235: hypothetical protein  hypothetical protein |
| 1501 | AGHY01001470.1:1..1501 | TldE/PmbA protein2C part of proposed TldE/TldD proteolytic complex (PMID 12029038)  hypothetical protein  hypothetical protein |
| 1500 | AGHY01001770.1:1..1500 |  |
| 1500 | AGHY01000726.1:1..1500 | Acyl-CoA-binding protein  FIG01210541: hypothetical protein |
| 1500 | AGHY01000593.1:1..1500 |  |
| 1498 | AGHY01001835.1:1..1498 | Fumarate hydratase class II (EC 4.2.1.2) |
| 1498 | AGHY01000979.1:1..1498 | hemagglutinin |
| 1496 | AGHY01001230.1:1..1496 | FIG053235: Diacylglucosamine hydrolase like  Transcriptional regulator2C LuxR family |
| 1496 | AGHY01001217.1:1..1496 |  |
| 1496 | AGHY01000604.1:1..1496 |  |
| 1495 | AGHY01001838.1:1..1495 |  |
| 1495 | AGHY01001081.1:1..1495 |  |
| 1494 | AGHY01000733.1:1..1494 | FIG01211114: hypothetical protein  FIG01211150: hypothetical protein |
| 1493 | AGHY01000844.1:1..1493 | Ribonuclease BN (EC 3.1.-.-) |
| 1492 | AGHY01001916.1:1..1492 |  |
| 1487 | AGHY01001267.1:1..1487 | CDP-glycerol: N-acetyl-beta-D-mannosaminyl-12C4-N-acetyl-D-glucosaminyldiphosphoundecaprenyl glycerophosphotransferase  beta 12C4 glucosyltransferase |
| 1486 | AGHY01000890.1:1..1486 | RpfF protein  Sensory/regulatory protein rpfC (EC 2.7.3.-) |
| 1485 | AGHY01000639.1:1..1485 | Glutamine synthetase type I (EC 6.3.1.2) |
| 1484 | AGHY01001034.1:1..1484 | Peptide deformylase (EC 3.5.1.88) |
| 1483 | AGHY01002247.1:1..1483 | Phosphoribosylamine--glycine ligase (EC 6.3.4.13) |
| 1483 | AGHY01001537.1:1..1483 |  |
| 1483 | AGHY01000923.1:1..1483 | Vibriolysin2C extracellular zinc protease (EC 3.4.24.25) @ Pseudolysin2C extracellular zinc protease (EC 3.4.24.26)  hypothetical protein |
| 1481 | AGHY01000776.1:1..1481 | Putative inner membrane protein  hypothetical protein |
| 1481 | AGHY01000213.1:1..1481 | hypothetical protein  hypothetical protein |
| 1480 | AGHY01001791.1:1..1480 | UPF0234 protein YajQ  hypothetical protein |
| 1479 | AGHY01001591.1:1..1479 | ATP-dependent protease HslV (EC 3.4.25.-)  Site-specific tyrosine recombinase  hypothetical protein |
| 1479 | AGHY01000544.1:1..1479 | Peptide methionine sulfoxide reductase MsrA (EC 1.8.4.11) |
| 1478 | AGHY01001197.1:1..1478 | Fructose-bisphosphate aldolase class I (EC 4.1.2.13) |
| 1477 | AGHY01002778.1:1..1477 | diguanylate cyclase/phosphodiesterase (GGDEF |
| 1476 | AGHY01000599.1:1..1476 | Hypothetical protein DUF1942C DegV family |
| 1476 | AGHY01000375.1:1..1476 | Membrane protein2C putative  Putative diheme cytochrome c-553  hypothetical protein |
| 1474 | AGHY01001584.1:1..1474 | Pyruvate kinase (EC 2.7.1.40)  Pyruvate kinase (EC 2.7.1.40) |
| 1472 | AGHY01001925.1:1..1472 | hypothetical protein |
| 1471 | AGHY01002158.1:1..1471 |  |
| 1471 | AGHY01000645.1:1..1471 | Acriflavin resistance protein  Acriflavin resistance protein |
| 1470 | AGHY01000758.1:1..1470 | Twitching motility protein PilT |
| 1466 | AGHY01002350.1:1..1466 | Exodeoxyribonuclease I (EC 3.1.11.1)  FIG01212834: hypothetical protein |
| 1466 | AGHY01000135.1:1..1466 | L-arabonate dehydratase (EC 4.2.1.25) |
| 1464 | AGHY01001669.1:1..1464 | N-acetylornithine carbamoyltransferase (EC 2.1.3.9) |
| 1464 | AGHY01000840.1:1..1464 | GTP-binding protein HflX  RNA-binding protein Hfq |
| 1462 | AGHY01001366.1:1..1462 | Sensor histidine kinase |
| 1458 | AGHY01001662.1:1..1458 | 4-diphosphocytidyl-2-C-methyl-D-erythritol kinase (EC 2.7.1.148)  Ribose-phosphate pyrophosphokinase (EC 2.7.6.1)  tRNA-Gln-TTG |
| 1457 | AGHY01001279.1:1..1457 | hypothetical protein |
| 1457 | AGHY01001065.1:1..1457 | Benzoate transport protein |
| 1455 | AGHY01001840.1:1..1455 | Cell division topological specificity factor MinE  Septum site-determining protein MinC  Septum site-determining protein MinD  Septum site-determining protein MinD |
| 1453 | AGHY01000159.1:1..1453 | Flagellar L-ring protein FlgH  Flagellar P-ring protein FlgI |
| 1451 | AGHY01001980.1:1..1451 | Xanthan biosynthesis exopolysaccharide polymerase GumE |
| 1451 | AGHY01001691.1:1..1451 | Ammonium transporter |
| 1450 | AGHY01000755.1:1..1450 | hypothetical protein |
| 1449 | AGHY01000621.1:1..1449 |  |
| 1448 | AGHY01000791.1:1..1448 | ggdef domain protein2C putative |
| 1445 | AGHY01000941.1:1..1445 |  |
| 1444 | AGHY01000879.1:1..1444 | DNA mismatch repair protein MutS |
| 1444 | AGHY01000852.1:1..1444 | Cyanophycinase and related exopeptidases-like |
| 1444 | AGHY01000120.1:1..1444 |  |
| 1442 | AGHY01001480.1:1..1442 | hypothetical protein  probable outer membrane protein |
| 1441 | AGHY01000918.1:1..1441 | phage integrase family protein  phage integrase family protein |
| 1439 | AGHY01000729.1:1..1439 | FIG00960671: hypothetical protein  hypothetical protein |
| 1439 | AGHY01000608.1:1..1439 | A/G-specific adenine glycosylase (EC 3.2.2.-) |
| 1439 | AGHY01000607.1:1..1439 |  |
| 1438 | AGHY01000940.1:1..1438 | Enoyl-CoA hydratase (EC 4.2.1.17) / 3-hydroxyacyl-CoA dehydrogenase (EC 1.1.1.35) / 3-hydroxybutyryl-CoA epimerase (EC 5.1.2.3)  Enoyl-CoA hydratase (EC 4.2.1.17) / 3-hydroxyacyl-CoA dehydrogenase (EC 1.1.1.35) / 3-hydroxybutyryl-CoA epimerase (EC 5.1.2.3) |
| 1436 | AGHY01001059.1:1..1436 | Ferrous iron transport protein B  Ferrous iron transport protein B |
| 1434 | AGHY01000891.1:1..1434 | RND efflux system2C membrane fusion protein CmeA |
| 1432 | AGHY01001289.1:1..1432 | Type II secretion system protein-like protein  Type II secretion system protein-like protein |
| 1429 | AGHY01002047.1:1..1429 | NfuA Fe-S protein maturation  Pterin-4-alpha-carbinolamine dehydratase (EC 4.2.1.96) |
| 1429 | AGHY01000822.1:1..1429 | Oar protein |
| 1423 | AGHY01000180.1:1..1423 |  |
| 1422 | AGHY01002409.1:1..1422 | FIG01211220: hypothetical protein  Flagellar motor rotation protein MotA |
| 1420 | AGHY01001207.1:1..1420 | Transmembrane protein  conserved hypothetical protein |
| 1420 | AGHY01000269.1:1..1420 | FIG01210385: hypothetical protein |
| 1419 | AGHY01001515.1:1..1419 |  |
| 1418 | AGHY01000327.1:1..1418 | hypothetical protein |
| 1417 | AGHY01002024.1:1..1417 | Ribosomal protein S12p Asp88 (E. coli) methylthiotransferase |
| 1414 | AGHY01002176.1:1..1414 | 2-C-methyl-D-erythritol 22C4-cyclodiphosphate synthase (EC 4.6.1.12)  hypothetical protein |
| 1414 | AGHY01001236.1:1..1414 | Hemolysins and related proteins containing CBS domains |
| 1412 | AGHY01001025.1:1..1412 | Organic hydroperoxide resistance protein |
| 1412 | AGHY01000005.1:1..1412 |  |
| 1411 | AGHY01001675.1:1..1411 |  |
| 1411 | AGHY01000700.1:1..1411 | Biotin carboxylase of acetyl-CoA carboxylase (EC 6.3.4.14)  Biotin carboxylase of acetyl-CoA carboxylase (EC 6.3.4.14) |
| 1410 | AGHY01001808.1:1..1410 |  |
| 1407 | AGHY01001828.1:1..1407 | transcriptional regulator lacI family |
| 1407 | AGHY01001742.1:1..1407 | 12C4-alpha-glucan (glycogen) branching enzyme2C GH-13-type (EC 2.4.1.18) |
| 1407 | AGHY01000270.1:1..1407 | PlcB2C ORFX2C ORFP2C ORFB2C ORFA2C ldh gene  hypothetical protein |
| 1405 | AGHY01000507.1:1..1405 | FIG00957062: hypothetical protein |
| 1405 | AGHY01000319.1:1..1405 | Butyryl-CoA dehydrogenase (EC 1.3.99.2)  Butyryl-CoA dehydrogenase (EC 1.3.99.2) |
| 1404 | AGHY01002182.1:1..1404 |  |
| 1402 | AGHY01001528.1:1..1402 | ankyrin-like protein  protease |
| 1401 | AGHY01002374.1:1..1401 | coagulation factor 5/8 type domain protein |
| 1399 | AGHY01001638.1:1..1399 | Uracil phosphoribosyltransferase (EC 2.4.2.9)  cellulase precursor( EC:3.2.1.4 ) |
| 1399 | AGHY01001604.1:1..1399 |  |
| 1399 | AGHY01001551.1:1..1399 | cytochrome C  hypothetical protein |
| 1397 | AGHY01001965.1:1..1397 | hypothetical protein  hypothetical protein  hypothetical protein |
| 1397 | AGHY01001429.1:1..1397 | Macrophage infectivity potentiator  hypothetical protein |
| 1397 | AGHY01001269.1:1..1397 | LSU ribosomal protein L13p (L13Ae)  SSU ribosomal protein S9p (S16e) |
| 1397 | AGHY01001080.1:1..1397 |  |
| 1395 | AGHY01001699.1:1..1395 | Fumarylacetoacetate hydrolase family protein  Large-conductance mechanosensitive channel  peptidase M28 |
| 1395 | AGHY01001481.1:1..1395 | transport protein |
| 1394 | AGHY01002464.1:1..1394 | 3-ketoacyl-CoA thiolase (EC 2.3.1.16) @ Acetyl-CoA acetyltransferase (EC 2.3.1.9) |
| 1389 | AGHY01001908.1:1..1389 | putative salt-induced outer membrane protein |
| 1384 | AGHY01000127.1:1..1384 | Polyribonucleotide nucleotidyltransferase (EC 2.7.7.8) |
| 1383 | AGHY01000171.1:1..1383 | hypothetical protein |
| 1382 | AGHY01000399.1:1..1382 | Osmotically inducible protein OsmY |
| 1382 | AGHY01000150.1:1..1382 | Putative cytoplasmic protein  hypothetical protein |
| 1381 | AGHY01001910.1:1..1381 | hypothetical protein  hypothetical protein |
| 1380 | AGHY01001555.1:1..1380 | PQQ-dependent oxidoreductase2C gdhB family  PQQ-dependent oxidoreductase2C gdhB family |
| 1378 | AGHY01001748.1:1..1378 | Cystathionine gamma-synthase (EC 2.5.1.48)  Homoserine O-acetyltransferase (EC 2.3.1.31) |
| 1378 | AGHY01001646.1:1..1378 | Alkylphosphonate utilization operon protein PhnA  cation transport protein |
| 1377 | AGHY01002529.1:1..1377 | Putative deoxyribonuclease YjjV |
| 1377 | AGHY01001466.1:1..1377 | Inner membrane protein |
| 1377 | AGHY01000977.1:1..1377 | efflux transporter2C RND family2C MFP subunit |
| 1376 | AGHY01000760.1:1..1376 | FIG01210377: hypothetical protein |
| 1375 | AGHY01001162.1:1..1375 | Lytic enzyme  hypothetical protein |
| 1374 | AGHY01001926.1:1..1374 | Cytochrome d ubiquinol oxidase subunit I (EC 1.10.3.-) |
| 1373 | AGHY01000665.1:1..1373 | ABC transporter permease  Histidine kinase/response regulator hybrid protein |
| 1372 | AGHY01001376.1:1..1372 | phosphate-binding protein |
| 1372 | AGHY01000417.1:1..1372 | Methionine ABC transporter ATP-binding protein |
| 1370 | AGHY01001820.1:1..1370 | 2-hydroxy-3-keto-5-methylthiopentenyl-1-phosphate phosphatase related protein |
| 1365 | AGHY01001883.1:1..1365 | hypothetical protein |
| 1364 | AGHY01001038.1:1..1364 |  |
| 1363 | AGHY01000081.1:1..1363 | hypothetical protein  hypothetical protein |
| 1361 | AGHY01001782.1:1..1361 |  |
| 1361 | AGHY01001386.1:1..1361 | Transcriptional regulator2C ArsR family  rhodanese-like domain protein |
| 1359 | AGHY01002223.1:1..1359 | Leucine-responsive regulatory protein2C regulator for leucine (or lrp) regulon and high-affinity branched-chain amino acid transport system  carbonic anhydrase2C family 3 |
| 1359 | AGHY01000811.1:1..1359 | LigA  Lytic transglycosylase2C catalytic  hypothetical protein |
| 1359 | AGHY01000725.1:1..1359 |  |
| 1355 | AGHY01001068.1:1..1355 | Na( ) H( ) antiporter subunit E  Na( ) H( ) antiporter subunit F  Na( ) H( ) antiporter subunit G  hypothetical protein |
| 1354 | AGHY01002728.1:1..1354 | FAD linked oxidase-like  oxidoreductase |
| 1354 | AGHY01001798.1:1..1354 | RpfN protein |
| 1354 | AGHY01000136.1:1..1354 | Na -driven multidrug efflux pump  protease IV |
| 1353 | AGHY01000933.1:1..1353 | hypothetical protein |
| 1352 | AGHY01001088.1:1..1352 | Cysteine desulfurase (EC 2.8.1.7)2C SufS subfamily  Cysteine desulfurase (EC 2.8.1.7)2C SufS subfamily  Cysteine desulfurase (EC 2.8.1.7)2C SufS subfamily |
| 1352 | AGHY01000046.1:1..1352 | regulator of pathogenicity factors |
| 1350 | AGHY01001831.1:1..1350 | CBSS-498211.3.peg.1514: hypothetical protein  GTP cyclohydrolase I (EC 3.5.4.16) type 1 |
| 1349 | AGHY01002135.1:1..1349 | FIG01210342: hypothetical protein  Flavodoxin reductases (ferredoxin-NADPH reductases) family 1; Vanillate O-demethylase oxidoreductase (EC 1.14.13.-) |
| 1349 | AGHY01000798.1:1..1349 | Flagellar M-ring protein FliF  Flagellar hook-basal body complex protein FliE |
| 1344 | AGHY01000932.1:1..1344 | Outer membrane protein  hypothetical protein |
| 1344 | AGHY01000708.1:1..1344 | Flagellar motor switch protein FliG |
| 1342 | AGHY01001500.1:1..1342 |  |
| 1341 | AGHY01001239.1:1..1341 | Uncharacterized glutathione S-transferase-like protein  inner-membrane translocator |
| 1340 | AGHY01001187.1:1..1340 |  |
| 1339 | AGHY01001893.1:1..1339 | FIG01212366: hypothetical protein  hypothetical protein |
| 1338 | AGHY01000785.1:1..1338 | FIG00446866: hypothetical protein  MgtC/SapB transporter |
| 1337 | AGHY01001705.1:1..1337 | FIG138928: iron-regulated membrane protein |
| 1337 | AGHY01001650.1:1..1337 | Type IV pilus biogenesis protein PilO  Type IV pilus biogenesis protein PilP |
| 1336 | AGHY01001092.1:1..1336 | 3-oxoacyl-[acyl-carrier protein] reductase (EC 1.1.1.100)  putative short-chain dehydrogenase( EC:1.1.1.100 )  tRNA-Arg-ACG |
| 1336 | AGHY01001029.1:1..1336 | Protocatechuate 32C4-dioxygenase beta chain (EC 1.13.11.3) |
| 1334 | AGHY01001953.1:1..1334 |  |
| 1334 | AGHY01001308.1:1..1334 | Glycyl-tRNA synthetase alpha chain (EC 6.1.1.14)  hypothetical protein |
| 1334 | AGHY01000512.1:1..1334 | FIG019278: hypothetical protein |
| 1333 | AGHY01001644.1:1..1333 |  |
| 1333 | AGHY01000540.1:1..1333 | Putative permease often clustered with de novo purine synthesis |
| 1329 | AGHY01001873.1:1..1329 | Oxidoreductase |
| 1329 | AGHY01000892.1:1..1329 | Superfamily II DNA/RNA helicases2C SNF2 family |
| 1329 | AGHY01000307.1:1..1329 | FIG01111872: hypothetical protein  Putative translation initiation inhibitor2C yjgF family |
| 1328 | AGHY01001046.1:1..1328 | Transcriptional regulator2C HxlR family  hypothetical protein  hypothetical protein |
| 1326 | AGHY01002658.1:1..1326 | Isopropylmalate/homocitrate/citramalate synthases  hypothetical protein  hypothetical protein |
| 1325 | AGHY01002323.1:1..1325 | Nucleoside-diphosphate-sugar epimerases  hypothetical protein |
| 1322 | AGHY01002320.1:1..1322 | Permease of the drug/metabolite transporter (DMT) superfamily  RarD protein2C chloamphenicol sensitive |
| 1321 | AGHY01001728.1:1..1321 | Glutaredoxin-like protein |
| 1321 | AGHY01000436.1:1..1321 | ice nucleation protein  ice nucleation protein |
| 1318 | AGHY01002250.1:1..1318 |  |
| 1318 | AGHY01000370.1:1..1318 | 3-hydroxyisobutirate dehydrogenase |
| 1316 | AGHY01001172.1:1..1316 | hypothetical protein |
| 1315 | AGHY01002153.1:1..1315 | 23S rRNA (guanine-N-2-) -methyltransferase rlmL EC 2.1.1.-)  LigA |
| 1314 | AGHY01000736.1:1..1314 | Flagellar biosynthesis protein FliC |
| 1313 | AGHY01000047.1:1..1313 | hypothetical protein |
| 1312 | AGHY01001661.1:1..1312 | hypothetical protein |
| 1312 | AGHY01000459.1:1..1312 |  |
| 1311 | AGHY01002661.1:1..1311 | Glutathione reductase (EC 1.8.1.7) |
| 1310 | AGHY01000872.1:1..1310 | Phosphate-specific outer membrane porin OprP ; Pyrophosphate-specific outer membrane porin OprO |
| 1306 | AGHY01000674.1:1..1306 | hypothetical protein |
| 1305 | AGHY01000802.1:1..1305 |  |
| 1303 | AGHY01000648.1:1..1303 | tRNA-Phe-GAA |
| 1303 | AGHY01000466.1:1..1303 | Tetratricopeptide TPR\_2 repeat protein |
| 1301 | AGHY01001069.1:1..1301 | amino acid transporter |
| 1299 | AGHY01000808.1:1..1299 |  |
| 1298 | AGHY01000469.1:1..1298 | Phage major capsid protein |
| 1297 | AGHY01001409.1:1..1297 | Chorismate mutase I (EC 5.4.99.5) / Prephenate dehydratase (EC 4.2.1.51)  Chorismate mutase I (EC 5.4.99.5) / Prephenate dehydratase (EC 4.2.1.51) |
| 1296 | AGHY01000562.1:1..1296 | hypothetical protein |
| 1294 | AGHY01002732.1:1..1294 | ATP-dependent DNA ligase (EC 6.5.1.1) LigC  ATP-dependent DNA ligase (EC 6.5.1.1) LigC |
| 1294 | AGHY01001052.1:1..1294 | proteinase  proteinase |
| 1293 | AGHY01000532.1:1..1293 |  |
| 1291 | AGHY01001912.1:1..1291 | transcriptional regulator2C LysR family |
| 1291 | AGHY01001792.1:1..1291 | FIG01111551: hypothetical protein |
| 1290 | AGHY01002328.1:1..1290 | Predicted sucrose-specific TonB-dependent receptor |
| 1288 | AGHY01001849.1:1..1288 | Oxidoreductase |
| 1288 | AGHY01000920.1:1..1288 | FIG01209769: hypothetical protein |
| 1284 | AGHY01001507.1:1..1284 | Signal transduction histidine kinase CheA (EC 2.7.3.-) |
| 1282 | AGHY01001136.1:1..1282 |  |
| 1281 | AGHY01001054.1:1..1281 | Manganese superoxide dismutase (EC 1.15.1.1)  Manganese superoxide dismutase (EC 1.15.1.1)  hypothetical protein |
| 1280 | AGHY01002082.1:1..1280 | Ubiquinone/menaquinone biosynthesis methyltransferase UbiE (EC 2.1.1.-) |
| 1280 | AGHY01001618.1:1..1280 | Preprotein translocase subunit YajC (TC 3.A.5.1.1)  tRNA-guanine transglycosylase (EC 2.4.2.29) |
| 1278 | AGHY01001656.1:1..1278 |  |
| 1278 | AGHY01000181.1:1..1278 |  |
| 1277 | AGHY01001158.1:1..1277 | Endonuclease |
| 1276 | AGHY01001688.1:1..1276 | Putrescine transport ATP-binding protein PotG (TC 3.A.1.11.2) |
| 1276 | AGHY01001485.1:1..1276 |  |
| 1276 | AGHY01000272.1:1..1276 | D-mannose isomerase (EC 5.3.1.7) |
| 1275 | AGHY01001991.1:1..1275 | L-alanine-DL-glutamate epimerase  probable exported protein STY2149 |
| 1274 | AGHY01000558.1:1..1274 | Acyl-CoA thioesterase II (EC 3.1.2.-)  hypothetical protein |
| 1273 | AGHY01002444.1:1..1273 |  |
| 1273 | AGHY01000711.1:1..1273 |  |
| 1273 | AGHY01000003.1:1..1273 |  |
| 1272 | AGHY01001361.1:1..1272 | Transcriptional regulator2C AraC family |
| 1272 | AGHY01000126.1:1..1272 | hypothetical protein |
| 1271 | AGHY01001731.1:1..1271 | hypothetical protein |
| 1269 | AGHY01001355.1:1..1269 |  |
| 1268 | AGHY01001016.1:1..1268 | FIG002903: a protein of unknown function perhaps involved in purine metabolism  chemotaxis protein |
| 1266 | AGHY01002268.1:1..1266 |  |
| 1266 | AGHY01001677.1:1..1266 | Sulfate adenylyltransferase subunit 2 (EC 2.7.7.4)  Sulfate adenylyltransferase subunit 2 (EC 2.7.7.4) |
| 1266 | AGHY01001631.1:1..1266 | NAD kinase (EC 2.7.1.23) |
| 1266 | AGHY01001071.1:1..1266 | ABC-type nitrate/sulfonate/bicarbonate transport system2C ATPase component |
| 1266 | AGHY01000084.1:1..1266 | ATPase2C AFG1 family |
| 1263 | AGHY01002415.1:1..1263 |  |
| 1263 | AGHY01001750.1:1..1263 | hypothetical protein |
| 1263 | AGHY01000105.1:1..1263 | Ferric siderophore transport system2C periplasmic binding protein TonB  MotA/TolQ/ExbB proton channel family protein |
| 1262 | AGHY01001391.1:1..1262 | cytochrome P450 hydroxylase |
| 1260 | AGHY01001658.1:1..1260 | Epoxyqueuosine (oQ) reductase QueG |
| 1258 | AGHY01001494.1:1..1258 | L-lactate dehydrogenase (EC 1.1.2.3)  hypothetical protein |
| 1256 | AGHY01000825.1:1..1256 | FIG01210356: hypothetical protein  FIG136845: Rhodanese-related sulfurtransferase |
| 1255 | AGHY01001847.1:1..1255 | hypothetical protein |
| 1255 | AGHY01000875.1:1..1255 | ABC transporter ATP-binding protein |
| 1253 | AGHY01001188.1:1..1253 | hypothetical protein |
| 1252 | AGHY01001232.1:1..1252 | Ferrichrome-iron receptor |
| 1251 | AGHY01001724.1:1..1251 | Sensory subunit of low CO2-induced protein complex2C putative  hypothetical protein |
| 1250 | AGHY01000922.1:1..1250 |  |
| 1250 | AGHY01000019.1:1..1250 |  |
| 1247 | AGHY01001104.1:1..1247 | methyl-accepting chemotaxis protein |
| 1246 | AGHY01000565.1:1..1246 | Protein involved in catabolism of external DNA  hypothetical protein |
| 1246 | AGHY01000205.1:1..1246 | hypothetical protein  hypothetical protein |
| 1245 | AGHY01000789.1:1..1245 | hypothetical protein  hypothetical protein |
| 1243 | AGHY01002299.1:1..1243 | Phosphoserine aminotransferase (EC 2.6.1.52) |
| 1243 | AGHY01000167.1:1..1243 |  |
| 1240 | AGHY01001208.1:1..1240 | Beta-lactamase-like  hypothetical protein |
| 1240 | AGHY01000206.1:1..1240 |  |
| 1236 | AGHY01000514.1:1..1236 | hypothetical protein |
| 1233 | AGHY01000832.1:1..1233 |  |
| 1233 | AGHY01000396.1:1..1233 | Flagellar biosynthesis protein FliP  Flagellar biosynthesis protein FliQ |
| 1232 | AGHY01002237.1:1..1232 |  |
| 1232 | AGHY01001126.1:1..1232 | hypothetical protein |
| 1231 | AGHY01002006.1:1..1231 | UDP-glucose 4-epimerase (EC 5.1.3.2) |
| 1231 | AGHY01001311.1:1..1231 | FIG01210241: hypothetical protein  hypothetical protein |
| 1231 | AGHY01000151.1:1..1231 | Aspartate-semialdehyde dehydrogenase (EC 1.2.1.11) |
| 1230 | AGHY01001190.1:1..1230 | Soluble lytic murein transglycosylase precursor (EC 3.2.1.-)  Soluble lytic murein transglycosylase precursor (EC 3.2.1.-) |
| 1230 | AGHY01000654.1:1..1230 |  |
| 1230 | AGHY01000528.1:1..1230 | Kynurenine 3-monooxygenase (EC 1.14.13.9)  Kynurenine 3-monooxygenase (EC 1.14.13.9) |
| 1228 | AGHY01001580.1:1..1228 | TonB-dependent receptor  ferric enterobactin receptor |
| 1228 | AGHY01001011.1:1..1228 | Acetoacetyl-CoA reductase (EC 1.1.1.36)  PhbF |
| 1227 | AGHY01000949.1:1..1227 | Magnesium and cobalt transport protein CorA |
| 1227 | AGHY01000447.1:1..1227 |  |
| 1226 | AGHY01001573.1:1..1226 | Leucine-responsive regulatory protein2C regulator for leucine (or lrp) regulon and high-affinity branched-chain amino acid transport system |
| 1226 | AGHY01000995.1:1..1226 | Argininosuccinate synthase (EC 6.3.4.5)  Argininosuccinate synthase (EC 6.3.4.5)  Argininosuccinate synthase (EC 6.3.4.5) |
| 1226 | AGHY01000264.1:1..1226 | Cobalt-zinc-cadmium resistance protein CzcD  Threonine dehydrogenase and related Zn-dependent dehydrogenases |
| 1224 | AGHY01001903.1:1..1224 | Sulfate and thiosulfate binding protein CysP |
| 1223 | AGHY01000242.1:1..1223 | hypothetical protein  outer membrane protein |
| 1221 | AGHY01000202.1:1..1221 | Rod shape-determining protein RodA |
| 1219 | AGHY01000611.1:1..1219 | RND efflux system2C outer membrane lipoprotein CmeC  RND efflux system2C outer membrane lipoprotein CmeC |
| 1218 | AGHY01002664.1:1..1218 | DNA recombination protein RmuC |
| 1218 | AGHY01001716.1:1..1218 | hypothetical protein  hypothetical protein |
| 1217 | AGHY01001389.1:1..1217 | GGDEF family protein |
| 1216 | AGHY01000457.1:1..1216 | Vibrioferrin receptor PvuA |
| 1216 | AGHY01000430.1:1..1216 | RND efflux system2C membrane fusion protein CmeA |
| 1213 | AGHY01001938.1:1..1213 | Transcriptional regulator2C AraC family  Transcriptional regulator2C AraC family |
| 1213 | AGHY01000908.1:1..1213 | Xylanase  Xylanase |
| 1211 | AGHY01001992.1:1..1211 | Transcription accessory protein (S1 RNA-binding domain) |
| 1203 | AGHY01000973.1:1..1203 | Homocysteine S-methyltransferase (EC 2.1.1.10)  hypothetical protein |
| 1202 | AGHY01000394.1:1..1202 | Methylisocitrate lyase (EC 4.1.3.30) |
| 1202 | AGHY01000315.1:1..1202 | Biopolymer transport protein ExbD/TolR  Pyridoxine 5'-phosphate synthase (EC 2.6.99.2) |
| 1199 | AGHY01000619.1:1..1199 | Chromosomal replication initiator protein DnaA |
| 1197 | AGHY01000743.1:1..1197 |  |
| 1197 | AGHY01000537.1:1..1197 | D-alanyl-D-alanine carboxypeptidase (EC 3.4.16.4) |
| 1195 | AGHY01001300.1:1..1195 | Spermidine synthase (EC 2.5.1.16) |
| 1195 | AGHY01001285.1:1..1195 | D-xylose proton-symporter XylE  D-xylose proton-symporter XylE |
| 1193 | AGHY01000509.1:1..1193 | serine peptidase  serine peptidase |
| 1189 | AGHY01002115.1:1..1189 | ferric enterobactin receptor  ferric enterobactin receptor |
| 1189 | AGHY01001718.1:1..1189 | Phospholipase A1 precursor (EC 3.1.1.322C EC 3.1.1.4); Outer membrane phospholipase A |
| 1188 | AGHY01001961.1:1..1188 | hypothetical protein |
| 1188 | AGHY01001572.1:1..1188 | Zinc transporter ZupT |
| 1187 | AGHY01001567.1:1..1187 | hypothetical protein  hypothetical protein  tRNA-Leu-CAG |
| 1184 | AGHY01002495.1:1..1184 | FIG01209899: hypothetical protein  FIG01209899: hypothetical protein  hypothetical protein |
| 1184 | AGHY01001685.1:1..1184 | Queuosine Biosynthesis QueE Radical SAM  TPR repeat containing exported protein; Putative periplasmic protein contains a protein prenylyltransferase domain |
| 1184 | AGHY01001274.1:1..1184 | 16S rRNA processing protein RimM  SSU ribosomal protein S16p  tRNA (Guanine37-N1) -methyltransferase (EC 2.1.1.31) |
| 1181 | AGHY01001570.1:1..1181 | FIG01211564: hypothetical protein  hypothetical protein |
| 1179 | AGHY01001566.1:1..1179 | S-adenosyl-L-methionine dependent methyltransferase2C similar to cyclopropane-fatty-acyl-phospholipid synthase |
| 1179 | AGHY01000947.1:1..1179 |  |
| 1179 | AGHY01000184.1:1..1179 | FIG01209962: hypothetical protein  Methyl-accepting chemotaxis protein I (serine chemoreceptor protein) |
| 1178 | AGHY01000907.1:1..1178 | hypothetical protein  hypothetical protein |
| 1177 | AGHY01002429.1:1..1177 |  |
| 1177 | AGHY01001568.1:1..1177 | Alpha-ketoglutarate-dependent taurine dioxygenase (EC 1.14.11.17) |
| 1176 | AGHY01000247.1:1..1176 | Teichoic acid export ATP-binding protein TagH (EC 3.6.3.40)  hypothetical protein |
| 1176 | AGHY01000165.1:1..1176 | hypothetical protein |
| 1174 | AGHY01000576.1:1..1174 | integral membrane protein |
| 1173 | AGHY01001394.1:1..1173 |  |
| 1172 | AGHY01001859.1:1..1172 |  |
| 1170 | AGHY01000647.1:1..1170 | Chloride channel protein  Chloride channel protein  hypothetical protein |
| 1169 | AGHY01001950.1:1..1169 | Radical SAM domain protein |
| 1168 | AGHY01002085.1:1..1168 | Rrf2 family transcriptional regulator2C group III |
| 1167 | AGHY01000486.1:1..1167 | FIG01113143: hypothetical protein  hypothetical protein |
| 1165 | AGHY01002423.1:1..1165 | SAM-dependent methyltransferases  hypothetical protein |
| 1163 | AGHY01002457.1:1..1163 | 5-methyltetrahydropteroyltriglutamate--homocysteine methyltransferase (EC 2.1.1.14) |
| 1163 | AGHY01001200.1:1..1163 | Na( ) H( ) antiporter subunit A; Na( ) H( ) antiporter subunit B |
| 1163 | AGHY01000421.1:1..1163 | Sensor histidine kinase  hypothetical protein |
| 1162 | AGHY01001737.1:1..1162 | oxidoreductase |
| 1162 | AGHY01001696.1:1..1162 | N-acetylmuramoyl-L-alanine amidase (EC 3.5.1.28) |
| 1162 | AGHY01001305.1:1..1162 | hypothetical protein  hypothetical protein |
| 1162 | AGHY01000434.1:1..1162 | ATP-dependent DNA helicase RecQ |
| 1160 | AGHY01000653.1:1..1160 | Arginyl-tRNA synthetase (EC 6.1.1.19)  Arginyl-tRNA synthetase (EC 6.1.1.19) |
| 1158 | AGHY01002060.1:1..1158 | Positive regulator of CheA protein activity (CheW)  hypothetical protein |
| 1157 | AGHY01001398.1:1..1157 |  |
| 1154 | AGHY01001134.1:1..1154 | Tetracycline-efflux transporter |
| 1152 | AGHY01002272.1:1..1152 | RND multidrug efflux transporter; Acriflavin resistance protein  RND multidrug efflux transporter; Acriflavin resistance protein |
| 1152 | AGHY01002142.1:1..1152 | Inner membrane component of tripartite multidrug resistance system |
| 1152 | AGHY01001211.1:1..1152 | Aminopeptidase |
| 1152 | AGHY01001192.1:1..1152 | Xanthan biosynthesis glycosyltransferase GumD |
| 1149 | AGHY01002620.1:1..1149 | Predicted sucrose-specific TonB-dependent receptor |
| 1148 | AGHY01001330.1:1..1148 | N-acetyl-L2CL-diaminopimelate deacetylase (EC 3.5.1.47)  hypothetical protein |
| 1145 | AGHY01002280.1:1..1145 | hypothetical protein |
| 1145 | AGHY01001872.1:1..1145 | FIG065221: Holliday junction DNA helicase  FIG065221: Holliday junction DNA helicase |
| 1145 | AGHY01001862.1:1..1145 | FIG01210277: hypothetical protein  Hypothetical protein YaeR with similarity to glyoxylase family |
| 1145 | AGHY01000389.1:1..1145 |  |
| 1142 | AGHY01000051.1:1..1142 | hypothetical protein |
| 1141 | AGHY01000577.1:1..1141 |  |
| 1138 | AGHY01001536.1:1..1138 | Porphobilinogen synthase (EC 4.2.1.24) |
| 1135 | AGHY01002583.1:1..1135 | NAD(P)H oxidoreductase YRKL (EC 1.6.99.-) @ Putative NADPH-quinone reductase (modulator of drug activity B) @ Flavodoxin 2 |
| 1135 | AGHY01001540.1:1..1135 | Cytoplasmic axial filament protein CafA and Ribonuclease G (EC 3.1.4.-) |
| 1134 | AGHY01001406.1:1..1134 | Membrane protease family protein BA0301 |
| 1134 | AGHY01000682.1:1..1134 | Histidinol-phosphatase (EC 3.1.3.15) / Imidazoleglycerol-phosphate dehydratase (EC 4.2.1.19) |
| 1133 | AGHY01001671.1:1..1133 | Glycosyl transferases group 1:TPR repeat |
| 1132 | AGHY01001164.1:1..1132 |  |
| 1132 | AGHY01000555.1:1..1132 |  |
| 1130 | AGHY01002228.1:1..1130 | Sulfate adenylyltransferase subunit 1 (EC 2.7.7.4) / Adenylylsulfate kinase (EC 2.7.1.25) |
| 1130 | AGHY01001585.1:1..1130 |  |
| 1127 | AGHY01001751.1:1..1127 | chemotaxis protein  chemotaxis protein |
| 1127 | AGHY01000834.1:1..1127 | Permease of the drug/metabolite transporter (DMT) superfamily  peptidyl-prolyl cis-trans isomerase  peptidyl-prolyl cis-trans isomerase |
| 1124 | AGHY01002567.1:1..1124 | Lipopolysaccharide ABC transporter2C ATP-binding protein LptB |
| 1123 | AGHY01000228.1:1..1123 |  |
| 1122 | AGHY01001171.1:1..1122 |  |
| 1121 | AGHY01001726.1:1..1121 | RND efflux system2C inner membrane transporter CmeB |
| 1121 | AGHY01000478.1:1..1121 | hypothetical protein |
| 1121 | AGHY01000476.1:1..1121 | hypothetical protein  hypothetical protein |
| 1120 | AGHY01002139.1:1..1120 | hypothetical protein |
| 1120 | AGHY01001395.1:1..1120 | LigA |
| 1120 | AGHY01000986.1:1..1120 |  |
| 1120 | AGHY01000694.1:1..1120 |  |
| 1120 | AGHY01000190.1:1..1120 | Fibronectin type III domain protein |
| 1118 | AGHY01002169.1:1..1118 | ATPase |
| 1117 | AGHY01000862.1:1..1117 | FIG01209882: hypothetical protein  FIG01210288: hypothetical protein |
| 1116 | AGHY01001520.1:1..1116 |  |
| 1111 | AGHY01002095.1:1..1111 | Acyl-CoA thioester hydrolase  FIG01210044: hypothetical protein  hypothetical protein |
| 1107 | AGHY01001196.1:1..1107 | Flagellar motor rotation protein MotA |
| 1104 | AGHY01001786.1:1..1104 |  |
| 1103 | AGHY01001345.1:1..1103 | FIG000605: protein co-occurring with transport systems (COG1739)  FIG000605: protein co-occurring with transport systems (COG1739) |
| 1102 | AGHY01000391.1:1..1102 | transcriptional regulator2C LacI family |
| 1101 | AGHY01001733.1:1..1101 | 23S rRNA (Uracil-5-) -methyltransferase RumA (EC 2.1.1.-) |
| 1100 | AGHY01002051.1:1..1100 | Colicin V production protein |
| 1100 | AGHY01000662.1:1..1100 |  |
| 1100 | AGHY01000111.1:1..1100 |  |
| 1099 | AGHY01001746.1:1..1099 | hypothetical protein |
| 1097 | AGHY01001505.1:1..1097 |  |
| 1097 | AGHY01001147.1:1..1097 |  |
| 1097 | AGHY01000734.1:1..1097 |  |
| 1096 | AGHY01000580.1:1..1096 |  |
| 1095 | AGHY01001629.1:1..1095 | cationic amino acid transporter |
| 1095 | AGHY01001426.1:1..1095 | FIG01210495: hypothetical protein  RNA polymerase sigma-70 factor |
| 1093 | AGHY01002319.1:1..1093 | Glycerophosphoryl diester phosphodiesterase (EC 3.1.4.46) |
| 1093 | AGHY01002156.1:1..1093 |  |
| 1092 | AGHY01002515.1:1..1092 |  |
| 1092 | AGHY01001879.1:1..1092 | putative membrane protein |
| 1092 | AGHY01001173.1:1..1092 | hypothetical protein |
| 1091 | AGHY01001762.1:1..1091 | hypothetical protein |
| 1091 | AGHY01001719.1:1..1091 |  |
| 1091 | AGHY01000928.1:1..1091 | anti-sigma F factor antagonist  hypothetical protein |
| 1091 | AGHY01000622.1:1..1091 | Excinuclease ABC subunit A  thioesterase superfamily |
| 1090 | AGHY01002545.1:1..1090 | Lipoprotein releasing system ATP-binding protein LolD |
| 1088 | AGHY01000955.1:1..1088 |  |
| 1086 | AGHY01002474.1:1..1086 |  |
| 1086 | AGHY01001753.1:1..1086 | Acetolactate synthase large subunit (EC 2.2.1.6) |
| 1084 | AGHY01001918.1:1..1084 | aldose 1-epimerase |
| 1082 | AGHY01002113.1:1..1082 | hypothetical protein |
| 1082 | AGHY01001131.1:1..1082 |  |
| 1082 | AGHY01000934.1:1..1082 | UDP-N-acetylmuramoylalanyl-D-glutamyl-22C6-diaminopimelate--D-alanyl-D-alanine ligase (EC 6.3.2.10) |
| 1081 | AGHY01001218.1:1..1081 |  |
| 1081 | AGHY01000800.1:1..1081 |  |
| 1081 | AGHY01000002.1:1..1081 |  |
| 1076 | AGHY01001614.1:1..1076 | hypothetical protein |
| 1076 | AGHY01000118.1:1..1076 | Alpha-N-arabinofuranosidase 2 (EC 3.2.1.55) |
| 1075 | AGHY01001358.1:1..1075 | hypothetical protein |
| 1075 | AGHY01000481.1:1..1075 | Phosphoadenylyl-sulfate reductase [thioredoxin] (EC 1.8.4.8) |
| 1074 | AGHY01000946.1:1..1074 | hypothetical protein |
| 1073 | AGHY01002508.1:1..1073 | P-hydroxybenzoate hydroxylase (EC 1.14.13.2) |
| 1072 | AGHY01000952.1:1..1072 | TonB-dependent receptor |
| 1071 | AGHY01002688.1:1..1071 | FIG007317: hypothetical protein |
| 1071 | AGHY01001012.1:1..1071 | hypothetical protein |
| 1069 | AGHY01001301.1:1..1069 | Transcriptional regulator2C LysR family |
| 1068 | AGHY01001870.1:1..1068 | Protein of unknown function Smg  Rossmann fold nucleotide-binding protein Smf possibly involved in DNA uptake |
| 1068 | AGHY01000453.1:1..1068 | major facilitator superfamily MFS\_1 |
| 1067 | AGHY01002571.1:1..1067 | 5-Enolpyruvylshikimate-3-phosphate synthase (EC 2.5.1.19)  hypothetical protein |
| 1067 | AGHY01001539.1:1..1067 | putative transcriptional regulator2C ArsR family |
| 1067 | AGHY01000702.1:1..1067 | hypothetical protein |
| 1066 | AGHY01002674.1:1..1066 | hypothetical protein |
| 1066 | AGHY01001758.1:1..1066 | ABC transporter permease  hypothetical protein |
| 1066 | AGHY01000688.1:1..1066 | 3-isopropylmalate dehydratase large subunit (EC 4.2.1.33) |
| 1066 | AGHY01000411.1:1..1066 |  |
| 1066 | AGHY01000292.1:1..1066 | hypothetical protein  hypothetical protein |
| 1065 | AGHY01002102.1:1..1065 | tRNA pseudouridine synthase C (EC 4.2.1.70) |
| 1065 | AGHY01002078.1:1..1065 | Methionine ABC transporter ATP-binding protein |
| 1063 | AGHY01002278.1:1..1063 | hypothetical protein  transcriptional regulator2C AraC family |
| 1063 | AGHY01001087.1:1..1063 |  |
| 1062 | AGHY01001246.1:1..1062 | Cell division protein FtsL |
| 1061 | AGHY01002211.1:1..1061 | Oxidoreductase |
| 1060 | AGHY01001920.1:1..1060 | ABC transporter ATP-binding protein |
| 1058 | AGHY01002460.1:1..1058 |  |
| 1058 | AGHY01001491.1:1..1058 | NG2CNG-dimethylarginine dimethylaminohydrolase 1 (EC 3.5.3.18) |
| 1056 | AGHY01000060.1:1..1056 |  |
| 1055 | AGHY01001031.1:1..1055 | Carbonic anhydrase (EC 4.2.1.1) |
| 1055 | AGHY01000864.1:1..1055 |  |
| 1054 | AGHY01002598.1:1..1054 | PnuC protein |
| 1054 | AGHY01001115.1:1..1054 | Beta-xylosidase (EC 3.2.1.37) |
| 1053 | AGHY01001554.1:1..1053 |  |
| 1053 | AGHY01000271.1:1..1053 |  |
| 1052 | AGHY01001789.1:1..1052 | DNA mismatch repair protein MutL |
| 1050 | AGHY01002408.1:1..1050 | Octaprenyl-diphosphate synthase (EC 2.5.1.-) / Dimethylallyltransferase (EC 2.5.1.1) / Geranyltranstransferase (farnesyldiphosphate synthase) (EC 2.5.1.10) / Geranylgeranyl pyrophosphate synthetase (EC 2.5.1.29) |
| 1050 | AGHY01000317.1:1..1050 | FIG01111643: hypothetical protein  hypothetical protein |
| 1047 | AGHY01002905.1:1..1047 | FIG004453: protein YceG like |
| 1046 | AGHY01001337.1:1..1046 |  |
| 1046 | AGHY01000831.1:1..1046 | Putative protein-S-isoprenylcysteine methyltransferase |
| 1045 | AGHY01001178.1:1..1045 | FIG01212863: hypothetical protein |
| 1043 | AGHY01001037.1:1..1043 | Transcriptional regulator |
| 1042 | AGHY01002093.1:1..1042 | hypothetical protein  hypothetical protein |
| 1042 | AGHY01000245.1:1..1042 | hypothetical protein  hypothetical protein  iron transporter |
| 1041 | AGHY01001565.1:1..1041 | Pirin |
| 1040 | AGHY01001730.1:1..1040 | D-beta-hydroxybutyrate dehydrogenase (EC 1.1.1.30) |
| 1040 | AGHY01000113.1:1..1040 | hypothetical protein  phosphoadenosine phosphosulfate reductase |
| 1039 | AGHY01001834.1:1..1039 | Adenosine (5')-pentaphospho-(5'')-adenosine pyrophosphohydrolase (EC 3.6.1.-)  tRNA-Gln-CTG  tRNA-Met-CAT |
| 1038 | AGHY01000376.1:1..1038 | Tryptophan synthase beta chain (EC 4.2.1.20) |
| 1035 | AGHY01001988.1:1..1035 | FIG000233: metal-dependent hydrolase  hypothetical protein |
| 1035 | AGHY01000033.1:1..1035 | Lipase  RND efflux system2C outer membrane lipoprotein CmeC |
| 1032 | AGHY01001651.1:1..1032 |  |
| 1032 | AGHY01000759.1:1..1032 | Superfamily II DNA/RNA helicases2C SNF2 family |
| 1031 | AGHY01001215.1:1..1031 | [Protein-PII] uridylyltransferase (EC 2.7.7.59) |
| 1031 | AGHY01000506.1:1..1031 | Arylesterase precursor (EC 3.1.1.2) |
| 1030 | AGHY01002368.1:1..1030 | Na -driven multidrug efflux pump |
| 1030 | AGHY01001245.1:1..1030 | Putative oligoketide cyclase/dehydratase or lipid transport protein YfjG  tmRNA-binding protein SmpB |
| 1029 | AGHY01001934.1:1..1029 | Putative sulfite oxidase subunit YedY |
| 1029 | AGHY01001287.1:1..1029 | Ferredoxin--NADP( ) reductase (EC 1.18.1.2) |
| 1028 | AGHY01000877.1:1..1028 | hypothetical protein |
| 1027 | AGHY01001143.1:1..1027 | hypothetical protein |
| 1027 | AGHY01000897.1:1..1027 | hypothetical protein |
| 1026 | AGHY01001787.1:1..1026 | Endoglucanase (EC 3.2.1.4)  probable cellulase |
| 1025 | AGHY01001548.1:1..1025 |  |
| 1025 | AGHY01000959.1:1..1025 |  |
| 1025 | AGHY01000322.1:1..1025 |  |
| 1024 | AGHY01002283.1:1..1024 | Osmosensitive K channel histidine kinase KdpD (EC 2.7.3.-) |
| 1023 | AGHY01000207.1:1..1023 |  |
| 1022 | AGHY01002700.1:1..1022 | Dehydrogenases with different specificities (related to short-chain alcohol dehydrogenases) |
| 1019 | AGHY01001241.1:1..1019 |  |
| 1018 | AGHY01001905.1:1..1018 | 3-deoxy-D-manno-octulosonic-acid kinase |
| 1017 | AGHY01002149.1:1..1017 | Xanthan biosynthesis pyruvyltransferase GumL |
| 1016 | AGHY01003143.1:1..1016 |  |
| 1016 | AGHY01001694.1:1..1016 | Unsaturated fatty acid biosythesis repressor FabR2C TetR family |
| 1014 | AGHY01002111.1:1..1014 | Excinuclease ABC subunit A paralog of unknown function |
| 1014 | AGHY01000930.1:1..1014 | Cyclopropane-fatty-acyl-phospholipid synthase-like protein2C clusters with FIG005069  Cyclopropane-fatty-acyl-phospholipid synthase-like protein2C clusters with FIG005069 |
| 1012 | AGHY01002160.1:1..1012 |  |
| 1010 | AGHY01001837.1:1..1010 |  |
| 1010 | AGHY01000990.1:1..1010 |  |
| 1008 | AGHY01002335.1:1..1008 | ATP-dependent helicase HrpB |
| 1007 | AGHY01000472.1:1..1007 | Putrescine utilization regulator |
| 1006 | AGHY01002522.1:1..1006 | Cysteinyl-tRNA synthetase (EC 6.1.1.16) |
| 1006 | AGHY01000211.1:1..1006 | tRNA-Ala-CGC  virulence regulator |
| 1005 | AGHY01001902.1:1..1005 |  |
| 1004 | AGHY01002038.1:1..1004 | Hydroxyacylglutathione hydrolase (EC 3.1.2.6) |
| 1004 | AGHY01001985.1:1..1004 |  |
| 1004 | AGHY01000963.1:1..1004 |  |
| 1002 | AGHY01001858.1:1..1002 | Dipeptidyl carboxypeptidase  Ribosomal-protein-S5p-alanine acetyltransferase |
| 1002 | AGHY01000343.1:1..1002 | DNA-binding response regulator2C LuxR family |
| 1000 | AGHY01001911.1:1..1000 | Tryptophan synthase beta chain (EC 4.2.1.20) |
