# Supplementary material for: Draft Genome Sequences of Xanthomonas sacchari and Two Banana-Associated Xanthomonads Reveal Insights into the Xanthomonas Group 1 Clade
Source: Genes (Basel). 2011 Dec 2;2(4):1050–65. doi: 10.3390/genes2041050 (PMC3927605; doi:10.3390/genes2041050)
Supplement: Supplementary File 1 — ZIP-Document (ZIP, 7075 KB) [file genes-02-01050-s001.zip › genes-11371-supplementary/NCPPB1131_genes_comparison.pdf]

NCPPB1131 predicted genes

Gene	Location	NCPPB1131	NCPPB1132	NCPPB4393
fig|487527.6.peg.2266 hypothetical protein	AGHY01000542.1:8..121	0.96	0.00	0.00
fig|487527.6.peg.2784 Threonine dehydrogenase and related Zn-dependent dehydrogenases	AGHY01000264.1:8..259	0.99	0.03	0.35
fig|487527.6.peg.1031 hypothetical protein	AGHY01001470.1:1004..606	1.00	0.00	0.00
fig|487527.6.peg.1085 hypothetical protein	AGHY01001422.1:545..390	1.00	0.00	0.00
fig|487527.6.peg.1086 Esterase/lipase	AGHY01001422.1:486..1571	1.00	0.00	0.00
fig|487527.6.peg.1132 hypothetical protein	AGHY01001378.1:2316..2071	1.00	0.00	0.00
fig|487527.6.peg.1195 hypothetical protein	AGHY01001320.1:1503..1682	1.00	0.00	0.00
fig|487527.6.peg.1196 hypothetical protein	AGHY01001319.1:406..218	1.00	0.00	0.00
fig|487527.6.peg.1207 hypothetical protein	AGHY01001311.1:490..855	1.00	0.00	0.00
fig|487527.6.peg.1315 hypothetical protein	AGHY01001234.1:43..240	1.00	0.00	0.00
fig|487527.6.peg.1322 FIG053235: Diacylglucosamine hydrolase like	AGHY01001230.1:103..765	1.00	0.00	0.00
fig|487527.6.peg.1342 hypothetical protein	AGHY01001219.1:600..448	1.00	0.00	0.00
fig|487527.6.peg.1389 hypothetical protein	AGHY01001179.1:232..107	1.00	0.00	0.00
fig|487527.6.peg.1422 hypothetical protein	AGHY01001149.1:476..898	1.00	0.00	0.00
fig|487527.6.peg.1450 FIG01210434: hypothetical protein	AGHY01001129.1:1515..2027	1.00	0.00	0.00
fig|487527.6.peg.1480 hypothetical protein	AGHY01001108.1:139..26	1.00	0.00	0.00
fig|487527.6.peg.1481 hypothetical protein	AGHY01001108.1:138..323	1.00	0.00	0.00
fig|487527.6.peg.1589 Transcriptional regulator2C AraC family	AGHY01001017.1:1085..75	1.00	0.00	0.00
fig|487527.6.peg.1590 hypothetical protein	AGHY01001017.1:1427..1293	1.00	0.00	0.00
fig|487527.6.peg.1606 Manganese transport protein MntH	AGHY01001004.1:832..455	1.00	0.00	0.00
fig|487527.6.peg.1612 hypothetical protein	AGHY01001000.1:2942..2775	1.00	0.00	0.00
fig|487527.6.peg.1704 hypothetical protein	AGHY01000923.1:319..155	1.00	0.00	0.00
fig|487527.6.peg.1764 hypothetical protein	AGHY01000886.1:3658..3482	1.00	0.00	0.00
fig|487527.6.peg.1811 hypothetical protein	AGHY01000856.1:279..413	1.00	0.00	0.00
fig|487527.6.peg.1830 hypothetical protein	AGHY01000845.1:1520..1654	1.00	0.00	0.00
fig|487527.6.peg.1831 hypothetical protein	AGHY01000845.1:4111..3953	1.00	0.00	0.00
fig|487527.6.peg.1837 hypothetical protein	AGHY01000843.1:1017..859	1.00	0.00	0.00
fig|487527.6.peg.1870 hypothetical protein	AGHY01000821.1:167..6	1.00	0.00	0.00
fig|487527.6.peg.1874 transcriptional regulator	AGHY01000821.1:3742..3074	1.00	0.00	0.00
fig|487527.6.peg.1875 putative%3B ORF located using Glimmer/Genemark	AGHY01000821.1:3885..4151	1.00	0.00	0.00
fig|487527.6.peg.1891 hypothetical protein	AGHY01000810.1:278..123	1.00	0.00	0.00
fig|487527.6.peg.1913 hypothetical protein	AGHY01000789.1:631..20	1.00	0.00	0.00
fig|487527.6.peg.1922 hypothetical protein	AGHY01000783.1:1401..1514	1.00	0.00	0.00
fig|487527.6.peg.1923 BatD	AGHY01000783.1:2057..1773	1.00	0.00	0.00
fig|487527.6.peg.1926 hypothetical protein	AGHY01000782.1:1718..2470	1.00	0.00	0.00
fig|487527.6.peg.1948 hypothetical protein	AGHY01000771.1:2997..2065	1.00	0.00	0.00
fig|487527.6.peg.1949 hypothetical protein	AGHY01000771.1:3344..2997	1.00	0.00	0.00
fig|487527.6.peg.1951 hypothetical protein	AGHY01000770.1:53..238	1.00	0.00	0.00
fig|487527.6.peg.1979 hypothetical protein	AGHY01000749.1:704..537	1.00	0.00	0.00
fig|487527.6.peg.1989 hypothetical protein	AGHY01000738.1:1296..1508	1.00	0.00	0.00
fig|487527.6.peg.1991 hypothetical protein	AGHY01000738.1:2830..2375	1.00	0.00	0.00
fig|487527.6.peg.2027 Glycosyl transferase2C family 2	AGHY01000705.1:337..1200	1.00	0.00	0.00
fig|487527.6.peg.2028 Glycosyl transferase2C family 2	AGHY01000705.1:1164..2219	1.00	0.00	0.00

NCPPB1131 predicted genes

fig|487527.6.peg.2068 hypothetical protein	AGHY01000686.1:1236..1571	✓	1.00	✗	0.00	✗	0.00
fig|487527.6.peg.2112 putative MarR family transcriptional regulator	AGHY01000658.1:746..1213	✓	1.00	✗	0.00	✗	0.00
fig|487527.6.peg.2136 Transcriptional regulator2C MarR family	AGHY01000640.1:985..1398	✓	1.00	✗	0.00	✗	0.00
fig|487527.6.peg.2139 hypothetical protein	AGHY01000638.1:487..371	✓	1.00	✗	0.00	✗	0.00
fig|487527.6.peg.2186 hypothetical protein	AGHY01000596.1:2325..1708	✓	1.00	✗	0.00	✗	0.00
fig|487527.6.peg.2187 lipase2C class 3	AGHY01000596.1:3416..2394	✓	1.00	✗	0.00	✗	0.00
fig|487527.6.peg.2306 Methyl-accepting chemotaxis protein I (serine chemoreceptor protein)	AGHY01000523.1:1639..119	✓	1.00	✗	0.00	✗	0.00
fig|487527.6.peg.2307 hypothetical protein	AGHY01000523.1:1869..2702	✓	1.00	✗	0.00	✗	0.00
fig|487527.6.peg.2308 hypothetical protein	AGHY01000523.1:2699..2935	✓	1.00	✗	0.00	✗	0.00
fig|487527.6.peg.2373 hypothetical protein	AGHY01000482.1:2736..2849	✓	1.00	✗	0.00	✗	0.00
fig|487527.6.peg.2374 hypothetical protein	AGHY01000482.1:2860..3294	✓	1.00	✗	0.00	✗	0.00
fig|487527.6.peg.2380 hypothetical protein	AGHY01000480.1:1655..1774	✓	1.00	✗	0.00	✗	0.00
fig|487527.6.peg.2387 hypothetical protein	AGHY01000477.1:1926..1798	✓	1.00	✗	0.00	✗	0.00
fig|487527.6.peg.2390 hypothetical protein	AGHY01000476.1:146..6	✓	1.00	✗	0.00	✗	0.00
fig|487527.6.peg.2421 extracellular protease(EC:3.4.21.-);Ontology_term=KEGG_ENZYME:3.4.21.-	AGHY01000456.1:115..1941	✓	1.00	✗	0.00	✗	0.00
fig|487527.6.peg.2445 hypothetical protein	AGHY01000444.1:2287..1541	✓	1.00	✗	0.00	✗	0.00
fig|487527.6.peg.2475 hypothetical protein	AGHY01000425.1:33..170	✓	1.00	✗	0.00	✗	0.00
fig|487527.6.peg.2484 hypothetical protein	AGHY01000420.1:605..207	✓	1.00	✗	0.00	✗	0.00
fig|487527.6.peg.2611 hypothetical protein	AGHY01000365.1:1825..1622	✓	1.00	✗	0.00	✗	0.00
fig|487527.6.peg.263 hypothetical protein	AGHY01002495.1:921..1178	✓	1.00	✗	0.00	✗	0.00
fig|487527.6.peg.2641 hypothetical protein	AGHY01000351.1:1484..507	✓	1.00	✗	0.00	✗	0.00
fig|487527.6.peg.2711 Putative translation initiation inhibitor2C yjgF family	AGHY01000307.1:1314..643	✓	1.00	✗	0.00	✗	0.00
fig|487527.6.peg.2733 Membrane-fusion protein	AGHY01000288.1:1929..2972	✓	1.00	✗	0.00	✗	0.00
fig|487527.6.peg.2734 colicin V secretion ABC transporter ATP-binding protein	AGHY01000288.1:3026..3793	✓	1.00	✗	0.00	✗	0.00
fig|487527.6.peg.2735 colicin V secretion ABC transporter ATP-binding protein	AGHY01000288.1:3847..5139	✓	1.00	✗	0.00	✗	0.00
fig|487527.6.peg.2736 hypothetical protein	AGHY01000288.1:5226..5342	✓	1.00	✗	0.00	✗	0.00
fig|487527.6.peg.2739 hypothetical protein	AGHY01000287.1:2136..2252	✓	1.00	✗	0.00	✗	0.00
fig|487527.6.peg.2740 hypothetical protein	AGHY01000287.1:2401..2249	✓	1.00	✗	0.00	✗	0.00
fig|487527.6.peg.2774 hypothetical protein	AGHY01000270.1:931..677	✓	1.00	✗	0.00	✗	0.00
fig|487527.6.peg.2815 Ribosomal protein S4 and related proteins	AGHY01000250.1:557..24	✓	1.00	✗	0.00	✗	0.00
fig|487527.6.peg.2816 Transcriptional regulator2C LysR family	AGHY01000250.1:676..1632	✓	1.00	✗	0.00	✗	0.00
fig|487527.6.peg.2817 Histone acetyltransferase HPA2 and related acetyltransferases	AGHY01000250.1:2176..1673	✓	1.00	✗	0.00	✗	0.00
fig|487527.6.peg.2830 hypothetical protein	AGHY01000245.1:376..723	✓	1.00	✗	0.00	✗	0.00
fig|487527.6.peg.2831 hypothetical protein	AGHY01000245.1:1032..880	✓	1.00	✗	0.00	✗	0.00
fig|487527.6.peg.2836 hypothetical protein	AGHY01000239.1:814..1854	✓	1.00	✗	0.00	✗	0.00
fig|487527.6.peg.2848 hypothetical protein	AGHY01000232.1:3072..3230	✓	1.00	✗	0.00	✗	0.00
fig|487527.6.peg.2866 hypothetical protein	AGHY01000219.1:3..185	✓	1.00	✗	0.00	✗	0.00
fig|487527.6.peg.2893 Transcriptional regulator2C LysR family	AGHY01000210.1:1599..1381	✓	1.00	✗	0.00	✗	0.00
fig|487527.6.peg.2924 COG0845: Membrane-fusion protein	AGHY01000193.1:2107..1772	✓	1.00	✗	0.00	✗	0.00
fig|487527.6.peg.2976 hypothetical protein	AGHY01000166.1:4257..4370	✓	1.00	✗	0.00	✗	0.00
fig|487527.6.peg.2977 transcriptional regulator2C MarR family	AGHY01000166.1:4665..4399	✓	1.00	✗	0.00	✗	0.00
fig|487527.6.peg.3048 Oxidoreductase2C short chain dehydrogenase/reductase family	AGHY01000128.1:3011..4216	✓	1.00	✗	0.00	✗	0.00
fig|487527.6.peg.3075 hypothetical protein	AGHY01000119.1:2141..1986	✓	1.00	✗	0.00	✗	0.00
fig|487527.6.peg.3084 FIG01212420: hypothetical protein	AGHY01000116.1:2363..1359	✓	1.00	✗	0.00	✗	0.00

fig|487527.6.peg.3085 FIG01212420: hypothetical protein	AGHY01000116.1:3752..2721	✓	1.00	✗	0.00	✗ 0.00
fig|487527.6.peg.3100 Phosphoglycerate mutase	AGHY01000108.1:1420..2040	✓	1.00	✗	0.00	✗ 0.00
fig|487527.6.peg.3101 (Acyl-carrier protein) phosphodiesterase(EC:3.1.4.14);Ontology_term=KEGG_ENZYME:3.1.4.14	AGHY01000108.1:2621..2121	✓	1.00	✗	0.00	✗ 0.00
fig|487527.6.peg.3102 hypothetical protein	AGHY01000106.1:1513..1067	✓	1.00	✗	0.00	✗ 0.00
fig|487527.6.peg.3157 hypothetical protein	AGHY01000093.1:1587..1706	✓	1.00	✗	0.00	✗ 0.00
fig|487527.6.peg.3158 Alpha-12C2-mannosidase	AGHY01000092.1:38..265	✓	1.00	✗	0.00	✗ 0.00
fig|487527.6.peg.3187 hypothetical protein	AGHY01000079.1:1818..1946	✓	1.00	✗	0.00	✗ 0.00
fig|487527.6.peg.3228 hypothetical protein	AGHY01000063.1:647..967	✓	1.00	✗	0.00	✗ 0.00
fig|487527.6.peg.3261 hypothetical protein	AGHY01000048.1:1306..1470	✓	1.00	✗	0.00	✗ 0.00
fig|487527.6.peg.3284 hypothetical protein	AGHY01000042.1:1450..1325	✓	1.00	✗	0.00	✗ 0.00
fig|487527.6.peg.3285 hypothetical protein	AGHY01000042.1:1627..1508	✓	1.00	✗	0.00	✗ 0.00
fig|487527.6.peg.3289 hypothetical protein	AGHY01000042.1:4487..4203	✓	1.00	✗	0.00	✗ 0.00
fig|487527.6.peg.3290 hypothetical protein	AGHY01000042.1:4894..4493	✓	1.00	✗	0.00	✗ 0.00
fig|487527.6.peg.3291 hypothetical protein	AGHY01000042.1:6229..6011	✓	1.00	✗	0.00	✗ 0.00
fig|487527.6.peg.3292 hypothetical protein	AGHY01000042.1:6814..6485	✓	1.00	✗	0.00	✗ 0.00
fig|487527.6.peg.3294 hypothetical protein	AGHY01000042.1:8189..8061	✓	1.00	✗	0.00	✗ 0.00
fig|487527.6.peg.3296 hypothetical protein	AGHY01000042.1:9158..8955	✓	1.00	✗	0.00	✗ 0.00
fig|487527.6.peg.33 hypothetical protein	AGHY01003061.1:44..166	✓	1.00	✗	0.00	✗ 0.00
fig|487527.6.peg.3334 hypothetical protein	AGHY01000029.1:2986..1814	✓	1.00	✗	0.00	✗ 0.00
fig|487527.6.peg.3338 O-antigen export system permease protein RfbD	AGHY01000028.1:715..32	✓	1.00	✗	0.00	✗ 0.00
fig|487527.6.peg.3339 FIG01212275: hypothetical protein	AGHY01000028.1:1242..922	✓	1.00	✗	0.00	✗ 0.00
fig|487527.6.peg.3340 putative%3B ORF located using Glimmer/Genemark	AGHY01000028.1:3181..1586	✓	1.00	✗	0.00	✗ 0.00
fig|487527.6.peg.3374 hypothetical protein	AGHY01000013.1:1955..273	✓	1.00	✗	0.00	✗ 0.00
fig|487527.6.peg.35 hypothetical protein	AGHY01003061.1:2380..2496	✓	1.00	✗	0.00	✗ 0.00
fig|487527.6.peg.354 hypothetical protein	AGHY01002323.1:149..6	✓	1.00	✗	0.00	✗ 0.00
fig|487527.6.peg.430 luciferase-like	AGHY01002215.1:684..520	✓	1.00	✗	0.00	✗ 0.00
fig|487527.6.peg.448 hypothetical protein	AGHY01002176.1:35..337	✓	1.00	✗	0.00	✗ 0.00
fig|487527.6.peg.452 hypothetical protein	AGHY01002170.1:388..609	✓	1.00	✗	0.00	✗ 0.00
fig|487527.6.peg.520 hypothetical protein	AGHY01002083.1:1851..2024	✓	1.00	✗	0.00	✗ 0.00
fig|487527.6.peg.521 DUF378 domain-containing protein	AGHY01002083.1:2248..2009	✓	1.00	✗	0.00	✗ 0.00
fig|487527.6.peg.530 hypothetical protein	AGHY01002069.1:592..449	✓	1.00	✗	0.00	✗ 0.00
fig|487527.6.peg.621 hypothetical protein	AGHY01001947.1:759..908	✓	1.00	✗	0.00	✗ 0.00
fig|487527.6.peg.796 methyl-accepting chemotaxis protein	AGHY01001732.1:595..152	✓	1.00	✗	0.00	✗ 0.00
fig|487527.6.peg.803 hypothetical protein	AGHY01001724.1:200..69	✓	1.00	✗	0.00	✗ 0.00
fig|487527.6.peg.870 hypothetical protein	AGHY01001648.1:563..856	✓	1.00	✗	0.00	✗ 0.00
fig|487527.6.peg.961 hypothetical protein	AGHY01001549.1:1507..1629	✓	1.00	✗	0.00	✗ 0.00
fig|487527.6.peg.994 hypothetical protein	AGHY01001511.1:877..1791	✓	1.00	✗	0.00	✗ 0.00
fig|487527.6.peg.995 hypothetical protein	AGHY01001510.1:417..127	✓	1.00	✗	0.00	✗ 0.00
fig|487527.6.peg.411 hypothetical protein	AGHY01002243.1:327..647	✓	1.00	✗	0.00	✗ 0.04
fig|487527.6.peg.896 hypothetical protein	AGHY01001605.1:167..376	✓	1.00	✗	0.00	✗ 0.11
fig|487527.6.peg.3286 hypothetical protein	AGHY01000042.1:2735..2610	✓	1.00	✗	0.00	✗ 0.15
fig|487527.6.peg.2974 hypothetical protein	AGHY01000166.1:2982..2671	✓	1.00	✗	0.00	✗ 0.16
fig|487527.6.peg.3288 Zonular occludens toxin	AGHY01000042.1:4203..3121	✓	1.00	✗	0.00	✗ 0.17
fig|487527.6.peg.3219 Type IV fimbrial biogenesis protein PilX	AGHY01000070.1:12814..12605	✓	1.00	✗	0.00	✗ 0.21

fig|487527.6.peg.1251 FIG01213967: hypothetical protein	AGHY01001281.1:3676..4467	✓ 1.00	✗ 0.00	✗ 0.25
fig|487527.6.peg.3215 Type IV pilus biogenesis protein PilE	AGHY01000070.1:8781..8416	✓ 1.00	✗ 0.00	✗ 0.28
fig|487527.6.peg.3218 Type IV fimbrial biogenesis protein PilY1	AGHY01000070.1:12543..11863	✓ 1.00	✗ 0.00	✗ 0.29
fig|487527.6.peg.2857 hypothetical protein	AGHY01000226.1:23..157	✓ 1.00	✗ 0.00	✗ 0.30
fig|487527.6.peg.2881 hypothetical acetyltransferase	AGHY01000214.1:873..1355	✓ 1.00	✗ 0.00	✗ 0.33
fig|487527.6.peg.2802 hypothetical protein	AGHY01000255.1:1465..1220	✓ 1.00	✗ 0.00	! 0.35
fig|487527.6.peg.3217 Type IV fimbrial biogenesis protein PilY1	AGHY01000070.1:11866..10124	✓ 1.00	✗ 0.00	! 0.36
fig|487527.6.peg.3255 hypothetical protein	AGHY01000052.1:6576..6376	✓ 1.00	✗ 0.00	! 0.41
fig|487527.6.peg.1761 hypothetical protein	AGHY01000886.1:2100..1933	✓ 1.00	✗ 0.00	! 0.43
fig|487527.6.peg.2975 monooxygenase2C FAD-binding	AGHY01000166.1:4253..2979	✓ 1.00	✗ 0.00	! 0.45
fig|487527.6.peg.3214 type 4 fimbrial biogenesis protein	AGHY01000070.1:7710..8201	✓ 1.00	✗ 0.00	! 0.46
fig|487527.6.peg.3293 Putative phage replication protein RstA	AGHY01000042.1:7763..6876	✓ 1.00	✗ 0.00	! 0.46
fig|487527.6.peg.1305 hypothetical protein	AGHY01001244.1:4..216	✓ 1.00	✗ 0.00	! 0.48
fig|487527.6.peg.3287 hypothetical protein	AGHY01000042.1:2993..2748	✓ 1.00	✗ 0.00	! 0.49
fig|487527.6.peg.690 hypothetical protein	AGHY01001854.1:297..440	✓ 1.00	✗ 0.00	! 0.53
fig|487527.6.peg.1938 hypothetical protein	AGHY01000774.1:2246..1026	✓ 1.00	✗ 0.00	! 0.57
fig|487527.6.peg.3254 prolyl oligopeptidase family protein	AGHY01000052.1:4118..6034	✓ 1.00	✗ 0.00	! 0.57
fig|487527.6.peg.3381 Chemotaxis protein methyltransferase CheR (EC 2.1.1.80);Ontology_term=KEGG_ENZYME:2.1.1.8	AGHY01000011.1:732..490	✓ 1.00	✗ 0.00	! 0.58
fig|487527.6.peg.1349 hypothetical protein	AGHY01001214.1:164..24	✓ 1.00	✗ 0.00	! 0.58
fig|487527.6.peg.1247 Major pilus subunit of type IV secretion complex2C VirB2	AGHY01001281.1:280..675	✓ 1.00	✗ 0.00	! 0.64
fig|487527.6.peg.1428 RND efflux system2C membrane fusion protein CmeA	AGHY01001145.1:1988..2203	✓ 1.00	✗ 0.00	! 0.65
fig|487527.6.peg.3315 hypothetical protein	AGHY01000034.1:3906..3778	✓ 1.00	✗ 0.00	✓ 0.67
fig|487527.6.peg.159 oxidoreductase	AGHY01002728.1:1258..800	✓ 1.00	✗ 0.00	✓ 0.68
fig|487527.6.peg.3216 Type IV fimbrial biogenesis protein PilY1	AGHY01000070.1:9524..8838	✓ 1.00	✗ 0.00	✓ 0.69
fig|487527.6.peg.3295 hypothetical protein	AGHY01000042.1:8398..8246	✓ 1.00	✗ 0.00	✓ 0.76
fig|487527.6.peg.3341 Phytoene desaturase (EC 1.14.99.-);Ontology_term=KEGG_ENZYME:1.14.99.-	AGHY01000028.1:3716..3201	✓ 1.00	✗ 0.00	✓ 0.77
fig|487527.6.peg.810 Beta-ketoadipate enol-lactone hydrolase (EC 3.1.1.24);Ontology_term=KEGG_ENZYME:3.1.1.24	AGHY01001712.1:735..595	✓ 1.00	✗ 0.00	✓ 0.82
fig|487527.6.peg.2087 hypothetical protein	AGHY01000672.1:1475..1305	✓ 1.00	✗ 0.00	✓ 0.82
fig|487527.6.peg.925 hypothetical protein	AGHY01001578.1:187..699	✓ 1.00	✗ 0.00	✓ 0.83
fig|487527.6.peg.1814 Transcriptional regulator2C TetR family	AGHY01000854.1:109..348	✓ 1.00	✗ 0.00	✓ 0.83
fig|487527.6.peg.2973 GCN5-related N-acetyltransferase	AGHY01000166.1:2146..2601	✓ 1.00	✗ 0.00	✓ 0.86
fig|487527.6.peg.1440 3-oxoacyl-[acyl-carrier protein] reductase (EC 1.1.1.100);Ontology_term=KEGG_ENZYME:1.1.1.100	AGHY01001137.1:1185..442	✓ 1.00	✗ 0.00	✓ 0.87
fig|487527.6.peg.3382 Chemotaxis protein methyltransferase CheR (EC 2.1.1.80);Ontology_term=KEGG_ENZYME:2.1.1.8	AGHY01000011.1:2663..705	✓ 1.00	✗ 0.00	✓ 0.88
fig|487527.6.peg.1429 RND efflux system2C inner membrane transporter CmeB	AGHY01001145.1:2213..2419	✓ 1.00	✗ 0.00	✓ 0.88
fig|487527.6.peg.2757 Transcriptional regulatory protein RtcR	AGHY01000284.1:1794..3386	✓ 1.00	✗ 0.00	✓ 0.91
fig|487527.6.peg.3316 hypothetical protein	AGHY01000034.1:3931..4269	✓ 1.00	✗ 0.00	✓ 0.97
fig|487527.6.peg.1658 Signal peptidase I (EC 3.4.21.89);Ontology_term=KEGG_ENZYME:3.4.21.89	AGHY01000956.1:989..1558	✓ 1.00	✗ 0.00	✓ 0.98
fig|487527.6.peg.2756 Putative ribonucleoprotein related-protein	AGHY01000284.1:1591..44	✓ 1.00	✗ 0.00	✓ 0.99
fig|487527.6.peg.2801 HlyD family secretion protein	AGHY01000255.1:9..1196	✓ 1.00	✗ 0.00	✓ 1.00
fig|487527.6.peg.2338 Succinate dehydrogenase hydrophobic membrane anchor protein	AGHY01000499.1:3267..2935	✓ 1.00	✗ 0.00	✓ 1.00
fig|487527.6.peg.1116 Uncharacterized glutathione S-transferase-like protein	AGHY01001390.1:828..1460	✓ 1.00	✗ 0.00	✗ 0.00
fig|487527.6.peg.1752 Superfamily II DNA/RNA helicases2C SNF2 family	AGHY01000892.1:1329..589	✓ 1.00	✗ 0.01	✗ 0.00
fig|487527.6.peg.1095 lipopolysaccharide biosynthesis protein	AGHY01001413.1:1776..1558	✓ 1.00	✗ 0.02	! 0.42
fig|487527.6.peg.1364 hypothetical protein	AGHY01001204.1:3569..5083	✓ 1.00	✗ 0.02	✓ 1.00

fig|487527.6.peg.2837 choline/carnitine/betaine transporter	AGHY01000239.1:1839..2138	1.00	0.02	0.00
fig|487527.6.peg.1478 alginate biosynthesis protein	AGHY01001111.1:3545..3081	1.00	0.03	0.64
fig|487527.6.peg.326 hypothetical protein	AGHY01002382.1:707..522	1.00	0.03	0.02
fig|487527.6.peg.1877 Gluconate transporter family protein	AGHY01000819.1:266..1003	1.00	0.04	0.04
fig|487527.6.peg.2984 wall associated protein	AGHY01000161.1:2965..869	1.00	0.05	0.60
fig|487527.6.peg.2358 FIG01211701: hypothetical protein	AGHY01000491.1:41..1573	1.00	0.05	0.10
fig|487527.6.peg.218 hypothetical protein	AGHY01002586.1:30..179	1.00	0.05	0.00
fig|487527.6.peg.1353 hypothetical protein	AGHY01001209.1:371..760	1.00	0.12	0.00
fig|487527.6.peg.2581 hypothetical protein	AGHY01000377.1:3208..2837	1.00	0.19	0.00
fig|487527.6.peg.1961 Glucose-1-phosphate thymidylyltransferase (EC 2.7.7.24);Ontology_term=KEGG_ENZYME:2.7.7.24	AGHY01000765.1:1345..590	1.00	0.19	0.00
fig|487527.6.peg.2679 sensor kinase	AGHY01000329.1:2353..3072	1.00	0.19	0.00
fig|487527.6.peg.3183 hypothetical protein	AGHY01000081.1:881..1222	1.00	0.23	0.00
fig|487527.6.peg.1146 hypothetical protein	AGHY01001363.1:874..996	1.00	0.24	0.00
fig|487527.6.peg.3099 hypothetical protein	AGHY01000108.1:1365..1249	1.00	0.33	0.00
fig|487527.6.peg.1664 GCN5-related N-acetyltransferase	AGHY01000950.1:2204..2725	1.00	0.34	0.00
fig|487527.6.peg.2291 hypothetical protein	AGHY01000529.1:59..238	1.00	0.42	0.00
fig|487527.6.peg.2073 TonB-dependent receptor	AGHY01000680.1:280..125	1.00	0.44	0.00
fig|487527.6.peg.222 hypothetical protein	AGHY01002578.1:54..206	1.00	0.47	0.05
fig|487527.6.peg.2909 hypothetical protein	AGHY01000200.1:1441..1572	1.00	0.55	0.00
fig|487527.6.peg.3071 hypothetical protein	AGHY01000119.1:164..36	1.00	0.57	0.00
fig|487527.6.peg.1329 putative%3B ORF located using Glimmer/Genemark	AGHY01001224.1:935..654	1.00	0.59	0.00
fig|487527.6.peg.1062 hypothetical protein	AGHY01001441.1:3891..3703	1.00	0.59	0.00
fig|487527.6.peg.568 Putative outer membrane or secreted lipoprotein	AGHY01002027.1:1572..463	1.00	0.65	0.03
fig|487527.6.peg.3347 hypothetical protein	AGHY01000027.1:2524..2697	1.00	0.67	0.05
fig|487527.6.peg.1960 dTDP-4-dehydrorhamnose 32C5-epimerase (EC 5.1.3.13);Ontology_term=KEGG_ENZYME:5.1.3.13	AGHY01000765.1:587..36	1.00	0.68	0.00
fig|487527.6.peg.1034 Transcriptional regulator2C AraC family	AGHY01001469.1:61..585	1.00	0.71	0.00
fig|487527.6.peg.2212 conserved hypothetical protein	AGHY01000585.1:1124..1531	1.00	0.78	0.00
fig|487527.6.peg.1536 hypothetical protein	AGHY01001064.1:634..386	1.00	0.82	0.00
fig|487527.6.peg.714 hypothetical protein	AGHY01001829.1:467..607	1.00	0.84	0.00
fig|487527.6.peg.3060 hypothetical protein	AGHY01000124.1:2983..3156	1.00	0.89	0.00
fig|487527.6.peg.2211 Transcriptional regulator2C AraC family	AGHY01000585.1:483..1067	1.00	0.91	0.00
fig|487527.6.peg.2811 hypothetical protein	AGHY01000252.1:2537..2743	1.00	0.99	0.00
fig|487527.6.peg.2104 TonB-dependent receptor	AGHY01000663.1:5910..4954	1.00	1.00	0.00
fig|487527.6.peg.2813 Di-/tripeptide transporter	AGHY01000252.1:2979..3368	1.00	1.00	0.00
fig|487527.6.peg.1033 hypothetical protein	AGHY01001469.1:210..16	1.00	1.00	0.00
fig|487527.6.peg.1211 hypothetical protein	AGHY01001308.1:510..250	1.00	1.00	0.00
fig|487527.6.peg.1898 hypothetical protein	AGHY01000805.1:45..191	1.00	1.00	0.00
fig|487527.6.peg.2102 TonB-dependent receptor	AGHY01000663.1:4199..3534	1.00	1.00	0.00
fig|487527.6.peg.2103 TonB-dependent receptor	AGHY01000663.1:4915..4196	1.00	1.00	0.00
fig|487527.6.peg.2812 Di-/tripeptide transporter	AGHY01000252.1:2713..2979	1.00	1.00	0.00
fig|487527.6.peg.3239 hypothetical protein	AGHY01000059.1:1416..1093	1.00	1.00	0.00
