# Supplementary material for: Draft Genome Sequences of Xanthomonas sacchari and Two Banana-Associated Xanthomonads Reveal Insights into the Xanthomonas Group 1 Clade
Source: Genes (Basel). 2011 Dec 2;2(4):1050–65. doi: 10.3390/genes2041050 (PMC3927605; doi:10.3390/genes2041050)
Supplement: Supplementary File 1 — ZIP-Document (ZIP, 7075 KB) [file genes-02-01050-s001.zip › genes-11371-supplementary/NCPPB1132-sequences-not-in-X_albilineans.html]

Regions of the Xanthomonas species NCPPB1132 genome that show no detectable nucleotide sequence similarity with X. albilineans


### Regions of the *Xanthomonas* species NCPPB1132 genome that show no detectable nucleotide sequence similarity with *X. albilineans*

No hits against these regions were found when performing *blastn* searches against the genome of *X. albilineans* GPE PC73 (RefSeq:NC\_013722), using the NCPPB1132 draft genome assembly as the query.

| Length of region (nucleotides) | GenBank accession and coordinates of region | Predicted genes in this region (using RAST: Aziz et al. 2008 BMC Genomics 9:75) |
| --- | --- | --- |
| 28737 | AGHZ01000109.1:1..28737 | 3'-to-5' oligoribonuclease (orn)  ATP/GTP-binding protein  Bifunctional protein: zinc-containing alcohol dehydrogenase; quinone oxidoreductase ( NADPH:quinone reductase) (EC 1.1.1.-); Similar to arginate lyase  FIG00452947: hypothetical protein  Glycine betaine-binding protein  Glyoxalase family protein  LysR family transcriptional regulator STM3121  Manganese transport protein MntH  Mn-dependent transcriptional regulator MntR  Phosphoenolpyruvate synthase (EC 2.7.9.2)  Potassium efflux system KefA protein / Small-conductance mechanosensitive channel  Rrf2 family transcriptional regulator2C group III  SAM-dependent methyltransferase BA1462 (UbiE paralog)  ThiJ/PfpI  Thioredoxin reductase (EC 1.8.1.9)  TonB-dependent siderophore receptor  Transcriptional regulator2C AraC family  Transcriptional regulator2C AraC family  Transcriptional regulator2C LysR family  conserved hypothetical protein  hypothetical protein  hypothetical protein  hypothetical protein  putative secreted protein  sensor kinase  tRNA-specific adenosine-34 deaminase (EC 3.5.4.-)  transcriptional regulator lysR family |
| 27598 | AGHZ01000095.1:1..27598 | ATPase provides energy for both assembly of type IV secretion complex and secretion of T-DNA complex (VirB11)  ATPase provides energy for both assembly of type IV secretion complex and secretion of T-DNA complex (VirB4)  Alpha-glucosidase (EC 3.2.1.20)  Bores hole in peptidoglycan layer allowing type IV secretion complex assembly to occur (VirB1)  FIG01211666: hypothetical protein  FIG01213967: hypothetical protein  Forms the bulk of type IV secretion complex that spans outer membrane and periplasm (VirB9)  Inner membrane protein forms channel for type IV secretion of T-DNA complex (VirB10)  Inner membrane protein forms channel for type IV secretion of T-DNA complex (VirB3)  Inner membrane protein forms channel for type IV secretion of T-DNA complex (VirB8)  Integral inner membrane protein of type IV secretion complex (VirB6)  Major pilus subunit of type IV secretion complex (VirB2)  Maltodextrin glucosidase (EC 3.2.1.20)  Predicted maltose transporter MalT  Six-hairpin glycosidase-like protein  TonB-dependent receptor  Type IV secretion system protein VirD4  hypothetical protein |
| 26052 | AGHZ01000865.1:1..26052 | 33 kDa chaperonin (Heat shock protein 33) (HSP33)  ABC transporter2C ATP-binding protein  Acyl-CoA dehydrogenase2C short-chain specific (EC 1.3.99.2)  AsmA family membrane protein  Beta-lactamase (EC 3.5.2.6)  COG1272: Predicted membrane protein hemolysin III homolog  Choline dehydrogenase (EC 1.1.99.1)  FIG01113126: hypothetical protein  FIG01209855: hypothetical protein  FIG01211173: hypothetical protein  FIG01211374: hypothetical protein  Glycosyltransferase  Inosine-5'-monophosphate dehydrogenase (EC 1.1.1.205)  Membrane proteins related to metalloendopeptidases  Monofunctional biosynthetic peptidoglycan transglycosylase (EC 2.4.2.-)  Peptide chain release factor 3  TonB-dependent receptor  Transcriptional regulator2C GntR family  Transcriptional regulator2C TetR family  alpha/beta hydrolase fold  hypothetical protein  hypothetical protein |
| 25257 | AGHZ01000147.1:1..25257 | 3-ketoacyl-CoA thiolase (EC 2.3.1.16) @ Acetyl-CoA acetyltransferase (EC 2.3.1.9)  Agmatine deiminase (EC 3.5.3.12)  Cytidylate kinase (EC 2.7.4.14)  Enoyl-CoA hydratase (EC 4.2.1.17) / 32C2-trans-enoyl-CoA isomerase (EC 5.3.3.8) / 3-hydroxyacyl-CoA dehydrogenase (EC 1.1.1.35)  FIG01210851: hypothetical protein  Heat shock (predicted periplasmic) protein YciM2C precursor  Integration host factor beta subunit  LSU ribosomal protein L36p  N-carbamoylputrescine amidase (3.5.1.53)  Nucleoside diphosphate kinase (EC 2.7.4.6)  Pheromone shutdown protein  Predicted transcriptional regulator for fatty acid degradation FadQ2C TetR family  Ribosomal RNA large subunit methyltransferase N (EC 2.1.1.-)  SSU ribosomal protein S1p  Transport protein  Transport protein  Type IV pilus biogenesis protein PilF  UDP-N-acetylglucosamine 42C6-dehydratase (EC 4.2.1.-)  UTP--glucose-1-phosphate uridylyltransferase (EC 2.7.7.9)  hypothetical protein  hypothetical protein  hypothetical protein  lipopolysaccharide core biosynthesis protein |
| 24118 | AGHZ01000013.1:1..24118 | Cystathionine beta-synthase (EC 4.2.1.22)  Cystathionine gamma-lyase (EC 4.4.1.1)  FIG01211631: hypothetical protein  Glycosyl transferase2C group 2 family protein  Glycosyl transferase2C group 2 family protein  O-antigen export system permease protein RfbD  Phytoene desaturase (EC 1.14.99.-)  Putative transmembrane oxidoreductase protein  Teichoic acid export ATP-binding protein TagH (EC 3.6.3.40)  UptF protein  glycosyl transferase2C family 2  hypothetical protein  hypothetical protein  hypothetical protein  outer membrane protein  oxidoreductase2C short-chain dehydrogenase/reductase family  putative; ORF located using Glimmer/Genemark |
| 22300 | AGHZ01000133.1:1..22300 | ATP-dependent endonuclease family protein  Dephospho-CoA kinase (EC 2.7.1.24)  Hypothetical protein  Leader peptidase (Prepilin peptidase) (EC 3.4.23.43) / N-methyltransferase (EC 2.1.1.-)  Phospholipase C  Ribosomal protein S6 glutaminyl transferase  TonB-dependent receptor  Two-component system regulatory protein  Type IV fimbriae expression regulatory protein PilR  Type IV fimbrial assembly protein PilC  Type IV fimbrial assembly2C ATPase PilB  Type IV pilin PilA  hypothetical protein  hypothetical protein  two-component system sensor protein |
| 22168 | AGHZ01000018.1:1..22168 | Alpha-12C2-mannosidase  DNA-binding protein  FIG00901053: hypothetical protein  FIG01008102: hypothetical protein  HipA protein  RNA polymerase ECF-type sigma factor  TonB-dependent receptor  hypothetical protein  hypothetical protein  hypothetical protein  hypothetical protein  possible DNA helicase |
| 22020 | AGHZ01000178.1:1..22020 | Adenylosuccinate synthetase (EC 6.3.4.4)  GGDEF domain protein  HflC protein  HflK protein  Mrr restriction endonuclease  Putative predicted metal-dependent hydrolase  Type I restriction-modification system2C DNA-methyltransferase subunit M (EC 2.1.1.72)  Type I restriction-modification system2C restriction subunit R (EC 3.1.21.3)  Type I restriction-modification system2C specificity subunit S (EC 3.1.21.3)  hypothetical protein  hypothetical protein  hypothetical protein  hypothetical protein  protease |
| 21536 | AGHZ01000049.1:1..21536 | Alpha-L-fucosidase (EC 3.2.1.51)  Beta-galactosidase (EC 3.2.1.23)  hypothetical protein  hypothetical protein  hypothetical protein  hypothetical protein  phage-related integrase  plasmid mobilization protein |
| 21250 | AGHZ01000085.1:1..21250 | DNA repair protein RadC  Succinoglycan biosynthesis protein  endonuclease precursor  hypothetical protein  hypothetical protein |
| 20489 | AGHZ01000039.1:1..20489 | 2-dehydropantoate 2-reductase (EC 1.1.1.169)  ABC-type multidrug transport system2C ATPase component  Deoxyribonuclease TatD  FIG01209941: hypothetical protein  Flp pilus assembly protein TadB  Outer membrane protein  SAF domain  Type II/IV secretion system ATP hydrolase TadA/VirB11/CpaF2C TadA subfamily  Type II/IV secretion system protein TadC2C associated with Flp pilus assembly  Uncharacterized secreted protein  hypothetical protein  hypothetical protein  hypothetical protein  hypothetical protein  hypothetical protein  hypothetical protein  hypothetical protein  hypothetical protein  hypothetical protein  hypothetical protein  permease  phenol hydroxylase  type II and III secretion system protein |
| 20066 | AGHZ01000430.1:1..20066 | 12C4-alpha-glucan (glycogen) branching enzyme2C GH-13-type (EC 2.4.1.18)  DNA-cytosine methyltransferase (EC 2.1.1.37)  Endonuclease precursor  FIG00715517: hypothetical protein  FIG01065397: hypothetical protein  FIG01211080: hypothetical protein  Integrase  RNA polymerase sigma factor RpoD  Very-short-patch mismatch repair endonuclease (G-T specific)  hypothetical protein  hypothetical protein  putative; ORF located using Glimmer/Genemark  sensor kinase  tRNA-Met-CAT |
| 19292 | AGHZ01000119.1:1..19292 | FIG01112335: hypothetical protein  FIG01210576: hypothetical protein  FIG014574: hypothetical protein  Phage T7 exclusion protein  Phage T7 exclusion protein associated hypothetical protein  Putative deoxyribonuclease similar to YcfH2C type 4  TonB-dependent receptor  hypothetical protein  hypothetical protein  hypothetical protein  hypothetical protein  hypothetical protein  hypothetical protein  hypothetical protein  hypothetical protein |
| 19079 | AGHZ01000057.1:1..19079 | 3-oxoacid CoA transferase beta subunit( EC:2.8.3.5 )  Electron transfer flavoprotein-ubiquinone oxidoreductase (EC 1.5.5.1)  Electron transfer flavoprotein2C alpha subunit  Electron transfer flavoprotein2C beta subunit  FIG01212420: hypothetical protein  Glucose-1-phosphate thymidylyltransferase (EC 2.7.7.24)  Mannose-6-phosphate isomerase (EC 5.3.1.8) / Mannose-1-phosphate guanylyltransferase (GDP) (EC 2.7.7.22)  Phosphomannomutase (EC 5.4.2.8)  Succinyl-CoA:3-ketoacid-coenzyme A transferase subunit A (EC 2.8.3.5)  dTDP-4-dehydrorhamnose 32C5-epimerase (EC 5.1.3.13)  dTDP-4-dehydrorhamnose reductase (EC 1.1.1.133)  dTDP-glucose 42C6-dehydratase (EC 4.2.1.46)  integral membrane protein  integral membrane protein  oxidoreductase |
| 18210 | AGHZ01000034.1:1..18210 | Cell division protein FtsH (EC 3.4.24.-)  DNA-3-methyladenine glycosylase II (EC 3.2.2.21)  FIG01111551: hypothetical protein  Gluconokinase (EC 2.7.1.12)  putative exported protein  wall associated protein  wall associated protein  wall associated protein |
| 17802 | AGHZ01000176.1:1..17802 | 2-deoxy-D-gluconate 3-dehydrogenase (EC 1.1.1.125)  4-deoxy-L-threo-5-hexosulose-uronate ketol-isomerase (EC 5.3.1.17)  Pentapeptide repeat:Glyoxalase/bleomycin resistance protein/dioxygenase  Phenylalanine-4-hydroxylase (EC 1.14.16.1)  Probable lipoprotein  Probable lipoprotein  Putative OMR family iron-siderophore receptor precursor  TRAP-type C4-dicarboxylate transport system2C large permease component  TRAP-type C4-dicarboxylate transport system2C periplasmic component  TRAP-type transport system2C small permease component2C predicted N-acetylneuraminate transporter  Transcriptional regulator2C AsnC family  UPF0028 protein YchK  hypothetical protein  hypothetical protein  lipoprotein2C putative  predicted 4-deoxy-L-threo-5-hexosulose-uronate ketol-isomerase (EC 5.3.1.17)  putative; ORF located using Glimmer/Genemark  putative; ORF located using Glimmer/Genemark  transcriptional regulator2C LacI family |
| 17577 | AGHZ01000206.1:1..17577 | ABC transporter permease protein  ABC transporter2C substrate binding protein  ABC-type nitrate/sulfonate/bicarbonate transport system2C ATPase component  Aconitate hydratase (EC 4.2.1.3) @ 2-methylisocitrate dehydratase (EC 4.2.1.99)  FIG00786362: hypothetical protein  Long-chain-fatty-acid--CoA ligase (EC 6.2.1.3)  LysR family transcriptional regulator YbhD  Lysyl-tRNA synthetase (class II) (EC 6.1.1.6)  Peptide chain release factor 2; programmed frameshift-containing  RpfF protein  Sensory/regulatory protein rpfC (EC 2.7.3.-)  hypothetical protein  hypothetical protein  response regulator |
| 16338 | AGHZ01000024.1:1..16338 | DNA-directed RNA polymerase beta subunit (EC 2.7.7.6)  DNA-directed RNA polymerase beta' subunit (EC 2.7.7.6)  LSU ribosomal protein L10p (P0)  LSU ribosomal protein L11p (L12e)  LSU ribosomal protein L1p (L10Ae)  LSU ribosomal protein L7/L12 (P1/P2)  Preprotein translocase subunit SecE (TC 3.A.5.1.1)  SSU ribosomal protein S12p (S23e)  SSU ribosomal protein S7p (S5e)  Transcription antitermination protein NusG  Translation elongation factor G  tRNA-Trp-CCA |
| 16311 | AGHZ01000038.1:1..16311 | FIG01210399: hypothetical protein  Methyl-accepting chemotaxis protein I (serine chemoreceptor protein)  Methyl-accepting chemotaxis protein I (serine chemoreceptor protein)  Methyl-accepting chemotaxis protein I (serine chemoreceptor protein)  Methyl-accepting chemotaxis protein I (serine chemoreceptor protein)  Methyl-accepting chemotaxis protein I (serine chemoreceptor protein)  Positive regulator of CheA protein activity (CheW)  hypothetical protein  hypothetical protein  hypothetical protein  hypothetical protein |
| 16186 | AGHZ01000143.1:1..16186 | DNA-directed RNA polymerase alpha subunit (EC 2.7.7.6)  LSU ribosomal protein L14p (L23e)  LSU ribosomal protein L15p (L27Ae)  LSU ribosomal protein L16p (L10e)  LSU ribosomal protein L17p  LSU ribosomal protein L18p (L5e)  LSU ribosomal protein L22p (L17e)  LSU ribosomal protein L23p (L23Ae)  LSU ribosomal protein L24p (L26e)  LSU ribosomal protein L29p (L35e)  LSU ribosomal protein L2p (L8e)  LSU ribosomal protein L30p (L7e)  LSU ribosomal protein L3p (L3e)  LSU ribosomal protein L4p (L1e)  LSU ribosomal protein L5p (L11e)  LSU ribosomal protein L6p (L9e)  Periplasmic thiol:disulfide oxidoreductase DsbB2C required for DsbA reoxidation  Preprotein translocase secY subunit (TC 3.A.5.1.1)  SSU ribosomal protein S10p (S20e)  SSU ribosomal protein S11p (S14e)  SSU ribosomal protein S13p (S18e)  SSU ribosomal protein S14p (S29e)  SSU ribosomal protein S17p (S11e)  SSU ribosomal protein S19p (S15e)  SSU ribosomal protein S3p (S3e)  SSU ribosomal protein S4p (S9e)  SSU ribosomal protein S5p (S2e)  SSU ribosomal protein S8p (S15Ae)  hypothetical protein |
| 15773 | AGHZ01000009.1:1..15773 | Decarboxylase family protein  Excinuclease ABC subunit B  TonB-dependent receptor  Type IV fimbrial biogenesis protein FimT  Type IV fimbrial biogenesis protein PilV  Type IV fimbrial biogenesis protein PilW  Type IV fimbrial biogenesis protein PilX  Type IV fimbrial biogenesis protein PilY1  Type IV pilus biogenesis protein PilE  alginate biosynthesis protein  tRNA-Asn-GTT  tRNA-Val-GAC |
| 15274 | AGHZ01000282.1:1..15274 | Acetyltransferase2C GNAT family  Deoxyribodipyrimidine photolyase (EC 4.1.99.3)  Glutaryl-CoA dehydrogenase (EC 1.3.99.7)  Glutathione S-transferase2C unnamed subgroup (EC 2.5.1.18)  Outer membrane protein A precursor  Putative deoxyribonuclease YjjV  Putative oxidoreductase  Replicative DNA helicase (EC 3.6.1.-)  Wax ester synthase/acyl-CoA:diacylglycerol acyltransferase  diguanylate cyclase/phosphodiesterase (GGDEF  hypothetical protein  hypothetical protein  hypothetical protein  hypothetical protein  probable exported protein YPO1624  transcriptional regulator |
| 15175 | AGHZ01000340.1:1..15175 | FIG01211701: hypothetical protein  Imidazole glycerol phosphate synthase cyclase subunit (EC 4.1.3.-)  Periplasmic aromatic amino acid aminotransferase beta precursor (EC 2.6.1.57)  Phosphoribosyl-AMP cyclohydrolase (EC 3.5.4.19) / Phosphoribosyl-ATP pyrophosphatase (EC 3.6.1.31)  carbonyl reductase [NADPH] 1 (nadph-dependent carbonylreductase 1) (20-beta-hydroxysteroid dehydrogenase) (prostaglandin-e(2) 9-reductase)( EC:1.1.1.1842CEC:1.1.1.1892CEC:1.1.1.197 )  conserved hypothetical protein  coproporphyrinogen III oxidase2C putative  hypothetical protein  hypothetical protein  hypothetical protein  hypothetical protein  hypothetical protein  peptide synthetase  permease  sulfotransferase |
| 15086 | AGHZ01000356.1:1..15086 | ATP-dependent Clp protease ATP-binding subunit ClpX  ATP-dependent Clp protease proteolytic subunit (EC 3.4.21.92)  ATP-dependent protease La (EC 3.4.21.53) Type I  Cell division trigger factor (EC 5.2.1.8)  DNA-binding protein HU-alpha  FIG01209733: hypothetical protein  ISBma12C transposase  Isocitrate dehydrogenase [NAD] (EC 1.1.1.41)  hypothetical protein  phage-related integrase  tRNA-Arg-TCT  tRNA-His-GTG  tRNA-Leu-TAG  tRNA-Lys-CTT  tRNA-Pro-TGG  tRNA-Val-TAC |
| 14456 | AGHZ01000092.1:1..14456 | Long-chain-fatty-acid--CoA ligase (EC 6.2.1.3)  PBS lyase HEAT domain protein repeat-containing protein  RNA polymerase sigma-70 factor2C ECF subfamily  SyrP-like protein  hypothetical protein  hypothetical protein  hypothetical protein( EC:2.1.4.1 )  putative transposase  thioesterase |
| 14381 | AGHZ01000079.1:1..14381 | Di-/tripeptide transporter  FIG01111510: hypothetical protein  FIG01112671: hypothetical protein  Glucose-methanol-choline (GMC) oxidoreductase:NAD binding site  Hydroxypyruvate isomerase (EC 5.3.1.22)  Inosose isomerase (EC 5.3.99.-)  Myo-inositol 2-dehydrogenase (EC 1.1.1.18)  Putative nucleoside transporter yegT  Single-stranded DNA-binding protein  Transcriptional (co)regulator CytR  Xylose isomerase-like TIM barrel  hypothetical protein  hypothetical protein  prolyl oligopeptidase family protein  prolyl oligopeptidase family protein  putative multi-domain protein |
| 14253 | AGHZ01000338.1:1..14253 | Dipeptidyl carboxypeptidase  FIG01211221: hypothetical protein  FIG01211837: hypothetical protein  FIG01211876: hypothetical protein  FIG01212144: hypothetical protein  FIG01212265: hypothetical protein  Inner membrane protein  Ribosomal-protein-S5p-alanine acetyltransferase  Two-component system regulatory protein  VirB6 protein  hypothetical protein  hypothetical protein  hypothetical protein  hypothetical protein  two-component system sensor protein |
| 13989 | AGHZ01000088.1:1..13989 | 3-deoxy-D-manno-octulosonate 8-phosphate phosphatase (EC 3.1.3.45)  Arabinose 5-phosphate isomerase (EC 5.3.1.13)  FIG000506: Predicted P-loop-containing kinase  FIG01211114: hypothetical protein  FIG01211150: hypothetical protein  FIG01211650: hypothetical protein  HPr kinase/phosphorylase (EC 2.7.1.-) (EC 2.7.4.-)  Lipopolysaccharide ABC transporter2C ATP-binding protein LptB  LptA2C protein essential for LPS transport across the periplasm  PTS system nitrogen-specific IIA component2C PtsN  PTS system2C mannose-specific IIA component (EC 2.7.1.69)  Phosphocarrier protein2C nitrogen regulation associated  Phosphoenolpyruvate-protein phosphotransferase of PTS system (EC 2.7.3.9)  RNA polymerase sigma-54 factor RpoN  Ribosome hibernation protein YhbH  UDP-N-acetylglucosamine 1-carboxyvinyltransferase (EC 2.5.1.7)  Uncharacterized protein YrbK clustered with lipopolysaccharide transporters  YrbA protein |
| 13727 | AGHZ01000078.1:1..13727 | 4-Hydroxy-2-oxoglutarate aldolase (EC 4.1.3.16) / 2-dehydro-3-deoxyphosphogluconate aldolase (EC 4.1.2.14)  Cell division protein BolA  FIG01097475: hypothetical protein  Hypothetical protein USSDB1E  N-Acetyl-D-glucosamine ABC transport system2C permease protein 1  N-Acetyl-D-glucosamine ABC transport system2C permease protein 2  OmpA-related protein  Sugar ABC transporter2C periplasmic sugar-binding protein USSDB1B  Transcriptional regulator2C LacI family |
| 13630 | AGHZ01000033.1:1..13630 | Alpha-L-fucosidase (EC 3.2.1.51)  Chemotaxis response regulator containing a CheY-like receiver domain and a methylesterase domain  Cytoplasmic copper homeostasis protein cutC  FIG00955472: hypothetical protein  FIG01112193: hypothetical protein  FIG01210372: hypothetical protein  FIG01210820: hypothetical protein  L-asparaginase (EC 3.5.1.1)  hypothetical protein |
| 13430 | AGHZ01000031.1:1..13430 | FIG01210126: hypothetical protein  FIG01210947: hypothetical protein  Peptidase2C S41 family  Polysaccharide deacetylase  RNA:NAD 2'-phosphotransferase  hypothetical protein  hypothetical protein  hypothetical protein  hypothetical protein  putative lytic enzyme  putative; ORF located using Glimmer/Genemark |
| 13370 | AGHZ01000255.1:1..13370 | Cell division protein FtsH (EC 3.4.24.-)  GMP synthase [glutamine-hydrolyzing] (EC 6.3.5.2)  Hypothetical transmembrane protein coupled to NADH-ubiquinone oxidoreductase chain 5 homolog  Inosine-5'-monophosphate dehydrogenase (EC 1.1.1.205)  Methylenetetrahydrofolate dehydrogenase (NADP ) (EC 1.5.1.5) / Methenyltetrahydrofolate cyclohydrolase (EC 3.5.4.9)  hypothetical protein  hypothetical protein  hypothetical protein  hypothetical protein  protein of unknown function DUF1244  putative Glutathione-regulated potassium-efflux system protein KefB  putative restriction endonuclease |
| 13318 | AGHZ01000017.1:1..13318 | Cytochrome O ubiquinol oxidase subunit I (EC 1.10.3.-)  Cytochrome O ubiquinol oxidase subunit II (EC 1.10.3.-)  Cytochrome O ubiquinol oxidase subunit III (EC 1.10.3.-)  Cytochrome O ubiquinol oxidase subunit IV (EC 1.10.3.-)  FIG01112656: hypothetical protein  IS1404 transposase  ISXoo3 transposase orfA  colicin V secretion ABC transporter ATP-binding protein  peptidase M61 domain protein  peptidase M61 domain protein  tRNA-Thr-CGT |
| 12862 | AGHZ01000042.1:1..12862 | 6-phosphofructokinase (EC 2.7.1.11)  Adenylate kinase (EC 2.7.4.3)  FIG01111142: hypothetical protein  FIG01210548: hypothetical protein  FIG01210920: hypothetical protein  Inorganic pyrophosphatase (EC 3.6.1.1)  Pyrophosphate-energized proton pump (EC 3.6.1.1)  Thiamin biosynthesis protein ThiC  hypothetical protein  hypothetical protein  response regulator |
| 12686 | AGHZ01000933.1:1..12686 | ABC transporter2C membrane spanning protein  Adenosylmethionine-8-amino-7-oxononanoate aminotransferase (EC 2.6.1.62)  Gamma-glutamyl-GABA hydrolase (EC 3.5.1.94)  Putrescine ABC transporter putrescine-binding protein PotF (TC 3.A.1.11.2)  Transcriptional regulator  glutamine synthetase family protein  glutamine synthetase family protein  hypothetical protein  hypothetical protein  hypothetical protein  putative oxidoreductase |
| 12633 | AGHZ01000076.1:1..12633 | COG4615: ABC-type siderophore export system2C fused ATPase and permease components  Penicillin amidase family protein  Salicylate hydroxylase (EC 1.14.13.1) |
| 12450 | AGHZ01000190.1:1..12450 | Acetylglutamate kinase (EC 2.7.2.8)  Acetylornithine deacetylase (EC 3.5.1.16)  Argininosuccinate lyase (EC 4.3.2.1)  Argininosuccinate synthase (EC 6.3.4.5)  Cysteinyl-tRNA synthetase (EC 6.1.1.16)  FIG01209702: hypothetical protein  FIG01209975: hypothetical protein  Histone acetyltransferase HPA2 and related acetyltransferases  N-acetyl-gamma-glutamyl-phosphate reductase (EC 1.2.1.38)  N-acetylglutamate synthase (EC 2.3.1.1)  N-acetylornithine carbamoyltransferase (EC 2.1.3.9)  Sulfur acceptor protein SufE for iron-sulfur cluster assembly  hypothetical protein |
| 12354 | AGHZ01000027.1:1..12354 | Aromatic-amino-acid aminotransferase (EC 2.6.1.57)  FIG01212315: hypothetical protein  Fructose-12C6-bisphosphatase2C type I (EC 3.1.3.11)  Radical SAM domain protein  Radical SAM domain protein  TonB-dependent receptor  hypothetical protein  hypothetical protein  hypothetical protein  hypothetical protein |
| 12288 | AGHZ01000277.1:1..12288 | ABC transporter ATP binding protein  Asparagine synthetase [glutamine-hydrolyzing] (EC 6.3.5.4)  Beta-lactamase (EC 3.5.2.6)  FIG01210913: hypothetical protein  GAF domain/sensory box/EAL domain protein  N-succinyl-L2CL-diaminopimelate desuccinylase (EC 3.5.1.18)  Penicillin acylase II  diguanylate cyclase (GGDEF domain)  hypothetical protein |
| 12188 | AGHZ01000231.1:1..12188 | COG0536: GTP-binding protein Obg  Copper metallochaperone2C bacterial analog of Cox17 protein  Excinuclease ABC subunit A  LSU ribosomal protein L21p  LSU ribosomal protein L27p  Proposed peptidoglycan lipid II flippase MurJ  SSU ribosomal protein S20p  hypothetical protein  thioesterase superfamily |
| 12122 | AGHZ01000350.1:1..12122 | Chromosomal replication initiator protein DnaA  DNA gyrase subunit B (EC 5.99.1.3)  DNA polymerase III beta subunit (EC 2.7.7.7)  DNA recombination and repair protein RecF  Inner membrane protein translocase component YidC2C long form  LSU ribosomal protein L34p  Ribonuclease P protein component (EC 3.1.26.5) |
| 12029 | AGHZ01000103.1:1..12029 | Dihydrodipicolinate synthase (EC 4.2.1.52)  Outer membrane protein romA  Pca regulon regulatory protein PcaR  RpfN protein  Transcriptional regulator2C TetR family  Transporter2C MFS superfamily  hypothetical protein  hypothetical protein  hypothetical protein  hypothetical protein  putative cytochrome b561 |
| 11879 | AGHZ01000478.1:1..11879 | 4-hydroxybenzoyl-CoA thioesterase family active site  Crossover junction endodeoxyribonuclease RuvC (EC 3.1.22.4)  FIG000859: hypothetical protein  Holliday junction DNA helicase RuvA  Holliday junction DNA helicase RuvB  Kup system potassium uptake protein  MotA/TolQ/ExbB proton channel family protein  Outer membrane lipoprotein omp16 precursor  Tol biopolymer transport system2C TolR protein  TolA protein  hypothetical protein  hypothetical protein  tolB protein precursor2C periplasmic protein involved in the tonb-independent uptake of group A colicins |
| 11788 | AGHZ01000037.1:1..11788 | Lysyl endopeptidase (EC 3.4.21.50)  Quino(hemo)protein alcohol dehydrogenase2C PQQ-dependent (EC 1.1.99.8)  TonB-dependent receptor  general stress protein  hypothetical protein  hypothetical protein  hypothetical protein  hypothetical protein  hypothetical protein  hypothetical protein  hypothetical protein  hypothetical protein  lipolytic enzyme2C G-D-S-L |
| 11521 | AGHZ01000028.1:1..11521 | Alpha-glucuronidase (EC 3.2.1.139)  Beta-xylosidase (EC 3.2.1.37)  Periplasmic beta-glucosidase (EC 3.2.1.21)  sal operon transcriptional repressor  sialic acid-specific 9-O-acetylesterase |
| 11510 | AGHZ01000047.1:1..11510 | ATP-dependent DNA helicase UvrD/PcrA  Cardiolipin synthetase (EC 2.7.8.-)  FIG01210192: hypothetical protein  FIG01210827: hypothetical protein  Maltose O-acetyltransferase (EC 2.3.1.79)  PDZ domain family protein  Transcriptional regulator2C LysR family  Universal stress protein family  hypothetical protein  hypothetical protein  integral membrane protein-like protein  putative secreted protein |
| 11307 | AGHZ01000406.1:1..11307 | Dehydrogenases with different specificities (related to short-chain alcohol dehydrogenases)  Phosphohydrolase (MutT/nudix family protein)  Putative conserved integral membrane protein  Ribonucleotide reductase of class II (coenzyme B12-dependent) (EC 1.17.4.1)  Ribonucleotide reductase of class II (coenzyme B12-dependent)2C alpha subunit (EC 1.17.4.1)  diguanylate phosphodiesterase with GAF sensor  histone protein  hypothetical protein  hypothetical protein |
| 11236 | AGHZ01000014.1:1..11236 | COG0779: clustered with transcription termination protein NusA  NADH-ubiquinone oxidoreductase chain G (EC 1.6.5.3)  NADH-ubiquinone oxidoreductase chain H (EC 1.6.5.3)  NADH-ubiquinone oxidoreductase chain I (EC 1.6.5.3)  NADH-ubiquinone oxidoreductase chain J (EC 1.6.5.3)  NADH-ubiquinone oxidoreductase chain K (EC 1.6.5.3)  NADH-ubiquinone oxidoreductase chain L (EC 1.6.5.3)  NADH-ubiquinone oxidoreductase chain M (EC 1.6.5.3)  NADH-ubiquinone oxidoreductase chain N (EC 1.6.5.3)  Transcription termination protein NusA  hypothetical protein  tRNA-Met-CAT |
| 11235 | AGHZ01001536.1:1..11235 | FIG01210269: hypothetical protein  Ferredoxin2C 2Fe-2S  Fumarylacetoacetate hydrolase family protein  Large-conductance mechanosensitive channel  Phospholipase/lecithinase/hemolysin  Sulfur carrier protein ThiS  Thiazole biosynthesis protein ThiG  hypothetical protein  putative phage-related integrase  sulfur deprivation response regulator  tRNA (guanine46-N7-)-methyltransferase (EC 2.1.1.33)  tRNA-Gly-CCC |
| 11223 | AGHZ01001045.1:1..11223 | Beta-galactosidase (EC 3.2.1.23)  Maltodextrin glucosidase (EC 3.2.1.20)  TonB-dependent receptor  galactose-binding protein regulator  sialic acid-specific 9-O-acetylesterase |
| 11135 | AGHZ01000121.1:1..11135 | Acetylornithine aminotransferase (EC 2.6.1.11)  Ferrichrome-iron receptor  Iron-uptake factor PiuC  S-adenosylmethionine:tRNA ribosyltransferase-isomerase (EC 5.-.-.-)  Transcriptional regulator2C AsnC family  hypothetical protein  hypothetical protein  hypothetical protein  lipoprotein2C putative |
| 11101 | AGHZ01000524.1:1..11101 | Aminotransferase  Cytochrome c2  FIG01209798: hypothetical protein  FIG01210424: hypothetical protein  FIG01213081: hypothetical protein  FIG01214411: hypothetical protein  cytochrome C  cytochrome like B561  glycosyl transferase  lipopolysaccharide biosynthesis protein  transferase2C putative |
| 11005 | AGHZ01000320.1:1..11005 | FIG01209870: hypothetical protein  FIG01210164: hypothetical protein  FIG01210356: hypothetical protein  FIG01211750: hypothetical protein  FIG136845: Rhodanese-related sulfurtransferase  Glycerol-3-phosphate dehydrogenase [NAD(P) ] (EC 1.1.1.94)  Protein export cytoplasm chaperone protein (SecB2C maintains protein to be exported in unfolded state)  Transcriptional regulator2C AraC family  Zinc protease  conserved hypothetical protein  tRNA (cytosine34-2'-O-)-methyltransferase (EC 2.1.1.-)  tetR-family transcriptional regulatory protein |
| 10989 | AGHZ01000004.1:1..10989 | Alpha-2-macroglobulin  Inosine-uridine preferring nucleoside hydrolase (EC 3.2.2.1)  PrpF protein involved in 2-methylcitrate cycle  TonB-dependent receptor  hypothetical protein  hypothetical protein  hypothetical protein |
| 10981 | AGHZ01000086.1:1..10981 | 2-keto-3-deoxy-D-arabino-heptulosonate-7-phosphate synthase II (EC 2.5.1.54)  FIG01210215: hypothetical protein  GTP-binding protein TypA/BipA  Membrane protein  Zinc-regulated outer membrane receptor  arabinofuranosidase  hypothetical protein |
| 10925 | AGHZ01000134.1:1..10925 | ABC transporter2C ATP-binding protein  ATP-dependent DNA helicase Rep  FIG01210778: hypothetical protein  Fatty acid desaturase (EC 1.14.19.1); Delta-9 fatty acid desaturase (EC 1.14.19.1)  Formamidopyrimidine-DNA glycosylase (EC 3.2.2.23)  Glucans biosynthesis protein D precursor  Glycyl-tRNA synthetase beta chain (EC 6.1.1.14)  Thymidine kinase (EC 2.7.1.21)  hypothetical protein  hypothetical protein |
| 10878 | AGHZ01000285.1:1..10878 | Putative hemolysin  Sensory box sensor histidine kinase/response regulator  Ser/Thr protein phosphatase family protein2C UDP-22C3-diacylglucosamine hydrolase (EC 3.6.1.-) homolog  hypothetical protein  hypothetical protein  hypothetical protein  hypothetical protein  hypothetical protein  hypothetical protein  methyltransferase  tRNA dihydrouridine synthase B (EC 1.-.-.-) |
| 10843 | AGHZ01000040.1:1..10843 | Acetyltransferase  Cysteine desulfurase (EC 2.8.1.7)2C SufS subfamily  FIG01209781: hypothetical protein  Ferredoxin2C 2Fe-2S  Iron-sulfur cluster assembly ATPase protein SufC  Iron-sulfur cluster assembly protein SufB  Iron-sulfur cluster assembly protein SufD  Iron-sulfur cluster regulator IscR  Proteins containing SET domain  ThiJ/PfpI family protein  hypothetical protein  peptidase |
| 10369 | AGHZ01000067.1:1..10369 | Anthranilate synthase2C amidotransferase component (EC 4.1.3.27) @ Para-aminobenzoate synthase2C amidotransferase component (EC 2.6.1.85)  Anthranilate synthase2C aminase component (EC 4.1.3.27)  FIG01211564: hypothetical protein  FMN-dependent NADH-azoreductase  Low-specificity L-threonine aldolase (EC 4.1.2.5)  Nitrilotriacetate monooxygenase component B (EC 1.14.13.-)  Probable signal peptide protein  Transcription regulator [contains diacylglycerol kinase catalytic domain]  hypothetical protein  hypothetical protein  hypothetical protein  transcriptional regulator |
| 10298 | AGHZ01000081.1:1..10298 | 2-oxoglutarate dehydrogenase E1 component (EC 1.2.4.2)  Dihydrolipoamide dehydrogenase of 2-oxoglutarate dehydrogenase (EC 1.8.1.4)  Dihydrolipoamide succinyltransferase component (E2) of 2-oxoglutarate dehydrogenase complex (EC 2.3.1.61)  FIG01111324: hypothetical protein  Lysine decarboxylase family  hypothetical protein  hypothetical protein |
| 10276 | AGHZ01000146.1:1..10276 | Isocitrate dehydrogenase [NADP] (EC 1.1.1.42); Monomeric isocitrate dehydrogenase [NADP] (EC 1.1.1.42)  Isocitrate dehydrogenase phosphatase (EC 2.7.11.5)/kinase (EC 3.1.3.-)  Predicted exported glycosyl hydrolase family 31 protein  Predicted secreted alpha-N-acetylgalactosaminidase (EC 3.2.1.49)  TonB-dependent receptor  hypothetical protein  hypothetical protein |
| 10184 | AGHZ01000556.1:1..10184 | FIG01112193: hypothetical protein  FIG01214567: hypothetical protein  succinoglycan biosynthesis protein |
| 10154 | AGHZ01000172.1:1..10154 | Beta-lactamase class C and other penicillin binding proteins  Ferredoxin-dependent glutamate synthase (EC 1.4.7.1)  GCN5-related N-acetyltransferase  Transcriptional regulator lacI family  diguanylate cyclase/phosphodiesterase (GGDEF  hypothetical protein  hypothetical protein  polysaccharide biosynthetic protein |
| 10071 | AGHZ01000105.1:1..10071 | Pass1-related protein  Peptide transport system permease protein sapC (TC 3.A.1.5.5)  TonB-dependent receptor  Tryptophan halogenase  Xylulose kinase (EC 2.7.1.17)  hypothetical protein  sal operon transcriptional repressor |
| 10068 | AGHZ01000099.1:1..10068 | ABC transporter ATP-binding protein  ABC-type antimicrobial peptide transport system2C permease component  FIG01111875: hypothetical protein  Methionine ABC transporter ATP-binding protein  Predicted zinc-binding protein  TrbP protein  conserved hypothetical protein  hypothetical protein  hypothetical protein  phospholipase D family protein  putative; ORF located using Glimmer/Genemark |
| 9899 | AGHZ01000218.1:1..9899 | 3-oxoacyl-[acyl-carrier protein] reductase (EC 1.1.1.100)  Extracellular protease precursor (EC 3.4.21.-)  FMN-dependent NADH-azoreductase( EC:1.6.99.2 )  L-asparaginase (EC 3.5.1.1)  Redox-sensing transcriptional regulator QorR2C putative  Rrf2-linked NADH-flavin reductase  Transcriptional regulator2C LysR family  conserved hypothetical protein  drug resistance transporter2C EmrB/QacA subfamily  hypothetical protein |
| 9832 | AGHZ01000073.1:1..9832 | Alpha/beta hydrolase fold (EC 3.8.1.5)  Beta-galactosidase (EC 3.2.1.23)  NAD(P)H oxidoreductase YRKL (EC 1.6.99.-) @ Putative NADPH-quinone reductase (modulator of drug activity B) @ Flavodoxin 2  Xylanase  Xylanase  conserved hypothetical protein  hypothetical protein  hypothetical protein  hypothetical protein  inner membrane protein  putative lipoprotein |
| 9602 | AGHZ01001647.1:1..9602 | 3-oxoacyl-[acyl-carrier protein] reductase (EC 1.1.1.100)  DNA repair protein RecN  Ferric uptake regulation protein FUR  Glucose-methanol-choline (GMC) oxidoreductase:NAD binding site  Outer membrane lipoprotein SmpA2C a component of the essential YaeT outer-membrane protein assembly complex  Putative oligoketide cyclase/dehydratase or lipid transport protein YfjG  Serine peptidase  hypothetical protein  hypothetical protein  hypothetical protein  tmRNA-binding protein SmpB |
| 9510 | AGHZ01001658.1:1..9510 | 12C2-dihydroxy-3-keto-5-methylthiopentene dioxygenase (EC 1.13.11.54)  Adenosylhomocysteinase (EC 3.3.1.1)  Amino acid permease  Amino acid permease  D-2-hydroxyglutarate dehydrogenase  D-3-phosphoglycerate dehydrogenase (EC 1.1.1.95)  Methylthioribulose-1-phosphate dehydratase (EC 4.2.1.109)  hypothetical protein |
| 9377 | AGHZ01000061.1:1..9377 | Bacteriophage protein gp37  Pyruvate dehydrogenase E1 component (EC 1.2.4.1)  hypothetical protein  hypothetical protein |
| 9248 | AGHZ01000316.1:1..9248 | Alr1013 protein  CDP-diacylglycerol--glycerol-3-phosphate 3-phosphatidyltransferase (EC 2.7.8.5)  Excinuclease ABC subunit C  Ferrichrome-iron receptor  hypothetical protein  hypothetical protein  tRNA-Cys-GCA  tRNA-Gly-GCC |
| 9223 | AGHZ01000010.1:1..9223 | FIG01210654: hypothetical protein  FIG01210744: hypothetical protein  Flagellar motor protein  TonB-dependent receptor  hypothetical protein  hypothetical protein  putative membrane protein |
| 9138 | AGHZ01000122.1:1..9138 | Cellulase  Cellulase  Cellulase  Glutamate synthase [NADPH] large chain (EC 1.4.1.13)  Glutamate synthase [NADPH] small chain (EC 1.4.1.13)  Methylglyoxal synthase (EC 4.2.3.3)  hypothetical protein |
| 9136 | AGHZ01001717.1:1..9136 | Alanine dehydrogenase (EC 1.4.1.1)  Aldehyde dehydrogenase (EC 1.2.1.3)  Omega-amino acid--pyruvate aminotransferase (EC 2.6.1.18)  Putrescine utilization regulator  hypothetical protein  hypothetical protein  hypothetical protein  hypothetical protein |
| 9125 | AGHZ01000594.1:1..9125 | ABC transporter ATP-binding protein  Chaperone protein DnaJ  Chaperone protein DnaK  Cyclohexadienyl dehydrogenase (EC 1.3.1.12)(EC 1.3.1.43)  Heat shock protein GrpE  Heat-inducible transcription repressor HrcA  Pyridoxal kinase (EC 2.7.1.35)  hypothetical protein |
| 9098 | AGHZ01000006.1:1..9098 | Acetyl-coenzyme A synthetase (EC 6.2.1.1)  Integral membrane protein  Tetratricopeptide TPR\_2 repeat protein  TonB-dependent receptor  Transcriptional regulator2C LuxR family  hypothetical protein  hypothetical protein  hypothetical protein |
| 8982 | AGHZ01000323.1:1..8982 | 1-acyl-sn-glycerol-3-phosphate acyltransferase (EC 2.3.1.51)  Conserved domain protein  Exodeoxyribonuclease III (EC 3.1.11.2)  FIG01210401: hypothetical protein  Glycerol-3-phosphate regulon repressor2C DeoR family  Transcriptional regulator2C LysR family  hypothetical protein  hypothetical protein  hypothetical protein  hypothetical protein |
| 8858 | AGHZ01000426.1:1..8858 | ABC transporter ATP-binding protein  ABC transporter permease  FIG01211682: hypothetical protein  Glucosamine--fructose-6-phosphate aminotransferase [isomerizing] (EC 2.6.1.16)  Lipoprotein releasing system ATP-binding protein LolD  Transcriptional regulator2C TetR family  hypothetical protein  putative monooxygenase. |
| 8698 | AGHZ01000165.1:1..8698 | ABC transporter2C permease protein2C putative  ABC-type multidrug transport system2C ATPase component  FIG01211096: hypothetical protein  FKBP-type peptidyl-prolyl cis-trans isomerase FkpA precursor (EC 5.2.1.8)  Glutathione peroxidase (EC 1.11.1.9)  Permease of the drug/metabolite transporter (DMT) superfamily  Protein slyX homolog  UDP-glucose dehydrogenase (EC 1.1.1.22)  hypothetical protein  hypothetical protein  transcriptional regulator gntR family |
| 8692 | AGHZ01000167.1:1..8692 | DNA repair protein RadC  Mycobacteriophage Barnyard protein gp56  hypothetical protein  hypothetical protein  hypothetical protein |
| 8670 | AGHZ01000168.1:1..8670 | Acetyltransferase2C GNAT family (EC 2.3.1.-)  FIG01210523: hypothetical protein  Fibronectin type III domain protein  Microcystin dependent protein  Microcystin dependent protein  Microcystin dependent protein |
| 8660 | AGHZ01000238.1:1..8660 | ABC transporter ATP-binding protein  ABC transporter ATP-binding protein  ABC transporter ATP-binding protein  ABC transporter permease  ABC transporter permease  ABC transporter permease  ABC transporter permease |
| 8615 | AGHZ01000377.1:1..8615 | Excinuclease ABC subunit A paralog of unknown function  GCN5-related N-acetyltransferase  Guanosine polyphosphate pyrophosphohydrolases/synthetases  hypothetical protein  hypothetical protein  hypothetical protein  hypothetical protein  hypothetical protein  hypothetical protein  hypothetical protein |
| 8586 | AGHZ01000188.1:1..8586 | Citrate synthase (si) (EC 2.3.3.1)  FIG01209925: hypothetical protein  Inosine-uridine preferring nucleoside hydrolase (EC 3.2.2.1)  LSU ribosomal protein L31p  Multimodular transpeptidase-transglycosylase (EC 2.4.1.129) (EC 3.4.-.-)  Type IV pilus biogenesis protein PilM  hypothetical protein |
| 8566 | AGHZ01000581.1:1..8566 | DnaJ domain protein  Ferric siderophore transport system2C periplasmic binding protein TonB  LSU m3Psi1915 methyltransferase RlmH  TonB-dependent receptor  TonB-dependent receptor |
| 8566 | AGHZ01000136.1:1..8566 | Alcohol dehydrogenase (EC 1.1.1.1)  COG0840: Methyl-accepting chemotaxis protein  Thioredoxin reductase  Thioredoxin reductase  Transcriptional regulator2C HxlR family  hypothetical protein  hypothetical protein  hypothetical protein  putative transcriptional regulator |
| 8562 | AGHZ01000335.1:1..8562 | Flagellar biosynthesis protein FliL  Flagellar biosynthesis protein FliP  Flagellar biosynthesis protein FliQ  Flagellar biosynthesis protein FliQ  Flagellar biosynthesis protein FliR  Flagellar hook-length control protein FliK  Flagellar motor switch protein FliM  Flagellar motor switch protein FliN  hypothetical protein |
| 8552 | AGHZ01000520.1:1..8552 | ClpB protein  Glutathione S-transferase  O-acetylhomoserine sulfhydrylase (EC 2.5.1.49) / O-succinylhomoserine sulfhydrylase (EC 2.5.1.48)  PROBABLE TRANSMEMBRANE PROTEIN  Transcriptional regulator2C LysR family  hypothetical protein  hypothetical protein |
| 8504 | AGHZ01000116.1:1..8504 | Fucose permease  L-fuconate dehydratase (EC 4.2.1.68)  Pirin2C N-terminal:Pirin2C C-terminal  Possible alternative L-fucose mutarotase  Transcriptional regulator2C IclR family  cytochrome C biogenesis protein  hypothetical protein  hypothetical protein  hypothetical protein |
| 8439 | AGHZ01000719.1:1..8439 | Adenosylhomocysteinase (EC 3.3.1.1)  Conserved hypothetical protein 2001  FIG01212366: hypothetical protein  Methyl-accepting chemotaxis protein I (serine chemoreceptor protein)  cytochrome P450 hydroxylase  hypothetical protein  hypothetical protein  methyltransferase  sulfotransferase |
| 8358 | AGHZ01001457.1:1..8358 | Inositol-1-monophosphatase (EC 3.1.3.25)  Lysyl-lysine 22C3-aminomutase  Phosphate-binding protein  diguanylate cyclase/phosphodiesterase (GGDEF  hypothetical protein  tRNA:Cm32/Um32 methyltransferase |
| 8317 | AGHZ01001522.1:1..8317 | Transcriptional regulator2C AraC family  hypothetical protein  hypothetical protein  hypothetical protein  hypothetical protein  hypothetical protein  hypothetical protein  hypothetical protein |
| 8286 | AGHZ01000170.1:1..8286 | Alanine racemase (EC 5.1.1.1)  D-amino acid dehydrogenase small subunit (EC 1.4.99.1)  FIG01111765: hypothetical protein  FIG01210350: hypothetical protein  L-sorbosone dehydrogenase  Sensor histidine kinase  hypothetical protein  hypothetical protein  hypothetical protein |
| 8281 | AGHZ01000083.1:1..8281 | FIG01211451: hypothetical protein  G:T/U mismatch-specific uracil/thymine DNA-glycosylase  Methionine ABC transporter ATP-binding protein  Methionine ABC transporter permease protein  Methionine ABC transporter substrate-binding protein  Salicylate hydroxylase (EC 1.14.13.1) (Salicylate 1-monooxygenase)  UPF0234 protein YajQ  hypothetical protein  outer membrane protein  putative membrane protein |
| 8273 | AGHZ01000227.1:1..8273 | DNA repair protein RadC  Fic family protein  Type I restriction-modification system2C DNA-methyltransferase subunit M (EC 2.1.1.72)  Type I restriction-modification system2C restriction subunit R (EC 3.1.21.3)  Type I restriction-modification system2C specificity subunit S (EC 3.1.21.3) |
| 8264 | AGHZ01000622.1:1..8264 | Beta-galactosidase (EC 3.2.1.23)  FIG01213069: hypothetical protein  TonB-dependent receptor  hypothetical protein |
| 8258 | AGHZ01000008.1:1..8258 | FIG01211949: hypothetical protein  FIG01213271: hypothetical protein  Glutathione S-transferase (EC 2.5.1.18)  Propionate--CoA ligase (EC 6.2.1.17)  hypothetical protein  hypothetical protein  hypothetical protein  hypothetical protein |
| 8239 | AGHZ01000187.1:1..8239 | Outer membrane receptor proteins2C mostly Fe transport  hypothetical protein  hypothetical protein  putative Glutathione-regulated potassium-efflux system protein KefB  putative secreted protein  sensor kinase |
| 8229 | AGHZ01001554.1:1..8229 | FIG01210031: hypothetical protein  Predicted sucrose-specific TonB-dependent receptor  ThiJ/PfpI family protein  alpha-amylase (EC 3.2.1.1) |
| 8196 | AGHZ01000423.1:1..8196 | 3-oxoacyl-[acyl-carrier-protein] synthase2C KASIII in hypothetical gene cluster  Acyl-CoA synthetase (AMP-forming)/AMP-acid ligase/Peptide synthase  FIG01210420: hypothetical protein  FIG01212698: hypothetical protein  Ferredoxin  Hydrolase2C alpha/beta fold family protein2C in hypothetical gene cluster  MloA |
| 8185 | AGHZ01000055.1:1..8185 | Xanthan biosynthesis acetyltransferase GumF  Xanthan biosynthesis glucuronosyltransferase GumK  Xanthan biosynthesis glycosyltransferase GumH  Xanthan biosynthesis glycosyltransferase GumI  Xanthan biosynthesis glycosyltransferase GumM  Xanthan biosynthesis oligosaccharidyl-lipid flippase GumJ  Xanthan biosynthesis pyruvyltransferase GumL |
| 8160 | AGHZ01001021.1:1..8160 | Acetyl-coenzyme A carboxyl transferase alpha chain (EC 6.4.1.2)  DNA polymerase III alpha subunit (EC 2.7.7.7)  Hypothetical membrane protein2C possible involvement in cytochrome functioning/assembly  Lipid-A-disaccharide synthase (EC 2.4.1.182)  Ribonuclease HII (EC 3.1.26.4)  hypothetical protein |
| 8142 | AGHZ01000561.1:1..8142 | C-di-GMP phosphodiesterase A  FIG01210950: hypothetical protein  Flagellar basal-body P-ring formation protein FlgA  Histidine kinase  HrpX related protein  Negative regulator of flagellin synthesis FlgM |
| 8116 | AGHZ01000130.1:1..8116 | Glutathione-regulated potassium-efflux system ATP-binding protein  TonB-dependent receptor  TonB-dependent receptor  hypothetical protein  hypothetical protein |
| 8101 | AGHZ01000823.1:1..8101 | 23S rRNA (guanosine-2'-O-) -methyltransferase rlmB (EC 2.1.1.-)  3'-to-5' exoribonuclease RNase R  Beta-glucosidase (EC 3.2.1.21)  Gfa-like protein  VirK family protein  hypothetical protein  hypothetical protein  tRNA-Leu-CAG  tRNA-Leu-CAG |
| 8087 | AGHZ01000256.1:1..8087 | D-alanyl-D-alanine carboxypeptidase (EC 3.4.16.4)  Lipoate synthase  Octanoate-[acyl-carrier-protein]-protein-N-octanoyltransferase  Proposed lipoate regulatory protein YbeD  hypothetical protein  probable outer membrane protein  tail-specific protease |
| 8057 | AGHZ01000182.1:1..8057 | Dipeptidyl peptidase IV  FIG01112087: hypothetical protein  FIG01211910: hypothetical protein  Glutathione S-transferase (EC 2.5.1.18)  hypothetical protein |
| 8049 | AGHZ01000331.1:1..8049 | 2-amino-4-hydroxy-6-hydroxymethyldihydropteridine pyrophosphokinase (EC 2.7.6.3)  COG1565: Uncharacterized conserved protein  FIG01111359: hypothetical protein  FolM Alternative dihydrofolate reductase 1  Histidine kinase/response regulator hybrid protein  Phytochrome2C two-component sensor histidine kinase (EC 2.7.3.-)  two-component system regulatory protein |
| 8023 | AGHZ01000181.1:1..8023 | COG28792C Hypothetical small protein yjiX  Carbon starvation protein A  Endonuclease V (EC 3.1.21.7)  FIG01210964: hypothetical protein  Phosphoglycerate mutase (EC 5.4.2.1)  Pirin-related protein  hypothetical protein  hypothetical protein |
| 7973 | AGHZ01000063.1:1..7973 | NADH ubiquinone oxidoreductase chain A (EC 1.6.5.3)  NADH-ubiquinone oxidoreductase chain B (EC 1.6.5.3)  NADH-ubiquinone oxidoreductase chain C (EC 1.6.5.3)  NADH-ubiquinone oxidoreductase chain D (EC 1.6.5.3)  NADH-ubiquinone oxidoreductase chain E (EC 1.6.5.3)  NADH-ubiquinone oxidoreductase chain F (EC 1.6.5.3)  Preprotein translocase subunit SecG (TC 3.A.5.1.1)  Triosephosphate isomerase (EC 5.3.1.1)  tRNA-Leu-GAG |
| 7933 | AGHZ01000792.1:1..7933 | Flavodoxin reductases (ferredoxin-NADPH reductases) family 1  Nitrogen regulation protein NR(I)  POSSIBLE LINOLEOYL-CoA DESATURASE (DELTA(6)-DESATURASE)  Pyruvate oxidase [ubiquinone2C cytochrome] (EC 1.2.2.2)  hypothetical protein  hypothetical protein  protein of unknown function DUF1452  two-component system sensor protein |
| 7900 | AGHZ01001685.1:1..7900 | Aconitate hydratase 2 (EC 4.2.1.3)  Chemotaxis response regulator protein-glutamate methylesterase CheB (EC 3.1.1.61)  FIG01210619: hypothetical protein  FIG01211894: hypothetical protein  Phosphate-specific outer membrane porin OprP ; Pyrophosphate-specific outer membrane porin OprO  diguanylate cyclase/phosphodiesterase (GGDEF  hypothetical protein |
| 7882 | AGHZ01000504.1:1..7882 | Alpha-L-fucosidase (EC 3.2.1.51)  FIG01210595: hypothetical protein  N-acetylglucosamine-regulated TonB-dependent outer membrane receptor  hypothetical protein  predicted N-acetylglucosamine kinase2C glucokinase-like (EC 2.7.1.59) |
| 7876 | AGHZ01000534.1:1..7876 | Beta-carotene ketolase (EC 1.14.-.-)  TonB-dependent receptor  hypothetical protein  prolyl oligopeptidase family protein |
| 7863 | AGHZ01001566.1:1..7863 | Autolysis response regulater LytR  Glucans biosynthesis glucosyltransferase H (EC 2.4.1.-)  Isochorismatase (EC 3.3.2.1)  Phospholipase/Carboxylesterase  histidine kinase-response regulator hybrid protein  hypothetical protein  hypothetical protein  hypothetical protein  single-domain response regulator |
| 7852 | AGHZ01000211.1:1..7852 | 4-hydroxybenzoate polyprenyltransferase (EC 2.5.1.-)  Competence protein F homolog2C phosphoribosyltransferase domain; protein YhgH required for utilization of DNA as sole source of carbon and energy  FIG01211043: hypothetical protein  MG(2 ) CHELATASE FAMILY PROTEIN / ComM-related protein  Nitrogen regulatory protein P-II  Putative hydrolase  Spermidine synthase (EC 2.5.1.16)  hypothetical protein  hypothetical protein  tRNA-Arg-CCG |
| 7846 | AGHZ01000145.1:1..7846 | 2-octaprenyl-3-methyl-6-methoxy-12C4-benzoquinol hydroxylase (EC 1.14.13.-)  Adenosine (5')-pentaphospho-(5'')-adenosine pyrophosphohydrolase (EC 3.6.1.-)  Bacterioferritin  Cyclic AMP receptor protein  LSU ribosomal protein L13p (L13Ae)  Quaternary ammonium compound-resistance protein sugE  S-adenosylmethionine decarboxylase proenzyme (EC 4.1.1.50)2C prokaryotic class 1A  SSU ribosomal protein S9p (S16e)  bacterioferritin-associated ferredoxin  hypothetical protein  hypothetical protein  tRNA-Gln-CTG  tRNA-Met-CAT  two-component system sensor protein |
| 7789 | AGHZ01000388.1:1..7789 | Protein Implicated in DNA repair function with RecA and MutS  RecA protein  Regulatory protein RecX  SOS-response repressor and protease LexA (EC 3.4.21.88)  Ubiquinone biosynthesis monooxygenase UbiB  hypothetical protein |
| 7787 | AGHZ01000420.1:1..7787 | 52C10-methylenetetrahydrofolate reductase (EC 1.5.1.20)  Alpha-amylase  Alpha-amylase  Alpha-amylase  FIG01211153: hypothetical protein  FIG01212745: hypothetical protein  hypothetical protein  hypothetical protein  hypothetical protein |
| 7777 | AGHZ01000163.1:1..7777 | Biotin carboxylase (EC 6.3.4.14)  Feruloyl-CoA synthetase  Hydrolase or peptidase  Probable FERULIC acid hydratase protein  hypothetical protein  hypothetical protein |
| 7737 | AGHZ01000545.1:1..7737 | Cation:proton antiporter  Uncharacterized glutathione S-transferase-like protein  hypothetical protein  peptidase M192C renal dipeptidase |
| 7688 | AGHZ01000101.1:1..7688 | ABC transporter ATP-binding protein USSDB6B  ABC-type transport system involved in resistance to organic solvents2C periplasmic component USSDB6C  ABC-type transport system involved in resistance to organic solvents2C permease component USSDB6A  DNA-binding protein H-NS  Membrane lipoprotein lipid attachment site containing protein USSDB6D  Prolyl-tRNA synthetase (EC 6.1.1.15)2C bacterial type  hypothetical protein |
| 7679 | AGHZ01000531.1:1..7679 | Catalase (EC 1.11.1.6)  Glycine dehydrogenase [decarboxylating] (glycine cleavage system P protein) (EC 1.4.4.2)  MFS transporter  hypothetical protein  hypothetical protein |
| 7664 | AGHZ01000278.1:1..7664 | ATP-dependent hsl protease ATP-binding subunit HslU  ATP-dependent protease HslV (EC 3.4.25.-)  Diaminopimelate epimerase (EC 5.1.1.7)  FIG01211065: hypothetical protein  NAD(P) transhydrogenase alpha subunit (EC 1.6.1.2)  Protein of unknown function DUF484  Site-specific tyrosine recombinase  hypothetical protein  hypothetical protein  hypothetical protein |
| 7658 | AGHZ01000525.1:1..7658 | DNA repair protein RadA  DltE  PDZ domain family protein  Transcriptional regulator2C MarR family  hypothetical protein  hypothetical protein  hypothetical protein |
| 7546 | AGHZ01000632.1:1..7546 | FIG01211412: hypothetical protein  FIG01213032: hypothetical protein  GCN5-related N-acetyltransferase  Lytic enzyme  Phosphoribosylaminoimidazole-succinocarboxamide synthase (EC 6.3.2.6)  Ribulose-phosphate 3-epimerase (EC 5.1.3.1)  hypothetical protein  hypothetical protein  hypothetical protein |
| 7474 | AGHZ01000069.1:1..7474 | Dehydrogenases with different specificities (related to short-chain alcohol dehydrogenases)  FIG01211551: hypothetical protein  Glucoamylase (EC 3.2.1.3)  RND efflux system2C outer membrane lipoprotein CmeC  hypothetical protein  hypothetical protein  hypothetical protein |
| 7426 | AGHZ01000002.1:1..7426 | GumB protein  Integration host factor alpha subunit  Phenylalanyl-tRNA synthetase beta chain (EC 6.1.1.20)  Transcriptional regulator2C MerR family  Xanthan biosynthesis chain length determinant protein GumC  Xanthan biosynthesis exopolysaccharide polymerase GumE  Xanthan biosynthesis glycosyltransferase GumD  tRNA-Pro-GGG |
| 7419 | AGHZ01000158.1:1..7419 | TonB-dependent receptor  TonB-dependent receptor  hypothetical protein  related to competence proteins |
| 7316 | AGHZ01000070.1:1..7316 | DNA recombination-dependent growth factor C  FIG01212167: hypothetical protein  hypothetical protein  hypothetical protein  hypothetical protein  hypothetical protein  hypothetical protein  photolyase protein family  tRNA(Cytosine32)-2-thiocytidine synthetase |
| 7296 | AGHZ01001553.1:1..7296 | Acetoacetyl-CoA reductase (EC 1.1.1.36)  Phosphate-specific outer membrane porin OprP ; Pyrophosphate-specific outer membrane porin OprO  Predicted maltose transporter MalT  Tricarboxylate transport transcriptional regulator TctD  Uncharacterized transporter2C similarity to citrate transporter  transcriptional regulator2C LacI family |
| 7277 | AGHZ01000065.1:1..7277 | Os01g0879400  Putative glycosyl hydrolase of unknown function (DUF1680)  TonB-dependent receptor  Ureidoglycolate/malate/sulfolactate dehydrogenase family (EC 1.1.1.-) |
| 7271 | AGHZ01000701.1:1..7271 | 4-carboxymuconolactone decarboxylase (EC 4.1.1.44)  4-oxalocrotonate tautomerase  Cell division protein FtsN  Oxidoreductase  TonB-dependent receptor  hypothetical protein  pyridoxamine 5'-phosphate oxidase-related2C FMN-binding |
| 7254 | AGHZ01000175.1:1..7254 | COG0436: Aspartate/tyrosine/aromatic aminotransferase  Prolyl endopeptidase (EC 3.4.21.26)  Protease II (EC 3.4.21.83)  hypothetical protein |
| 7219 | AGHZ01000436.1:1..7219 | 2-hydroxy-3-keto-5-methylthiopentenyl-1-phosphate phosphatase related protein  3-oxoacyl-[acyl-carrier-protein] synthase2C KASII (EC 2.3.1.41)  Aminotransferase2C class III  Coproporphyrinogen III oxidase2C oxygen-independent (EC 1.3.99.22)  hypothetical protein  putative sec-independent protein translocase protein TatC  tRNA-Leu-TAA |
| 7158 | AGHZ01000788.1:1..7158 | 3-oxoacyl-[acyl-carrier-protein] synthase2C KASIII (EC 2.3.1.41)  Acetyltransferase  Acyl carrier protein  Aminotransferase2C DegT/DnrJ/EryC1/StrS family  Flagellar regulatory protein FleQ  Oxidoreductase2C short chain dehydrogenase/reductase family  RNA polymerase sigma-54 factor RpoN  response regulator  short chain dehydrogenase |
| 7130 | AGHZ01001182.1:1..7130 | Alkyl hydroperoxide reductase protein F (EC 1.6.4.-)  Hydrogen peroxide-inducible genes activator  Peptide methionine sulfoxide reductase MsrA (EC 1.8.4.11)  Regulator of nucleoside diphosphate kinase  ThiJ/PfpI family protein  Transaldolase (EC 2.2.1.2)  hypothetical protein  hypothetical protein |
| 7100 | AGHZ01000440.1:1..7100 | Methyl-accepting chemotaxis protein I (serine chemoreceptor protein)  Signal transduction histidine kinase CheA (EC 2.7.3.-) |
| 7086 | AGHZ01000381.1:1..7086 | Outer membrane protein  Protein of unknown function YceH  acetyltransferase  hypothetical protein  hypothetical protein |
| 7080 | AGHZ01000226.1:1..7080 | hypothetical protein  hypothetical protein  putative Cytochrome bd22C subunit I  putative Cytochrome bd22C subunit II |
| 7045 | AGHZ01000114.1:1..7045 | FIG01210277: hypothetical protein  FIG01211069: hypothetical protein  Homocysteine S-methyltransferase (EC 2.1.1.10)  Hypothetical protein YaeR with similarity to glyoxylase family  Methionyl-tRNA synthetase (EC 6.1.1.10) |
| 7033 | AGHZ01000263.1:1..7033 | ADP-ribosylglycohydrolase  Asparaginyl-tRNA synthetase (EC 6.1.1.22)  FIG01209847: hypothetical protein  FIG01211446: hypothetical protein  Iron binding protein SufA for iron-sulfur cluster assembly  LSU ribosomal protein L9p  SSU ribosomal protein S18p  SSU ribosomal protein S6p  hypothetical protein  hypothetical protein  transcriptional regulator protein Pai2 |
| 7016 | AGHZ01000207.1:1..7016 | Chemotaxis protein methyltransferase CheR (EC 2.1.1.80)  Chemotaxis response regulator protein-glutamate methylesterase CheB (EC 3.1.1.61)  Putative permease  Putative regulatory protein  diguanylate cyclase/phosphodiesterase (GGDEF |
| 7012 | AGHZ01000965.1:1..7012 | FIG01211838: hypothetical protein  FOG: PAS/PAC domain  Glutathione S-transferase (EC 2.5.1.18)  Probable protease htpX homolog (EC 3.4.24.-)  YaeQ protein  hypothetical protein  hypothetical protein  major cold shock protein |
| 6993 | AGHZ01000286.1:1..6993 | Ethidium bromide-methyl viologen resistance protein EmrE  Periplasmic aromatic aldehyde oxidoreductase2C iron-sulfur subunit YagT  TonB-dependent receptor  Zinc-regulated outer membrane receptor |
| 6990 | AGHZ01000634.1:1..6990 | Butyryl-CoA dehydrogenase (EC 1.3.99.2)  RND efflux system2C inner membrane transporter CmeB  Transcriptional regulator2C ArsR family / Methyltransferase fusion |
| 6988 | AGHZ01000351.1:1..6988 | Flagellar L-ring protein FlgH  Flagellar P-ring protein FlgI  Flagellar basal-body rod modification protein FlgD  Flagellar basal-body rod protein FlgB  Flagellar basal-body rod protein FlgC  Flagellar basal-body rod protein FlgF  Flagellar basal-body rod protein FlgG  Flagellar hook protein FlgE |
| 6958 | AGHZ01000451.1:1..6958 | 4-hydroxythreonine-4-phosphate dehydrogenase (EC 1.1.1.262)  ApaG protein  Bis(5'-nucleosyl)-tetraphosphatase2C symmetrical (EC 3.6.1.41)  Dihydrofolate reductase (EC 1.5.1.3)  Dimethyladenosine transferase (EC 2.1.1.-)  FIG004694: Hypothetical protein  Prolipoprotein diacylglyceryl transferase (EC 2.4.99.-)  Thymidylate synthase (EC 2.1.1.45) |
| 6953 | AGHZ01000243.1:1..6953 | Exodeoxyribonuclease V alpha chain (EC 3.1.11.5)  FIG01210492: hypothetical protein  Lysophospholipase (EC 3.1.1.5)  Putative DNA-binding protein in cluster with Type I restriction-modification system  hypothetical protein  hypothetical protein  hypothetical protein  hypothetical protein |
| 6891 | AGHZ01000491.1:1..6891 | FIG01210302: hypothetical protein  Oxidoreductase  Pca regulon regulatory protein PcaR  Putative oxidoreductase YncB  Ribosomal large subunit pseudouridine synthase E (EC 4.2.1.70)  hypothetical protein  macromolecule metabolism; macromolecule synthesis2C modification; dna - replication2C repair2C restr./modif. |
| 6882 | AGHZ01000299.1:1..6882 | FIG01210644: hypothetical protein  Flavoredoxin  Molybdenum ABC transporter2C periplasmic molybdenum-binding protein ModA (TC 3.A.1.8.1)  Molybdenum transport ATP-binding protein ModC (TC 3.A.1.8.1)  Molybdenum transport system permease protein ModB (TC 3.A.1.8.1)  ankyrin-related protein  transcriptional regulator blaI family |
| 6870 | AGHZ01000064.1:1..6870 | 6-phosphogluconolactonase (EC 3.1.1.31)2C eukaryotic type  Folate-dependent protein for Fe/S cluster synthesis/repair in oxidative stress  Glucokinase (EC 2.7.1.2)  Glucose-6-phosphate 1-dehydrogenase (EC 1.1.1.49)  hypothetical protein  sugar ABC transporter ATP-binding protein |
| 6868 | AGHZ01000230.1:1..6868 | Aerobic C4-dicarboxylate transporter for fumarate2C L-malate2C D-malate2C succunate2C aspartate  NADP-dependent malic enzyme (EC 1.1.1.40)  Phosphate-specific outer membrane porin OprP ; Pyrophosphate-specific outer membrane porin OprO  hypothetical protein  hypothetical protein  hypothetical protein |
| 6834 | AGHZ01000711.1:1..6834 | LSU ribosomal protein L20p  LSU ribosomal protein L35p  Phenylalanyl-tRNA synthetase alpha chain (EC 6.1.1.20)  Threonyl-tRNA synthetase (EC 6.1.1.3)  Transcriptional regulator of maltose utilization2C LacI family  Translation initiation factor 3  hypothetical protein |
| 6792 | AGHZ01000026.1:1..6792 | FIG01214235: hypothetical protein  L-sorbosone dehydrogenase  Membrane protein2C putative  Methionine aminopeptidase (EC 3.4.11.18)  RND multidrug efflux transporter; Acriflavin resistance protein  hypothetical protein |
| 6739 | AGHZ01000052.1:1..6739 | Polyribonucleotide nucleotidyltransferase (EC 2.7.7.8)  SSU ribosomal protein S15p (S13e)  diguanylate cyclase with GAF sensor  hypothetical protein  tRNA pseudouridine synthase B (EC 4.2.1.70) |
| 6708 | AGHZ01000249.1:1..6708 | Rod shape-determining protein MreB  Sugar kinase  hypothetical protein  hypothetical protein |
| 6699 | AGHZ01000138.1:1..6699 | 5-Enolpyruvylshikimate-3-phosphate synthase (EC 2.5.1.19)  Chorismate mutase I (EC 5.4.99.5) / Prephenate dehydratase (EC 4.2.1.51)  FIG001196: putative membrane protein  Phosphoserine aminotransferase (EC 2.6.1.52)  Putative sulfite oxidase subunit YedY  hypothetical protein  hypothetical protein  hypothetical protein |
| 6657 | AGHZ01000851.1:1..6657 | Potassium-transporting ATPase A chain (EC 3.6.3.12) (TC 3.A.3.7.1)  Potassium-transporting ATPase B chain (EC 3.6.3.12) (TC 3.A.3.7.1)  hypothetical protein  hypothetical protein  hypothetical protein |
| 6652 | AGHZ01000447.1:1..6652 | Hypothetical protein YggS2C proline synthase co-transcribed bacterial homolog PROSC  Twitching motility protein PilT  Twitching motility protein PilT  hypothetical protein  putative secreted protein |
| 6596 | AGHZ01000090.1:1..6596 | ATP-dependent helicase DinG/Rad3  Catalase (EC 1.11.1.6)  FOG: Ankyrin repeat  Shikimate 5-dehydrogenase I alpha (EC 1.1.1.25)  hypothetical protein  hypothetical protein  hypothetical protein |
| 6582 | AGHZ01001267.1:1..6582 | 1-hydroxy-2-methyl-2-(E)-butenyl 4-diphosphate synthase (EC 1.17.7.1)  Chaperone protein hscC (Hsc62)  Membrane-associated phospholipid phosphatase  hypothetical protein  hypothetical protein  hypothetical protein  hypothetical protein |
| 6502 | AGHZ01000575.1:1..6502 | Acyltransferase  FIG01210478: hypothetical protein  Hypothetical protein YaeJ with similarity to translation release factor  O-methyltransferase  TonB-dependent receptor  tRNA pseudouridine synthase C (EC 4.2.1.70) |
| 6443 | AGHZ01000441.1:1..6443 | FIG01210717: hypothetical protein  FIG01211456: hypothetical protein  Glucose dehydrogenase2C PQQ-dependent (EC 1.1.5.2)  NAD synthetase (EC 6.3.1.5) / Glutamine amidotransferase chain of NAD synthetase  hypothetical protein |
| 6435 | AGHZ01000260.1:1..6435 | 5'-nucleotidase (EC 3.1.3.5)  NAD kinase (EC 2.7.1.23)  NAD-specific glutamate dehydrogenase (EC 1.4.1.2)2C large form  hypothetical protein  hypothetical protein |
| 6410 | AGHZ01000343.1:1..6410 | FIG01212902: hypothetical protein  Putative exported protein precursor  Twin-arginine translocation pathway signal( EC:1.11.1.10 )  hypothetical protein  hypothetical protein  hypothetical protein  hypothetical protein  tRNA-Gly-GCC  two-component system regulatory protein |
| 6388 | AGHZ01000839.1:1..6388 | ABC-type multidrug transport system2C ATPase component  ABC-type multidrug transport system2C permease component  FIG01210488: hypothetical protein  FIG01211407: hypothetical protein  Leucine-responsive regulatory protein2C regulator for leucine (or lrp) regulon and high-affinity branched-chain amino acid transport system  Na /H antiporter  Permease of the drug/metabolite transporter (DMT) superfamily |
| 6373 | AGHZ01000596.1:1..6373 | FIG01209691: hypothetical protein  S-(hydroxymethyl)glutathione dehydrogenase (EC 1.1.1.284)  TonB-dependent receptor  surface antigen gene  transcriptional regulator |
| 6349 | AGHZ01000044.1:1..6349 | OmpA-related protein  Orotidine 5'-phosphate decarboxylase (EC 4.1.1.23)  Transcriptional regulator2C LacI family  Tryptophan halogenase  coagulation factor 5/8 type-like |
| 6322 | AGHZ01001709.1:1..6322 | Di-/tripeptide transporter  FIG01211421: hypothetical protein  FIG01211502: hypothetical protein  FIG01211598: hypothetical protein  Transcription repressor  hypothetical protein |
| 6313 | AGHZ01000469.1:1..6313 | Catalase (EC 1.11.1.6)  FIG01210888: hypothetical protein  Oxidoreductase  Protein yciF  Unsaturated fatty acid biosythesis repressor FabR2C TetR family  hypothetical protein  hypothetical protein |
| 6312 | AGHZ01000219.1:1..6312 | General secretion pathway protein E / Type II secretion cytoplasmic ATP binding protein (PulE2C ATPase)  General secretion pathway protein F  Outer membrane protein  Protease  hypothetical protein |
| 6291 | AGHZ01001713.1:1..6291 | Exodeoxyribonuclease I (EC 3.1.11.1)  FIG01210780: hypothetical protein  FIG01212834: hypothetical protein  Kynureninase (EC 3.7.1.3)  Kynurenine 3-monooxygenase (EC 1.14.13.9)  hypothetical protein  hypothetical protein |
| 6227 | AGHZ01000053.1:1..6227 | (3R)-hydroxymyristoyl-[acyl carrier protein] dehydratase (EC 4.2.1.-)  Acyl-[acyl-carrier-protein]--UDP-N-acetylglucosamine O-acyltransferase (EC 2.3.1.129)  Membrane-associated zinc metalloprotease  Outer membrane protein assembly factor YaeT precursor  UDP-3-O-[3-hydroxymyristoyl] glucosamine N-acyltransferase (EC 2.3.1.-) |
| 6225 | AGHZ01000413.1:1..6225 | CDP-diacylglycerol pyrophosphatase (EC 3.6.1.26)  Carboxypeptidase C (cathepsin A)  Probable low-affinity inorganic phosphate transporter  Topoisomerase IV subunit B (EC 5.99.1.-) |
| 6204 | AGHZ01000045.1:1..6204 | Succinyl-CoA ligase [ADP-forming] alpha chain (EC 6.2.1.5)  Succinyl-CoA ligase [ADP-forming] beta chain (EC 6.2.1.5)  Two-component sensor PilS |
| 6201 | AGHZ01000046.1:1..6201 | Carbon-nitrogen hydrolase  Glutamyl-tRNA synthetase (EC 6.1.1.17)  Histone acetyltransferase HPA2 and related acetyltransferases  Zinc uptake regulation protein ZUR  putative cytoplasmic protein |
| 6185 | AGHZ01000391.1:1..6185 | Potassium efflux system KefA protein / Small-conductance mechanosensitive channel  Quinone oxidoreductase (EC 1.6.5.5)  Ubiquinone biosynthesis monooxygenase UbiB  hypothetical protein |
| 6178 | AGHZ01000084.1:1..6178 | FIG01210409: hypothetical protein  TonB-dependent receptor |
| 6159 | AGHZ01000269.1:1..6159 | Adenylosuccinate lyase (EC 4.3.2.2)  FIG01111779: hypothetical protein  FIG01211089: hypothetical protein  Fumarate hydratase class II (EC 4.2.1.2)  hypothetical protein  hypothetical protein |
| 6150 | AGHZ01000366.1:1..6150 | FIG01209954: hypothetical protein  Nicotinamide phosphoribosyltransferase (EC 2.4.2.12)  Nicotinamide-nucleotide adenylyltransferase2C NadM family (EC 2.7.7.1) / ADP-ribose pyrophosphatase (EC 3.6.1.13)  arabinogalactan endo-12C4-beta-galactosidase  transcriptional regulator |
| 6149 | AGHZ01000703.1:1..6149 | FIG01111128: hypothetical protein  N-acetylgalactosamine kinase2C ROK-type (EC 2.7.1.157)  N-acetylglucosamine-6-phosphate deacetylase (EC 3.5.1.25)  NADPH dependent preQ0 reductase  Tagatose-6-phosphate kinase AgaZ (EC 2.7.1.144)  Transcriptional repressor of aga operon  hypothetical protein  hypothetical protein |
| 6117 | AGHZ01000751.1:1..6117 | 16S rRNA processing protein RimM  2-nitropropane dioxygenase  Aminodeoxychorismate lyase (EC 4.1.3.38)  LSU ribosomal protein L19p  Na -driven multidrug efflux pump  Ribosome-associated heat shock protein implicated in the recycling of the 50S subunit (S4 paralog)  SSU ribosomal protein S16p  tRNA (Guanine37-N1) -methyltransferase (EC 2.1.1.31) |
| 6092 | AGHZ01000425.1:1..6092 | Periplasmic beta-glucosidase (EC 3.2.1.21)  Predicted sodium-dependent galactose transporter  diguanylate cyclase/phosphodiesterase (GGDEF |
| 6050 | AGHZ01000818.1:1..6050 | Metallopeptidase  Metallopeptidase  hypothetical protein  integral membrane rhomboid family serine protease MJ0610.1  methylated-DNA-protein-cysteine S-methyltransferase related protein |
| 6043 | AGHZ01000132.1:1..6043 | Sulfate adenylyltransferase subunit 1 (EC 2.7.7.4) / Adenylylsulfate kinase (EC 2.7.1.25)  Sulfate adenylyltransferase subunit 2 (EC 2.7.7.4)  bacterial lipid A biosynthesis acyltransferase family |
| 6027 | AGHZ01000222.1:1..6027 | FIG01210028: hypothetical protein  GCN5-related N-acetyltransferase  Histidine kinase/response regulator hybrid protein  N-acetylglucosamine-1-phosphate uridyltransferase (EC 2.7.7.23) / Glucosamine-1-phosphate N-acetyltransferase (EC 2.3.1.157)  Periplasmic chorismate mutase I precursor (EC 5.4.99.5)  Sensor histidine kinase |
| 6025 | AGHZ01000806.1:1..6025 | Amidases related to nicotinamidase  LigA  Mannose-6-phosphate isomerase  Transcriptional regulator2C AraC family  efflux transporter2C RND family2C MFP subunit |
| 6020 | AGHZ01000692.1:1..6020 | FIG006972: hypothetical protein  Protein export cytoplasm protein SecA ATPase RNA helicase (TC 3.A.5.1.1)  UDP-3-O-[3-hydroxymyristoyl] N-acetylglucosamine deacetylase (EC 3.5.1.-)  hypothetical protein  peptidase |
| 6010 | AGHZ01000191.1:1..6010 | Putative protein-S-isoprenylcysteine methyltransferase  Short-chain alcohol dehydrogenase family  Two-component system regulatory protein  hypothetical protein  hypothetical protein  predicted membrane protein  two-component system sensor protein |
| 6009 | AGHZ01000955.1:1..6009 | 23S rRNA (guanine-N-2-) -methyltransferase rlmL EC 2.1.1.-)  FIG00506329: hypothetical protein  hypothetical protein  two-component system regulatory protein |
| 6003 | AGHZ01001518.1:1..6003 | FIG01211483: hypothetical protein  L-serine dehydratase (EC 4.3.1.17)  Probable signal peptide protein  cytochrome B561  hypothetical protein |
| 5976 | AGHZ01000155.1:1..5976 | ABC transporter involved in cytochrome c biogenesis2C ATPase component CcmA  FIG01209836: hypothetical protein  Methionine aminotransferase2C PLP-dependent  Possible hydrolase  hypothetical protein  hypothetical protein  hypothetical protein |
| 5964 | AGHZ01000307.1:1..5964 | ATP synthase alpha chain (EC 3.6.3.14)  ATP synthase beta chain (EC 3.6.3.14)  ATP synthase delta chain (EC 3.6.3.14)  ATP synthase epsilon chain (EC 3.6.3.14)  ATP synthase gamma chain (EC 3.6.3.14) |
| 5937 | AGHZ01000797.1:1..5937 | ATP-dependent Clp protease ATP-binding subunit ClpA  ATP-dependent Clp protease adaptor protein ClpS  Leucyl/phenylalanyl-tRNA--protein transferase (EC 2.3.2.6)  Nudix-like NDP and NTP phosphohydrolase YmfB  Translation initiation factor 1  conserved hypothetical protein  hypothetical protein |
| 5894 | AGHZ01000710.1:1..5894 | FIG01209964: hypothetical protein  Glycerol-3-phosphate acyltransferase (EC 2.3.1.15)  hypothetical protein  hypothetical protein  hypothetical protein  protein of unknown function DUF1130 |
| 5869 | AGHZ01001254.1:1..5869 | Endonuclease/exonuclease/phosphatase family protein  Periplasmic thiol:disulfide interchange protein DsbA  TonB-dependent receptor |
| 5837 | AGHZ01000365.1:1..5837 | Fructose-bisphosphate aldolase class I (EC 4.1.2.13)  Metal-dependent phosphohydrolase2C HD subdomain  hypothetical protein  hypothetical protein  putative signal transduction histidine kinase  two component transcriptional regulator2C winged helix family |
| 5833 | AGHZ01000120.1:1..5833 | Glycerophosphoryl diester phosphodiesterase (EC 3.1.4.46)  Putative preQ0 transporter  TonB-dependent receptor  hypothetical protein |
| 5818 | AGHZ01000332.1:1..5818 | FIG01211164: hypothetical protein  GMP synthase [glutamine-hydrolyzing] (EC 6.3.5.2)  Hypothetical ABC transport system2C periplasmic component  Twin-arginine translocation protein TatC  glutamyl endopeptidase  hypothetical protein |
| 5804 | AGHZ01000020.1:1..5804 | 4-diphosphocytidyl-2-C-methyl-D-erythritol kinase (EC 2.7.1.148)  GTP-binding and nucleic acid-binding protein YchF  LSU ribosomal protein L25p  Outer membrane lipoprotein LolB precursor  Peptidyl-tRNA hydrolase (EC 3.1.1.29)  Ribose-phosphate pyrophosphokinase (EC 2.7.6.1)  tRNA-Gln-TTG  tRNA-Gly-TCC  tRNA-Thr-GGT  tRNA-Tyr-GTA |
| 5764 | AGHZ01000030.1:1..5764 | TdcF protein  TonB-dependent receptor  cytochrome C6  flavin monoamine oxidase-related protein  hypothetical protein |
| 5755 | AGHZ01000487.1:1..5755 | Acetyl-CoA synthetase (ADP-forming) alpha and beta chains2C putative  Glyoxalase family protein  hypothetical protein  hypothetical protein  hypothetical protein |
| 5753 | AGHZ01000386.1:1..5753 | ABC-type Na efflux pump2C permease component  ABC-type Na transport system2C ATPase component  FIG01211213: hypothetical protein  FIG032621: Hydrolase2C alpha/beta hydrolase fold family  Transcriptional regulator2C Cro/CI family |
| 5734 | AGHZ01000626.1:1..5734 | FIG01211086: hypothetical protein  FIG01217180: hypothetical protein  Ketoglutarate semialdehyde dehydrogenase (EC 1.2.1.26)  Predicted 4-hydroxyproline dipeptidase |
| 5725 | AGHZ01001688.1:1..5725 | FIG01210154: hypothetical protein  Glyoxalase family protein  Permeases of the major facilitator superfamily  Transcriptional regulator containing an amidase domain and an AraC-type DNA-binding HTH domain  Uroporphyrinogen III decarboxylase (EC 4.1.1.37)  hypothetical protein |
| 5724 | AGHZ01000032.1:1..5724 | LigA  Oxidoreductase (EC 1.1.1.-)  hypothetical protein  hypothetical protein  sensor histidine kinase |
| 5723 | AGHZ01000396.1:1..5723 | Molybdenum cofactor biosynthesis protein MoaB  TPR domain protein2C putative component of TonB system  Transcriptional regulator  hypothetical protein  hypothetical protein  hypothetical protein  thioredoxin |
| 5689 | AGHZ01000021.1:1..5689 | Alpha-amylase (EC 3.2.1.1)  Trehalose synthase (EC 5.4.99.16) |
| 5679 | AGHZ01000186.1:1..5679 | Dihydroneopterin aldolase (EC 4.1.2.25)  Protein containing domains DUF403  Protein containing domains DUF4042C DUF407  Small-conductance mechanosensitive channel  YgjD/Kae1/Qri7 family2C required for threonylcarbamoyladenosine (t(6)A) formation in tRNA |
| 5675 | AGHZ01000314.1:1..5675 | GCN5-related N-acetyltransferase  ferric enterobactin receptor  hypothetical protein  hypothetical protein |
| 5670 | AGHZ01000435.1:1..5670 | Glyoxalase family protein  Outer membrane protein romA  Transcriptional regulator2C TetR family  transcriptional regulator2C LysR family |
| 5660 | AGHZ01000209.1:1..5660 | Alanyl-tRNA synthetase (EC 6.1.1.7)  Carbon storage regulator  Glycogen debranching enzyme (EC 3.2.1.-)  hypothetical protein  hypothetical protein  hypothetical protein  hypothetical protein  putative; ORF located using Glimmer/Genemark  tRNA-Ser-GCT |
| 5659 | AGHZ01000011.1:1..5659 | 5S RNA  Large Subunit Ribosomal RNA; lsuRNA; LSU rRNA  Small Subunit Ribosomal RNA; ssuRNA; SSU rRNA  tRNA-Ala-TGC  tRNA-Ile-GAT |
| 5639 | AGHZ01000997.1:1..5639 | 2-hydroxy-6-oxo-6-phenylhexa-22C4-dienoate hydrolase (EC 3.7.1.-)  Aspartokinase (EC 2.7.2.4) / Homoserine dehydrogenase (EC 1.1.1.3)  Chitinase (EC 3.2.1.14)  hypothetical protein  response regulator receiver domain protein (CheY-like) |
| 5632 | AGHZ01000468.1:1..5632 | Acriflavin resistance protein  FIG01111895: hypothetical protein  membrane fusion protein |
| 5592 | AGHZ01000467.1:1..5592 | Cytochrome c family protein  Lysine-epsilon oxidase (EC 1.4.3.20) antimicrobial protein LodA  Transporter  hypothetical protein |
| 5574 | AGHZ01000220.1:1..5574 | Extracellular ribonuclease precursor (EC 3.1.-.-)  Heat shock protein 60 family chaperone GroEL  Heat shock protein 60 family co-chaperone GroES  Periplasmic divalent cation tolerance protein cutA |
| 5545 | AGHZ01000616.1:1..5545 | D-alanyl-D-alanine dipeptidase  FIG01210473: hypothetical protein  FIG01211119: hypothetical protein  FIG01211609: hypothetical protein  hydrolase haloacid delahogenase-like family  hypothetical protein  hypothetical protein |
| 5536 | AGHZ01001336.1:1..5536 | N-acetyl glucosamine transporter2C NagP  N-acetylglucosamine related transporter2C NagX  Predicted transcriptional regulator of N-Acetylglucosamine utilization2C GntR family  Predicted transcriptional regulator of N-Acetylglucosamine utilization2C LacI family  hypothetical protein |
| 5512 | AGHZ01000367.1:1..5512 | ATP synthase A chain (EC 3.6.3.14)  ATP synthase B chain (EC 3.6.3.14)  ATP synthase C chain (EC 3.6.3.14)  Dihydrolipoamide dehydrogenase of pyruvate dehydrogenase complex (EC 1.8.1.4)  FIG01210264: hypothetical protein  hypothetical protein  hypothetical protein |
| 5506 | AGHZ01000223.1:1..5506 | Alpha-12C2-mannosidase  Beta-galactosidase (EC 3.2.1.23)  FIG01210126: hypothetical protein |
| 5464 | AGHZ01000715.1:1..5464 | 2-amino-3-ketobutyrate coenzyme A ligase (EC 2.3.1.29)  Sulfate and thiosulfate binding protein CysP  Sulfate transport system permease protein CysT  Sulfate transport system permease protein CysW |
| 5447 | AGHZ01000189.1:1..5447 | Flavodoxins  Ribonucleotide reductase of class Ia (aerobic)2C alpha subunit (EC 1.17.4.1)  Ribonucleotide reductase of class Ia (aerobic)2C beta subunit (EC 1.17.4.1)  Thioredoxin  hypothetical protein |
| 5442 | AGHZ01000115.1:1..5442 | Acriflavin resistance protein  DNA-binding heavy metal response regulator  Probable Co/Zn/Cd efflux system membrane fusion protein |
| 5399 | AGHZ01000215.1:1..5399 | M. jannaschii predicted coding region MJ1233  Membrane protein2C putative  Transcriptional regulator  Uncharacterized protein with a chitinase homolog domain  hypothetical protein  hypothetical protein |
| 5392 | AGHZ01000135.1:1..5392 | hypothetical protein  putative; ORF located using Glimmer/Genemark  sensor kinase |
| 5385 | AGHZ01000347.1:1..5385 | Chromosome (plasmid) partitioning protein ParA / Sporulation initiation inhibitor protein Soj  Chromosome (plasmid) partitioning protein ParB / Stage 0 sporulation protein J  Dolichol-phosphate mannosyltransferase  FIG01209701: hypothetical protein  FIG01210656: hypothetical protein  Mitomycin resistance protein  dTDP-glucose 42C6-dehydratase (EC 4.2.1.46) |
| 5378 | AGHZ01000074.1:1..5378 | Arylesterase precursor (EC 3.1.1.2)  Response regulators consisting of a CheY-like receiver domain and a winged-helix DNA-binding domain  glycosyl hydrolase family 29 (alpha-L-fucosidase)  hypothetical protein  two-component system sensor protein |
| 5363 | AGHZ01001050.1:1..5363 | Carbonic anhydrase (EC 4.2.1.1)  FIG01111044: hypothetical protein  Phosphate ABC transporter2C periplasmic phosphate-binding protein PstS (TC 3.A.1.7.1)  Phosphate ABC transporter2C periplasmic phosphate-binding protein PstS (TC 3.A.1.7.1)  hypothetical protein  hypothetical protein |
| 5338 | AGHZ01000352.1:1..5338 | FIG01209931: hypothetical protein  GTP-binding protein Era  Ribonuclease III (EC 3.1.26.3)  Signal peptidase I (EC 3.4.21.89)  Translation elongation factor LepA |
| 5332 | AGHZ01000530.1:1..5332 | Beta-mannosidase (EC 3.2.1.25)  hypothetical protein |
| 5314 | AGHZ01001049.1:1..5314 | 4-hydroxy-3-methylbut-2-enyl diphosphate reductase (EC 1.17.1.2)  Isoleucyl-tRNA synthetase (EC 6.1.1.5)  Lipoprotein signal peptidase (EC 3.4.23.36)  Riboflavin kinase (EC 2.7.1.26) / FMN adenylyltransferase (EC 2.7.7.2)  hypothetical protein |
| 5296 | AGHZ01000169.1:1..5296 | Adenylate cyclase (EC 4.6.1.1)  FIG01113202: hypothetical protein  Sulfate permease  hypothetical protein  hypothetical protein |
| 5295 | AGHZ01000112.1:1..5295 | FIG00537880: hypothetical protein  Glycogen debranching enzyme (EC 3.2.1.-) |
| 5239 | AGHZ01000203.1:1..5239 | Fumarylacetoacetate hydrolase family protein  Maleylacetoacetate isomerase (EC 5.2.1.2) @ Glutathione S-transferase2C zeta (EC 2.5.1.18)  Type II secretion system protein-like protein |
| 5219 | AGHZ01000828.1:1..5219 | Chloride channel protein  FIG065221: Holliday junction DNA helicase  Protein crcB homolog  hypothetical protein  hypothetical protein |
| 5205 | AGHZ01001607.1:1..5205 | Acyl-CoA dehydrogenase (EC 1.3.99.3)  HNH endonuclease family protein  Nitrate/nitrite response regulator protein  hypothetical protein |
| 5180 | AGHZ01000559.1:1..5180 | ATPase2C AFG1 family  Alpha/beta hydrolase  NAD-dependent protein deacetylase of SIR2 family  Organic hydroperoxide resistance protein  Organic hydroperoxide resistance transcriptional regulator  Putative metal chaperone2C involved in Zn homeostasis2C GTPase of COG0523 family  hypothetical protein  integral membrane protein |
| 5179 | AGHZ01000604.1:1..5179 | Proline iminopeptidase (EC 3.4.11.5)  Protein-N(5)-glutamine methyltransferase PrmC2C methylates polypeptide chain release factors RF1 and RF2  Transcriptional regulator2C LysR family  Transglutaminase-like domain  hypothetical protein |
| 5144 | AGHZ01001627.1:1..5144 | DNA-binding response regulator  PTS system2C fructose-specific IIB component (EC 2.7.1.69) / PTS system2C fructose-specific IIC component (EC 2.7.1.69)  regulator of pathogenicity factors |
| 5132 | AGHZ01000403.1:1..5132 | Permeases of the major facilitator superfamily  hypothetical protein  hypothetical protein  oxidoreductase  phosphoesterase2C putative |
| 5126 | AGHZ01000883.1:1..5126 | 3-oxoacyl-[acyl-carrier protein] reductase (EC 1.1.1.100)  RND efflux system2C inner membrane transporter CmeB  RND efflux system2C membrane fusion protein CmeA  hypothetical protein |
| 5116 | AGHZ01000482.1:1..5116 | Ammonium transporter  Glutamine synthetase type I (EC 6.3.1.2)  Nitrogen regulatory protein P-II  Undecaprenyl-diphosphatase (EC 3.6.1.27)  hypothetical protein  hypothetical protein |
| 5114 | AGHZ01000793.1:1..5114 | FIG01212003: hypothetical protein  Membrane protease family protein BA0301  hypothetical protein  hypothetical protein  hypothetical protein  ring hydroxylating dioxygenase alpha-subunit |
| 5098 | AGHZ01000825.1:1..5098 | Aminopeptidase N  Cytosine deaminase (EC 3.5.4.1)  FIG01149443: hypothetical protein  Ubiquinone/menaquinone biosynthesis methyltransferase UbiE (EC 2.1.1.-)  hypothetical protein |
| 5081 | AGHZ01001533.1:1..5081 | COG2907: Amine oxidase2C flavin-containing  FIG001571: Hypothetical protein  Fatty acid desaturase (EC 1.14.19.1); Delta-9 fatty acid desaturase (EC 1.14.19.1)  S-adenosyl-L-methionine dependent methyltransferase2C similar to cyclopropane-fatty-acyl-phospholipid synthase |
| 5071 | AGHZ01000565.1:1..5071 | Penicillin-binding protein 2 (PBP-2)  Rod shape-determining protein MreC  Rod shape-determining protein MreD  Rod shape-determining protein RodA |
| 5068 | AGHZ01000497.1:1..5068 | Aldose 1-epimerase (EC 5.1.3.3)  Alpha-N-arabinofuranosidase 2 (EC 3.2.1.55)  Translation elongation factor G-related protein  hypothetical protein |
| 5063 | AGHZ01000281.1:1..5063 | Cytosol aminopeptidase PepA (EC 3.4.11.1)  DNA polymerase III chi subunit (EC 2.7.7.7)  Valyl-tRNA synthetase (EC 6.1.1.9) |
| 5041 | AGHZ01000212.1:1..5041 | 2-Keto-3-deoxy-D-manno-octulosonate-8-phosphate synthase (EC 2.5.1.55)  CTP synthase (EC 6.3.4.2)  Enolase (EC 4.2.1.11)  conserved hypothetical protein  hypothetical protein |
| 5017 | AGHZ01000058.1:1..5017 | tRNA-Leu-CAA  two-component system regulatory protein  two-component system sensor protein |
| 5007 | AGHZ01000784.1:1..5007 | A/G-specific adenine glycosylase (EC 3.2.2.-)  FIG001341: Probable Fe(2 )-trafficking protein YggX  FIG01111754: hypothetical protein  Signal recognition particle receptor protein FtsY (alpha subunit) (TC 3.A.5.1.1)  Zn-dependent protease with chaperone function PA4632  hypothetical protein  hypothetical protein  serine/threonine kinase |
| 5006 | AGHZ01000094.1:1..5006 | Catechol 12C2-dioxygenase (EC 1.13.11.1)  Membrane protein involved in aromatic hydrocarbon degradation  Muconate cycloisomerase (EC 5.5.1.1)  Muconolactone isomerase (EC 5.3.3.4)  salicylate esterase |
| 4960 | AGHZ01000663.1:1..4960 | FOG: Ankyrin repeat  Glutamate--cysteine ligase (EC 6.3.2.2)  PlcB2C ORFX2C ORFP2C ORFB2C ORFA2C ldh gene  hypothetical protein  hypothetical protein  two-component system sensor protein |
| 4953 | AGHZ01001081.1:1..4953 | Oligopeptide transporter  alanyl dipeptidyl peptidase  hypothetical protein |
| 4947 | AGHZ01000005.1:1..4947 | FIG01210787: hypothetical protein  TonB-dependent siderophore receptor  Transcriptional regulator protein  hypothetical protein  putative secreted protein |
| 4946 | AGHZ01000180.1:1..4946 | Cardiolipin synthetase (EC 2.7.8.-)  Radical SAM domain protein |
| 4936 | AGHZ01001690.1:1..4936 | COGs COG3737  Cell division protein FtsH (EC 3.4.24.-)  Cell division protein FtsJ / Ribosomal RNA large subunit methyltransferase E (EC 2.1.1.-)  FIG004454: RNA binding protein  Lipoprotein NlpD |
| 4922 | AGHZ01000301.1:1..4922 | AAA ATPase2C central region  FIG01211637: hypothetical protein  Phosphohistidine phosphatase SixA  partition protein |
| 4904 | AGHZ01000214.1:1..4904 | Aldehyde dehydrogenase (EC 1.2.1.3); Probable coniferyl aldehyde dehydrogenase (EC 1.2.1.68)  Exodeoxyribonuclease III (EC 3.1.11.2)  Gfa-like protein  HipA protein  HipB protein  transglycosylase associated protein |
| 4903 | AGHZ01000817.1:1..4903 | FIG01210885: hypothetical protein |
| 4883 | AGHZ01000432.1:1..4883 | Membrane-associated phospholipid phosphatase  Mlr6856 protein  TolA protein  Uncharacterized zinc-type alcohol dehydrogenase-like protein ybdR  hypothetical protein |
| 4869 | AGHZ01000179.1:1..4869 | FIG01210460: hypothetical protein  Indolepyruvate ferredoxin oxidoreductase2C alpha and beta subunits  Maebl  SAM-dependent methyltransferases  Staphylolytic protease preproenzyme LasA  hypothetical protein |
| 4868 | AGHZ01000938.1:1..4868 | FIG01209725: hypothetical protein  FIG01210483: hypothetical protein  Inner membrane protein  Sensor protein PhoQ (EC 2.7.13.3)  hypothetical protein |
| 4828 | AGHZ01000684.1:1..4828 | FIG01209976: hypothetical protein  L-threonine 3-dehydrogenase (EC 1.1.1.103)  hypothetical protein |
| 4825 | AGHZ01000104.1:1..4825 | FIG01211801: hypothetical protein  Nitrogen regulation protein NR(I)  amino acid transporter  hypothetical protein  hypothetical protein  hypothetical protein  two component sensor kinase |
| 4816 | AGHZ01000439.1:1..4816 | hypothetical protein |
| 4811 | AGHZ01000444.1:1..4811 | DUF819 domain-containing protein  accessory protein  hydrolase  phospholipase A1 |
| 4802 | AGHZ01000786.1:1..4802 | Diadenosine tetraphosphate (Ap4A) hydrolase and other HIT family hydrolases  Dienelactone hydrolase family  FIG01209679: hypothetical protein  Ribosomal protein S12p Asp88 (E. coli) methylthiotransferase  Transcription elongation factor GreB  hypothetical protein |
| 4795 | AGHZ01001413.1:1..4795 | Glycosyltransferase  hypothetical protein |
| 4782 | AGHZ01000164.1:1..4782 | FIG01212910: hypothetical protein  FIG01213330: hypothetical protein  Gluconolactonase  glucose-fructose oxidoreductase  hypothetical protein  hypothetical protein |
| 4756 | AGHZ01000758.1:1..4756 | Exopolyphosphatase (EC 3.6.1.11)  Phosphate regulon sensor protein PhoR (SphS) (EC 2.7.13.3)  Polyphosphate kinase (EC 2.7.4.1) |
| 4744 | AGHZ01001255.1:1..4744 | Chorismate synthase (EC 4.2.3.5)  Cytochrome oxidase biogenesis protein Sco1/SenC/PrrC2C putative copper metallochaperone  Phosphatidylserine decarboxylase (EC 4.1.1.65)  Protein-N(5)-glutamine methyltransferase PrmB2C methylates LSU ribosomal protein L3p  membrane-bound lytic murein transglycosylase D precursor |
| 4737 | AGHZ01000154.1:1..4737 | Stringent starvation protein A  Ubiquinol--cytochrome c reductase2C cytochrome B subunit (EC 1.10.2.2)  Ubiquinol-cytochrome C reductase iron-sulfur subunit (EC 1.10.2.2)  soluble lytic murein transglycosylase  ubiquinol cytochrome C oxidoreductase2C cytochrome C1 subunit |
| 4720 | AGHZ01001022.1:1..4720 | Intracellular PHB depolymerase (EC 3.1.1.-)  Transcriptional regulator2C PadR family  hypothetical protein  sensor protein |
| 4717 | AGHZ01000476.1:1..4717 | Aldehyde dehydrogenase (EC 1.2.1.3)  Nucleoside-diphosphate-sugar epimerases  Topoisomerase IV subunit A (EC 5.99.1.-)  hypothetical protein  probable DNA-binding protein  transcriptional regulator2C TetR family |
| 4717 | AGHZ01000369.1:1..4717 | FIG01213780: hypothetical protein  Succinate dehydrogenase cytochrome b-556 subunit  Succinate dehydrogenase flavoprotein subunit (EC 1.3.99.1)  Succinate dehydrogenase hydrophobic membrane anchor protein  Succinate dehydrogenase iron-sulfur protein (EC 1.3.99.1)  YgfY COG2938 |
| 4687 | AGHZ01000842.1:1..4687 | Alpha-12C2-mannosidase |
| 4649 | AGHZ01000446.1:1..4649 | N-acetylglucosamine-regulated TonB-dependent outer membrane receptor |
| 4647 | AGHZ01000648.1:1..4647 | 3-hydroxydecanoyl-[acyl-carrier-protein] dehydratase (EC 4.2.1.60)  3-oxoacyl-[acyl-carrier-protein] synthase2C KASI (EC 2.3.1.41)  DNA polymerase IV (EC 2.7.7.7)  Rrf2-linked NADH-flavin reductase  SH32C type 3 |
| 4642 | AGHZ01000318.1:1..4642 | Outer membrane protein Imp2C required for envelope biogenesis / Organic solvent tolerance protein precursor  Survival protein SurA precursor (Peptidyl-prolyl cis-trans isomerase SurA) (EC 5.2.1.8)  acetoin utilization family protein |
| 4638 | AGHZ01000137.1:1..4638 | Isovaleryl-CoA dehydrogenase (EC 1.3.99.10)  Methylcrotonyl-CoA carboxylase carboxyl transferase subunit (EC 6.4.1.4)  hypothetical protein |
| 4624 | AGHZ01000422.1:1..4624 | FIG01211097: hypothetical protein  INTEGRAL MEMBRANE PROTEIN (Rhomboid family)  Long-chain fatty acid transport protein  Proline dehydrogenase (EC 1.5.99.8) (Proline oxidase) / Delta-1-pyrroline-5-carboxylate dehydrogenase (EC 1.5.1.12) |
| 4609 | AGHZ01001651.1:1..4609 | FIG01211029: hypothetical protein  L-proline glycine betaine ABC transport system permease protein ProV (TC 3.A.1.12.1)  Probable component of the lipoprotein assembly complex (forms a complex with YaeT2C YfgL2C and NlpB)  hypothetical protein |
| 4607 | AGHZ01000234.1:1..4607 | CoA tranferase  hypothetical protein  putative RecF protein |
| 4602 | AGHZ01000523.1:1..4602 | TonB-dependent receptor  Transcriptional repressor2C BlaI/MecI family  hypothetical protein  hypothetical protein |
| 4573 | AGHZ01000271.1:1..4573 | Beta-galactosidase (EC 3.2.1.23)  LysR family transcriptional regulator YeiE  TonB-dependent receptor  hypothetical protein |
| 4567 | AGHZ01000876.1:1..4567 | FIG01211032: hypothetical protein  Redox-sensing transcriptional regulator QorR  hypothetical protein  outer membrane lipoprotein  protein of unknown function DUF1486  putative salt-induced outer membrane protein |
| 4545 | AGHZ01000411.1:1..4545 | 3-hydroxyanthranilate 32C4-dioxygenase (EC 1.13.11.6)  Carbonic anhydrase (EC 4.2.1.1)  FIG01212272: hypothetical protein  SECRETION ACTIVATOR PROTEIN  hypothetical protein  hypothetical protein  hypothetical protein  hypothetical protein |
| 4532 | AGHZ01001534.1:1..4532 | 2-ketoglutaric semialdehyde dehydrogenase (EC 1.2.1.26)  Putative sugar ABC transport system2C periplasmic binding protein YtfQ precursor  SUGAR TRANSPORTER |
| 4519 | AGHZ01001030.1:1..4519 | ABC-type phosphate/phosphonate transport system periplasmic component  Aerotaxis sensor receptor protein  Arginine-tRNA-protein transferase (EC 2.3.2.8)  Roadblock/LC7 family protein  hypothetical protein |
| 4518 | AGHZ01001603.1:1..4518 | ABC-type antimicrobial peptide transport system2C permease component  FIG01210546: hypothetical protein  Permease of the drug/metabolite transporter (DMT) superfamily  RarD protein2C chloamphenicol sensitive  hypothetical protein |
| 4511 | AGHZ01000582.1:1..4511 | Isocitrate lyase (EC 4.1.3.1)  Malate synthase (EC 2.3.3.9)  cAMP-binding proteins - catabolite gene activator and regulatory subunit of cAMP-dependent protein kinases  hypothetical protein  hypothetical protein |
| 4511 | AGHZ01000540.1:1..4511 | Na /H exchange protein  TonB-dependent receptor |
| 4499 | AGHZ01000507.1:1..4499 | Dipeptidyl carboxypeptidase Dcp (EC 3.4.15.5)  Ferredoxin--NADP( ) reductase (EC 1.18.1.2)  Glutathione peroxidase (EC 1.11.1.9)  hypothetical protein  hypothetical protein |
| 4494 | AGHZ01000572.1:1..4494 | General secretion pathway protein D / Type II secretion outermembrane pore forming protein (PulD)  General secretion pathway protein N |
| 4490 | AGHZ01000142.1:1..4490 | 5'-nucleotidase YjjG (EC 3.1.3.5)  Acetate permease ActP (cation/acetate symporter)  FIG01111534: hypothetical protein  Putative membrane protein2C clustering with ActP |
| 4482 | AGHZ01000401.1:1..4482 | Putative large exoprotein involved in heme utilization or adhesion of ShlA/HecA/FhaA family |
| 4453 | AGHZ01000228.1:1..4453 | COG1451: Predicted metal-dependent hydrolase  EAL domain protein  FIG01210445: hypothetical protein  Hypothetical Zinc-finger containing protein  Small-conductance mechanosensitive channel  nucleoprotein/polynucleotide-associated enzyme  tRNA pseudouridine synthase A (EC 4.2.1.70) |
| 4452 | AGHZ01000141.1:1..4452 | FIG01210123: hypothetical protein  Outer membrane lipoprotein Blc  hypothetical protein  hypothetical protein  tRNA-Phe-GAA  transcriptional regulator marR family |
| 4447 | AGHZ01000093.1:1..4447 | FrmR: Negative transcriptional regulator of formaldehyde detoxification operon  Glutathione-dependent formaldehyde-activating enzyme (EC 4.4.1.22)  S-(hydroxymethyl)glutathione dehydrogenase (EC 1.1.1.284)  S-formylglutathione hydrolase (EC 3.1.2.12)  two-component system regulatory protein |
| 4442 | AGHZ01000224.1:1..4442 | Plasmid maintenance system antidote protein |
| 4406 | AGHZ01000707.1:1..4406 | Dihydrodipicolinate synthase (EC 4.2.1.52)  FIG01209675: hypothetical protein  Glycine cleavage system transcriptional antiactivator GcvR  Predicted ATPase related to phosphate starvation-inducible protein PhoH  Thiol peroxidase2C Bcp-type (EC 1.11.1.15) |
| 4394 | AGHZ01000144.1:1..4394 | Chaperone protein HtpG |
| 4393 | AGHZ01001315.1:1..4393 | Bifunctional protein: zinc-containing alcohol dehydrogenase; quinone oxidoreductase ( NADPH:quinone reductase) (EC 1.1.1.-); Similar to arginate lyase  COG2755: Lysophospholipase L1 and related esterases  Oxidoreductase  hypothetical protein |
| 4374 | AGHZ01000048.1:1..4374 | Proton/glutamate symport protein @ Sodium/glutamate symport protein  Transketolase (EC 2.2.1.1)  hypothetical protein |
| 4372 | AGHZ01000541.1:1..4372 | Cellulase  FIG01112224: hypothetical protein  POSSIBLE CONSERVED MEMBRANE PROTEIN  Similarity with glutathionylspermidine synthase (EC 6.3.1.8)2C group 1  hypothetical protein |
| 4367 | AGHZ01000488.1:1..4367 | Type IV pilus biogenesis protein PilO  Type IV pilus biogenesis protein PilP  Type IV pilus biogenesis protein PilQ |
| 4336 | AGHZ01000185.1:1..4336 | hypothetical protein  hypothetical protein  hypothetical protein  possible xylosidase/arabinosidase  putative secreted protein |
| 4336 | AGHZ01000001.1:1..4336 | Methyl-accepting chemotaxis protein  NAD-dependent glyceraldehyde-3-phosphate dehydrogenase (EC 1.2.1.12)  Outer membrane protein W precursor  hypothetical protein |
| 4317 | AGHZ01000400.1:1..4317 | Dihydroxy-acid dehydratase (EC 4.2.1.9)  hypothetical protein  site-specific recombinase |
| 4297 | AGHZ01000789.1:1..4297 | Acetyltransferase  Acetyltransferase (isoleucine patch superfamily)  GbcA Glycine betaine demethylase subunit A  hypothetical protein  hypothetical protein |
| 4291 | AGHZ01000290.1:1..4291 | Transcription-repair coupling factor  hypothetical protein |
| 4281 | AGHZ01001495.1:1..4281 | FIG01211317: hypothetical protein  Glutaredoxin-like protein  acetylxylan esterase  hypothetical protein  hypothetical protein |
| 4278 | AGHZ01000773.1:1..4278 | Histone acetyltransferase HPA2 and related acetyltransferases  Protein involved in catabolism of external DNA  hypothetical protein |
| 4274 | AGHZ01000328.1:1..4274 | FIG01211818: hypothetical protein  Two-component system regulatory protein  tRNA dihydrouridine synthase A |
| 4256 | AGHZ01000173.1:1..4256 | Large exoproteins involved in heme utilization or adhesion |
| 4254 | AGHZ01001194.1:1..4254 | Alpha2Calpha-trehalose-phosphate synthase [UDP-forming] (EC 2.4.1.15)  Glucoamylase (EC 3.2.1.3)  Trehalose-6-phosphate phosphatase (EC 3.1.3.12) |
| 4251 | AGHZ01000637.1:1..4251 | FIG01210342: hypothetical protein  Flavodoxin reductases (ferredoxin-NADPH reductases) family 1; Vanillate O-demethylase oxidoreductase (EC 1.14.13.-)  P-hydroxybenzoate hydroxylase (EC 1.14.13.2)  transcriptional regulator2C GntR family |
| 4236 | AGHZ01001568.1:1..4236 | AmpG permease  Anhydro-N-acetylmuramic acid kinase (EC 2.7.1.-)  Exodeoxyribonuclease III (EC 3.1.11.2)  Exonuclease SbcC  Orotate phosphoribosyltransferase (EC 2.4.2.10)  hypothetical protein |
| 4219 | AGHZ01000696.1:1..4219 | Cytochrome c oxidase polypeptide I (EC 1.9.3.1)  Cytochrome c oxidase polypeptide III (EC 1.9.3.1)  Cytochrome oxidase biogenesis protein Cox11-CtaG2C copper delivery to Cox1  FIG01210764: hypothetical protein  FIG01212439: hypothetical protein |
| 4218 | AGHZ01000820.1:1..4218 | 4-amino-4-deoxy-L-arabinose transferase and related glycosyltransferases of PMT family  FIG01214694: hypothetical protein |
| 4217 | AGHZ01000394.1:1..4217 | Transcriptional regulator lysR family  Transporter2C LysE family  hypothetical protein  tRNA-Lys-TTT  transcriptional regulator uid family |
| 4202 | AGHZ01000152.1:1..4202 | FIG01211359: hypothetical protein  Heme A synthase2C cytochrome oxidase biogenesis protein Cox15-CtaA  Heme O synthase2C protoheme IX farnesyltransferase (EC 2.5.1.-) COX10-CtaB  methyl-accepting chemotaxis protein2C putative |
| 4194 | AGHZ01000007.1:1..4194 | Aminomethyltransferase (glycine cleavage system T protein) (EC 2.1.2.10)  Glycine cleavage system H protein  histone H1 |
| 4162 | AGHZ01000538.1:1..4162 | FIG01213947: hypothetical protein  TonB-dependent receptor |
| 4152 | AGHZ01001034.1:1..4152 | DNA-directed RNA polymerase omega subunit (EC 2.7.7.6)  GTP pyrophosphokinase (EC 2.7.6.5)2C (p)ppGpp synthetase II / Guanosine-3'2C5'-bis(diphosphate) 3'-pyrophosphohydrolase (EC 3.1.7.2)  Guanylate kinase (EC 2.7.4.8)  Protein YicC |
| 4147 | AGHZ01000502.1:1..4147 | Carbamoyl-phosphate synthase large chain (EC 6.3.5.5)  FIG01209712: hypothetical protein  Transcription elongation factor GreA |
| 4139 | AGHZ01000857.1:1..4139 | FIG016519: Putative DNA-binding protein  GTP-binding protein EngA  Mlr7403 protein  Outer membrane protein YfgL2C lipoprotein component of the protein assembly complex (forms a complex with YaeT2C YfiO2C and NlpB) |
| 4138 | AGHZ01000689.1:1..4138 | Flagellar hook-associated protein FlgK  Flagellar hook-associated protein FlgL  Flagellar protein FlgJ [peptidoglycan hydrolase] (EC 3.2.1.-) |
| 4138 | AGHZ01000640.1:1..4138 | Na( ) H( ) antiporter subunit A; Na( ) H( ) antiporter subunit B  Na( ) H( ) antiporter subunit C |
| 4122 | AGHZ01000311.1:1..4122 | Protein-L-isoaspartate O-methyltransferase (EC 2.1.1.77)  Transcriptional regulator2C TetR family  Type I secretion outer membrane protein2C TolC precursor  hypothetical protein  putative thermostable hemolysin |
| 4120 | AGHZ01000100.1:1..4120 | 3-demethylubiquinone-9 3-methyltransferase (EC 2.1.1.64)  S-adenosylhomocysteine deaminase (EC 3.5.4.28); Methylthioadenosine deaminase  Similar to phosphoglycolate phosphatase2C clustered with ubiquinone biosynthesis SAM-dependent O-methyltransferase  hypothetical protein |
| 4117 | AGHZ01000670.1:1..4117 | Histidyl-tRNA synthetase (EC 6.1.1.21)  hypothetical protein  hypothetical protein  transcriptional regulator2C Crp/Fnr family |
| 4114 | AGHZ01000344.1:1..4114 | Glycerophosphoryl diester phosphodiesterase (EC 3.1.4.46)  TonB-dependent receptor |
| 4090 | AGHZ01000515.1:1..4090 | FIG01209842: hypothetical protein  Lipoprotein releasing system ATP-binding protein LolD  Lipoprotein releasing system transmembrane protein LolC  Nucleoside-diphosphate-sugar epimerases  hypothetical protein |
| 4085 | AGHZ01000714.1:1..4085 | Methyl-accepting chemotaxis protein I (serine chemoreceptor protein)  Preprotein translocase subunit YajC (TC 3.A.5.1.1)  Protein-export membrane protein SecD (TC 3.A.5.1.1)  Protein-export membrane protein SecF (TC 3.A.5.1.1) |
| 4083 | AGHZ01000159.1:1..4083 | Coproporphyrinogen III oxidase2C aerobic (EC 1.3.3.3)  DNA polymerase I (EC 2.7.7.7)  hypothetical protein  hypothetical protein |
| 4082 | AGHZ01000830.1:1..4082 | 2-methylcitrate dehydratase FeS dependent (EC 4.2.1.79)  hypothetical protein |
| 4078 | AGHZ01000296.1:1..4078 | Ferric siderophore transport system2C periplasmic binding protein TonB  NfuA Fe-S protein maturation  Pterin-4-alpha-carbinolamine dehydratase (EC 4.2.1.96)  Zinc transporter ZupT |
| 4076 | AGHZ01000291.1:1..4076 | Cell division protein FtsL  Cell division protein MraZ  rRNA small subunit methyltransferase H |
| 4074 | AGHZ01000199.1:1..4074 | Sensory box/GGDEF family protein  hypothetical protein |
| 4055 | AGHZ01000824.1:1..4055 | hydrolase2C haloacid dehalogenase-like family  hypothetical protein  hypothetical protein  hypothetical protein |
| 4051 | AGHZ01000412.1:1..4051 | DNA topoisomerase I (EC 5.99.1.2)  FIG01210152: hypothetical protein |
| 4039 | AGHZ01000283.1:1..4039 | Chemotaxis regulator - transmits chemoreceptor signals to flagelllar motor components CheY  Chemotaxis response - phosphatase CheZ  Flagellar biosynthesis protein FlhF  Flagellar synthesis regulator FleN  RNA polymerase sigma factor for flagellar operon |
| 4027 | AGHZ01000521.1:1..4027 | Phosphoribosylformylglycinamidine synthase2C synthetase subunit (EC 6.3.5.3) / Phosphoribosylformylglycinamidine synthase2C glutamine amidotransferase subunit (EC 6.3.5.3)  hypothetical protein |
| 4019 | AGHZ01000341.1:1..4019 | Aspartyl-tRNA synthetase (EC 6.1.1.12)  acetyltransferase  conserved hypothetical protein  hypothetical protein |
| 4000 | AGHZ01001270.1:1..4000 | Beta-lactamase (EC 3.5.2.6)  Phospholipase C 4 precursor (EC 3.1.4.3)  Xylanase  hypothetical protein |
| 4000 | AGHZ01000678.1:1..4000 | ADP-heptose--lipooligosaccharide heptosyltransferase II (EC 2.4.1.-)  FIG01209666: hypothetical protein  hypothetical protein  hypothetical protein  putative rhamnogalacturonase B precursor  tRNA-Ser-CGA |
| 3987 | AGHZ01000438.1:1..3987 | Di-/tripeptide transporter  FIG01210281: hypothetical protein  Tryptophan 22C3-dioxygenase (EC 1.13.11.11) |
| 3984 | AGHZ01000051.1:1..3984 | hypothetical protein |
| 3936 | AGHZ01001464.1:1..3936 | 5'-methylthioadenosine phosphorylase (EC 2.4.2.28)  Beta N-acetyl-glucosaminidase (EC 3.2.1.52)  Cold shock protein CspD  Hypoxanthine-guanine phosphoribosyltransferase (EC 2.4.2.8) |
| 3935 | AGHZ01000225.1:1..3935 | 3-beta hydroxysteroid dehydrogenase/isomerase family protein in hypothetical gene cluster  hypothetical protein  prolyl oligopeptidase family protein |
| 3933 | AGHZ01001511.1:1..3933 | NAD-dependent protein deacetylase of SIR2 family  Permeases of the major facilitator superfamily  biphenyl-22C3-diol 12C2-dioxygenase III-related protein |
| 3931 | AGHZ01001598.1:1..3931 | COG0488: ATPase components of ABC transporters with duplicated ATPase domains  FIG01211504: hypothetical protein  hypothetical protein  hypothetical protein |
| 3926 | AGHZ01001443.1:1..3926 | FIG01210004: hypothetical protein  Magnesium transporter  hypothetical protein  hypothetical protein |
| 3926 | AGHZ01000097.1:1..3926 | Biopolymer transport protein ExbD/TolR  Biopolymer transport protein ExbD/TolR  Biopolymer transport protein ExbD/TolR  Ferric siderophore transport system2C periplasmic binding protein TonB  MotA/TolQ/ExbB proton channel family protein  Pyridoxine 5'-phosphate synthase (EC 2.6.99.2) |
| 3915 | AGHZ01000510.1:1..3915 | FecR protein  TonB-dependent receptor |
| 3880 | AGHZ01001035.1:1..3880 | Glyoxalase family protein  Nucleoside 5-triphosphatase RdgB (dHAPTP2C dITP2C XTP-specific) (EC 3.6.1.15)  Radical SAM family enzyme2C similar to coproporphyrinogen III oxidase2C oxygen-independent2C clustered with nucleoside-triphosphatase RdgB  Ribonuclease PH (EC 2.7.7.56) |
| 3873 | AGHZ01000387.1:1..3873 | Aspartokinase (EC 2.7.2.4) / Diaminopimelate decarboxylase (EC 4.1.1.20)  hypothetical protein |
| 3862 | AGHZ01000501.1:1..3862 | hypothetical protein |
| 3847 | AGHZ01000298.1:1..3847 | ADP-ribosylglycohydrolase family protein  Inactive homolog of metal-dependent proteases2C putative molecular chaperone  TonB-dependent receptor |
| 3840 | AGHZ01000627.1:1..3840 | Glucoamylase (EC 3.2.1.3) |
| 3833 | AGHZ01000349.1:1..3833 |  |
| 3828 | AGHZ01000642.1:1..3828 | DNA primase (EC 2.7.7.-)  Sodium - Bile acid symporter  hypothetical protein |
| 3821 | AGHZ01000068.1:1..3821 | FIG01212049: hypothetical protein  Formate dehydrogenase chain D (EC 1.2.1.2)  Putative formate dehydrogenase oxidoreductase protein |
| 3803 | AGHZ01000958.1:1..3803 | Conserved hypothetical protein (perhaps related to histidine degradation)  DNA transport competence protein  FIG01210990: hypothetical protein  succinyl-diaminopimelate desuccinylase |
| 3803 | AGHZ01000791.1:1..3803 | Cytochrome d ubiquinol oxidase subunit II (EC 1.10.3.-)  Glutaryl-7-ACA acylase  hypothetical protein  hypothetical protein  hypothetical protein  tRNA-Pro-CGG |
| 3791 | AGHZ01001387.1:1..3791 | COG1801: Uncharacterized conserved protein  DNA-3-methyladenine glycosylase (EC 3.2.2.20)  FAD dependent oxidoreductase  FIG01209760: hypothetical protein |
| 3781 | AGHZ01000244.1:1..3781 | RND efflux system2C inner membrane transporter CmeB |
| 3774 | AGHZ01000360.1:1..3774 | FIG01210275: hypothetical protein  hypothetical protein  hypothetical protein |
| 3772 | AGHZ01000127.1:1..3772 | Fusaric acid resistance protein fusE  SprB |
| 3762 | AGHZ01000117.1:1..3762 | Ribonuclease BN (EC 3.1.-.-)  SSU ribosomal protein S21p  Transamidase GatB domain protein  hypothetical protein |
| 3756 | AGHZ01001023.1:1..3756 | Permease of the drug/metabolite transporter (DMT) superfamily  hypothetical protein  peptidyl-prolyl cis-trans isomerase  transcriptional regulator |
| 3741 | AGHZ01000434.1:1..3741 | 5-formyltetrahydrofolate cyclo-ligase (EC 6.3.3.2)  FIG01210448: hypothetical protein  Protein of unknown function DUF55  Protein sirB1  Ribose 5-phosphate isomerase A (EC 5.3.1.6)  Rubredoxin |
| 3738 | AGHZ01001028.1:1..3738 | Cell division protein FtsK  Thioredoxin reductase (EC 1.8.1.9) |
| 3731 | AGHZ01000240.1:1..3731 | Flagellar biosynthesis protein FlhA  Flagellar biosynthesis protein FlhB |
| 3723 | AGHZ01000380.1:1..3723 | Alkylphosphonate utilization operon protein PhnA  MFS transporter  N-ethylmaleimide reductase (EC 1.-.-.-) |
| 3713 | AGHZ01001164.1:1..3713 | DNA gyrase subunit A (EC 5.99.1.3) |
| 3710 | AGHZ01000414.1:1..3710 | Nuclease  Putative metal chaperone2C involved in Zn homeostasis2C GTPase of COG0523 family  hypothetical protein |
| 3708 | AGHZ01001072.1:1..3708 | hypothetical protein |
| 3695 | AGHZ01000473.1:1..3695 | 1-acyl-sn-glycerol-3-phosphate acyltransferase (EC 2.3.1.51)  Interferon-induced transmembrane protein  Interferon-induced transmembrane protein  Transmembrane protein  conserved hypothetical protein |
| 3679 | AGHZ01000258.1:1..3679 | D-mannose isomerase (EC 5.3.1.7)  Predicted mannose transporter2C GGP family  RNA polymerase sigma factor RpoD  hypothetical protein |
| 3670 | AGHZ01000458.1:1..3670 | Phosphoglycerate kinase (EC 2.7.2.3)  Phosphoglycolate phosphatase (EC 3.1.3.18) |
| 3664 | AGHZ01000124.1:1..3664 | FecR protein  TonB-dependent ferrichrome-iron receptor  tRNA pseudouridine 13 synthase (EC 4.2.1.-) |
| 3656 | AGHZ01000056.1:1..3656 | FIG01210050: hypothetical protein  Glutaminyl-tRNA synthetase (EC 6.1.1.18)  two-component system sensor protein  two-component system sensor protein |
| 3632 | AGHZ01000192.1:1..3632 | ATP-dependent DNA helicase RecQ  Non-specific DNA-binding protein Dps / Iron-binding ferritin-like antioxidant protein / Ferroxidase (EC 1.16.3.1)  hypothetical protein |
| 3620 | AGHZ01000764.1:1..3620 | FIG01211497: hypothetical protein  Glyoxalase/Bleomycin resistance protein/Dioxygenase family protein  Permease  hypothetical protein |
| 3620 | AGHZ01000669.1:1..3620 | Predicted Zn-dependent peptidases |
| 3619 | AGHZ01000217.1:1..3619 | Seryl-tRNA synthetase (EC 6.1.1.11)  Transcriptional regulator2C LysR family  hypothetical protein  tRNA-Ser-TGA |
| 3597 | AGHZ01000577.1:1..3597 | Benzoate transport protein  FIG01211439: hypothetical protein  Phytochrome2C two-component sensor histidine kinase (EC 2.7.3.-) |
| 3596 | AGHZ01001542.1:1..3596 | FIG01211262: hypothetical protein  PROBABLE THIAMINE BIOSYNTHESIS LIPOPROTEIN APBE PRECURSOR TRANSMEMBRANE  PUTATIVE SIGNAL PEPTIDE PROTEIN  hypothetical secreted protein |
| 3588 | AGHZ01000505.1:1..3588 | putative integrase |
| 3588 | AGHZ01000455.1:1..3588 | Aromatic hydrocarbon utilization transcriptional regulator CatR (LysR family)  FIG01210755: hypothetical protein  hypothetical protein |
| 3561 | AGHZ01000886.1:1..3561 | TonB-dependent receptor  hypothetical protein  hypothetical protein |
| 3547 | AGHZ01000989.1:1..3547 | Cold shock protein CspG  FIG01212380: hypothetical protein  HesA/MoeB/ThiF family protein  Peptidoglycan-associated outer membrane lipoprotein  probable membrane protein NMA1128 |
| 3543 | AGHZ01000385.1:1..3543 | Putative inner membrane protein  Transcriptional regulator2C AsnC family  carbonic anhydrase2C family 3  hypothetical protein |
| 3541 | AGHZ01000247.1:1..3541 | FIG01210190: hypothetical protein  TonB-dependent receptor  short chain dehydrogenase |
| 3540 | AGHZ01000456.1:1..3540 | Glycerol kinase (EC 2.7.1.30)  Glycerol uptake facilitator protein  hypothetical protein |
| 3539 | AGHZ01000558.1:1..3539 | Leucyl-tRNA synthetase (EC 6.1.1.4)  hypothetical protein |
| 3535 | AGHZ01000374.1:1..3535 | Cobalt-zinc-cadmium resistance protein CzcA; Cation efflux system protein CusA  FIG01113058: hypothetical protein |
| 3524 | AGHZ01001675.1:1..3524 |  |
| 3522 | AGHZ01000988.1:1..3522 | Alkaline phosphatase (EC 3.1.3.1)  Proton/glutamate symport protein @ Sodium/glutamate symport protein |
| 3519 | AGHZ01000050.1:1..3519 | Alcohol dehydrogenase (EC 1.1.1.1)  Aldehyde dehydrogenase (EC 1.2.1.3)  FIG01210906: hypothetical protein |
| 3504 | AGHZ01000668.1:1..3504 | hypothetical protein |
| 3492 | AGHZ01000261.1:1..3492 | Beta-lactamase related protein  hypothetical protein  hypothetical protein  hypothetical protein |
| 3485 | AGHZ01000418.1:1..3485 | Diaminohydroxyphosphoribosylaminopyrimidine deaminase (EC 3.5.4.26) / 5-amino-6-(5-phosphoribosylamino)uracil reductase (EC 1.1.1.193)  Riboflavin synthase alpha chain (EC 2.5.1.9)  hypothetical protein |
| 3480 | AGHZ01000195.1:1..3480 |  |
| 3479 | AGHZ01000589.1:1..3479 | Glutathione synthetase (EC 6.3.2.3)  twitching motility protein PilG  twitching motility protein PilH  type IV pili signal transduction protein PilI |
| 3473 | AGHZ01000693.1:1..3473 | 3-isopropylmalate dehydratase small subunit (EC 4.2.1.33)  3-isopropylmalate dehydrogenase (EC 1.1.1.85)  hypothetical protein |
| 3465 | AGHZ01000921.1:1..3465 | Methyl-accepting chemotaxis protein I (serine chemoreceptor protein)  Zinc metalloprotease (EC 3.4.24.-) |
| 3458 | AGHZ01001168.1:1..3458 | Acetylornithine aminotransferase (EC 2.6.1.11)  Lactoylglutathione lyase (EC 4.4.1.5)  Potassium voltage-gated channel subfamily KQT; possible potassium channel2C VIC family |
| 3457 | AGHZ01000914.1:1..3457 | Bacterioferritin  FIG01211244: hypothetical protein  hypothetical protein  putative; ORF located using Glimmer/Genemark |
| 3456 | AGHZ01000361.1:1..3456 | Cobalt-zinc-cadmium resistance protein CzcD  Putative membrane protein YeiH  hypothetical protein |
| 3454 | AGHZ01001626.1:1..3454 | N-acetylglucosamine-6-phosphate deacetylase (EC 3.5.1.25)  Peptidase M23B  Ribulosamine/erythrulosamine 3-kinase potentially involved in protein deglycation  hypothetical protein |
| 3445 | AGHZ01000126.1:1..3445 | Permease of the drug/metabolite transporter (DMT) superfamily  sensor histidine kinase  tRNA pseudouridine synthase A (EC 4.2.1.70) |
| 3443 | AGHZ01000319.1:1..3443 | Alcohol dehydrogenase (EC 1.1.1.1)  hypothetical protein  peptidase M28 |
| 3436 | AGHZ01000174.1:1..3436 | 3-hydroxyisobutyrate dehydrogenase and related beta-hydroxyacid dehydrogenases  Conditioned medium factor  FIG01211006: hypothetical protein  Formyltetrahydrofolate deformylase (EC 3.5.1.10) |
| 3419 | AGHZ01000759.1:1..3419 | COG1272: Predicted membrane protein hemolysin III homolog  Hemolysin III |
| 3411 | AGHZ01000306.1:1..3411 | FIG01210053: hypothetical protein  Molybdenum cofactor biosynthesis protein MoaA  Molybdenum cofactor biosynthesis protein MoaC  Molybdenum cofactor biosynthesis protein MoaD  Molybdenum cofactor biosynthesis protein MoaE  tRNA-Ser-GGA |
| 3411 | AGHZ01000059.1:1..3411 | hypothetical protein |
| 3398 | AGHZ01001171.1:1..3398 | GGDEF family protein  Ribonuclease T (EC 3.1.13.-)  hypothetical protein  hypothetical protein  hypothetical protein |
| 3389 | AGHZ01000583.1:1..3389 | Distant similarity with leukotriene C4 synthase (microsomal glutathione S-transferase)  FIG000906: Predicted Permease  FIG000988: Predicted permease |
| 3388 | AGHZ01000096.1:1..3388 | Acetyltransferase  Polysaccharide deacetylase  hypothetical protein |
| 3387 | AGHZ01000494.1:1..3387 | TonB-dependent receptor |
| 3386 | AGHZ01001350.1:1..3386 | 3-oxoacyl-[acyl-carrier protein] reductase (EC 1.1.1.100)  Rhodanese-related sulfurtransferase  hypothetical protein  tRNA-Arg-ACG  tRNA-Arg-ACG  transcriptional regulator |
| 3382 | AGHZ01000029.1:1..3382 | COG2363  FIG01209965: hypothetical protein  hypothetical protein  two-component system regulatory protein |
| 3379 | AGHZ01000619.1:1..3379 | Beta-glucosidase (EC 3.2.1.21)  FIG01209726: hypothetical protein  FIG01209890: hypothetical protein  PQQ-dependent oxidoreductase2C gdhB family |
| 3363 | AGHZ01000506.1:1..3363 | Beta-glucosidase (EC 3.2.1.21)  Xylosidase/arabinosidase |
| 3360 | AGHZ01000509.1:1..3360 | Membrane proteins related to metalloendopeptidases  Tyrosyl-tRNA synthetase (EC 6.1.1.1) |
| 3331 | AGHZ01000544.1:1..3331 | ABC transporter-related protein  PUTATIVE TRANSMEMBRANE PROTEIN  Ribose ABC transport system2C permease protein RbsC (TC 3.A.1.2.1)  inner-membrane translocator |
| 3307 | AGHZ01001082.1:1..3307 | Broad-specificity glycerol dehydrogenase (EC 1.1.99.22)2C subunit SldA  hypothetical protein  putative tetratricopeptide repeat family protein |
| 3290 | AGHZ01000547.1:1..3290 | Acriflavin resistance protein  LigA |
| 3279 | AGHZ01000555.1:1..3279 | Branched-chain amino acid aminotransferase (EC 2.6.1.42)  FIG01210375: hypothetical protein  probably aromatic ring hydroxylating enzyme2C evidenced by COGnitor; PaaD-like protein (DUF59) involved in Fe-S cluster assembly |
| 3279 | AGHZ01000232.1:1..3279 | Enoyl-CoA hydratase (EC 4.2.1.17) / 3-hydroxyacyl-CoA dehydrogenase (EC 1.1.1.35) / 3-hydroxybutyryl-CoA epimerase (EC 5.1.2.3)  RNA polymerase sigma factor RpoE |
| 3277 | AGHZ01000728.1:1..3277 | Outer membrane receptor proteins2C mostly Fe transport  Phospholipase A1 precursor (EC 3.1.1.322C EC 3.1.1.4); Outer membrane phospholipase A  hypothetical protein  hypothetical protein |
| 3277 | AGHZ01000213.1:1..3277 | probable oxidoreductase/Short-chain dehydrogenase |
| 3276 | AGHZ01000140.1:1..3276 | Lipase  RND efflux system2C outer membrane lipoprotein CmeC  transcriptional regulator2C AraC family |
| 3273 | AGHZ01001635.1:1..3273 | Gamma-glutamyltranspeptidase (EC 2.3.2.2)  Heavy metal sensor histidine kinase  Zn-dependent hydrolases2C including glyoxylases |
| 3263 | AGHZ01000308.1:1..3263 | FIG01212614: hypothetical protein  hypothetical protein |
| 3262 | AGHZ01000166.1:1..3262 | Branched-chain alpha-keto acid dehydrogenase2C E1 component2C alpha subunit (EC 1.2.4.4)  Branched-chain alpha-keto acid dehydrogenase2C E1 component2C beta subunit (EC 1.2.4.4)  FIG01209918: hypothetical protein |
| 3257 | AGHZ01000785.1:1..3257 | Ribosomal large subunit pseudouridine synthase C (EC 4.2.1.70)  hypothetical protein |
| 3242 | AGHZ01001084.1:1..3242 | Alpha-ketoglutarate-dependent taurine dioxygenase (EC 1.14.11.17)  Taurine-binding periplasmic protein TauA |
| 3240 | AGHZ01000551.1:1..3240 | L-arabonate dehydratase (EC 4.2.1.25)  RNA polymerase sigma-54 factor RpoN |
| 3224 | AGHZ01001158.1:1..3224 | ATPase  FIG01211831: hypothetical protein  GDP-mannose pyrophosphatase YffH  hypothetical protein |
| 3218 | AGHZ01000664.1:1..3218 | Glutathione reductase (EC 1.8.1.7)  Permease of the drug/metabolite transporter (DMT) superfamily  Transport protein |
| 3215 | AGHZ01000330.1:1..3215 | FIG00582408: hypothetical protein  TonB-dependent receptor  hypothetical protein  hypothetical protein |
| 3213 | AGHZ01000312.1:1..3213 | DNA polymerase III delta prime subunit (EC 2.7.7.7)  Endonuclease  Type IV pilus biogenesis protein PilZ  YefM protein (antitoxin to YoeB)  YoeB toxin protein  tRNA-Val-CAC |
| 3203 | AGHZ01000397.1:1..3203 | DNA polymerase related protein  Sensory subunit of low CO2-induced protein complex2C putative  hypothetical protein  unknown |
| 3200 | AGHZ01000297.1:1..3200 | Biopolymer transport protein ExbD/TolR  Ferric siderophore transport system2C biopolymer transport protein ExbB  Lipid A export ATP-binding/permease protein MsbA (EC 3.6.3.25) |
| 3194 | AGHZ01000082.1:1..3194 | Pectate lyase precursor (EC 4.2.2.2)  hypothetical protein |
| 3187 | AGHZ01000899.1:1..3187 | 12C4-lactonase (EC 3.1.1.25)  2-dehydro-3-deoxyphosphogalactonate aldolase (EC 4.1.2.21)  Galactonate dehydratase (EC 4.2.1.6) |
| 3183 | AGHZ01000428.1:1..3183 | Cyclohexadienyl dehydratase (EC 4.2.1.51)(EC 4.2.1.91)  Transcriptional regulator2C AraC family  hypothetical protein  hypothetical protein |
| 3177 | AGHZ01000035.1:1..3177 | 5-nucleotidase SurE (EC 3.1.3.5)  FIG139438: lipoprotein B  Protein-L-isoaspartate O-methyltransferase (EC 2.1.1.77)  hypothetical protein  ortholog of Bordetella pertussis (BX470248) BP2475 |
| 3175 | AGHZ01000424.1:1..3175 | FIG01209995: hypothetical protein  Ribonucleotide reductase transcriptional regulator NrdR  Serine hydroxymethyltransferase (EC 2.1.2.1)  acetyltransferase |
| 3167 | AGHZ01000845.1:1..3167 | FIG01209779: hypothetical protein  Magnesium and cobalt transport protein CorA  Succinate-semialdehyde dehydrogenase [NAD] (EC 1.2.1.24); Succinate-semialdehyde dehydrogenase [NADP ] (EC 1.2.1.16) |
| 3166 | AGHZ01000284.1:1..3166 | FIG01210301: hypothetical protein  TPR domain protein2C putative component of TonB system  Zn-dependent protease with chaperone function PA4632 |
| 3163 | AGHZ01000960.1:1..3163 | Flagellar motor rotation protein MotA  Signal transduction histidine kinase CheA (EC 2.7.3.-) |
| 3162 | AGHZ01000516.1:1..3162 | Acyl-CoA-binding protein  FIG01210541: hypothetical protein  Transcriptional regulator2C TetR family  acetyltransferase2C GNAT family  hypothetical protein |
| 3155 | AGHZ01000772.1:1..3155 | FIG01112874: hypothetical protein  esterase/lipase/thioesterase family protein  tRNA uridine 5-carboxymethylaminomethyl modification enzyme GidA |
| 3152 | AGHZ01000398.1:1..3152 | Cytochrome c heme lyase subunit CcmL  Cytochrome c-type biogenesis protein CcmG/DsbE2C thiol:disulfide oxidoreductase  FIG024006: iron uptake protein  putative C-type cytochrome biogenesis protein |
| 3146 | AGHZ01000251.1:1..3146 | FIG01211261: hypothetical protein  FIG01212194: hypothetical protein  Methylcrotonyl-CoA carboxylase biotin-containing subunit (EC 6.4.1.4)  cold-shock DNA-binding domain protein |
| 3141 | AGHZ01001068.1:1..3141 | FIG01209899: hypothetical protein  RND efflux system2C outer membrane lipoprotein CmeC |
| 3139 | AGHZ01000964.1:1..3139 | Selenoprotein O and cysteine-containing homologs  transcriptional regulator2C TetR family |
| 3139 | AGHZ01000815.1:1..3139 | FIG00509706: hypothetical protein |
| 3139 | AGHZ01000590.1:1..3139 | 5-methyltetrahydrofolate--homocysteine methyltransferase (EC 2.1.1.13) |
| 3137 | AGHZ01000463.1:1..3137 | Nitroreductase  Protein yceI precursor  Transcriptional regulator2C PadR family  iron-chelator utilization protein |
| 3137 | AGHZ01000419.1:1..3137 | 2-methylcitrate synthase (EC 2.3.3.5)  Acid-resistant locus arl7 (Fragment)  Methylisocitrate lyase (EC 4.1.3.30) |
| 3132 | AGHZ01000809.1:1..3132 | 3-oxoacyl-[acyl-carrier protein] reductase (EC 1.1.1.100)  Transcriptional regulator2C AraC family  Transcriptional regulator2C HxlR family  hypothetical protein |
| 3126 | AGHZ01000579.1:1..3126 | Cellulose synthase catalytic subunit [UDP-forming] (EC 2.4.1.12) |
| 3125 | AGHZ01000329.1:1..3125 | 23Sr RNA gene  Biotin carboxyl carrier protein of acetyl-CoA carboxylase  Biotin carboxylase of acetyl-CoA carboxylase (EC 6.3.4.14)  hypothetical protein  hypothetical protein  hypothetical protein |
| 3122 | AGHZ01000856.1:1..3122 | ADP compounds hydrolase NudE (EC 3.6.1.-)  Adenosylmethionine-8-amino-7-oxononanoate aminotransferase (EC 2.6.1.62) |
| 3115 | AGHZ01000080.1:1..3115 | C4-type zinc finger protein2C DksA/TraR family  hypothetical protein  hypothetical protein  hypothetical protein  putative secreted protein  putative secreted protein |
| 3092 | AGHZ01000317.1:1..3092 | FIG01113176: hypothetical protein |
| 3092 | AGHZ01000156.1:1..3092 | Ribulose-5-phosphate 4-epimerase and related epimerases and aldolases  Xylulose kinase (EC 2.7.1.17) |
| 3081 | AGHZ01000546.1:1..3081 | Polymyxin resistance protein ArnT2C undecaprenyl phosphate-alpha-L-Ara4N transferase; Melittin resistance protein PqaB  Ribosomal RNA small subunit methyltransferase B (EC 2.1.1.-) |
| 3067 | AGHZ01001351.1:1..3067 | Pirin |
| 3066 | AGHZ01000736.1:1..3066 |  |
| 3059 | AGHZ01001091.1:1..3059 | 23S rRNA (Uracil-5-) -methyltransferase RumA (EC 2.1.1.-)  FIG01210304: hypothetical protein |
| 3058 | AGHZ01000832.1:1..3058 | N-formylglutamate deformylase (EC 3.5.1.68)  Urocanate hydratase (EC 4.2.1.49) |
| 3048 | AGHZ01000108.1:1..3048 | Hemin uptake protein  TonB-dependent hemin 2C ferrichrome receptor  voltage-gated potassium channel beta subunit |
| 3042 | AGHZ01000091.1:1..3042 | hypothetical protein |
| 3040 | AGHZ01000393.1:1..3040 | Arginase (EC 3.5.3.1)  Entericidin EcnAB  Protein yjbJ  entericidin A  hypothetical protein  hypothetical protein  tRNA-Thr-TGT |
| 3032 | AGHZ01000462.1:1..3032 | Radical SAM domain protein  biotin synthesis protein  hypothetical protein |
| 3023 | AGHZ01000408.1:1..3023 | Cell division protein FtsA  Cell division protein FtsQ |
| 3022 | AGHZ01000966.1:1..3022 | TonB-dependent receptor |
| 3022 | AGHZ01000268.1:1..3022 | Beta-lactamase (EC 3.5.2.6)  COG1801: Uncharacterized conserved protein |
| 3014 | AGHZ01000303.1:1..3014 | Adenosylhomocysteinase (EC 3.3.1.1)  D-2-hydroxyglutarate dehydrogenase  DNA-binding response regulator KdpE  Osmosensitive K channel histidine kinase KdpD (EC 2.7.3.-)  hypothetical protein |
| 3013 | AGHZ01000310.1:1..3013 | Lipid A export ATP-binding/permease protein MsbA  NG2CNG-dimethylarginine dimethylaminohydrolase 1 (EC 3.5.3.18) |
| 3008 | AGHZ01001274.1:1..3008 | Dipeptidyl aminopeptidases/acylaminoacyl-peptidases |
| 3008 | AGHZ01001147.1:1..3008 | Ferrichrome-iron receptor  PnuC protein  Transcriptional regulator2C GntR family |
| 3005 | AGHZ01000287.1:1..3005 | 1-acyl-sn-glycerol-3-phosphate acyltransferase (EC 2.3.1.51)  CDP-diacylglycerol-glycerol-3-phosphate 3-phosphatidyltransferase-related protein  DNA-binding protein Fis  Phosphatidate cytidylyltransferase (EC 2.7.7.41)  hypothetical protein |
| 3000 | AGHZ01000036.1:1..3000 | Fumarate hydratase class I2C aerobic (EC 4.2.1.2)  Glutathione S-transferase (EC 2.5.1.18) |
| 2990 | AGHZ01000875.1:1..2990 | Transcriptional regulator2C TetR (AcrR) family [USSDB4A] |
| 2983 | AGHZ01001176.1:1..2983 | L-fuco-beta-pyranose dehydrogenase  PUTATIVE HYDROLASE PHOSPHATASE PROTEIN( EC:3.1.3.- )  hypothetical protein |
| 2963 | AGHZ01000990.1:1..2963 | FIG01211332: hypothetical protein  hypothetical protein |
| 2961 | AGHZ01001639.1:1..2961 | 3-hydroxyacyl-CoA dehydrogenase type II  Hydroxymethylglutaryl-CoA lyase (EC 4.1.3.4)  Lactoylglutathione lyase and related lyases  Translation elongation factor P-related protein |
| 2961 | AGHZ01000346.1:1..2961 | Alkaline phosphatase (EC 3.1.3.1)  Glucose-6-phosphate isomerase (EC 5.3.1.9) |
| 2958 | AGHZ01000672.1:1..2958 | 3-oxoacyl-[acyl-carrier protein] reductase (EC 1.1.1.100)  FIG01210333: hypothetical protein  Histidine utilization repressor |
| 2953 | AGHZ01000292.1:1..2953 | Dipeptidyl carboxypeptidase Dcp (EC 3.4.15.5)  hypothetical protein |
| 2951 | AGHZ01001573.1:1..2951 | Outer membrane receptor for ferric coprogen and ferric-rhodotorulic acid  Tetraacyldisaccharide 4'-kinase (EC 2.7.1.130) |
| 2951 | AGHZ01000161.1:1..2951 | Diacylglycerol kinase (EC 2.7.1.107)  FIG01209891: hypothetical protein  Integral membrane protein TerC  LemA family protein |
| 2948 | AGHZ01000533.1:1..2948 | FIG01210371: hypothetical protein  Transcriptional regulator2C TetR family |
| 2941 | AGHZ01000618.1:1..2941 | hypothetical protein  pilus biogenesis protein |
| 2930 | AGHZ01001080.1:1..2930 |  |
| 2930 | AGHZ01000909.1:1..2930 | Enoyl-CoA hydratase (EC 4.2.1.17)  Hypothetical nudix hydrolase YeaB  Macrophage infectivity potentiator |
| 2924 | AGHZ01000752.1:1..2924 | Catalase (EC 1.11.1.6) / Peroxidase (EC 1.11.1.7)  hypothetical protein |
| 2919 | AGHZ01001027.1:1..2919 | FIG01210385: hypothetical protein  FIG01211525: hypothetical protein  Glycosyltransferase  UDP-22C3-diacylglucosamine hydrolase (EC 3.6.1.-) |
| 2917 | AGHZ01000333.1:1..2917 | Membrane-bound lytic murein transglycosylase D precursor (EC 3.2.1.-)  Peptidyl-prolyl cis-trans isomerase ppiD (EC 5.2.1.8) |
| 2917 | AGHZ01000077.1:1..2917 | ABC-type anion transport system2C duplicated permease component  ABC-type nitrate/sulfonate/bicarbonate transport system2C ATPase component  hypothetical protein  hypothetical protein |
| 2913 | AGHZ01001066.1:1..2913 | Dipeptidyl peptidase IV in 4-hydroxyproline catabolic gene cluster  FIG01211780: hypothetical protein |
| 2911 | AGHZ01000747.1:1..2911 | Ribosome recycling factor  Uridylate kinase (EC 2.7.4.-)  hypothetical protein |
| 2904 | AGHZ01000089.1:1..2904 | 3-oxoacyl-[acyl-carrier protein] reductase (EC 1.1.1.100)  3-oxoacyl-[acyl-carrier-protein] synthase2C KASII (EC 2.3.1.41)  Acyl carrier protein  Para-aminobenzoate synthase2C aminase component (EC 2.6.1.85) |
| 2902 | AGHZ01001521.1:1..2902 | Beta-ketoadipate enol-lactone hydrolase (EC 3.1.1.24)  FIG00964901: hypothetical protein  Uncharacterized protein conserved in bacteria  hypothetical protein  hypothetical protein |
| 2892 | AGHZ01000652.1:1..2892 | Ribosome-binding factor A  Translation initiation factor 2 |
| 2887 | AGHZ01000730.1:1..2887 | N-acetyl-L2CL-diaminopimelate deacetylase (EC 3.5.1.47) |
| 2887 | AGHZ01000662.1:1..2887 | amino acid transporter  hypothetical protein  hypothetical protein |
| 2887 | AGHZ01000554.1:1..2887 | Phosphate transport ATP-binding protein PstB (TC 3.A.1.7.1)  Phosphate transport system permease protein PstA (TC 3.A.1.7.1)  Phosphate transport system permease protein PstC (TC 3.A.1.7.1) |
| 2883 | AGHZ01000410.1:1..2883 | FIG01212086: hypothetical protein  hypothetical protein  transcriptional regulator |
| 2880 | AGHZ01000353.1:1..2880 | DUF1022 domain-containing protein  proteinase |
| 2865 | AGHZ01000373.1:1..2865 | Glutathione-regulated potassium-efflux system protein KefB  hypothetical protein |
| 2855 | AGHZ01000974.1:1..2855 | hypothetical protein |
| 2855 | AGHZ01000409.1:1..2855 | Cell division protein FtsW  Phospho-N-acetylmuramoyl-pentapeptide-transferase (EC 2.7.8.13)  UDP-N-acetylmuramoylalanyl-D-glutamyl-22C6-diaminopimelate--D-alanyl-D-alanine ligase (EC 6.3.2.10) |
| 2854 | AGHZ01000885.1:1..2854 | FIG01209962: hypothetical protein  LigA |
| 2852 | AGHZ01001501.1:1..2852 | hypothetical protein  hypothetical protein  hypothetical protein  putative secreted protein |
| 2850 | AGHZ01001683.1:1..2850 |  |
| 2848 | AGHZ01000578.1:1..2848 | LSU m5C1962 methyltransferase RlmI  export protein |
| 2841 | AGHZ01000453.1:1..2841 | NAD(P) transhydrogenase alpha subunit (EC 1.6.1.2)  NAD(P) transhydrogenase subunit beta (EC 1.6.1.2)  RNA polymerase sigma-54 factor RpoN |
| 2840 | AGHZ01000836.1:1..2840 | 3-oxoacyl-[acyl-carrier-protein] synthase2C KASIII (EC 2.3.1.41)  COG1399 protein2C clustered with ribosomal protein L32p  LSU ribosomal protein L32p |
| 2839 | AGHZ01000735.1:1..2839 | Aldehyde dehydrogenase B (EC 1.2.1.22)  tRNA-Arg-CCT |
| 2825 | AGHZ01001130.1:1..2825 | 5-Hydroxyisourate Hydrolase (HIUase) (EC 3.5.2.17)  FIG00483075: hypothetical protein  Oxidoreductase (EC 1.1.1.-) |
| 2821 | AGHZ01001006.1:1..2821 | Aspartate aminotransferase (EC 2.6.1.1) |
| 2812 | AGHZ01000348.1:1..2812 | FIG01213181: hypothetical protein  Phosphoribosylformylglycinamidine cyclo-ligase (EC 6.3.3.1)  hypothetical protein |
| 2806 | AGHZ01000808.1:1..2806 | Glutamate-1-semialdehyde aminotransferase (EC 5.4.3.8)  hypothetical protein |
| 2801 | AGHZ01000543.1:1..2801 | carboxylesterase type B  hypothetical protein  hypothetical protein |
| 2799 | AGHZ01000576.1:1..2799 | Cytoplasmic axial filament protein CafA and Ribonuclease G (EC 3.1.4.-)  Septum formation protein Maf  hypothetical protein |
| 2790 | AGHZ01000162.1:1..2790 | N-acetylglucosamine-regulated TonB-dependent outer membrane receptor  cellulase |
| 2789 | AGHZ01000539.1:1..2789 | FIG01211880: hypothetical protein |
| 2778 | AGHZ01000452.1:1..2778 |  |
| 2776 | AGHZ01000790.1:1..2776 | Cytochrome d ubiquinol oxidase subunit I (EC 1.10.3.-) |
| 2775 | AGHZ01001469.1:1..2775 | Outer membrane component of tripartite multidrug resistance system  Putrescine transport ATP-binding protein PotG (TC 3.A.1.11.2)  Putrescine transport system permease protein PotH (TC 3.A.1.11.2) |
| 2775 | AGHZ01001014.1:1..2775 | 3-hydroxydecanoyl-[ACP] dehydratase (EC 4.2.1.60)  3-oxoacyl-[ACP] reductase (EC 1.1.1.100)  FIG021862: membrane protein2C exporter  bll7766; hypothetical protein |
| 2760 | AGHZ01000595.1:1..2760 | avirulence protein  hypothetical protein |
| 2759 | AGHZ01000552.1:1..2759 | Cytochrome c-type biogenesis protein CcmC2C putative heme lyase for CcmE  Cytochrome c-type biogenesis protein CcmE2C heme chaperone  hypothetical protein |
| 2758 | AGHZ01000526.1:1..2758 | TonB-dependent receptor |
| 2756 | AGHZ01000498.1:1..2756 | Membrane-bound lytic murein transglycosylase B precursor (EC 3.2.1.-) |
| 2755 | AGHZ01000313.1:1..2755 | FIG027190: Putative transmembrane protein  Nucleoside triphosphate pyrophosphohydrolase MazG  Protein of unknown function UPF0060  Putative activity regulator of membrane protease YbbK  Putative stomatin/prohibitin-family membrane protease subunit YbbK  hypothetical protein |
| 2746 | AGHZ01001703.1:1..2746 | Cyclopropane-fatty-acyl-phospholipid synthase-like protein2C clusters with FIG005069  Endoribonuclease L-PSP  FIG005069: Hypothetical protein  hypothetical protein |
| 2743 | AGHZ01000666.1:1..2743 | Biotin synthase related domain containing protein  Putative cytoplasmic protein |
| 2740 | AGHZ01000245.1:1..2740 | Outer membrane lipoprotein carrier protein LolA  Transglutaminase-like enzymes2C putative cysteine proteases |
| 2738 | AGHZ01000868.1:1..2738 |  |
| 2731 | AGHZ01000782.1:1..2731 | 3-ketoacyl-CoA thiolase (EC 2.3.1.16) @ Acetyl-CoA acetyltransferase (EC 2.3.1.9)  hypothetical protein  outer membrane protein |
| 2729 | AGHZ01000826.1:1..2729 | hypothetical protein |
| 2722 | AGHZ01000621.1:1..2722 | Methyl-accepting chemotaxis protein I (serine chemoreceptor protein)  hypothetical protein |
| 2712 | AGHZ01000233.1:1..2712 | Acetyl-CoA acetyltransferase (EC 2.3.1.9) @ Beta-ketoadipyl CoA thiolase (EC 2.3.1.-)  Protocatechuate 32C4-dioxygenase alpha chain (EC 1.13.11.3)  Protocatechuate 32C4-dioxygenase beta chain (EC 1.13.11.3) |
| 2711 | AGHZ01000705.1:1..2711 | Cystathionine gamma-synthase (EC 2.5.1.48)  Homoserine O-acetyltransferase (EC 2.3.1.31) |
| 2704 | AGHZ01000700.1:1..2704 | Oar protein |
| 2704 | AGHZ01000322.1:1..2704 | Acetoacetyl-CoA reductase (EC 1.1.1.36)  FIG01210620: hypothetical protein  PhbF  hypothetical protein |
| 2697 | AGHZ01000449.1:1..2697 | Cell division protein FtsX  Response regulator protein  Uracil-DNA glycosylase2C family 1 |
| 2687 | AGHZ01000742.1:1..2687 | Amidophosphoribosyltransferase (EC 2.4.2.14)  Colicin V production protein  DedD protein |
| 2678 | AGHZ01001459.1:1..2678 | Radical SAM domain protein  hypothetical protein |
| 2673 | AGHZ01000741.1:1..2673 | LigA |
| 2659 | AGHZ01000107.1:1..2659 | Putative OMR family iron-siderophore receptor precursor  Sensor histidine kinase |
| 2648 | AGHZ01000812.1:1..2648 | FIG01111726: hypothetical protein  Thymidine phosphorylase (EC 2.4.2.4)  Uncharacterized protein conserved in bacteria |
| 2648 | AGHZ01000763.1:1..2648 | FIG01213735: hypothetical protein  PHA synthase subunit  Polyhydroxyalkanoic acid synthase  Putative stress-responsive transcriptional regulator |
| 2645 | AGHZ01001193.1:1..2645 | Glucose dehydrogenase2C PQQ-dependent (EC 1.1.5.2)  hypothetical protein |
| 2637 | AGHZ01000727.1:1..2637 | Gamma-glutamyltranspeptidase (EC 2.3.2.2)  hypothetical protein  transcriptional regulator lysR family |
| 2634 | AGHZ01000216.1:1..2634 | phage-related integrase |
| 2632 | AGHZ01000518.1:1..2632 | 4-hydroxybenzoate transporter  Quinate/shikimate dehydrogenase [Pyrroloquinoline-quinone] (EC 1.1.99.25) |
| 2630 | AGHZ01000514.1:1..2630 | FIG01210021: hypothetical protein  Thymidine phosphorylase (EC 2.4.2.4)  Xaa-Pro aminopeptidase (EC 3.4.11.9)  Xaa-Pro dipeptidase PepQ (EC 3.4.13.9) |
| 2623 | AGHZ01000496.1:1..2623 | hypothetical protein  hypothetical protein  putative diguanylate cyclase (GGDEF)/phosphodiesterase (EAL) with PAS domain |
| 2622 | AGHZ01000716.1:1..2622 | Two-component response regulator  two-component system sensor protein |
| 2619 | AGHZ01000125.1:1..2619 | Cytochrome c-type biogenesis protein CcmC2C putative heme lyase for CcmE  Cytochrome c-type biogenesis protein CcmE2C heme chaperone  hypothetical protein  serine protease |
| 2614 | AGHZ01000952.1:1..2614 | Peptide deformylase (EC 3.5.1.88)  Uncharacterized protein with LysM domain2C COG1652 |
| 2608 | AGHZ01000607.1:1..2608 | Nucleoside permease NupC |
| 2588 | AGHZ01001077.1:1..2588 | Vibrioferrin amide bond forming protein PvsD @ Siderophore synthetase superfamily2C group A |
| 2585 | AGHZ01000025.1:1..2585 | FIG01211260: hypothetical protein  Lytic transglycosylase  hypothetical protein |
| 2580 | AGHZ01000492.1:1..2580 | Na -driven multidrug efflux pump  protease IV |
| 2565 | AGHZ01000106.1:1..2565 | 3-carboxy-cis2Ccis-muconate cycloisomerase (EC 5.5.1.2)  4-carboxymuconolactone decarboxylase (EC 4.1.1.44)  Beta-ketoadipate enol-lactone hydrolase (EC 3.1.1.24)  Transcriptional regulator2C HxlR family |
| 2559 | AGHZ01001019.1:1..2559 | 1-acyl-sn-glycerol-3-phosphate acyltransferase (EC 2.3.1.51)  FIG01211085: hypothetical protein |
| 2557 | AGHZ01001236.1:1..2557 | ABC transporter2C ATP-binding protein  putative MFS transporter |
| 2556 | AGHZ01000995.1:1..2556 | Flagellar biosynthesis protein FliS  Flagellar hook-associated protein FliD  hypothetical protein |
| 2554 | AGHZ01001629.1:1..2554 | Hemolysins and related proteins containing CBS domains  hypothetical protein |
| 2545 | AGHZ01001042.1:1..2545 | DNA topology modulation protein FLAR-related protein  FIG01212077: hypothetical protein  Phosphopantetheine adenylyltransferase (EC 2.7.7.3)  Ribosomal RNA small subunit methyltransferase D (EC 2.1.1.-)  similar to nucleoside-diphosphate-sugar epimerases |
| 2545 | AGHZ01000295.1:1..2545 | Cardiolipin synthetase (EC 2.7.8.-)  Hydroxyacylglutathione hydrolase (EC 3.1.2.6) |
| 2542 | AGHZ01001427.1:1..2542 | SprB |
| 2542 | AGHZ01000667.1:1..2542 | Periplasmic binding protein  Pyridoxamine 5'-phosphate oxidase (EC 1.4.3.5)  Shikimate kinase I (EC 2.7.1.71) |
| 2533 | AGHZ01000991.1:1..2533 | Acyl-CoA dehydrogenase (EC 1.3.99.3) |
| 2530 | AGHZ01001219.1:1..2530 | FIG01211728: hypothetical protein |
| 2530 | AGHZ01000625.1:1..2530 | Uncharacterized ABC transporter2C ATP-binding protein YrbF  Uncharacterized ABC transporter2C auxiliary component YrbC  Uncharacterized ABC transporter2C periplasmic component YrbD  Uncharacterized ABC transporter2C permease component YrbE |
| 2522 | AGHZ01001240.1:1..2522 | TonB-dependent receptor |
| 2520 | AGHZ01000630.1:1..2520 | Vibriolysin2C extracellular zinc protease (EC 3.4.24.25) @ Pseudolysin2C extracellular zinc protease (EC 3.4.24.26) |
| 2512 | AGHZ01001567.1:1..2512 | TPR domain protein in aerotolerance operon |
| 2505 | AGHZ01000198.1:1..2505 | INTEGRAL MEMBRANE PROTEIN (Rhomboid family)  Transcription termination factor Rho |
| 2498 | AGHZ01000838.1:1..2498 | Outer membrane vitamin B12 receptor BtuB  hypothetical protein  hypothetical protein |
| 2495 | AGHZ01000638.1:1..2495 | O-antigen acetylase  lipoprotein2C putative |
| 2494 | AGHZ01000075.1:1..2494 | D-serine/D-alanine/glycine transporter  Magnesium and cobalt transport protein |
| 2492 | AGHZ01001349.1:1..2492 | extracellular serine protease  putative ankyrin-like membrane protein |
| 2490 | AGHZ01001620.1:1..2490 | Soluble lytic murein transglycosylase precursor (EC 3.2.1.-) |
| 2490 | AGHZ01001011.1:1..2490 | 12C4-alpha-glucan (glycogen) branching enzyme2C GH-13-type (EC 2.4.1.18) |
| 2488 | AGHZ01000378.1:1..2488 | Xanthine dehydrogenase2C molybdenum binding subunit (EC 1.17.1.4)  hypothetical protein |
| 2486 | AGHZ01001079.1:1..2486 | Sulfite reductase [NADPH] flavoprotein alpha-component (EC 1.8.1.2) |
| 2481 | AGHZ01000890.1:1..2481 | FIG001154: CcsA-related protein  Signal recognition particle2C subunit Ffh SRP54 (TC 3.A.5.1.1) |
| 2468 | AGHZ01000770.1:1..2468 | D-xylose proton-symporter XylE  hypothetical protein |
| 2461 | AGHZ01000688.1:1..2461 | FIG01210495: hypothetical protein  FIG01210706: hypothetical protein  Ferredoxin II  RNA polymerase sigma-70 factor |
| 2460 | AGHZ01000334.1:1..2460 | Membrane protein2C putative  Putative diheme cytochrome c-553 |
| 2446 | AGHZ01001051.1:1..2446 | Endonuclease III (EC 4.2.99.18)  Sulfate transporter family protein in cluster with carbonic anhydrase |
| 2445 | AGHZ01000023.1:1..2445 | FIG071884: Hypothetical protein  Integral membrane protein CcmA involved in cell shape determination  probable iron binding protein from the HesB\_IscA\_SufA family |
| 2441 | AGHZ01000326.1:1..2441 | TonB-dependent receptor |
| 2440 | AGHZ01001046.1:1..2440 | Xylulose kinase (EC 2.7.1.17) |
| 2438 | AGHZ01000854.1:1..2438 | Transcriptional regulator  hypothetical protein |
| 2435 | AGHZ01000765.1:1..2435 | FIG004453: protein YceG like  Para-aminobenzoate synthase2C aminase component (EC 2.6.1.85)  Thymidylate kinase (EC 2.7.4.9) |
| 2434 | AGHZ01001297.1:1..2434 | FIG01210093: hypothetical protein  FIG01212404: hypothetical protein |
| 2429 | AGHZ01000677.1:1..2429 | DNA polymerase III epsilon subunit (EC 2.7.7.7)  FIG005121: SAM-dependent methyltransferase (EC 2.1.1.-)  Ribonuclease HI (EC 3.1.26.4)  protein phosphatase |
| 2415 | AGHZ01000609.1:1..2415 | FIG01210241: hypothetical protein  Nucleoside-diphosphate-sugar epimerases  Porphobilinogen synthase (EC 4.2.1.24) |
| 2408 | AGHZ01001435.1:1..2408 | membrane protein2C putative  virulence protein |
| 2405 | AGHZ01000600.1:1..2405 | Bacterioferritin  Peroxiredoxin  low molecular weight heat shock protein |
| 2405 | AGHZ01000184.1:1..2405 | Pectate lyase precursor (EC 4.2.2.2)  Ribosomal-protein-S18p-alanine acetyltransferase (EC 2.3.1.-) |
| 2375 | AGHZ01001641.1:1..2375 | 4-hydroxyproline epimerase (EC 5.1.1.8)  Transcriptional regulator2C AraC family |
| 2375 | AGHZ01000383.1:1..2375 | Histidinol-phosphatase (EC 3.1.3.15) / Imidazoleglycerol-phosphate dehydratase (EC 4.2.1.19)  Imidazole glycerol phosphate synthase amidotransferase subunit (EC 2.4.2.-) |
| 2374 | AGHZ01000720.1:1..2374 | Acetyl-coenzyme A carboxyl transferase beta chain (EC 6.4.1.2) |
| 2365 | AGHZ01001711.1:1..2365 | Vibrioferrin receptor PvuA |
| 2359 | AGHZ01000712.1:1..2359 | Cellulose synthase operon protein C  Peptide deformylase (EC 3.5.1.88) |
| 2357 | AGHZ01000364.1:1..2357 | FIG017670: hypothetical protein  FIG019278: hypothetical protein  diguanylate cyclase/phosphodiesterase (GGDEF |
| 2353 | AGHZ01001111.1:1..2353 | Oligopeptidase A (EC 3.4.24.70) |
| 2340 | AGHZ01000177.1:1..2340 | FIG01211915: hypothetical protein |
| 2334 | AGHZ01001668.1:1..2334 | aminopeptidase |
| 2333 | AGHZ01000656.1:1..2333 | Thiol:disulfide interchange protein DsbC  Tyrosine recombinase XerD |
| 2332 | AGHZ01001094.1:1..2332 | Cellulase  Methyl-accepting chemotaxis protein I (serine chemoreceptor protein)  Uracil phosphoribosyltransferase (EC 2.4.2.9) |
| 2331 | AGHZ01000962.1:1..2331 | Molybdopterin biosynthesis protein MoeA  Sulfur carrier protein adenylyltransferase ThiF |
| 2330 | AGHZ01000457.1:1..2330 | UPF0225 protein YchJ |
| 2328 | AGHZ01001000.1:1..2328 | hypothetical protein  hypothetical protein |
| 2322 | AGHZ01001099.1:1..2322 | Endonuclease |
| 2322 | AGHZ01000265.1:1..2322 | DNA recombination protein RmuC  Glutathione peroxidase (EC 1.11.1.9) |
| 2318 | AGHZ01000241.1:1..2318 | FIG01210486: hypothetical protein  Methyltransferase (EC 2.1.1.-)  hypothetical protein |
| 2315 | AGHZ01001115.1:1..2315 | MFS permease protein  hypothetical protein |
| 2315 | AGHZ01000932.1:1..2315 | FIG000605: protein co-occurring with transport systems (COG1739)  hypothetical protein |
| 2311 | AGHZ01000927.1:1..2311 | CBSS-498211.3.peg.1514: hypothetical protein  GTP cyclohydrolase I (EC 3.5.4.16) type 1 |
| 2302 | AGHZ01000429.1:1..2302 | Putative outer membrane or secreted lipoprotein  hypothetical protein  hypothetical protein |
| 2300 | AGHZ01001078.1:1..2300 | FIG00537023: hypothetical protein  PsiF  Vibrioferrin decarboxylase protein PvsE |
| 2300 | AGHZ01001056.1:1..2300 | Azurin  Periplasmic aromatic amino acid aminotransferase beta precursor (EC 2.6.1.57)  transcriptional regulator |
| 2291 | AGHZ01001613.1:1..2291 | FKBP-type peptidyl-prolyl cis-trans isomerase SlyD (EC 5.2.1.8)  Putative heat shock protein YegD  cation transport protein |
| 2291 | AGHZ01000754.1:1..2291 | FIG01211108: hypothetical protein  N-acetyltransferase  Translation elongation factor P Lys34:lysine transferase |
| 2290 | AGHZ01000796.1:1..2290 | FIG002903: a protein of unknown function perhaps involved in purine metabolism  methyl-accepting chemotaxis sensory transducer |
| 2287 | AGHZ01000210.1:1..2287 | Methyl-accepting chemotaxis protein I (serine chemoreceptor protein)  hypothetical protein |
| 2285 | AGHZ01000849.1:1..2285 | Tryptophan synthase beta chain (EC 4.2.1.20) |
| 2285 | AGHZ01000022.1:1..2285 | conserved hypothetical protein  hypothetical protein |
| 2276 | AGHZ01001695.1:1..2276 | Acetolactate synthase large subunit (EC 2.2.1.6)  Acetolactate synthase small subunit (EC 2.2.1.6)2C Xanthomonadales type |
| 2273 | AGHZ01001233.1:1..2273 | Methyl-accepting chemotaxis protein I (serine chemoreceptor protein) |
| 2273 | AGHZ01000382.1:1..2273 | Arginyl-tRNA synthetase (EC 6.1.1.19) |
| 2273 | AGHZ01000157.1:1..2273 | conserved hypothetical protein |
| 2272 | AGHZ01000236.1:1..2272 | Coenzyme PQQ synthesis protein B  Coenzyme PQQ synthesis protein C |
| 2269 | AGHZ01000123.1:1..2269 | RNA polymerase sigma-70 factor  putative virK protein |
| 2264 | AGHZ01000183.1:1..2264 | Multicopper oxidase |
| 2259 | AGHZ01000054.1:1..2259 | chemotaxis protein  hypothetical protein |
| 2252 | AGHZ01000149.1:1..2252 | Flagellar motor rotation protein MotA  hypothetical protein |
| 2246 | AGHZ01000775.1:1..2246 | Acyl carrier protein (ACP1)  FIG022199: FAD-binding protein |
| 2240 | AGHZ01000324.1:1..2240 | Membrane fusion component of tripartite multidrug resistance system |
| 2237 | AGHZ01001144.1:1..2237 | FIG01210504: hypothetical protein |
| 2234 | AGHZ01000834.1:1..2234 | cytidine and deoxycytidylate deaminase family protein  hypothetical protein  hypothetical protein  integral membrane protein |
| 2233 | AGHZ01000201.1:1..2233 | Two-component response regulator CreB |
| 2232 | AGHZ01000811.1:1..2232 | FIG01213672: hypothetical protein  Nicotinate phosphoribosyltransferase (EC 2.4.2.11) |
| 2229 | AGHZ01000855.1:1..2229 | Arsenate reductase (EC 1.20.4.1)  hypothetical protein |
| 2224 | AGHZ01000111.1:1..2224 |  |
| 2205 | AGHZ01000768.1:1..2205 | 22C3-dihydroxy-22C3-dihydro-phenylpropionate dehydrogenase (EC 1.3.1.-)  hypothetical protein |
| 2203 | AGHZ01000658.1:1..2203 | Beta-glucosidase (EC 3.2.1.21) |
| 2199 | AGHZ01001057.1:1..2199 | COG2833: uncharacterized protein  peptidyl-Asp metalloendopeptidase |
| 2199 | AGHZ01000150.1:1..2199 | Glycerophosphoryl diester phosphodiesterase (EC 3.1.4.46)  hypothetical protein |
| 2189 | AGHZ01001160.1:1..2189 | hypothetical protein  putative secreted protein  truncated cellulase S |
| 2186 | AGHZ01000529.1:1..2186 | 3-oxoadipate CoA-transferase subunit A (EC 2.8.3.6)  3-oxoadipate CoA-transferase subunit B (EC 2.8.3.6) |
| 2185 | AGHZ01000248.1:1..2185 | S-adenosylmethionine synthetase (EC 2.5.1.6) |
| 2184 | AGHZ01001545.1:1..2184 | Ferrochelatase2C protoheme ferro-lyase (EC 4.99.1.1) |
| 2184 | AGHZ01000771.1:1..2184 | GTP cyclohydrolase I (EC 3.5.4.16) type 2  tRNA-Glu-CTC |
| 2184 | AGHZ01000585.1:1..2184 | putative ABC transporter ATP-binding protein |
| 2181 | AGHZ01000237.1:1..2181 | ABC transporter ATP-binding protein |
| 2180 | AGHZ01000472.1:1..2180 | Cytochrome c heme lyase subunit CcmH  Homoserine O-acetyltransferase (EC 2.3.1.31)  Transport ATP-binding protein CydC  hypothetical protein |
| 2177 | AGHZ01000924.1:1..2177 | Epoxyqueuosine (oQ) reductase QueG |
| 2167 | AGHZ01001674.1:1..2167 | Chromosome partition protein smc |
| 2165 | AGHZ01000193.1:1..2165 | Segregation and condensation protein A |
| 2165 | AGHZ01000072.1:1..2165 | 3-deoxy-D-manno-octulosonic acid kinase (EC 2.7.1.-) |
| 2163 | AGHZ01001366.1:1..2163 | Autolysin sensor kinase (EC 2.7.3.-)  hypothetical protein |
| 2162 | AGHZ01000289.1:1..2162 | ATP-dependent DNA ligase (EC 6.5.1.1)  hypothetical protein |
| 2161 | AGHZ01000553.1:1..2161 |  |
| 2158 | AGHZ01000760.1:1..2158 | Biosynthetic arginine decarboxylase (EC 4.1.1.19)  hypothetical protein |
| 2158 | AGHZ01000756.1:1..2158 |  |
| 2148 | AGHZ01001504.1:1..2148 | 2-keto-3-deoxy-D-arabino-heptulosonate-7-phosphate synthase I alpha (EC 2.5.1.54) |
| 2136 | AGHZ01000404.1:1..2136 | Uncharacterized protein conserved in bacteria  Valine--pyruvate aminotransferase (EC 2.6.1.66) |
| 2132 | AGHZ01000276.1:1..2132 | L-alanine-DL-glutamate epimerase  probable exported protein STY2149 |
| 2122 | AGHZ01001628.1:1..2122 | ExoD protein  Luciferase-like  hypothetical protein |
| 2121 | AGHZ01001546.1:1..2121 | Two-component response regulator |
| 2118 | AGHZ01000405.1:1..2118 | BarA-associated response regulator UvrY ( GacA SirA) |
| 2114 | AGHZ01000853.1:1..2114 | Dipeptidyl peptidase IV |
| 2107 | AGHZ01000362.1:1..2107 | Isopropylmalate/homocitrate/citramalate synthases  Protein of unknown function DUF218 |
| 2106 | AGHZ01000608.1:1..2106 | ATP-dependent DNA ligase (EC 6.5.1.1) LigC  mRNA 3-end processing factor |
| 2102 | AGHZ01001644.1:1..2102 | hypothetical protein |
| 2100 | AGHZ01001179.1:1..2100 | UDP-N-acetylenolpyruvoylglucosamine reductase (EC 1.1.1.158)  integral membrane protein |
| 2100 | AGHZ01000895.1:1..2100 | Ketol-acid reductoisomerase (EC 1.1.1.86) |
| 2096 | AGHZ01001096.1:1..2096 | FIG01210443: hypothetical protein  FIG01211151: hypothetical protein  Trypsin-like serine proteases2C typically periplasmic2C contain C-terminal PDZ domain  hypothetical protein |
| 2096 | AGHZ01000774.1:1..2096 | RND efflux system2C membrane fusion protein CmeA |
| 2089 | AGHZ01001650.1:1..2089 | FIG138315: Putative alpha helix protein  TldD protein2C part of proposed TldE/TldD proteolytic complex (PMID 12029038) |
| 2089 | AGHZ01000557.1:1..2089 | FIG01209938: hypothetical protein  Superoxide dismutase [Cu-Zn] precursor (EC 1.15.1.1)  Uncharacterized domain COG3236 / GTP cyclohydrolase II (EC 3.5.4.25) |
| 2088 | AGHZ01000718.1:1..2088 | D-tyrosyl-tRNA(Tyr) deacylase |
| 2084 | AGHZ01000959.1:1..2084 | COG3178: Predicted phosphotransferase related to Ser/Thr protein kinases  FIG006611: nucleotidyltransferase |
| 2073 | AGHZ01001112.1:1..2073 | aminoglycoside 3'-phosphotransferase  hypothetical protein |
| 2070 | AGHZ01000454.1:1..2070 | Phenazine biosynthesis protein PhzF like  hypothetical protein  hypothetical protein |
| 2062 | AGHZ01000395.1:1..2062 | 6-phosphogluconate dehydrogenase2C decarboxylating (EC 1.1.1.44)  FIG01210026: hypothetical protein |
| 2055 | AGHZ01001064.1:1..2055 | hypothetical protein |
| 2054 | AGHZ01000399.1:1..2054 | Taurine transport system permease protein TauC  hypothetical protein  hypothetical protein |
| 2052 | AGHZ01000794.1:1..2052 | Tryptophanyl-tRNA synthetase (EC 6.1.1.2) |
| 2050 | AGHZ01000982.1:1..2050 | DNA polymerase III alpha subunit (EC 2.7.7.7)  FIG00454545: hypothetical protein  Transcriptional regulator2C ArsR family |
| 2050 | AGHZ01000597.1:1..2050 | RND efflux system2C inner membrane transporter CmeB |
| 2049 | AGHZ01001686.1:1..2049 | Chemotaxis protein CheD  Chemotaxis protein methyltransferase CheR (EC 2.1.1.80) |
| 2044 | AGHZ01000475.1:1..2044 | hypothetical protein |
| 2035 | AGHZ01000813.1:1..2035 | Uncharacterized iron-regulated membrane protein; Iron-uptake factor PiuB  hypothetical protein |
| 2034 | AGHZ01000985.1:1..2034 | Oxidoreductase  Phosphoglucosamine mutase (EC 5.4.2.10)  short chain dehydrogenase |
| 2033 | AGHZ01001190.1:1..2033 | FIG01210804: hypothetical protein  Glutaredoxin 3 |
| 2032 | AGHZ01000479.1:1..2032 | Acriflavin resistance protein |
| 2028 | AGHZ01000302.1:1..2028 | FIG01210133: hypothetical protein  TPR repeat |
| 2027 | AGHZ01000274.1:1..2027 | hypothetical protein |
| 2026 | AGHZ01001609.1:1..2026 | Nitroreductase |
| 2026 | AGHZ01000470.1:1..2026 | ABC transporter2C ATP-binding/permease protein  hypothetical protein |
| 2024 | AGHZ01001017.1:1..2024 | probable aminopeptidase |
| 2021 | AGHZ01000753.1:1..2021 | hypothetical protein |
| 2011 | AGHZ01001451.1:1..2011 | GTP-binding protein EngB  methyl parathion hydrolase |
| 2010 | AGHZ01000615.1:1..2010 | ABC transporter2C ATP-binding protein  hypothetical protein |
| 2009 | AGHZ01001202.1:1..2009 | Trehalase (EC 3.2.1.28); Periplasmic trehalase precursor (EC 3.2.1.28)  hypothetical protein |
| 2007 | AGHZ01000305.1:1..2007 | Thioredoxin |
| 2005 | AGHZ01000925.1:1..2005 | LysR-family transcriptional regulator clustered with PA0057  Phosphoglycolate phosphatase (EC 3.1.3.18) |
| 1996 | AGHZ01001153.1:1..1996 | Cell division protein ZipA  MORN repeat family protein |
| 1991 | AGHZ01000769.1:1..1991 | Cysteine synthase (EC 2.5.1.47)  Siroheme synthase / Precorrin-2 oxidase (EC 1.3.1.76) / Sirohydrochlorin ferrochelatase (EC 4.99.1.4) / Uroporphyrinogen-III methyltransferase (EC 2.1.1.107)  XdhC protein (assists in molybdopterin insertion into xanthine dehydrogenase) |
| 1990 | AGHZ01000992.1:1..1990 | N-acetylmuramoyl-L-alanine amidase (EC 3.5.1.28) |
| 1988 | AGHZ01001461.1:1..1988 |  |
| 1987 | AGHZ01000131.1:1..1987 | Chemotaxis protein cheA (EC 2.7.3.-) |
| 1983 | AGHZ01000981.1:1..1983 | Phosphoribosylaminoimidazole carboxylase catalytic subunit (EC 4.1.1.21)  UPF0434 protein YcaR |
| 1983 | AGHZ01000537.1:1..1983 | FIG01111872: hypothetical protein  LSU ribosomal protein L28p  LSU ribosomal protein L33p |
| 1980 | AGHZ01001074.1:1..1980 | Positive regulator of CheA protein activity (CheW)  hypothetical protein  tRNA-Ala-CGC  virulence regulator |
| 1979 | AGHZ01001346.1:1..1979 | Two-component system sensor protein |
| 1971 | AGHZ01001550.1:1..1971 | FIG01210076: hypothetical protein |
| 1971 | AGHZ01000477.1:1..1971 |  |
| 1966 | AGHZ01000695.1:1..1966 | Histone-like protein  putative membrane protein |
| 1960 | AGHZ01000871.1:1..1960 | Sensory box histidine kinase/response regulator |
| 1957 | AGHZ01001092.1:1..1957 | Queuosine Biosynthesis QueE Radical SAM  TPR repeat containing exported protein; Putative periplasmic protein contains a protein prenylyltransferase domain |
| 1949 | AGHZ01000415.1:1..1949 | 2-amino-4-hydroxy-6-hydroxymethyldihydropteridine pyrophosphokinase (EC 2.7.6.3)  3-methyl-2-oxobutanoate hydroxymethyltransferase (EC 2.1.2.11) |
| 1946 | AGHZ01000819.1:1..1946 | Methionine ABC transporter ATP-binding protein |
| 1945 | AGHZ01000671.1:1..1945 | Glycyl-tRNA synthetase alpha chain (EC 6.1.1.14)  hypothetical protein |
| 1945 | AGHZ01000060.1:1..1945 | FIG01209846: hypothetical protein  FIG01211155: hypothetical protein |
| 1941 | AGHZ01001394.1:1..1941 | Bis(5'-nucleosyl)-tetraphosphatase (asymmetrical) (EC 3.6.1.17)  FIG001454: Transglutaminase-like enzymes2C putative cysteine proteases  Recombination protein RecR  Starvation lipoprotein Slp paralog |
| 1932 | AGHZ01001692.1:1..1932 | DNA mismatch repair protein MutS |
| 1931 | AGHZ01001632.1:1..1931 | 3-oxoacyl-(acyl carrier protein) synthase (EC 2.3.1.41)  Glycosyl transferase2C family 2  putative acyltransferase (PhnO) |
| 1929 | AGHZ01000726.1:1..1929 | Peptidase B (EC 3.4.11.23)  Putative cytoplasmic protein |
| 1923 | AGHZ01001146.1:1..1923 | Putative homoserine kinase type II2C PnuC-associated2C THI-regulated branch |
| 1920 | AGHZ01001061.1:1..1920 | Transcriptional regulator2C LysR family |
| 1920 | AGHZ01000852.1:1..1920 | Hydroxyacylglutathione hydrolase (EC 3.1.2.6) |
| 1919 | AGHZ01000548.1:1..1919 | FIG143263: Glycosyl transferase @ Dolichyl-phosphate mannose synthase related protein |
| 1913 | AGHZ01000869.1:1..1913 | COG1201: Lhr-like helicases |
| 1910 | AGHZ01001631.1:1..1910 | 22C32C42C5-tetrahydropyridine-22C6-dicarboxylate N-succinyltransferase (EC 2.3.1.117) |
| 1910 | AGHZ01000484.1:1..1910 |  |
| 1906 | AGHZ01001321.1:1..1906 | hypothetical protein |
| 1902 | AGHZ01001172.1:1..1902 | FIG00446866: hypothetical protein  MgtC/SapB transporter |
| 1896 | AGHZ01000363.1:1..1896 | Gamma-glutamyltranspeptidase (EC 2.3.2.2) |
| 1891 | AGHZ01000204.1:1..1891 | 4-hydroxyphenylpyruvate dioxygenase (EC 1.13.11.27)  Transcriptional regulator2C MarR family |
| 1888 | AGHZ01000623.1:1..1888 |  |
| 1886 | AGHZ01001344.1:1..1886 | Flagellar M-ring protein FliF  Flagellar motor switch protein FliG |
| 1886 | AGHZ01000907.1:1..1886 | FIG01210246: hypothetical protein  Transcriptional regulator2C GntR family domain / Aspartate aminotransferase (EC 2.6.1.1) |
| 1875 | AGHZ01001140.1:1..1875 | Acid phosphatase |
| 1875 | AGHZ01000891.1:1..1875 | Transport ATP-binding protein CydD |
| 1872 | AGHZ01001187.1:1..1872 | Methionine aminopeptidase (EC 3.4.11.18)  Sigma-fimbriae tip adhesin |
| 1870 | AGHZ01001003.1:1..1870 | FIG01209703: hypothetical protein  Negative regulator of beta-lactamase expression |
| 1869 | AGHZ01001531.1:1..1869 | Single-stranded-DNA-specific exonuclease RecJ (EC 3.1.-.-) |
| 1869 | AGHZ01000967.1:1..1869 | 5-methyltetrahydropteroyltriglutamate--homocysteine methyltransferase (EC 2.1.1.14) |
| 1862 | AGHZ01000500.1:1..1862 | FIG01111853: hypothetical protein |
| 1859 | AGHZ01000649.1:1..1859 |  |
| 1859 | AGHZ01000300.1:1..1859 | Fimbrial protein precursor  Protein of unknown function Smg  Rossmann fold nucleotide-binding protein Smf possibly involved in DNA uptake |
| 1854 | AGHZ01000459.1:1..1854 | Methyl-accepting chemotaxis protein I (serine chemoreceptor protein) |
| 1854 | AGHZ01000442.1:1..1854 | Oxidoreductase2C short chain dehydrogenase/reductase family  Rhodanese domain protein UPF01762C Betaproteobacterial subgroup |
| 1853 | AGHZ01000421.1:1..1853 | Protein of unknown function DUF541 |
| 1843 | AGHZ01000368.1:1..1843 | Gamma-glutamyl phosphate reductase (EC 1.2.1.41)  Glutamate 5-kinase (EC 2.7.2.11) |
| 1839 | AGHZ01000519.1:1..1839 | Alkyl hydroperoxide reductase protein C (EC 1.6.4.-) |
| 1837 | AGHZ01000293.1:1..1837 | Transcriptional regulator2C LysR family |
| 1836 | AGHZ01001643.1:1..1836 | FIG01211770: hypothetical protein  Short chain dehydrogenase |
| 1836 | AGHZ01000016.1:1..1836 | hypothetical protein  hypothetical protein  hypothetical protein |
| 1832 | AGHZ01001465.1:1..1832 | Chemotaxis regulator - transmits chemoreceptor signals to flagelllar motor components CheY  FIG01209829: hypothetical protein  Positive regulator of CheA protein activity (CheW) |
| 1829 | AGHZ01000490.1:1..1829 | FIG138056: a glutathione-dependent thiol reductase  Histone acetyltransferase HPA2 and related acetyltransferases  hypothetical protein |
| 1824 | AGHZ01001453.1:1..1824 | beta 12C4 glucosyltransferase |
| 1823 | AGHZ01000270.1:1..1823 | hydrolase  transport protein |
| 1812 | AGHZ01000471.1:1..1812 | IMP cyclohydrolase (EC 3.5.4.10) / Phosphoribosylaminoimidazolecarboxamide formyltransferase (EC 2.1.2.3) |
| 1811 | AGHZ01000733.1:1..1811 | Osmotically inducible protein OsmY |
| 1811 | AGHZ01000370.1:1..1811 | dicarboxylate transport protein  hypothetical protein |
| 1810 | AGHZ01000631.1:1..1810 | amino acid transporter |
| 1804 | AGHZ01000250.1:1..1804 | Beta-lactamase  Metallo-beta-lactamase family protein( EC:3.1.2.6 )  Transcriptional regulator2C ArsR family |
| 1797 | AGHZ01000864.1:1..1797 | Inner membrane protein CreD |
| 1797 | AGHZ01000288.1:1..1797 | Dethiobiotin synthetase (EC 6.3.3.3)  FIG01210979: hypothetical protein  Queuosine biosynthesis QueD2C PTPS-I |
| 1796 | AGHZ01000844.1:1..1796 | Membrane-bound lytic murein transglycosylase B precursor (EC 3.2.1.-) |
| 1796 | AGHZ01000821.1:1..1796 | Amino acid permease in 4-hydroxyproline catabolic gene cluster |
| 1794 | AGHZ01000936.1:1..1794 |  |
| 1790 | AGHZ01000776.1:1..1790 | FIGfam138462: Acyl-CoA synthetase2C AMP-(fatty) acid ligase  glucokinase  putative pteridine-dependent deoxygenase like protein |
| 1788 | AGHZ01000309.1:1..1788 | D-alanine--D-alanine ligase (EC 6.3.2.4)  UDP-N-acetylmuramate--alanine ligase (EC 6.3.2.8)  hypothetical protein |
| 1787 | AGHZ01000657.1:1..1787 | 32C4-dihydroxy-2-butanone 4-phosphate synthase / GTP cyclohydrolase II (EC 3.5.4.25)  62C7-dimethyl-8-ribityllumazine synthase (EC 2.5.1.9)  Transcription termination protein NusB |
| 1785 | AGHZ01001528.1:1..1785 | Acyl-CoA dehydrogenase/oxidase domain protein  LmbE-like protein  Methyltransferase type 12 |
| 1784 | AGHZ01000919.1:1..1784 | Cell wall endopeptidase2C family M23/M37 |
| 1778 | AGHZ01000160.1:1..1778 | RNA-binding protein Hfq  tRNA delta(2)-isopentenylpyrophosphate transferase (EC 2.5.1.8) |
| 1777 | AGHZ01000110.1:1..1777 | UDP-glucose 4-epimerase (EC 5.1.3.2)  hypothetical protein |
| 1772 | AGHZ01000128.1:1..1772 |  |
| 1764 | AGHZ01000416.1:1..1764 | hypothetical protein |
| 1762 | AGHZ01000071.1:1..1762 |  |
| 1758 | AGHZ01001497.1:1..1758 | FIG01111849: hypothetical protein |
| 1758 | AGHZ01001300.1:1..1758 | FIG01209993: hypothetical protein  Putative permease often clustered with de novo purine synthesis |
| 1756 | AGHZ01001291.1:1..1756 | 6-phospho-beta-glucosidase  ribokinase |
| 1756 | AGHZ01001060.1:1..1756 | Histidinol dehydrogenase (EC 1.1.1.23) |
| 1755 | AGHZ01000970.1:1..1755 | Hypothetical2C related to broad specificity phosphatases COG0406 |
| 1747 | AGHZ01000860.1:1..1747 | efflux transporter2C RND family2C MFP subunit |
| 1742 | AGHZ01000235.1:1..1742 | 4-carboxymuconolactone decarboxylase family protein  Oxidoreductase |
| 1739 | AGHZ01001139.1:1..1739 | Redox-sensitive transcriptional activator SoxR  hypothetical protein |
| 1737 | AGHZ01001196.1:1..1737 | histidine kinase/response regulator hybrid protein |
| 1734 | AGHZ01000945.1:1..1734 | Sulfate transporter2C CysZ-type  hypothetical protein  probable cellulase |
| 1731 | AGHZ01000873.1:1..1731 | FIG01211391: hypothetical protein  NADH dehydrogenase (EC 1.6.99.3)  RNA polymerase sigma factor RpoH |
| 1731 | AGHZ01000087.1:1..1731 | RND efflux system2C outer membrane lipoprotein CmeC  hypothetical protein |
| 1730 | AGHZ01001624.1:1..1730 | Vibrioferrin amide bond forming protein PvsB @ Siderophore synthetase superfamily2C group B |
| 1730 | AGHZ01000956.1:1..1730 | ApbE-like lipoprotein  calcium-binding protein |
| 1720 | AGHZ01000273.1:1..1720 | Endoglucanase (EC 3.2.1.4) |
| 1713 | AGHZ01000272.1:1..1713 | 3-ketoacyl-CoA thiolase (EC 2.3.1.16) @ Acetyl-CoA acetyltransferase (EC 2.3.1.9) |
| 1712 | AGHZ01001175.1:1..1712 | 2-keto-3-deoxy-L-fuconate dehydrogenase  22C4-diketo-3-deoxy-L-fuconate hydrolase |
| 1712 | AGHZ01000392.1:1..1712 | General secretion pathway protein E |
| 1712 | AGHZ01000148.1:1..1712 | Chloride channel protein |
| 1708 | AGHZ01000503.1:1..1708 | CDP-diacylglycerol--glycerol-3-phosphate 3-phosphatidyltransferase (EC 2.7.8.5) |
| 1708 | AGHZ01000461.1:1..1708 | FIG01111989: hypothetical protein  Na( ) H( ) antiporter subunit D  Na( ) H( ) antiporter subunit E  Na( ) H( ) antiporter subunit F  Na( ) H( ) antiporter subunit G |
| 1707 | AGHZ01000804.1:1..1707 | hypothetical protein |
| 1702 | AGHZ01001148.1:1..1702 | (3R)-hydroxymyristoyl-[ACP] dehydratase (EC 4.2.1.-)  FIG017861: hypothetical protein  FIG018329: 1-acyl-sn-glycerol-3-phosphate acyltransferase |
| 1701 | AGHZ01000749.1:1..1701 |  |
| 1698 | AGHZ01001197.1:1..1698 |  |
| 1691 | AGHZ01001479.1:1..1691 | DNA mismatch repair protein MutL  FIG01209920: hypothetical protein |
| 1691 | AGHZ01000466.1:1..1691 | Acyl-CoA thioesterase II (EC 3.1.2.-)  pathogenicity-related protein |
| 1681 | AGHZ01000560.1:1..1681 | Putative preQ0 transporter  Transporter |
| 1680 | AGHZ01000098.1:1..1680 | B. burgdorferi predicted coding region BB0646  Drug:proton antiporter |
| 1679 | AGHZ01000660.1:1..1679 | Cell division inhibitor  Putative exported protein |
| 1676 | AGHZ01000358.1:1..1676 | hypothetical protein |
| 1668 | AGHZ01000629.1:1..1668 | Inner membrane component of tripartite multidrug resistance system |
| 1667 | AGHZ01000882.1:1..1667 | 3-isopropylmalate dehydratase large subunit (EC 4.2.1.33) |
| 1666 | AGHZ01001126.1:1..1666 | Acriflavin resistance protein  hypothetical protein |
| 1665 | AGHZ01001043.1:1..1665 | Lipid A biosynthesis lauroyl acyltransferase (EC 2.3.1.-)  Phosphinothricin N-acetyltransferase (EC 2.3.1.-) |
| 1663 | AGHZ01000569.1:1..1663 | Methyl-accepting chemotaxis protein I (serine chemoreceptor protein) |
| 1660 | AGHZ01000633.1:1..1660 |  |
| 1659 | AGHZ01000493.1:1..1659 | Malate dehydrogenase (EC 1.1.1.37)  hypothetical protein |
| 1654 | AGHZ01000532.1:1..1654 | Cyclic di-GMP binding protein precursor |
| 1652 | AGHZ01000372.1:1..1652 | ECF sigma factor |
| 1647 | AGHZ01000041.1:1..1647 | aminopeptidase N  hypothetical protein |
| 1645 | AGHZ01000879.1:1..1645 | Serine phosphatase RsbU2C regulator of sigma subunit  anti-sigma F factor antagonist  hypothetical protein |
| 1643 | AGHZ01001232.1:1..1643 |  |
| 1643 | AGHZ01000567.1:1..1643 | FIG01085402: hypothetical protein |
| 1637 | AGHZ01000647.1:1..1637 | Uncharacterized membrane protein |
| 1635 | AGHZ01000566.1:1..1635 | D-beta-hydroxybutyrate dehydrogenase (EC 1.1.1.30)  Mutator mutT protein (72C8-dihydro-8-oxoguanine-triphosphatase) (EC 3.6.1.-) |
| 1635 | AGHZ01000486.1:1..1635 | Cytochrome C4  Cytochrome C552  FIG01111038: hypothetical protein |
| 1634 | AGHZ01000584.1:1..1634 | hypothetical protein |
| 1630 | AGHZ01000704.1:1..1630 | Putative benzaldehyde dehydrogenase oxidoreductase protein (EC 1.2.1.28)  Transcriptional regulator PobR2C AraC family |
| 1629 | AGHZ01001338.1:1..1629 |  |
| 1628 | AGHZ01001428.1:1..1628 | hypothetical protein |
| 1626 | AGHZ01000822.1:1..1626 |  |
| 1622 | AGHZ01001467.1:1..1622 | FIG01210022: hypothetical protein  Inner membrane protein |
| 1618 | AGHZ01000592.1:1..1618 | Methyl-accepting chemotaxis protein I (serine chemoreceptor protein) |
| 1612 | AGHZ01000898.1:1..1612 | ADP-ribose pyrophosphatase (EC 3.6.1.13)  hypothetical protein |
| 1610 | AGHZ01000699.1:1..1610 | tRNA-i(6)A37 methylthiotransferase |
| 1604 | AGHZ01000908.1:1..1604 | ECF sigma factor |
| 1599 | AGHZ01001069.1:1..1599 | hypothetical protein |
| 1594 | AGHZ01000043.1:1..1594 | Hemolysin activation/secretion protein associated with VreARI signalling system |
| 1589 | AGHZ01000580.1:1..1589 | DNA-binding response regulator2C LuxR family |
| 1588 | AGHZ01001258.1:1..1588 | RND efflux system2C outer membrane lipoprotein2C NodT family |
| 1588 | AGHZ01000574.1:1..1588 | Ferrous iron transport protein B  hypothetical protein |
| 1587 | AGHZ01001225.1:1..1587 | Ribonuclease BN (EC 3.1.-.-) |
| 1586 | AGHZ01001134.1:1..1586 | Rieske 2Fe-2S domain protein  diguanylate cyclase/phosphodiesterase (GGDEF |
| 1583 | AGHZ01000221.1:1..1583 |  |
| 1578 | AGHZ01001114.1:1..1578 |  |
| 1577 | AGHZ01000443.1:1..1577 | 3-oxoacyl-[acyl-carrier protein] reductase (EC 1.1.1.100) |
| 1575 | AGHZ01001323.1:1..1575 | Possible transmembrane protein |
| 1575 | AGHZ01001098.1:1..1575 |  |
| 1571 | AGHZ01000878.1:1..1571 |  |
| 1571 | AGHZ01000202.1:1..1571 |  |
| 1557 | AGHZ01000953.1:1..1557 |  |
| 1550 | AGHZ01000916.1:1..1550 | UDP-N-acetylmuramoylalanyl-D-glutamate--22C6-diaminopimelate ligase (EC 6.3.2.13) |
| 1548 | AGHZ01000941.1:1..1548 |  |
| 1546 | AGHZ01000636.1:1..1546 | Cell division topological specificity factor MinE  Septum site-determining protein MinC  Septum site-determining protein MinD |
| 1543 | AGHZ01000654.1:1..1543 | FIG01211823: hypothetical protein  RNA polymerase sigma-E factor |
| 1538 | AGHZ01001328.1:1..1538 | DNA-directed RNA polymerase specialized sigma subunit2C sigma24-like  probable RebB like protein |
| 1529 | AGHZ01001156.1:1..1529 | FIG01210697: hypothetical protein  TldE/PmbA protein2C part of proposed TldE/TldD proteolytic complex (PMID 12029038) |
| 1520 | AGHZ01000345.1:1..1520 | FIG140336: TPR domain protein |
| 1518 | AGHZ01001293.1:1..1518 | Inosine-uridine preferring nucleoside hydrolase (EC 3.2.2.1) |
| 1518 | AGHZ01000337.1:1..1518 | Methyl-accepting chemotaxis protein I (serine chemoreceptor protein)  Positive regulator of CheA protein activity (CheW) |
| 1516 | AGHZ01001502.1:1..1516 |  |
| 1515 | AGHZ01000863.1:1..1515 |  |
| 1514 | AGHZ01000931.1:1..1514 | Phosphoenolpyruvate carboxylase (EC 4.1.1.31)  Transcriptional regulator2C TetR family |
| 1504 | AGHZ01001543.1:1..1504 | 4-alpha-glucanotransferase (amylomaltase) (EC 2.4.1.25) |
| 1500 | AGHZ01000949.1:1..1500 | Phosphate starvation-inducible ATPase PhoH with RNA binding motif |
| 1499 | AGHZ01001308.1:1..1499 | extracellular protease  hypothetical protein |
| 1497 | AGHZ01000937.1:1..1497 | Flagellar hook-basal body complex protein FliE |
| 1496 | AGHZ01001083.1:1..1496 | Manganese superoxide dismutase (EC 1.15.1.1) |
| 1493 | AGHZ01000911.1:1..1493 | Malate permease |
| 1484 | AGHZ01000996.1:1..1484 | FIG023677: hypothetical protein |
| 1482 | AGHZ01001015.1:1..1482 | Distant homolog of E. coli HemX protein in Xanthomonadaceae |
| 1478 | AGHZ01000972.1:1..1478 | Response regulator containing a CheY-like receiver domain and a GGDEF domain |
| 1472 | AGHZ01000969.1:1..1472 | Aspartate-semialdehyde dehydrogenase (EC 1.2.1.11) |
| 1472 | AGHZ01000639.1:1..1472 | Biotin synthase (EC 2.8.1.6)  Competence protein F homolog2C phosphoribosyltransferase domain; protein YhgH required for utilization of DNA as sole source of carbon and energy |
| 1469 | AGHZ01000601.1:1..1469 | Mg(2 ) transport ATPase protein C |
| 1469 | AGHZ01000015.1:1..1469 | Phosphomannomutase (EC 5.4.2.8) / Phosphoglucomutase (EC 5.4.2.2) |
| 1468 | AGHZ01000939.1:1..1468 | FIG00960671: hypothetical protein |
| 1466 | AGHZ01000481.1:1..1466 | Phosphate regulon transcriptional regulatory protein PhoB (SphR)  Protease |
| 1462 | AGHZ01000950.1:1..1462 |  |
| 1462 | AGHZ01000858.1:1..1462 | Acyl-CoA thioester hydrolase  FIG01210044: hypothetical protein  hypothetical protein |
| 1462 | AGHZ01000522.1:1..1462 | hypothetical protein |
| 1459 | AGHZ01000930.1:1..1459 | Deoxyuridine 5'-triphosphate nucleotidohydrolase (EC 3.6.1.23)  Phosphopantothenoylcysteine decarboxylase (EC 4.1.1.36) / Phosphopantothenoylcysteine synthetase (EC 6.3.2.5) |
| 1458 | AGHZ01000464.1:1..1458 | Polysaccharide deacetylase2C caspase activity |
| 1450 | AGHZ01000327.1:1..1450 | TonB-dependent receptor |
| 1444 | AGHZ01000536.1:1..1444 |  |
| 1442 | AGHZ01000810.1:1..1442 | porin2C putative |
| 1441 | AGHZ01001104.1:1..1441 | FIG01209826: hypothetical protein  Methylmalonate-semialdehyde dehydrogenase [inositol] (EC 1.2.1.27)  hypothetical protein |
| 1441 | AGHZ01000831.1:1..1441 |  |
| 1437 | AGHZ01000961.1:1..1437 | Endoglucanase precursor (EC 3.2.1.4) |
| 1434 | AGHZ01001137.1:1..1434 | His repressor |
| 1433 | AGHZ01000171.1:1..1433 | Leucine-responsive regulatory protein2C regulator for leucine (or lrp) regulon and high-affinity branched-chain amino acid transport system  hypothetical protein  putative AtsE |
| 1432 | AGHZ01000915.1:1..1432 | Polygalacturonase (EC 3.2.1.15) |
| 1431 | AGHZ01000379.1:1..1431 | Alkaline phosphatase D |
| 1430 | AGHZ01001016.1:1..1430 | Galactose-binding protein regulator  predicted L-arabinose 1-dehydrogenase (EC 1.1.1.46) |
| 1429 | AGHZ01001095.1:1..1429 | Leucine dehydrogenase (EC 1.4.1.9) |
| 1429 | AGHZ01000499.1:1..1429 | Endoglucanase (EC 3.2.1.4) |
| 1427 | AGHZ01000904.1:1..1427 | Dihydrolipoamide acetyltransferase component of pyruvate dehydrogenase complex (EC 2.3.1.12) |
| 1426 | AGHZ01001298.1:1..1426 | Carbamoyl-phosphate synthase small chain (EC 6.3.5.5) |
| 1426 | AGHZ01000870.1:1..1426 | Hypothetical protein DUF1942C DegV family |
| 1423 | AGHZ01000431.1:1..1423 | UDP-N-acetylglucosamine--N-acetylmuramyl-(pentapeptide) pyrophosphoryl-undecaprenol N-acetylglucosamine transferase (EC 2.4.1.227) |
| 1420 | AGHZ01001249.1:1..1420 |  |
| 1417 | AGHZ01001180.1:1..1417 |  |
| 1413 | AGHZ01000935.1:1..1413 | FIG005080: Possible exported protein |
| 1411 | AGHZ01000019.1:1..1411 |  |
| 1409 | AGHZ01000275.1:1..1409 | FIG01210377: hypothetical protein |
| 1403 | AGHZ01000321.1:1..1403 | Homogentisate 12C2-dioxygenase (EC 1.13.11.5) |
| 1400 | AGHZ01000571.1:1..1400 | Ribosomal protein L11 methyltransferase (EC 2.1.1.-)  Transcriptional regulator pbsX family |
| 1391 | AGHZ01000983.1:1..1391 | CDP-diacylglycerol--serine O-phosphatidyltransferase (EC 2.7.8.8)  FIG01213248: hypothetical protein |
| 1391 | AGHZ01000748.1:1..1391 | Translation elongation factor Ts |
| 1387 | AGHZ01001288.1:1..1387 |  |
| 1386 | AGHZ01001075.1:1..1386 | FIG01095481: hypothetical protein |
| 1384 | AGHZ01001373.1:1..1384 | Optional hypothetical component of the B12 transporter BtuM |
| 1384 | AGHZ01000650.1:1..1384 | RND multidrug efflux transporter; Acriflavin resistance protein |
| 1382 | AGHZ01001033.1:1..1382 |  |
| 1381 | AGHZ01001666.1:1..1381 | Transcriptional regulator2C MarR family |
| 1374 | AGHZ01001589.1:1..1374 |  |
| 1372 | AGHZ01001424.1:1..1372 | Lactate-responsive regulator LldR in Enterobacteria2C GntR family |
| 1372 | AGHZ01001401.1:1..1372 | 1-deoxy-D-xylulose 5-phosphate synthase (EC 2.2.1.7)  GumN protein |
| 1371 | AGHZ01000371.1:1..1371 | COG21102C Macro domain2C possibly ADP-ribose binding module |
| 1367 | AGHZ01000139.1:1..1367 | Lipid A biosynthesis lauroyl acyltransferase (EC 2.3.1.-)  membrane protein |
| 1365 | AGHZ01000922.1:1..1365 | Phosphoadenylyl-sulfate reductase [thioredoxin] (EC 1.8.4.8)  Sulfite reductase [NADPH] hemoprotein beta-component (EC 1.8.1.2) |
| 1364 | AGHZ01001448.1:1..1364 | Chromosome (plasmid) partitioning protein ParA / Sporulation initiation inhibitor protein Soj |
| 1364 | AGHZ01000859.1:1..1364 | Cell wall-associated hydrolases (invasion-associated proteins) |
| 1363 | AGHZ01000874.1:1..1363 | sigma-54 dependent DNA-binding response regulator |
| 1350 | AGHZ01000675.1:1..1350 | putative sigma-54-dependent transcriptional regulator |
| 1350 | AGHZ01000267.1:1..1350 | 2-C-methyl-D-erythritol 22C4-cyclodiphosphate synthase (EC 4.6.1.12)  2-C-methyl-D-erythritol 4-phosphate cytidylyltransferase (EC 2.7.7.60) |
| 1342 | AGHZ01000489.1:1..1342 | rRNA small subunit methyltransferase I |
| 1341 | AGHZ01000724.1:1..1341 | FIG01212489: hypothetical protein |
| 1341 | AGHZ01000450.1:1..1341 | Phosphatidate cytidylyltransferase (EC 2.7.7.41)  Undecaprenyl pyrophosphate synthetase (EC 2.5.1.31) |
| 1338 | AGHZ01000495.1:1..1338 | Dienelactone hydrolase family protein  UDP-N-acetylmuramoylalanine--D-glutamate ligase (EC 6.3.2.9) |
| 1333 | AGHZ01001018.1:1..1333 | Aspartate carbamoyltransferase (EC 2.1.3.2) |
| 1331 | AGHZ01001203.1:1..1331 | Exodeoxyribonuclease V gamma chain (EC 3.1.11.5) |
| 1331 | AGHZ01000998.1:1..1331 | Phosphoribosylanthranilate isomerase (EC 5.3.1.24)  tRNA pseudouridine synthase A (EC 4.2.1.70) |
| 1329 | AGHZ01000354.1:1..1329 | Signal transduction histidine kinase CheA (EC 2.7.3.-) |
| 1325 | AGHZ01000912.1:1..1325 | Peptide chain release factor 1 |
| 1321 | AGHZ01000568.1:1..1321 | Acid phosphatase (EC 3.1.3.2) |
| 1314 | AGHZ01001053.1:1..1314 | Thymidine phosphorylase (EC 2.4.2.4) |
| 1310 | AGHZ01000840.1:1..1310 |  |
| 1309 | AGHZ01000929.1:1..1309 | phosphoanhydride phosphohydrolase |
| 1305 | AGHZ01000460.1:1..1305 |  |
| 1304 | AGHZ01000881.1:1..1304 |  |
| 1302 | AGHZ01000697.1:1..1302 |  |
| 1300 | AGHZ01001551.1:1..1300 |  |
| 1297 | AGHZ01000803.1:1..1297 | ferrous iron transport protein |
| 1295 | AGHZ01000659.1:1..1295 |  |
| 1293 | AGHZ01000194.1:1..1293 | hypothetical protein |
| 1292 | AGHZ01000694.1:1..1292 | Phenazine biosynthesis protein PhzF like  hypothetical protein |
| 1289 | AGHZ01000605.1:1..1289 | Homoserine kinase (EC 2.7.1.39)  hypothetical protein |
| 1287 | AGHZ01001302.1:1..1287 |  |
| 1286 | AGHZ01001005.1:1..1286 |  |
| 1282 | AGHZ01000713.1:1..1282 | tRNA-guanine transglycosylase (EC 2.4.2.29) |
| 1281 | AGHZ01000315.1:1..1281 | Low molecular weight protein tyrosine phosphatase (EC 3.1.3.48) |
| 1279 | AGHZ01001222.1:1..1279 | Ribosomal small subunit pseudouridine synthase A (EC 4.2.1.70)  ribosomal RNA small subunit methyltransferase C |
| 1277 | AGHZ01000795.1:1..1277 | 1-deoxy-D-xylulose 5-phosphate reductoisomerase (EC 1.1.1.267) |
| 1277 | AGHZ01000698.1:1..1277 | Transcriptional regulator2C LysR family |
| 1276 | AGHZ01001548.1:1..1276 |  |
| 1276 | AGHZ01000905.1:1..1276 | 1-pyrroline-4-hydroxy-2-carboxylate deaminase (EC 3.5.4.22) |
| 1274 | AGHZ01001616.1:1..1274 |  |
| 1272 | AGHZ01000563.1:1..1272 | Dihydrolipoamide acyltransferase component of branched-chain alpha-keto acid dehydrogenase complex (EC 2.3.1.168) |
| 1267 | AGHZ01000799.1:1..1267 | hypothetical protein |
| 1261 | AGHZ01001107.1:1..1261 | FIG01210006: hypothetical protein  FIG01217340: hypothetical protein |
| 1261 | AGHZ01000641.1:1..1261 |  |
| 1260 | AGHZ01001301.1:1..1260 | Coenzyme PQQ synthesis protein D |
| 1257 | AGHZ01001623.1:1..1257 | Putative lipase in cluster with Phosphatidate cytidylyltransferase |
| 1257 | AGHZ01001206.1:1..1257 | Cytochrome c heme lyase subunit CcmF  Cytochrome c-type biogenesis protein CcmG/DsbE2C thiol:disulfide oxidoreductase |
| 1254 | AGHZ01001029.1:1..1254 | DUF124 domain-containing protein |
| 1251 | AGHZ01001289.1:1..1251 |  |
| 1246 | AGHZ01000527.1:1..1246 |  |
| 1244 | AGHZ01000887.1:1..1244 | Cysteine desulfurase (EC 2.8.1.7)  L-proline glycine betaine binding ABC transporter protein ProX (TC 3.A.1.12.1) / Osmotic adaptation |
| 1243 | AGHZ01001149.1:1..1243 | Lysophospholipid acyltransferase |
| 1243 | AGHZ01000951.1:1..1243 | Iojap protein  hypothetical protein |
| 1240 | AGHZ01001330.1:1..1240 |  |
| 1237 | AGHZ01001470.1:1..1237 | Cytochrome c-type biogenesis protein DsbD2C protein-disulfide reductase (EC 1.8.1.8) |
| 1237 | AGHZ01001177.1:1..1237 | two-component system regulatory protein |
| 1237 | AGHZ01000897.1:1..1237 | ATP-dependent RNA helicase RhlB  Cell division transporter2C ATP-binding protein FtsE (TC 3.A.5.1.1) |
| 1227 | AGHZ01001370.1:1..1227 |  |
| 1227 | AGHZ01000888.1:1..1227 |  |
| 1223 | AGHZ01001661.1:1..1223 | RNA polymerase sigma-54 factor RpoN |
| 1223 | AGHZ01000279.1:1..1223 | Quinolinate phosphoribosyltransferase [decarboxylating] (EC 2.4.2.19) |
| 1216 | AGHZ01001218.1:1..1216 | Sigma-fimbriae tip adhesin |
| 1216 | AGHZ01001129.1:1..1216 | FIG01212343: hypothetical protein |
| 1215 | AGHZ01001178.1:1..1215 | Aspartate racemase (EC 5.1.1.13) |
| 1215 | AGHZ01001039.1:1..1215 |  |
| 1214 | AGHZ01001025.1:1..1214 |  |
| 1212 | AGHZ01000355.1:1..1212 | Exodeoxyribonuclease V beta chain (EC 3.1.11.5) |
| 1209 | AGHZ01001266.1:1..1209 |  |
| 1206 | AGHZ01001133.1:1..1206 | Phosphate transport system regulatory protein PhoU |
| 1206 | AGHZ01000003.1:1..1206 | ISXoo3 transposase orfB |
| 1204 | AGHZ01001117.1:1..1204 | Dehydrogenases with different specificities (related to short-chain alcohol dehydrogenases) |
| 1204 | AGHZ01000940.1:1..1204 | SSU ribosomal protein S2p (SAe) |
| 1204 | AGHZ01000197.1:1..1204 | IS1404 transposase  ISXoo3 transposase orfA |
| 1199 | AGHZ01001391.1:1..1199 |  |
| 1198 | AGHZ01001215.1:1..1198 | TonB protein |
| 1196 | AGHZ01000196.1:1..1196 |  |
| 1195 | AGHZ01001124.1:1..1195 | FIG01210307: hypothetical protein |
| 1189 | AGHZ01000586.1:1..1189 | Glycosyltransferase |
| 1187 | AGHZ01001572.1:1..1187 | FIG01212309: hypothetical protein  Permeases of the drug/metabolite transporter (DMT) superfamily  Permeases of the drug/metabolite transporter (DMT) superfamily |
| 1183 | AGHZ01001110.1:1..1183 |  |
| 1181 | AGHZ01000926.1:1..1181 | hypothetical protein  putative 6-aminohexanoate-dimer hydrolase |
| 1181 | AGHZ01000894.1:1..1181 | COGs COG3146 |
| 1170 | AGHZ01001334.1:1..1170 |  |
| 1167 | AGHZ01000987.1:1..1167 | hypothetical protein  hypothetical protein |
| 1165 | AGHZ01001277.1:1..1165 | hypothetical protein |
| 1164 | AGHZ01001295.1:1..1164 |  |
| 1162 | AGHZ01000762.1:1..1162 | conserved hypothetical protein |
| 1161 | AGHZ01000837.1:1..1161 | MFS transporter |
| 1160 | AGHZ01001383.1:1..1160 |  |
| 1159 | AGHZ01000239.1:1..1159 |  |
| 1152 | AGHZ01001002.1:1..1152 | ATPase YjeE2C predicted to have essential role in cell wall biosynthesis |
| 1148 | AGHZ01001406.1:1..1148 | transcriptional regulator2C putative |
| 1148 | AGHZ01001136.1:1..1148 |  |
| 1142 | AGHZ01000587.1:1..1142 | hypothetical protein |
| 1141 | AGHZ01000384.1:1..1141 | putative; ORF located using Glimmer/Genemark |
| 1131 | AGHZ01001436.1:1..1131 |  |
| 1130 | AGHZ01001052.1:1..1130 | 3-dehydroquinate dehydratase II (EC 4.2.1.10) |
| 1127 | AGHZ01000229.1:1..1127 | two-component system sensor protein |
| 1123 | AGHZ01001007.1:1..1123 | SAM-dependent methyltransferase |
| 1116 | AGHZ01001402.1:1..1116 | FIG00537784: hypothetical protein  Transcriptional regulator2C LuxR family |
| 1116 | AGHZ01000062.1:1..1116 | FIG01210236: hypothetical protein |
| 1115 | AGHZ01000780.1:1..1115 | hypothetical protein  hypothetical protein |
| 1112 | AGHZ01001024.1:1..1112 | FIG01211473: hypothetical protein |
| 1111 | AGHZ01001224.1:1..1111 |  |
| 1111 | AGHZ01000208.1:1..1111 |  |
| 1110 | AGHZ01000612.1:1..1110 | Lipoprotein |
| 1108 | AGHZ01001693.1:1..1108 | Glutathione S-transferase (EC 2.5.1.18)  Transcriptional regulator2C DeoR family |
| 1108 | AGHZ01000880.1:1..1108 |  |
| 1105 | AGHZ01000787.1:1..1105 | Deoxycytidine triphosphate deaminase (EC 3.5.4.13) |
| 1104 | AGHZ01001405.1:1..1104 |  |
| 1103 | AGHZ01001354.1:1..1103 |  |
| 1103 | AGHZ01000746.1:1..1103 | ABC transporter related |
| 1102 | AGHZ01001657.1:1..1102 | Helicase PriA essential for oriC/DnaA-independent DNA replication  hypothetical protein |
| 1100 | AGHZ01000480.1:1..1100 | hypothetical protein |
| 1099 | AGHZ01001145.1:1..1099 |  |
| 1099 | AGHZ01000433.1:1..1099 | hypothetical protein |
| 1094 | AGHZ01001362.1:1..1094 | BatA (Bacteroides aerotolerance operon) |
| 1094 | AGHZ01000676.1:1..1094 | FIG01212518: hypothetical protein |
| 1094 | AGHZ01000252.1:1..1094 | Flagellar protein FliJ  Flagellum-specific ATP synthase FliI |
| 1091 | AGHZ01000262.1:1..1091 | Predicted endonuclease distantly related to archaeal Holliday junction resolvase  Putative membrane-bound metal-dependent hydrolases |
| 1090 | AGHZ01001462.1:1..1090 | FIG006285: hypothetical protein |
| 1087 | AGHZ01001284.1:1..1087 | 4-carboxymuconolactone decarboxylase domain/alkylhydroperoxidase AhpD family core domain protein |
| 1083 | AGHZ01001122.1:1..1083 |  |
| 1080 | AGHZ01000280.1:1..1080 | Outer membrane component of tripartite multidrug resistance system |
| 1077 | AGHZ01000325.1:1..1077 |  |
| 1074 | AGHZ01000975.1:1..1074 |  |
| 1073 | AGHZ01000339.1:1..1073 | ATP dependent RNA helicase |
| 1068 | AGHZ01001541.1:1..1068 | FIG000233: metal-dependent hydrolase  hypothetical protein  hypothetical protein |
| 1067 | AGHZ01001510.1:1..1067 |  |
| 1061 | AGHZ01001331.1:1..1061 | FIG01210489: hypothetical protein  hypothetical protein |
| 1060 | AGHZ01000923.1:1..1060 |  |
| 1060 | AGHZ01000896.1:1..1060 |  |
| 1059 | AGHZ01001557.1:1..1059 |  |
| 1056 | AGHZ01001345.1:1..1056 | Free methionine-(R)-sulfoxide reductase2C contains GAF domain |
| 1053 | AGHZ01000628.1:1..1053 |  |
| 1052 | AGHZ01000877.1:1..1052 | hypothetical protein  hypothetical protein |
| 1052 | AGHZ01000342.1:1..1052 | probable membrane protein STY4873 |
| 1050 | AGHZ01000993.1:1..1050 | Alcohol dehydrogenase (EC 1.1.1.1)  twitching motility protein PilH |
| 1047 | AGHZ01000200.1:1..1047 | hypothetical protein |
| 1038 | AGHZ01001442.1:1..1038 | Plasmid replication/partition related protein |
| 1038 | AGHZ01001038.1:1..1038 | Catalase (EC 1.11.1.6) |
| 1030 | AGHZ01001575.1:1..1030 | AcrA/AcrE family protein |
| 1030 | AGHZ01000994.1:1..1030 |  |
| 1029 | AGHZ01001364.1:1..1029 | RNA polymerase sigma-70 factor |
| 1028 | AGHZ01000721.1:1..1028 | Citrate synthase (si) (EC 2.3.3.1)  SOS-response repressor and protease LexA (EC 3.4.21.88) |
| 1027 | AGHZ01000610.1:1..1027 |  |
| 1026 | AGHZ01001123.1:1..1026 | CDP-diacylglycerol--serine O-phosphatidyltransferase (EC 2.7.8.8) |
| 1026 | AGHZ01001070.1:1..1026 |  |
| 1026 | AGHZ01000679.1:1..1026 | Magnesium and cobalt efflux protein CorC |
| 1026 | AGHZ01000653.1:1..1026 | hypothetical protein |
| 1021 | AGHZ01001001.1:1..1021 | outer membrane protein |
| 1018 | AGHZ01001113.1:1..1018 |  |
| 1018 | AGHZ01000766.1:1..1018 | FIG01210453: hypothetical protein  FIG01211156: hypothetical protein |
| 1012 | AGHZ01001416.1:1..1012 |  |
| 1008 | AGHZ01001020.1:1..1008 | cardiolipin synthase |
| 1007 | AGHZ01001437.1:1..1007 |  |
| 1007 | AGHZ01001339.1:1..1007 |  |
| 1007 | AGHZ01000814.1:1..1007 |  |
| 1005 | AGHZ01001227.1:1..1005 |  |
| 1002 | AGHZ01000336.1:1..1002 | hypothetical protein |
| 1001 | AGHZ01001071.1:1..1001 |  |
| 1001 | AGHZ01001004.1:1..1001 | FIG01211600: hypothetical protein  hypothetical protein |
