# Supplementary material for: Draft Genome Sequences of Xanthomonas sacchari and Two Banana-Associated Xanthomonads Reveal Insights into the Xanthomonas Group 1 Clade
Source: Genes (Basel). 2011 Dec 2;2(4):1050–65. doi: 10.3390/genes2041050 (PMC3927605; doi:10.3390/genes2041050)
Supplement: Supplementary File 1 — ZIP-Document (ZIP, 7075 KB) [file genes-02-01050-s001.zip › genes-11371-supplementary/NCPPB1132_genes_comparison.pdf]

Gene	Location	NCPPB1131	NCPPB1132	NCPPB4393
fig|487528.5.peg.1873 Endonuclease precursor	AGHZ01000430.1:6749..9697	✗ 0.00	✓ 1.00	✗ 0.00
fig|487528.5.peg.2750 hypothetical protein	AGHZ01000185.1:3393..2107	✗ 0.00	✓ 1.00	✗ 0.00
fig|487528.5.peg.355 hypothetical protein	AGHZ01001350.1:49..264	✗ 0.00	✓ 1.00	✓ 0.74
fig|487528.5.peg.3712 possible DNA helicase	AGHZ01000018.1:19825..17969	✗ 0.01	✓ 1.00	✗ 0.00
fig|487528.5.peg.81 hypothetical protein	AGHZ01001647.1:6083..6559	✗ 0.01	✓ 1.00	✗ 0.32
fig|487528.5.peg.2533 hypothetical protein	AGHZ01000243.1:3863..4087	✗ 0.01	✓ 1.00	✓ 0.95
fig|487528.5.peg.3373 Salicylate hydroxylase (EC 1.14.13.1);Ontology_term=KEGG_ENZYME:1.14.13.1	AGHZ01000076.1:1278..2219	✗ 0.01	✓ 1.00	✓ 0.80
fig|487528.5.peg.2172 hypothetical protein	AGHZ01000340.1:5995..5240	✗ 0.01	✓ 1.00	✓ 1.00
fig|487528.5.peg.2574 hypothetical protein	AGHZ01000230.1:4437..4282	✗ 0.01	✓ 1.00	✓ 1.00
fig|487528.5.peg.2588 Type I restriction-modification system2C specificity subunit S (EC 3.1.21.3);Ontology_term=KEGG_ENZYM	AGHZ01000227.1:6618..7931	✗ 0.01	✓ 1.00	✗ 0.00
fig|487528.5.peg.1756 hypothetical protein	AGHZ01000472.1:536..408	✗ 0.02	✓ 1.00	! 0.43
fig|487528.5.peg.3312 hypothetical protein	AGHZ01000085.1:756..5105	✗ 0.02	✓ 1.00	✗ 0.00
fig|487528.5.peg.3272 Long-chain-fatty-acid--CoA ligase (EC 6.2.1.3);Ontology_term=KEGG_ENZYME:6.2.1.3	AGHZ01000092.1:14450..10332	✗ 0.02	✓ 1.00	✗ 0.00
fig|487528.5.peg.3454 oxidoreductase	AGHZ01000057.1:1513..206	✗ 0.02	✓ 1.00	✗ 0.18
fig|487528.5.peg.110 hypothetical protein	AGHZ01001628.1:1561..1707	✗ 0.02	✓ 1.00	✓ 1.00
fig|487528.5.peg.2398 hypothetical protein	AGHZ01000282.1:1426..1554	✗ 0.02	✓ 1.00	✓ 1.00
fig|487528.5.peg.1592 TonB-dependent receptor	AGHZ01000534.1:3864..6755	✗ 0.02	✓ 1.00	✗ 0.00
fig|487528.5.peg.1489 TonB-dependent receptor	AGHZ01000581.1:3632..747	✗ 0.02	✓ 1.00	✗ 0.00
fig|487528.5.peg.3601 TonB-dependent receptor	AGHZ01000037.1:5760..8672	✗ 0.02	✓ 1.00	✓ 1.00
fig|487528.5.peg.2849 Acetyltransferase2C GNAT family (EC 2.3.1.-);Ontology_term=KEGG_ENZYME:2.3.1.-	AGHZ01000168.1:7188..7760	✗ 0.02	✓ 1.00	✓ 0.99
fig|487528.5.peg.2897 TonB-dependent receptor	AGHZ01000158.1:7420..4730	✗ 0.03	✓ 1.00	✓ 0.99
fig|487528.5.peg.2705 hypothetical protein	AGHZ01000191.1:435..319	✗ 0.03	✓ 1.00	✓ 1.00
fig|487528.5.peg.3646 TonB-dependent receptor	AGHZ01000030.1:165..2849	✗ 0.03	✓ 1.00	✓ 1.00
fig|487528.5.peg.2496 hypothetical protein	AGHZ01000255.1:3442..812	✗ 0.03	✓ 1.00	✗ 0.00
fig|487528.5.peg.785 hypothetical protein	AGHZ01000951.1:40..153	✗ 0.03	✓ 1.00	✓ 1.00
fig|487528.5.peg.626 hypothetical protein	AGHZ01001049.1:5232..4849	✗ 0.03	✓ 1.00	✗ 0.19
fig|487528.5.peg.3747 glycosyl transferase2C family 2	AGHZ01000013.1:15898..13601	✗ 0.03	✓ 1.00	✗ 0.00
fig|487528.5.peg.3375 Penicillin amidase family protein	AGHZ01000076.1:7196..4881	✗ 0.03	✓ 1.00	✗ 0.00
fig|487528.5.peg.3713 FIG00901053: hypothetical protein	AGHZ01000018.1:22121..19827	✗ 0.03	✓ 1.00	✗ 0.00
fig|487528.5.peg.2791 FIG01211915: hypothetical protein	AGHZ01000177.1:2..2281	✗ 0.03	✓ 1.00	✓ 0.99
fig|487528.5.peg.3628 FIG00955472: hypothetical protein	AGHZ01000033.1:11261..13465	✗ 0.03	✓ 1.00	✗ 0.00
fig|487528.5.peg.3720 colicin V secretion ABC transporter ATP-binding protein	AGHZ01000017.1:8962..6851	✗ 0.03	✓ 1.00	✗ 0.00
fig|487528.5.peg.2468 ADP-ribosylglycohydrolase	AGHZ01000263.1:5369..5746	✗ 0.03	✓ 1.00	✗ 0.03
fig|487528.5.peg.1944 hypothetical protein	AGHZ01000411.1:895..437	✗ 0.03	✓ 1.00	✓ 1.00
fig|487528.5.peg.3409 Glucoamylase (EC 3.2.1.3);Ontology_term=KEGG_ENZYME:3.2.1.3	AGHZ01000069.1:5825..3972	✗ 0.04	✓ 1.00	✓ 1.00
fig|487528.5.peg.2432 hypothetical protein	AGHZ01000277.1:6394..4532	✗ 0.04	✓ 1.00	✗ 0.00
fig|487528.5.peg.3104 Phage T7 exclusion protein	AGHZ01000119.1:8202..9974	✗ 0.04	✓ 1.00	✗ 0.00
fig|487528.5.peg.2169 FIG01211701: hypothetical protein	AGHZ01000340.1:3540..1846	✗ 0.04	✓ 1.00	✓ 1.00
fig|487528.5.peg.2173 coproporphyrinogen III oxidase2C putative	AGHZ01000340.1:7350..6001	✗ 0.04	✓ 1.00	✓ 1.00
fig|487528.5.peg.1878 DNA-cytosine methyltransferase (EC 2.1.1.37);Ontology_term=KEGG_ENZYME:2.1.1.37	AGHZ01000430.1:16626..15022	✗ 0.04	✓ 1.00	✗ 0.00
fig|487528.5.peg.2422 hypothetical protein	AGHZ01000278.1:2879..3109	✗ 0.04	✓ 1.00	! 0.65
fig|487528.5.peg.2784 protease	AGHZ01000178.1:11744..13327	✗ 0.04	✓ 1.00	✗ 0.00
fig|487528.5.peg.3647 flavin monoamine oxidase-related protein	AGHZ01000030.1:3058..4632	✗ 0.04	✓ 1.00	✓ 1.00

fig|487528.5.peg.1872 FIG00715517: hypothetical protein	AGHZ01000430.1:5235..6752	✗	0.05	✓	1.00	✗	0.00
fig|487528.5.peg.2621 Rrf2-linked NADH-flavin reductase	AGHZ01000218.1:3386..3841	✗	0.05	✓	1.00	✗	0.00
fig|487528.5.peg.3666 hypothetical protein	AGHZ01000027.1:9039..8911	✗	0.05	✓	1.00	✓	1.00
fig|487528.5.peg.645 tRNA(Ile)-lysidine synthetase	AGHZ01001036.1:609..358	✗	0.05	✓	1.00	✓	1.00
fig|487528.5.peg.2658 hypothetical protein	AGHZ01000210.1:2120..684	✗	0.05	✓	1.00	✓	0.96
fig|487528.5.peg.3704 hypothetical protein	AGHZ01000018.1:5538..5723	✗	0.05	✓	1.00	✓	1.00
fig|487528.5.peg.2623 drug resistance transporter2C EmrB/QacA subfamily	AGHZ01000218.1:4651..6054	✗	0.05	✓	1.00	✗	0.00
fig|487528.5.peg.2640 HipA protein	AGHZ01000214.1:2290..3603	✗	0.05	✓	1.00	✗	0.00
fig|487528.5.peg.3741 FIG01211631: hypothetical protein	AGHZ01000013.1:3159..4349	✗	0.06	✓	1.00	✗	0.00
fig|487528.5.peg.1590 prolyl oligopeptidase family protein	AGHZ01000534.1:1222..3219	✗	0.07	✓	1.00	✗	0.00
fig|487528.5.peg.3766 alginate biosynthesis protein	AGHZ01000009.1:6082..5087	✗	0.07	✓	1.00	✗	0.00
fig|487528.5.peg.1979 site-specific recombinase	AGHZ01000400.1:3200..2247	✗	0.07	✓	1.00	✗	0.00
fig|487528.5.peg.3500 plasmid mobilization protein	AGHZ01000049.1:13040..14680	✗	0.08	✓	1.00	✗	0.00
fig|487528.5.peg.2113 phage-related integrase	AGHZ01000356.1:10633..12363	✗	0.10	✓	1.00	✗	0.00
fig|487528.5.peg.3564 hypothetical protein	AGHZ01000039.1:6265..7047	✗	0.10	✓	1.00	✗	0.00
fig|487528.5.peg.3106 FIG014574: hypothetical protein	AGHZ01000119.1:11375..12025	✗	0.11	✓	1.00	✗	0.00
fig|487528.5.peg.2589 DNA repair protein RadC	AGHZ01000227.1:8249..7950	✗	0.11	✓	1.00	✗	0.00
fig|487528.5.peg.2384 hypothetical protein	AGHZ01000285.1:7172..7339	✗	0.17	✓	1.00	✗	0.00
fig|487528.5.peg.3460 Glucose-1-phosphate thymidylyltransferase (EC 2.7.7.24);Ontology_term=KEGG_ENZYME:2.7.7.24	AGHZ01000057.1:7050..7937	✗	0.19	✓	1.00	✗	0.00
fig|487528.5.peg.2056 hypothetical protein	AGHZ01000377.1:8485..8207	✗	0.25	✓	1.00	✗	0.00
fig|487528.5.peg.3663 hypothetical protein	AGHZ01000027.1:6036..5761	✗	0.25	✓	1.00	✗	0.00
fig|487528.5.peg.3111 hypothetical protein	AGHZ01000119.1:16761..17027	✗	0.26	✓	1.00	✗	0.00
fig|487528.5.peg.3745 hypothetical protein	AGHZ01000013.1:10920..10762	!	0.36	✓	1.00	✗	0.00
fig|487528.5.peg.1442 hypothetical protein	AGHZ01000604.1:2290..1619	!	0.44	✓	1.00	✗	0.00
fig|487528.5.peg.2724 FIG01209702: hypothetical protein	AGHZ01000190.1:12080..12337	!	0.45	✓	1.00	✗	0.00
fig|487528.5.peg.2380 Sensory box sensor histidine kinase/response regulator	AGHZ01000285.1:2216..4273	!	0.49	✓	1.00	✗	0.00
fig|487528.5.peg.231 Transcriptional regulator2C AraC family	AGHZ01001522.1:7359..7883	!	0.54	✓	1.00	✗	0.00
fig|487528.5.peg.2532 hypothetical protein	AGHZ01000243.1:2755..3126	!	0.59	✓	1.00	✗	0.00
fig|487528.5.peg.3461 dTDP-4-dehydrorhamnose 32C5-epimerase (EC 5.1.3.13);Ontology_term=KEGG_ENZYME:5.1.3.13	AGHZ01000057.1:7934..8491	!	0.63	✓	1.00	✗	0.00
fig|487528.5.peg.2788 hypothetical protein	AGHZ01000178.1:19219..19085	!	0.64	✓	1.00	✗	0.00
fig|487528.5.peg.2215 Hypothetical ABC transport system2C periplasmic component	AGHZ01000332.1:861..184	✓	0.69	✓	1.00	✗	0.00
fig|487528.5.peg.2266 conserved hypothetical protein	AGHZ01000320.1:5582..5175	✓	0.73	✓	1.00	✗	0.00
fig|487528.5.peg.2381 hypothetical protein	AGHZ01000285.1:4726..4340	✓	0.75	✓	1.00	✗	0.00
fig|487528.5.peg.2531 hypothetical protein	AGHZ01000243.1:2407..2751	✓	0.77	✓	1.00	✗	0.00
fig|487528.5.peg.1881 Putative outer membrane or secreted lipoprotein	AGHZ01000429.1:435..1196	✓	0.77	✓	1.00	✗	0.00
fig|487528.5.peg.2297 YoeB toxin protein	AGHZ01000312.1:1335..1589	✓	0.78	✓	1.00	✗	0.00
fig|487528.5.peg.1441 Transcriptional regulator2C LysR family	AGHZ01000604.1:474..1565	✓	0.81	✓	1.00	✗	0.00
fig|487528.5.peg.2267 Transcriptional regulator2C AraC family	AGHZ01000320.1:6495..5638	✓	0.84	✓	1.00	✗	0.00
fig|487528.5.peg.3044 hypothetical protein	AGHZ01000133.1:2305..2601	✓	0.86	✓	1.00	✗	0.02
fig|487528.5.peg.2382 hypothetical protein	AGHZ01000285.1:5093..5746	✓	0.89	✓	1.00	✗	0.00
fig|487528.5.peg.910 TonB-dependent receptor	AGHZ01000865.1:13564..10685	✓	0.92	✓	1.00	✗	0.03
fig|487528.5.peg.1321 hypothetical protein	AGHZ01000671.1:1099..1359	✓	1.00	✓	1.00	✗	0.00
fig|487528.5.peg.2385 hypothetical protein	AGHZ01000285.1:7547..8146	✓	1.00	✓	1.00	✗	0.00
fig|487528.5.peg.678 hypothetical protein	AGHZ01001021.1:5016..5879	✓	1.00	✓	1.00	✗	0.00
