# Supplementary material for: Draft Genome Sequences of Xanthomonas sacchari and Two Banana-Associated Xanthomonads Reveal Insights into the Xanthomonas Group 1 Clade
Source: Genes (Basel). 2011 Dec 2;2(4):1050–65. doi: 10.3390/genes2041050 (PMC3927605; doi:10.3390/genes2041050)
Supplement: Supplementary File 1 — ZIP-Document (ZIP, 7075 KB) [file genes-02-01050-s001.zip › genes-11371-supplementary/NCPPB4393-sequences-not-in-X_albilineans.html]

Regions of the Xanthomonas sacchari NCPPB4393 genome that show no detectable nucleotide sequence similarity with X. albilineans


### Regions of the *Xanthomonas sacchari* NCPPB4393 genome that show no detectable nucleotide sequence similarity with *X. albilineans*

No hits against these regions were found when performing *blastn* searches against the genome of *X. albilineans* GPE PC73 (RefSeq:NC\_013722), using the NCPPB4393 draft genome assembly as the query.

| Length of region (nucleotides) | GenBank accession and coordinates of region | Predicted genes in this region (using RAST: Aziz et al. 2008 BMC Genomics 9:75) |
| --- | --- | --- |
| 63509 | AGDB01000023.1:1..63509 | ABC transporter ATP-binding protein  ABC-type antimicrobial peptide transport system2C permease component  Diaminobutyrate-pyruvate aminotransferase (EC 2.6.1.46)  FIG01111875: hypothetical protein  FIG01211092: hypothetical protein  FIG01211473: hypothetical protein  FIG01212747: hypothetical protein  Methionine ABC transporter ATP-binding protein  Peptide synthetase  Predicted zinc-binding protein  Putative phage replication protein RstA  Radical SAM domain protein  Rhs family protein  Salicylate hydroxylase (EC 1.14.13.1)  SyrP-like protein  Thioesterase  TniA putative transposase  conserved hypothetical protein  hypothetical protein  hypothetical protein  hypothetical protein  hypothetical protein  hypothetical protein  hypothetical protein  hypothetical protein  hypothetical protein  hypothetical protein  hypothetical protein  hypothetical protein  hypothetical protein  phage integrase family protein  putative phospholipase  putative; ORF located using Glimmer/Genemark  transcriptional regulator2C LysR family |
| 62317 | AGDB01000377.1:1..62317 | 22C3-dihydroxy-22C3-dihydro-phenylpropionate dehydrogenase (EC 1.3.1.-)  3-carboxy-cis2Ccis-muconate cycloisomerase (EC 5.5.1.2)  3-oxoadipate CoA-transferase subunit A (EC 2.8.3.6)  3-oxoadipate CoA-transferase subunit B (EC 2.8.3.6)  4-carboxymuconolactone decarboxylase (EC 4.1.1.44)  Acetyl-CoA acetyltransferase (EC 2.3.1.9) @ Beta-ketoadipyl CoA thiolase (EC 2.3.1.-)  Adenylate cyclase (EC 4.6.1.1)  Beta-ketoadipate enol-lactone hydrolase (EC 3.1.1.24)  Biotin carboxylase (EC 6.3.4.14)  COG1073: Hydrolases of the alpha/beta superfamily  Cupin domain-containing protein  Dipeptidyl carboxypeptidase Dcp (EC 3.4.15.5)  ECF sigma factor  FIG01113202: hypothetical protein  FIG01210302: hypothetical protein  FIG01210342: hypothetical protein  Feruloyl-CoA synthetase  Flavodoxin reductases (ferredoxin-NADPH reductases) family 1; Vanillate O-demethylase oxidoreductase (EC 1.14.13.-)  Glyoxalase family protein  Hydrolase or peptidase  Isoaspartyl aminopeptidase (EC 3.4.19.5) @ Asp-X dipeptidase  Isocitrate lyase (EC 4.1.3.1)  Malate synthase (EC 2.3.3.9)  Outer membrane protein romA  Oxidoreductase  P-hydroxybenzoate hydroxylase (EC 1.14.13.2)  Permeases of the major facilitator superfamily  Probable FERULIC acid hydratase protein  Protocatechuate 32C4-dioxygenase alpha chain (EC 1.13.11.3)  Protocatechuate 32C4-dioxygenase beta chain (EC 1.13.11.3)  Putative benzaldehyde dehydrogenase oxidoreductase protein (EC 1.2.1.28)  Putative oxidoreductase YncB  Ribosomal large subunit pseudouridine synthase E (EC 4.2.1.70)  Signal peptidase I (EC 3.4.21.89)  Sulfate permease  Transcriptional regulator2C AraC family  Transcriptional regulator2C HxlR family  Transcriptional regulator2C LysR family  Transcriptional regulator2C MarR family  Transcriptional regulator2C TetR family  Vanillate O-demethylase oxygenase subunit (EC 1.14.13.82)  cAMP-binding proteins - catabolite gene activator and regulatory subunit of cAMP-dependent protein kinases  hypothetical protein  hypothetical protein  hypothetical protein  hypothetical protein  hypothetical protein  hypothetical protein  hypothetical protein  hypothetical protein  hypothetical protein  hypothetical protein  hypothetical protein  macromolecule metabolism; macromolecule synthesis2C modification; dna - replication2C repair2C restr./modif.  major facilitator superfamily MFS\_1  probable oxidoreductase/Short-chain dehydrogenase  transcriptional regulator PobR2C AraC family  transcriptional regulator2C GntR family  transcriptional regulator2C LysR family  two-component system sensor protein |
| 57839 | AGDB01000049.1:1..57839 | 12C4-alpha-glucan (glycogen) branching enzyme2C GH-13-type (EC 2.4.1.18)  ADP-ribose 1"-phosphate phophatase related protein  Alpha-12C2-mannosidase  Alpha-12C2-mannosidase  Alpha-amylase (EC 3.2.1.1)  Catalase (EC 1.11.1.6)  DNA-binding protein  Dihydrodipicolinate synthase (EC 4.2.1.52)  FIG01210888: hypothetical protein  FIG01211529: hypothetical protein  FIG01214235: hypothetical protein  HEN1 C-terminal domain; double-stranded RNA 3'-methylase  HipA protein  L-sorbosone dehydrogenase  Methionine aminopeptidase (EC 3.4.11.18)  Outer membrane protein romA  Pca regulon regulatory protein PcaR  Protein yciF  RND efflux system2C membrane fusion protein CmeA  RND multidrug efflux transporter; Acriflavin resistance protein  RpfN protein  Signal transduction histidine kinase  TonB-dependent receptor  Transcriptional regulator2C TetR family  Transporter2C MFS superfamily  Trehalose synthase (EC 5.4.99.16)  Two-component response regulator  chemotaxis protein  hypothetical protein  hypothetical protein  hypothetical protein  hypothetical protein  hypothetical protein  hypothetical protein  hypothetical protein  hypothetical protein  hypothetical protein  putative cytochrome b561  putative phosphatase  tRNA-Met-CAT  transcriptional regulator |
| 55650 | AGDB01000021.1:1..55650 | 3-dehydroshikimate dehydratase  5'-nucleotidase/2'2C3'-cyclic phosphodiesterase and related esterases  ATP-dependent helicase DinG/Rad3  Acid phosphatase  Catalase (EC 1.11.1.6)  Cellulase  Dihydroxy-acid dehydratase (EC 4.2.1.9)  Endonuclease  FIG01111041: hypothetical protein  FIG01111120: hypothetical protein  FIG01112224: hypothetical protein  FIG01211474: hypothetical protein  FIG01211497: hypothetical protein  FOG: Ankyrin repeat  Glyoxalase/Bleomycin resistance protein/Dioxygenase family protein  MoxR-like ATPases  POSSIBLE CONSERVED MEMBRANE PROTEIN  Permease  Permeases of the major facilitator superfamily  Putative inner membrane protein  SAM-dependent methyltransferases  Shikimate 5-dehydrogenase I alpha (EC 1.1.1.25)  Similarity with glutathionylspermidine synthase (EC 6.3.1.8)2C group 1  Transcriptional regulator2C AsnC family  Uncharacterized iron-regulated membrane protein; Iron-uptake factor PiuB  Uncharacterized membrane protein  carbonic anhydrase2C family 3  hypothetical protein  hypothetical protein  hypothetical protein  hypothetical protein  hypothetical protein  hypothetical protein  hypothetical protein  hypothetical protein  hypothetical protein  hypothetical protein  hypothetical protein  hypothetical protein  hypothetical protein  hypothetical protein  hypothetical protein  hypothetical protein  oxidoreductase  phosphoesterase2C putative  porin2C putative |
| 52313 | AGDB01000010.1:1..52313 | Alpha-glucuronidase (EC 3.2.1.139)  Beta-xylosidase (EC 3.2.1.37)  D-alanyl-D-alanine dipeptidase  FIG01209832: hypothetical protein  FIG01210443: hypothetical protein  FIG01210473: hypothetical protein  FIG01210986: hypothetical protein  FIG01211119: hypothetical protein  FIG01211151: hypothetical protein  FIG01211332: hypothetical protein  FIG01211609: hypothetical protein  FIG01217284: hypothetical protein  Glucan 12C4-beta-glucosidase  L-alanine-DL-glutamate epimerase  N-acetylmuramoyl-L-alanine amidase (EC 3.5.1.28)  Pass1-related protein  Peptidase B (EC 3.4.11.23)  Peptide transport system permease protein sapC (TC 3.A.1.5.5)  Periplasmic beta-glucosidase (EC 3.2.1.21)  Putative cytoplasmic protein  TonB-dependent receptor  TonB-dependent receptor  Tryptophan halogenase  Xylose isomerase (EC 5.3.1.5)  Xylosidase/arabinosidase  Xylulose kinase (EC 2.7.1.17)  hydrolase  hydrolase2C haloacid delahogenase-like family  hypothetical protein  hypothetical protein  hypothetical protein  hypothetical protein  hypothetical protein  probable exported protein STY2149  sal operon transcriptional repressor  sal operon transcriptional repressor  sialic acid-specific 9-O-acetylesterase  transport protein |
| 48575 | AGDB01000011.1:1..48575 | ABC transporter ATP-binding protein USSDB6B  ABC-type transport system involved in resistance to organic solvents2C periplasmic component USSDB6C  ABC-type transport system involved in resistance to organic solvents2C permease component USSDB6A  ATP binding component of ABC-transporter  Acetyl-CoA:acetoacetyl-CoA transferase2C beta subunit (EC 2.8.3.8)  Alkylated DNA repair protein AlkB  CDP-diacylglycerol--serine O-phosphatidyltransferase (EC 2.7.8.8)  Cystathionine beta-synthase (EC 4.2.1.22)  Cystathionine gamma-lyase (EC 4.4.1.1)  DNA-binding protein H-NS  Electron transfer flavoprotein-ubiquinone oxidoreductase (EC 1.5.5.1)  Electron transfer flavoprotein2C alpha subunit  Electron transfer flavoprotein2C beta subunit  FIG01212400: hypothetical protein  FIG01213248: hypothetical protein  GDP-mannose 42C6 dehydratase (EC 4.2.1.47)  Glucose-1-phosphate thymidylyltransferase (EC 2.7.7.24)  Glycosyltransferase  Mannose-6-phosphate isomerase (EC 5.3.1.8) / Mannose-1-phosphate guanylyltransferase (GDP) (EC 2.7.7.22)  Membrane lipoprotein lipid attachment site containing protein USSDB6D  Methyltransferase type 11  O-antigen export system permease protein RfbD  Outer membrane protein  Phosphomannomutase (EC 5.4.2.8)  Phytoene desaturase (EC 1.14.99.-)  Prolyl-tRNA synthetase (EC 6.1.1.15)2C bacterial type  Putative transmembrane oxidoreductase protein  Succinyl-CoA:3-ketoacid-coenzyme A transferase subunit A (EC 2.8.3.5)  Type II secretion system protein-like protein  UDP-glucose 4-epimerase (EC 5.1.3.2)  UptF protein  dTDP-4-dehydrorhamnose 32C5-epimerase (EC 5.1.3.13)  dTDP-4-dehydrorhamnose reductase (EC 1.1.1.133)  dTDP-glucose 42C6-dehydratase (EC 4.2.1.46)  glycosyltransferase WbpX  hypothetical protein  hypothetical protein  hypothetical protein  hypothetical protein  integral membrane protein  integral membrane protein  outer membrane protein  oxidoreductase  oxidoreductase2C short-chain dehydrogenase/reductase family |
| 45384 | AGDB01000200.1:1..45384 | ABC transporter2C permease protein2C putative  Alpha-ketoglutarate-dependent taurine dioxygenase (EC 1.14.11.17)  Arylesterase precursor (EC 3.1.1.2)  Cell division transporter2C ATP-binding protein FtsE (TC 3.A.5.1.1)  Cobalt-zinc-cadmium resistance protein CzcA; Cation efflux system protein CusA  Diacylglycerol kinase (EC 2.7.1.107)  FIG01209891: hypothetical protein  FIG01210409: hypothetical protein  FIG01211353: hypothetical protein  Hemin transport protein  Hemin uptake protein  Integral membrane protein TerC  LemA family protein  Nucleoside permease NupC  Probable Co/Zn/Cd efflux system membrane fusion protein  Putative hemolysin  Response regulators consisting of a CheY-like receiver domain and a winged-helix DNA-binding domain  Ribokinase (EC 2.7.1.15)  S-adenosylmethionine synthetase (EC 2.5.1.6)  Sensor protein basS/pmrB (EC 2.7.3.-)  Ser/Thr protein phosphatase family protein2C UDP-22C3-diacylglucosamine hydrolase (EC 3.6.1.-) homolog  Taurine transport ATP-binding protein TauB  Taurine transport system permease protein TauC  Taurine-binding periplasmic protein TauA  TonB-dependent hemin 2C ferrichrome receptor  TonB-dependent receptor  Two component Transcriptional regulator2C Winged helix family  Voltage-gated potassium channel beta subunit  conserved hypothetical protein  conserved hypothetical protein  hypothetical protein  hypothetical protein  methyltransferase  phosphoanhydride phosphohydrolase  putative membrane protein  tRNA dihydrouridine synthase B (EC 1.-.-.-)  two-component system sensor protein |
| 45173 | AGDB01000328.1:1..45173 | 3-hydroxyacyl-CoA dehydrogenase type II  ABC transporter permease protein  ABC-type nitrate/sulfonate/bicarbonate transport system2C ATPase component  Aconitate hydratase (EC 4.2.1.3) @ 2-methylisocitrate dehydratase (EC 4.2.1.99)  Aconitate hydratase 2 (EC 4.2.1.3)  Carbamoyl-phosphate synthase large chain (EC 6.3.5.5)  Carbamoyl-phosphate synthase small chain (EC 6.3.5.5)  Chemotaxis response regulator protein-glutamate methylesterase CheB (EC 3.1.1.61)  Dihydrodipicolinate reductase (EC 1.3.1.26)  FIG00786362: hypothetical protein  FIG01209712: hypothetical protein  FIG01210619: hypothetical protein  FIG01211894: hypothetical protein  Ferrous iron transport protein B  Hydroxymethylglutaryl-CoA lyase (EC 4.1.3.4)  Hydroxymethylpyrimidine ABC transporter2C substrate-binding component  Lactoylglutathione lyase and related lyases  Long-chain-fatty-acid--CoA ligase (EC 6.2.1.3)  LysR family transcriptional regulator YbhD  Lysyl-tRNA synthetase (class II) (EC 6.1.1.6)  Methylglutaconyl-CoA hydratase (EC 4.2.1.18)  Peptide chain release factor 2; programmed frameshift-containing  Phosphate-specific outer membrane porin OprP ; Pyrophosphate-specific outer membrane porin OprO  Regulatory protein2C RpfE type  RpfF protein  Sensory/regulatory protein rpfC (EC 2.7.3.-)  Single-stranded-DNA-specific exonuclease RecJ (EC 3.1.-.-)  Transcription elongation factor GreA  Translation elongation factor P-related protein  Uncharacterized protein conserved in bacteria  Valine--pyruvate aminotransferase (EC 2.6.1.66)  diguanylate cyclase/phosphodiesterase (GGDEF  ferrous iron transport protein  hypothetical protein  hypothetical protein  hypothetical protein  hypothetical protein  regulatory protein  response regulator |
| 44358 | AGDB01000314.1:1..44358 | ADP-ribosylglycohydrolase family protein  Alpha-L-fucosidase (EC 3.2.1.51)  Chemotaxis protein cheA (EC 2.7.3.-)  Chemotaxis response regulator containing a CheY-like receiver domain and a methylesterase domain  Cytoplasmic copper homeostasis protein cutC  DNA-directed RNA polymerase specialized sigma subunit2C sigma24-like  DinG family ATP-dependent helicase YoaA  FIG01112193: hypothetical protein  FIG01210372: hypothetical protein  FIG01210820: hypothetical protein  FIG01211536: hypothetical protein  FIG01212489: hypothetical protein  Glutathione synthetase (EC 6.3.2.3)  Glycosyltransferase  Inactive homolog of metal-dependent proteases2C putative molecular chaperone  L-asparaginase (EC 3.5.1.1)  Multimodular transpeptidase-transglycosylase (EC 2.4.1.129) (EC 3.4.-.-)  Thioredoxin reductase  Thioredoxin reductase  TonB protein  TonB-dependent receptor  conserved hypothetical protein  hypothetical protein  hypothetical protein  hypothetical protein  hypothetical protein  hypothetical protein  hypothetical protein  hypothetical protein  hypothetical protein  pilus biogenesis protein  probable RebB like protein  twitching motility protein PilG  twitching motility protein PilH  type IV pili signal transduction protein PilI |
| 42547 | AGDB01000426.1:1..42547 | 2-hydroxy-6-oxo-6-phenylhexa-22C4-dienoate hydrolase (EC 3.7.1.-)  ABC transporter2C ATP-binding protein  Amidases related to nicotinamidase  AsmA family membrane protein  Aspartokinase (EC 2.7.2.4) / Homoserine dehydrogenase (EC 1.1.1.3)  COG1272: Predicted membrane protein hemolysin III homolog  Chitinase (EC 3.2.1.14)  Cystathionine gamma-synthase (EC 2.5.1.48)  FIG01210154: hypothetical protein  FIG01211483: hypothetical protein  Glycosyltransferase  Histidine kinase/response regulator hybrid protein  Histidine kinase/response regulator hybrid protein  Homoserine O-acetyltransferase (EC 2.3.1.31)  Inosine-5'-monophosphate dehydrogenase (EC 1.1.1.205)  L-serine dehydratase (EC 4.3.1.17)  Mannose-6-phosphate isomerase  Peptide chain release factor 3  Permeases of the major facilitator superfamily  Probable signal peptide protein  RND multidrug efflux transporter; Acriflavin resistance protein  Transcriptional regulator2C AraC family  Transcriptional regulator2C GntR family  Uroporphyrinogen III decarboxylase (EC 4.1.1.37)  cytochrome B561  efflux transporter2C RND family2C MFP subunit  hypothetical protein  hypothetical protein  hypothetical protein  hypothetical protein  response regulator receiver domain protein (CheY-like) |
| 42378 | AGDB01000106.1:1..42378 | 2-octaprenyl-3-methyl-6-methoxy-12C4-benzoquinol hydroxylase (EC 1.14.13.-)  Adenosine (5')-pentaphospho-(5'')-adenosine pyrophosphohydrolase (EC 3.6.1.-)  Anthranilate phosphoribosyltransferase (EC 2.4.2.18)  Anthranilate synthase2C amidotransferase component (EC 4.1.3.27) @ Para-aminobenzoate synthase2C amidotransferase component (EC 2.6.1.85)  Anthranilate synthase2C aminase component (EC 4.1.3.27)  Bacterioferritin  Cyclic AMP receptor protein  FIG01209882: hypothetical protein  FIG01210288: hypothetical protein  FIG01211412: hypothetical protein  FIG01211564: hypothetical protein  FIG01213032: hypothetical protein  FIG034602: Probable transmembrane protein  FIG035830: Two-component system regulatory protein  FIG037127: Two-component system sensor protein  FIG071884: Hypothetical protein  FMN-dependent NADH-azoreductase  GCN5-related N-acetyltransferase  Indole-3-glycerol phosphate synthase (EC 4.1.1.48)  Integral membrane protein CcmA involved in cell shape determination  LSU ribosomal protein L13p (L13Ae)  Low-specificity L-threonine aldolase (EC 4.1.2.5)  Lytic enzyme  Na( ) H( ) antiporter subunit A; Na( ) H( ) antiporter subunit B  Na( ) H( ) antiporter subunit C  Nitrilotriacetate monooxygenase component B (EC 1.14.13.-)  Phosphoribosylaminoimidazole-succinocarboxamide synthase (EC 6.3.2.6)  Phosphoserine phosphatase  Probable signal peptide protein  Quaternary ammonium compound-resistance protein sugE  Ribulose-phosphate 3-epimerase (EC 5.1.3.1)  S-adenosylmethionine decarboxylase proenzyme (EC 4.1.1.50)2C prokaryotic class 1A  SSU ribosomal protein S9p (S16e)  Sulfate transporter2C CysZ-type  Transcription regulator [contains diacylglycerol kinase catalytic domain]  bacterioferritin-associated ferredoxin  hypothetical protein  hypothetical protein  hypothetical protein  hypothetical protein  hypothetical protein  hypothetical protein  hypothetical protein  hypothetical protein  probable cellulase  tRNA-Gln-CTG  tRNA-Met-CAT  transcriptional regulator |
| 42272 | AGDB01000313.1:1..42272 | 6-phospho-beta-glucosidase  Alcohol dehydrogenase (EC 1.1.1.1)  Alpha-12C2-mannosidase  Alpha-L-fucosidase (EC 3.2.1.51)  Beta-galactosidase (EC 3.2.1.23)  Beta-galactosidase (EC 3.2.1.23)  Beta-hexosaminidase (EC 3.2.1.52)  Beta-mannosidase (EC 3.2.1.25)  FIG01210377: hypothetical protein  FIG01210595: hypothetical protein  FIG01213069: hypothetical protein  Glucan 12C4-beta-glucosidase  IS1595 transposase  N-acetylglucosamine-regulated TonB-dependent outer membrane receptor  Ribokinase (EC 2.7.1.15)  TonB-dependent receptor  Transcriptional regulator2C HxlR family  hypothetical protein  hypothetical protein  hypothetical protein  hypothetical protein  predicted N-acetylglucosamine kinase2C glucokinase-like (EC 2.7.1.59)  putative transcriptional regulator |
| 41785 | AGDB01000027.1:1..41785 | 2-keto-3-deoxy-D-arabino-heptulosonate-7-phosphate synthase II (EC 2.5.1.54)  22C4-dienoyl-CoA reductase [NADPH] (EC 1.3.1.34)  DNA-directed RNA polymerase alpha subunit (EC 2.7.7.6)  Dienelactone hydrolase and related enzyme  FIG01210215: hypothetical protein  FIG01211222: hypothetical protein  FIG01211949: hypothetical protein  FIG01213271: hypothetical protein  FIG01213947: hypothetical protein  GTP-binding protein TypA/BipA  Glutathione S-transferase (EC 2.5.1.18)  Glutathione S-transferase (EC 2.5.1.18)  Glycerophosphoryl diester phosphodiesterase (EC 3.1.4.46)  LSU ribosomal protein L15p (L27Ae)  LSU ribosomal protein L17p  LSU ribosomal protein L18p (L5e)  LSU ribosomal protein L30p (L7e)  LSU ribosomal protein L6p (L9e)  LysR family transcriptional regulator STM2281  Malate dehydrogenase (EC 1.1.1.37)  Membrane protein  Peptidyl-prolyl cis-trans isomerase (EC 5.2.1.8)  Periplasmic thiol:disulfide oxidoreductase DsbB2C required for DsbA reoxidation  Preprotein translocase secY subunit (TC 3.A.5.1.1)  Propionate--CoA ligase (EC 6.2.1.17)  SSU ribosomal protein S11p (S14e)  SSU ribosomal protein S13p (S18e)  SSU ribosomal protein S4p (S9e)  SSU ribosomal protein S5p (S2e)  SSU ribosomal protein S8p (S15Ae)  TonB-dependent receptor  Zinc-regulated outer membrane receptor  arabinofuranosidase  hypothetical protein  hypothetical protein  hypothetical protein  hypothetical protein  hypothetical protein |
| 41280 | AGDB01000004.1:1..41280 | 3-beta hydroxysteroid dehydrogenase/isomerase family protein in hypothetical gene cluster  3-ketoacyl-CoA thiolase (EC 2.3.1.16) @ Acetyl-CoA acetyltransferase (EC 2.3.1.9)  3-oxoacyl-[acyl-carrier-protein] synthase2C KASIII in hypothetical gene cluster  Acyl-CoA synthetase (AMP-forming)/AMP-acid ligase/Peptide synthase  Acyltransferase  Distant homolog of E. coli HemX protein in Xanthomonadaceae  FIG01209870: hypothetical protein  FIG01209938: hypothetical protein  FIG01210164: hypothetical protein  FIG01210356: hypothetical protein  FIG01210420: hypothetical protein  FIG01210478: hypothetical protein  FIG01211750: hypothetical protein  FIG01211965: hypothetical protein  FIG01212698: hypothetical protein  FIG136845: Rhodanese-related sulfurtransferase  Ferredoxin  Glycerol-3-phosphate dehydrogenase [NAD(P) ] (EC 1.1.1.94)  Histone acetyltransferase HPA2 and related acetyltransferases  Homolog of E. coli HemY protein  Hydrolase2C alpha/beta fold family protein2C in hypothetical gene cluster  Hypothetical protein YaeJ with similarity to translation release factor  MloA  Nitrogen regulation protein NR(I)  O-methyltransferase  Protein YigP (COG3165) clustered with ubiquinone biosynthetic genes  Protein export cytoplasm chaperone protein (SecB2C maintains protein to be exported in unfolded state)  Superoxide dismutase [Cu-Zn] precursor (EC 1.15.1.1)  Superoxide dismutase [Cu-Zn] precursor (EC 1.15.1.1)  Two-component system sensor protein  Ubiquinone biosynthesis monooxygenase UbiB  Uncharacterized domain COG3236 / GTP cyclohydrolase II (EC 3.5.4.25)  Uroporphyrinogen-III synthase (EC 4.2.1.75)  Zinc protease  hypothetical protein  hypothetical protein  prolyl oligopeptidase family protein  putative 6-aminohexanoate-dimer hydrolase  tRNA (cytosine34-2'-O-)-methyltransferase (EC 2.1.1.-)  tRNA pseudouridine synthase C (EC 4.2.1.70)  tetR-family transcriptional regulatory protein |
| 40588 | AGDB01000439.1:1..40588 | 4-hydroxybenzoyl-CoA thioesterase family active site  ATP dependent RNA helicase  ATP-dependent DNA helicase RecQ  Aspartyl-tRNA synthetase (EC 6.1.1.12)  Chloride channel protein  Cob(I)alamin adenosyltransferase (EC 2.5.1.17)  Coenzyme PQQ synthesis protein B  Coenzyme PQQ synthesis protein C  Coenzyme PQQ synthesis protein D  Coenzyme PQQ synthesis protein E  Crossover junction endodeoxyribonuclease RuvC (EC 3.1.22.4)  FIG000859: hypothetical protein  FIG01210307: hypothetical protein  GTP pyrophosphokinase (EC 2.7.6.5)  Holliday junction DNA helicase RuvA  Holliday junction DNA helicase RuvB  Kup system potassium uptake protein  MotA/TolQ/ExbB proton channel family protein  Non-specific DNA-binding protein Dps / Iron-binding ferritin-like antioxidant protein / Ferroxidase (EC 1.16.3.1)  Outer membrane lipoprotein omp16 precursor  Queuosine Biosynthesis QueC ATPase  Queuosine Biosynthesis QueE Radical SAM  TPR repeat containing exported protein; Putative periplasmic protein contains a protein prenylyltransferase domain  Tol biopolymer transport system2C TolR protein  TolA protein  TonB-dependent receptor  Transcriptional regulator lysR family  Transporter2C LysE family  acetyltransferase  conserved hypothetical protein  hypothetical protein  hypothetical protein  hypothetical protein  hypothetical protein  hypothetical protein  tRNA-Lys-TTT  tolB protein precursor2C periplasmic protein involved in the tonb-independent uptake of group A colicins  transcriptional regulator uid family |
| 40053 | AGDB01000267.1:1..40053 | 1-deoxy-D-xylulose 5-phosphate synthase (EC 2.2.1.7)  Acyl-CoA dehydrogenase (EC 1.3.99.3)  Cyclomaltodextrin glucanotransferase  GumB protein  GumN protein  HNH endonuclease family protein  Integration host factor alpha subunit  LSU ribosomal protein L20p  LSU ribosomal protein L35p  Nitrate/nitrite response regulator protein  Phenylalanyl-tRNA synthetase alpha chain (EC 6.1.1.20)  Phenylalanyl-tRNA synthetase beta chain (EC 6.1.1.20)  Predicted maltose transporter MalT  Sensory box histidine kinase/response regulator  Threonyl-tRNA synthetase (EC 6.1.1.3)  Transcriptional regulator of maltose utilization2C LacI family  Transcriptional regulator2C MerR family  Translation initiation factor 3  Xanthan biosynthesis acetyltransferase GumF  Xanthan biosynthesis chain length determinant protein GumC  Xanthan biosynthesis exopolysaccharide polymerase GumE  Xanthan biosynthesis glucuronosyltransferase GumK  Xanthan biosynthesis glycosyltransferase GumD  Xanthan biosynthesis glycosyltransferase GumH  Xanthan biosynthesis glycosyltransferase GumI  Xanthan biosynthesis glycosyltransferase GumM  Xanthan biosynthesis oligosaccharidyl-lipid flippase GumJ  Xanthan biosynthesis pyruvyltransferase GumL  adenylyl cyclase class-3/4/guanylyl cyclase  hypothetical protein  hypothetical protein  hypothetical protein  hypothetical protein  hypothetical protein  hypothetical protein  serine/threonine protein kinase  tRNA-Pro-GGG |
| 38384 | AGDB01000237.1:1..38384 | 3-hydroxyanthranilate 32C4-dioxygenase (EC 1.13.11.6)  5'-nucleotidase (EC 3.1.3.5)  Asparaginyl-tRNA synthetase (EC 6.1.1.22)  Carbonic anhydrase (EC 4.2.1.1)  Cell division protein ZipA  Chromosome partition protein smc  Exodeoxyribonuclease I (EC 3.1.11.1)  FIG01209847: hypothetical protein  FIG01210780: hypothetical protein  FIG01211446: hypothetical protein  FIG01212086: hypothetical protein  FIG01212272: hypothetical protein  FIG01212390: hypothetical protein  FIG01212834: hypothetical protein  Iron binding protein SufA for iron-sulfur cluster assembly  Kynureninase (EC 3.7.1.3)  Kynurenine 3-monooxygenase (EC 1.14.13.9)  LSU ribosomal protein L9p  NAD kinase (EC 2.7.1.23)  NAD-specific glutamate dehydrogenase (EC 1.4.1.2)2C large form  Periplasmic aromatic amino acid aminotransferase beta precursor (EC 2.6.1.57)  SECRETION ACTIVATOR PROTEIN  SSU ribosomal protein S18p  SSU ribosomal protein S6p  Spermidine synthase-like protein  Transcriptional regulator2C TetR (AcrR) family [USSDB4A]  hypothetical protein  hypothetical protein  hypothetical protein  hypothetical protein  hypothetical protein  hypothetical protein  hypothetical protein  hypothetical protein  hypothetical protein  hypothetical protein  hypothetical protein  putative secreted protein  transcriptional regulator  transcriptional regulator protein Pai2 |
| 37173 | AGDB01000067.1:1..37173 | 5-methyltetrahydropteroyltriglutamate--homocysteine methyltransferase (EC 2.1.1.14)  Conditioned medium factor  FIG01209725: hypothetical protein  FIG01210483: hypothetical protein  FIG01211006: hypothetical protein  FIG01211818: hypothetical protein  FMN oxidoreductase  Formyltetrahydrofolate deformylase (EC 3.5.1.10)  Inner membrane protein  LysR family transcriptional regulator STM3121  NAD-dependent protein deacetylase of SIR2 family  Outer membrane protein  Permeases of the major facilitator superfamily  Protease  RND efflux system2C inner membrane transporter CmeB  RND efflux system2C membrane fusion protein CmeA  RND efflux system2C outer membrane lipoprotein CmeC  Response regulator receiver:ATP-binding region2C ATPase-like:Histidine kinase A-like:Histidine kinase  Response regulator2C ompR family [USSDB2A]  Sensor protein PhoQ (EC 2.7.13.3)  Sensory histidine kinase BaeS  Two-component system regulatory protein  biphenyl-22C3-diol 12C2-dioxygenase III-related protein  dehydrogenase  hypothetical protein  probable two-component response regulator  tRNA dihydrouridine synthase A |
| 36361 | AGDB01000355.1:1..36361 | Acetylglutamate kinase (EC 2.7.2.8)  Acetylornithine deacetylase (EC 3.5.1.16)  Argininosuccinate lyase (EC 4.3.2.1)  Argininosuccinate synthase (EC 6.3.4.5)  B. burgdorferi predicted coding region BB0646  C4-type zinc finger protein2C DksA/TraR family  Cysteinyl-tRNA synthetase (EC 6.1.1.16)  Dihydroorotase (EC 3.5.2.3)  Dipeptidyl aminopeptidases/acylaminoacyl-peptidases-like protein  Drug:proton antiporter  FIG01112450: hypothetical protein  FIG01209975: hypothetical protein  GGDEF family protein  Gamma-glutamyl phosphate reductase (EC 1.2.1.41)  Glutamate 5-kinase (EC 2.7.2.11)  Histone acetyltransferase HPA2 and related acetyltransferases  N-acetyl-gamma-glutamyl-phosphate reductase (EC 1.2.1.38)  N-acetylglutamate synthase (EC 2.3.1.1)  N-acetylornithine carbamoyltransferase (EC 2.1.3.9)  Protein YidD  Sulfur acceptor protein SufE for iron-sulfur cluster assembly  Unnamed hypothetical gene product in the file!!  hypothetical protein  hypothetical protein  hypothetical protein  hypothetical protein  hypothetical protein  hypothetical protein  pectinesterase  peptidase  predicted cell surface protein/ lipoprotein  putative immediate early protein  putative secreted protein  secretion protein2C putative |
| 35715 | AGDB01000273.1:1..35715 | 3-ketoacyl-CoA thiolase (EC 2.3.1.16) @ Acetyl-CoA acetyltransferase (EC 2.3.1.9)  Agmatine deiminase (EC 3.5.3.12)  Cytidylate kinase (EC 2.7.4.14)  Enoyl-CoA hydratase (EC 4.2.1.17) / 32C2-trans-enoyl-CoA isomerase (EC 5.3.3.8) / 3-hydroxyacyl-CoA dehydrogenase (EC 1.1.1.35)  FIG01210851: hypothetical protein  FIG016519: Putative DNA-binding protein  GTP-binding protein EngA  Heat shock (predicted periplasmic) protein YciM2C precursor  Integration host factor beta subunit  LSU ribosomal protein L36p  Mlr7403 protein  Molybdopterin biosynthesis protein MoeA  N-carbamoylputrescine amidase (3.5.1.53)  Nucleoside diphosphate kinase (EC 2.7.4.6)  Outer membrane protein YfgL2C lipoprotein component of the protein assembly complex (forms a complex with YaeT2C YfiO2C and NlpB)  Pheromone shutdown protein  Predicted transcriptional regulator for fatty acid degradation FadQ2C TetR family  Probable Co/Zn/Cd efflux system membrane fusion protein  Ribosomal RNA large subunit methyltransferase N (EC 2.1.1.-)  SSU ribosomal protein S1p  Sulfur carrier protein adenylyltransferase ThiF  Transport protein  Transport protein  Type IV pilus biogenesis protein PilF  UDP-N-acetylglucosamine 42C6-dehydratase (EC 4.2.1.-)  UTP--glucose-1-phosphate uridylyltransferase (EC 2.7.7.9)  hypothetical protein  lipopolysaccharide core biosynthesis protein  molybdopterin-guanine dinucleotide biosynthesis protein A |
| 35092 | AGDB01000125.1:1..35092 | Acriflavin resistance protein  Acriflavin resistance protein  FIG01111128: hypothetical protein  Galactosamine-6-phosphate isomerase (EC 5.3.1.-)  INTEGRAL MEMBRANE PROTEIN (Rhomboid family)  Isocitrate dehydrogenase [NADP] (EC 1.1.1.42); Monomeric isocitrate dehydrogenase [NADP] (EC 1.1.1.42)  Isocitrate dehydrogenase phosphatase (EC 2.7.11.5)/kinase (EC 3.1.3.-)  N-acetyl-L2CL-diaminopimelate deacetylase (EC 3.5.1.47)  N-acetylgalactosamine kinase2C ROK-type (EC 2.7.1.157)  N-acetylglucosamine-6-phosphate deacetylase (EC 3.5.1.25)  NADPH dependent preQ0 reductase  Predicted exported glycosyl hydrolase family 31 protein  Predicted secreted alpha-N-acetylgalactosaminidase (EC 3.2.1.49)  Probable Co/Zn/Cd efflux system membrane fusion protein  Serine/threonine protein kinase  Tagatose-6-phosphate kinase AgaZ (EC 2.7.1.144)  TonB-dependent receptor  Transcription termination factor Rho  Transcriptional repressor of aga operon  hypothetical protein  hypothetical protein  hypothetical protein  putative secreted protein |
| 33356 | AGDB01000031.1:1..33356 | ATPase  Aspartate-semialdehyde dehydrogenase (EC 1.2.1.11)  Beta-carotene ketolase (EC 1.14.-.-)  Chorismate synthase (EC 4.2.3.5)  Cytochrome oxidase biogenesis protein Sco1/SenC/PrrC2C putative copper metallochaperone  Deoxycytidine triphosphate deaminase (EC 3.5.4.13)  Diadenosine tetraphosphate (Ap4A) hydrolase and other HIT family hydrolases  Dienelactone hydrolase family  FIG01209679: hypothetical protein  GDP-mannose pyrophosphatase YffH  Glyoxylate reductase (EC 1.1.1.79) / Glyoxylate reductase (EC 1.1.1.26) / Hydroxypyruvate reductase (EC 1.1.1.81); 2-ketoaldonate reductase2C broad specificity (EC 1.1.1.215) (EC 1.1.1.-)  Phosphatidylserine decarboxylase (EC 4.1.1.65)  Protein-N(5)-glutamine methyltransferase PrmB2C methylates LSU ribosomal protein L3p  Ribosomal protein S12p Asp88 (E. coli) methylthiotransferase  Scaffold protein for [4Fe-4S] cluster assembly ApbC2C MRP-like  TonB-dependent receptor  TonB-dependent receptor  Transcription elongation factor GreB  hypothetical protein  hypothetical protein  hypothetical protein  hypothetical protein  hypothetical protein  integral membrane protein  membrane-bound lytic murein transglycosylase D precursor  prolyl oligopeptidase family protein |
| 33176 | AGDB01000257.1:1..33176 | 4'-phosphopantetheinyl transferase (EC 2.7.8.-)  5'-nucleotidase YjjG (EC 3.1.3.5)  Acetate permease ActP (cation/acetate symporter)  Acetyl-coenzyme A synthetase (EC 6.2.1.1)  Aldehyde dehydrogenase (EC 1.2.1.3)  Alkaline phosphatase D  Chromosome (plasmid) partitioning protein ParA / Sporulation initiation inhibitor protein Soj  Chromosome (plasmid) partitioning protein ParB / Stage 0 sporulation protein J  Dolichol-phosphate mannosyltransferase  Exodeoxyribonuclease III (EC 3.1.11.2)  FIG01111534: hypothetical protein  FIG01209701: hypothetical protein  FIG01210064: hypothetical protein  FIG01210656: hypothetical protein  FIG01211021: hypothetical protein  Gfa-like protein  Integral membrane protein  Mitomycin resistance protein  Putative membrane protein2C clustering with ActP  Sensor histidine kinase  TonB-dependent receptor  Transcriptional regulator2C LuxR family  dTDP-glucose 42C6-dehydratase (EC 4.2.1.46)  hypothetical protein  peptidyl-prolyl cis-trans isomerase  putative TPR repeat protein  rRNA small subunit methyltransferase2C glucose inhibited division protein GidB  transglycosylase associated protein |
| 33057 | AGDB01000210.1:1..33057 | Alkylphosphonate utilization operon protein PhnA  B12 binding domain / kinase domain / Methylmalonyl-CoA mutase (EC 5.4.99.2)  COG1451: Predicted metal-dependent hydrolase  Cation transport protein  EAL domain protein  FIG01210445: hypothetical protein  FKBP-type peptidyl-prolyl cis-trans isomerase SlyD (EC 5.2.1.8)  Hypothetical Zinc-finger containing protein  Lysyl endopeptidase (EC 3.4.21.50)  MFS transporter  N-ethylmaleimide reductase (EC 1.-.-.-)  Putative heat shock protein YegD  Quino(hemo)protein alcohol dehydrogenase2C PQQ-dependent (EC 1.1.99.8)  Small-conductance mechanosensitive channel  TPR repeat  TonB-dependent receptor  general stress protein  hypothetical protein  hypothetical protein  hypothetical protein  hypothetical protein  hypothetical protein  hypothetical protein  hypothetical protein  hypothetical protein  hypothetical protein  lipolytic enzyme2C G-D-S-L  nucleoprotein/polynucleotide-associated enzyme  peptidase S92C prolyl oligopeptidase active site domain protein  tRNA pseudouridine synthase A (EC 4.2.1.70) |
| 32776 | AGDB01000276.1:1..32776 | 3'-to-5' oligoribonuclease (orn)  Bifunctional protein: zinc-containing alcohol dehydrogenase; quinone oxidoreductase ( NADPH:quinone reductase) (EC 1.1.1.-); Similar to arginate lyase  Chemotaxis protein methyltransferase CheR (EC 2.1.1.80)  FIG00452947: hypothetical protein  GCN5-related N-acetyltransferase  Glycine betaine-binding protein  Glyoxalase family protein  LysR family transcriptional regulator STM3121  Manganese transport protein MntH  Mn-dependent transcriptional regulator MntR  NADH dehydrogenase2C subunit 5  Phosphoenolpyruvate synthase (EC 2.7.9.2)  Potassium efflux system KefA protein / Small-conductance mechanosensitive channel  Rrf2 family transcriptional regulator2C group III  SAM-dependent methyltransferase BA1462 (UbiE paralog)  ThiJ/PfpI  Thioredoxin reductase (EC 1.8.1.9)  TonB-dependent siderophore receptor  Transcriptional regulator2C AraC family  Transcriptional regulator2C AraC family  Transcriptional regulator2C LysR family  Transcriptional regulator2C LysR family  conserved hypothetical protein  hypothetical protein  hypothetical protein  hypothetical protein  hypothetical protein  hypothetical protein  monooxygenase2C FAD-binding  sensor kinase  tRNA-specific adenosine-34 deaminase (EC 3.5.4.-)  transcriptional regulator2C MarR family |
| 32771 | AGDB01000081.1:1..32771 | 4-hydroxy-3-methylbut-2-enyl diphosphate reductase (EC 1.17.1.2)  Acyl-CoA thioesterase II (EC 3.1.2.-)  Arginine-tRNA-protein transferase (EC 2.3.2.8)  COG0536: GTP-binding protein Obg  Copper metallochaperone2C bacterial analog of Cox17 protein  Cytochrome O ubiquinol oxidase subunit I (EC 1.10.3.-)  Cytochrome O ubiquinol oxidase subunit II (EC 1.10.3.-)  Cytochrome O ubiquinol oxidase subunit III (EC 1.10.3.-)  Cytochrome O ubiquinol oxidase subunit IV (EC 1.10.3.-)  DNA repair protein RadA  DltE  Enoyl-CoA hydratase (EC 4.2.1.17)  Excinuclease ABC subunit A  FIG01209955: hypothetical protein  Isoleucyl-tRNA synthetase (EC 6.1.1.5)  LSU ribosomal protein L21p  LSU ribosomal protein L27p  Lipoprotein signal peptidase (EC 3.4.23.36)  PDZ domain family protein  Pathogenicity-related protein  Phosphoanhydride phosphohydrolase  Proposed peptidoglycan lipid II flippase MurJ  Riboflavin kinase (EC 2.7.1.26) / FMN adenylyltransferase (EC 2.7.7.2)  SSU ribosomal protein S20p  Transcriptional regulator2C MarR family  hypothetical protein  hypothetical protein  hypothetical protein  hypothetical protein  hypothetical protein  tRNA-Thr-CGT  thioesterase superfamily |
| 32281 | AGDB01000170.1:1..32281 | ADP-ribose pyrophosphatase (EC 3.6.1.13)  Alkyl hydroperoxide reductase protein C (EC 1.6.4.-)  Alkyl hydroperoxide reductase protein F (EC 1.6.4.-)  Aminopeptidase N  Cytosine deaminase (EC 3.5.4.1)  DNA polymerase I (EC 2.7.7.7)  FIG01111726: hypothetical protein  FIG01149443: hypothetical protein  Hydrogen peroxide-inducible genes activator  Methylase of polypeptide chain release factors  Nitroreductase  Peptide methionine sulfoxide reductase MsrA (EC 1.8.4.11)  Proline iminopeptidase (EC 3.4.11.5)  Regulator of nucleoside diphosphate kinase  ThiJ/PfpI family protein  Thymidine phosphorylase (EC 2.4.2.4)  Thymidine phosphorylase (EC 2.4.2.4)  Transaldolase (EC 2.2.1.2)  Transglutaminase-like domain  Ubiquinone/menaquinone biosynthesis methyltransferase UbiE (EC 2.1.1.-)  Uncharacterized protein conserved in bacteria  hydrolase2C haloacid dehalogenase-like family  hypothetical protein  hypothetical protein  hypothetical protein  hypothetical protein  hypothetical protein  hypothetical protein  hypothetical protein  putative lipase |
| 32070 | AGDB01000288.1:1..32070 | 2-deoxy-D-gluconate 3-dehydrogenase (EC 1.1.1.125)  2-keto-4-pentenoate hydratase (EC 4.2.1.-)  4-deoxy-L-threo-5-hexosulose-uronate ketol-isomerase (EC 5.3.1.17)  ABC-type phosphate transport system2C periplasmic component  Acid phosphatase( EC:3.1.3.2 )  DUF124 domain-containing protein  FIG01210586: hypothetical protein  Methyl-accepting chemotaxis protein I (serine chemoreceptor protein)  Pentapeptide repeat:Glyoxalase/bleomycin resistance protein/dioxygenase  Phenylalanine-4-hydroxylase (EC 1.14.16.1)  Probable lipoprotein  Probable lipoprotein  Putative OMR family iron-siderophore receptor precursor  TRAP-type C4-dicarboxylate transport system2C large permease component  TRAP-type C4-dicarboxylate transport system2C periplasmic component  TRAP-type transport system2C small permease component2C predicted N-acetylneuraminate transporter  TonB-dependent receptor  Transcriptional regulator2C AsnC family  UPF0028 protein YchK  Xylanase  carboxylesterase type B  hypothetical protein  hypothetical protein  hypothetical protein  hypothetical protein  hypothetical protein  hypothetical protein  lipoprotein2C putative  predicted 4-deoxy-L-threo-5-hexosulose-uronate ketol-isomerase (EC 5.3.1.17)  putative diguanylate cyclase (GGDEF) with PAS/PAC domain  putative iron-regulated membrane protein  transcriptional regulator2C LacI family |
| 31414 | AGDB01000404.1:1..31414 | Alanine dehydrogenase (EC 1.4.1.1)  Aldehyde dehydrogenase (EC 1.2.1.3)  FIG01209826: hypothetical protein  FIG01212049: hypothetical protein  Formate dehydrogenase chain D (EC 1.2.1.2)  Gamma-glutamyl-GABA hydrolase (EC 3.5.1.94)  MFS permease  Major facilitator superfamily (MFS\_1) transporter  Methylmalonate-semialdehyde dehydrogenase (EC 1.2.1.27)  Omega-amino acid--pyruvate aminotransferase (EC 2.6.1.18)  Oxidoreductase  Plasmid replication/partition related protein  Probable sensor/response regulator hybrid (EC 2.7.3.-)  Putative formate dehydrogenase oxidoreductase protein  Putrescine utilization regulator  Transcriptional regulator  glutamine synthetase family protein  glutamine synthetase family protein  hypothetical protein  hypothetical protein  hypothetical protein  hypothetical protein  hypothetical protein  hypothetical protein  probable membrane protein STY4873  response regulator receiver modulated diguanylate cyclase |
| 31353 | AGDB01000061.1:1..31353 | 1-acyl-sn-glycerol-3-phosphate acyltransferase (EC 2.3.1.51)  AAA ATPase2C central region  ATP-dependent RNA helicase  Adenylate cyclase (EC 4.6.1.1)  COG1272: Predicted membrane protein hemolysin III homolog  ComA operon protein 2  FIG01112904: hypothetical protein  FIG01210460: hypothetical protein  FIG01211085: hypothetical protein  FIG01211245: hypothetical protein  FIG01211637: hypothetical protein  FIG01212614: hypothetical protein  Indolepyruvate ferredoxin oxidoreductase2C alpha and beta subunits  MFS transporter  Maebl  Malate permease  Phosphohistidine phosphatase SixA  Phospholipid-binding protein  SAM-dependent methyltransferases  Staphylolytic protease preproenzyme LasA  cardiolipin synthase  coagulation factor 5/8 type domain protein  hypothetical protein  hypothetical protein  hypothetical protein  partition protein  putative secreted protein |
| 31346 | AGDB01000416.1:1..31346 | (3R)-hydroxymyristoyl-[acyl carrier protein] dehydratase (EC 4.2.1.-)  1-deoxy-D-xylulose 5-phosphate reductoisomerase (EC 1.1.1.267)  Acetyl-coenzyme A carboxyl transferase alpha chain (EC 6.4.1.2)  Acyl-[acyl-carrier-protein]--UDP-N-acetylglucosamine O-acyltransferase (EC 2.3.1.129)  DNA polymerase III alpha subunit (EC 2.7.7.7)  FIG01111683: hypothetical protein  FIG01211822: hypothetical protein  Hypothetical membrane protein2C possible involvement in cytochrome functioning/assembly  Intracellular PHB depolymerase (EC 3.1.1.-)  Lipid-A-disaccharide synthase (EC 2.4.1.182)  Membrane-associated zinc metalloprotease  Outer membrane protein assembly factor YaeT precursor  Permease of the drug/metabolite transporter (DMT) superfamily  Phosphatidate cytidylyltransferase (EC 2.7.7.41)  Ribonuclease HII (EC 3.1.26.4)  Ribosome recycling factor  Transcriptional regulator2C PadR family  Translation elongation factor Ts  UDP-3-O-[3-hydroxymyristoyl] glucosamine N-acyltransferase (EC 2.3.1.-)  Undecaprenyl pyrophosphate synthetase (EC 2.5.1.31)  Uridylate kinase (EC 2.7.4.-)  hypothetical protein  hypothetical protein  hypothetical protein  peptidyl-prolyl cis-trans isomerase  putative transcriptional regulator2C ArsR family  sensor protein  transcriptional regulator |
| 30723 | AGDB01000174.1:1..30723 | COG28792C Hypothetical small protein yjiX  Carbon starvation protein A  DUF819 domain-containing protein  Di-/tripeptide transporter  FIG01111510: hypothetical protein  FIG01112671: hypothetical protein  Glucose-methanol-choline (GMC) oxidoreductase:NAD binding site  Hydroxypyruvate isomerase (EC 5.3.1.22)  Inosose isomerase (EC 5.3.99.-)  Oligopeptide transporter  Putative nucleoside transporter yegT  Single-stranded DNA-binding protein  Transcriptional (co)regulator CytR  Xylose isomerase-like TIM barrel  accessory protein  alanyl dipeptidyl peptidase  hydrolase  hypothetical protein  hypothetical protein  oxidoreductase-like  phospholipase A1  prolyl oligopeptidase family protein  putative multi-domain protein |
| 30435 | AGDB01000014.1:1..30435 | Acyl-CoA dehydrogenase/oxidase domain protein  Catalase (EC 1.11.1.6)  Cell division topological specificity factor MinE  Dipeptidyl carboxypeptidase  FIG01211221: hypothetical protein  FIG01211837: hypothetical protein  FIG01211876: hypothetical protein  FIG01212144: hypothetical protein  FIG01212265: hypothetical protein  Glycine dehydrogenase [decarboxylating] (glycine cleavage system P protein) (EC 1.4.4.2)  Glycosyl transferase2C group 2 family protein  Inner membrane protein  LmbE-like protein  MFS transporter  Membrane-associated phospholipid phosphatase  Methyltransferase type 12  Ribosomal-protein-S5p-alanine acetyltransferase  Septum site-determining protein MinC  Septum site-determining protein MinD  TolA protein  Two-component system regulatory protein  Uncharacterized zinc-type alcohol dehydrogenase-like protein ybdR  VirB6 protein  hypothetical protein  hypothetical protein  hypothetical protein  hypothetical protein  hypothetical protein  hypothetical protein  two-component system sensor protein |
| 29946 | AGDB01000417.1:1..29946 | Acetylornithine aminotransferase (EC 2.6.1.11)  COG3178: Predicted phosphotransferase related to Ser/Thr protein kinases  Calcium-binding protein  Chromosomal replication initiator protein DnaA  Conserved hypothetical protein (perhaps related to histidine degradation)  DNA transport competence protein  DNA-binding response regulator  FIG006611: nucleotidyltransferase  FIG01111643: hypothetical protein  FIG01112835: hypothetical protein  FIG01209993: hypothetical protein  FIG01210990: hypothetical protein  FIG01213181: hypothetical protein  Ferrichrome-iron receptor  Iron-uptake factor PiuC  Phosphoribosylformylglycinamidine cyclo-ligase (EC 6.3.3.1)  Phosphoribosylglycinamide formyltransferase (EC 2.1.2.2)  Putative permease often clustered with de novo purine synthesis  S-adenosylmethionine:tRNA ribosyltransferase-isomerase (EC 5.-.-.-)  Thiamin biosynthesis lipoprotein ApbE  Transcriptional regulator2C AsnC family  hypothetical protein  hypothetical protein  hypothetical protein  hypothetical protein  hypothetical protein  hypothetical protein  lipoprotein2C putative  succinyl-diaminopimelate desuccinylase |
| 29620 | AGDB01000050.1:1..29620 | 12C2-dihydroxy-3-keto-5-methylthiopentene dioxygenase (EC 1.13.11.54)  22C3-diketo-5-methylthiopentyl-1-phosphate enolase-phosphatase (EC 3.1.3.77)  3-oxoacyl-[acyl-carrier protein] reductase (EC 1.1.1.100)  ATP phosphoribosyltransferase (EC 2.4.2.17)  FIG01211701: hypothetical protein  His repressor  Histidinol dehydrogenase (EC 1.1.1.23)  Histidinol-phosphatase (EC 3.1.3.15) / Imidazoleglycerol-phosphate dehydratase (EC 4.2.1.19)  Histidinol-phosphate aminotransferase (EC 2.6.1.9)  Histidyl-tRNA synthetase (EC 6.1.1.21)  Imidazole glycerol phosphate synthase amidotransferase subunit (EC 2.4.2.-)  Imidazole glycerol phosphate synthase cyclase subunit (EC 4.1.3.-)  MORN repeat family protein  Methylthioribulose-1-phosphate dehydratase (EC 4.2.1.109)  Phosphoribosyl-AMP cyclohydrolase (EC 3.5.4.19) / Phosphoribosyl-ATP pyrophosphatase (EC 3.6.1.31)  Phosphoribosylformimino-5-aminoimidazole carboxamide ribotide isomerase (EC 5.3.1.16)  Threonine synthase (EC 4.2.3.1)  conserved hypothetical protein  coproporphyrinogen III oxidase2C putative  hypothetical protein  hypothetical protein  hypothetical protein  hypothetical protein  hypothetical protein  hypothetical protein  hypothetical protein  peptide synthetase  permease  putative lipoprotein  sulfotransferase  transcriptional regulator2C Crp/Fnr family |
| 29197 | AGDB01000250.1:1..29197 | 4-Hydroxy-2-oxoglutarate aldolase (EC 4.1.3.16) / 2-dehydro-3-deoxyphosphogluconate aldolase (EC 4.1.2.14)  6-phosphogluconolactonase (EC 3.1.1.31)2C eukaryotic type  Cell division protein BolA  FIG01097475: hypothetical protein  Folate-dependent protein for Fe/S cluster synthesis/repair in oxidative stress  Glucokinase (EC 2.7.1.2)  Glucose-6-phosphate 1-dehydrogenase (EC 1.1.1.49)  Hypothetical protein USSDB1E  N-Acetyl-D-glucosamine ABC transport system2C permease protein 1  N-Acetyl-D-glucosamine ABC transport system2C permease protein 2  OmpA-related protein  Phosphogluconate dehydratase (EC 4.2.1.12)  Segregation and condensation protein A  Segregation and condensation protein B  Succinate dehydrogenase cytochrome b-556 subunit  Succinate dehydrogenase flavoprotein subunit (EC 1.3.99.1)  Succinate dehydrogenase hydrophobic membrane anchor protein  Succinate dehydrogenase iron-sulfur protein (EC 1.3.99.1)  Sugar ABC transporter2C periplasmic sugar-binding protein USSDB1B  Transcriptional regulator2C LacI family  YciL protein  sugar ABC transporter ATP-binding protein |
| 28935 | AGDB01000283.1:1..28935 | Acetyl-coenzyme A carboxyl transferase beta chain (EC 6.4.1.2)  COG0779: clustered with transcription termination protein NusA  Glycine cleavage system transcriptional activator  NADH ubiquinone oxidoreductase chain A (EC 1.6.5.3)  NADH-ubiquinone oxidoreductase chain B (EC 1.6.5.3)  NADH-ubiquinone oxidoreductase chain C (EC 1.6.5.3)  NADH-ubiquinone oxidoreductase chain D (EC 1.6.5.3)  NADH-ubiquinone oxidoreductase chain E (EC 1.6.5.3)  NADH-ubiquinone oxidoreductase chain F (EC 1.6.5.3)  NADH-ubiquinone oxidoreductase chain G (EC 1.6.5.3)  NADH-ubiquinone oxidoreductase chain H (EC 1.6.5.3)  NADH-ubiquinone oxidoreductase chain I (EC 1.6.5.3)  NADH-ubiquinone oxidoreductase chain J (EC 1.6.5.3)  NADH-ubiquinone oxidoreductase chain K (EC 1.6.5.3)  NADH-ubiquinone oxidoreductase chain L (EC 1.6.5.3)  NADH-ubiquinone oxidoreductase chain M (EC 1.6.5.3)  NADH-ubiquinone oxidoreductase chain N (EC 1.6.5.3)  Oxidoreductase  Phosphoglucosamine mutase (EC 5.4.2.10)  Phosphoribosylanthranilate isomerase (EC 5.3.1.24)  Preprotein translocase subunit SecG (TC 3.A.5.1.1)  Transcription termination protein NusA  Triosephosphate isomerase (EC 5.3.1.1)  Tryptophan synthase alpha chain (EC 4.2.1.20)  Tryptophan synthase beta chain (EC 4.2.1.20)  hypothetical protein  short chain dehydrogenase  tRNA pseudouridine synthase A (EC 4.2.1.70)  tRNA-Leu-GAG  tRNA-Met-CAT |
| 28903 | AGDB01000232.1:1..28903 | 3-oxoacyl-[acyl-carrier protein] reductase (EC 1.1.1.100)  3-oxoacyl-[acyl-carrier-protein] synthase2C KASII (EC 2.3.1.41)  3-oxoacyl-[acyl-carrier-protein] synthase2C KASIII (EC 2.3.1.41)  4-amino-4-deoxy-L-arabinose transferase and related glycosyltransferases of PMT family  Acyl carrier protein  COG1399 protein2C clustered with ribosomal protein L32p  Citrate synthase (si) (EC 2.3.3.1)  DNA polymerase III alpha subunit (EC 2.7.7.7)  DNA polymerase III delta prime subunit (EC 2.7.7.7)  DNA polymerase-like protein PA0670  Endonuclease  FIG004453: protein YceG like  FIG00454545: hypothetical protein  FIG01211730: hypothetical protein  FIG01214694: hypothetical protein  FIG022979: MoxR-like ATPases  FIG146278: Maf/YceF/YhdE family protein  LSU ribosomal protein L32p  Malonyl CoA-acyl carrier protein transacylase (EC 2.3.1.39)  Para-aminobenzoate synthase2C aminase component (EC 2.6.1.85)  Phage-related protein  RecA/RadA recombinase  SOS-response repressor and protease LexA (EC 3.4.21.88)  Thymidylate kinase (EC 2.7.4.9)  Transcriptional regulator2C ArsR family  Type IV pilus biogenesis protein PilZ  YefM protein (antitoxin to YoeB)  hypothetical protein  tRNA-Val-CAC |
| 28637 | AGDB01000058.1:1..28637 | Acetyl-CoA hydrolase  BatA (Bacteroides aerotolerance operon)  BatD  Beta-lactamase  FIG01210644: hypothetical protein  FIG01210654: hypothetical protein  FIG01210744: hypothetical protein  FIG01211734: hypothetical protein  Ferric siderophore transport system2C periplasmic binding protein TonB  Flagellar motor protein  Flavoredoxin  Metallo-beta-lactamase family protein( EC:3.1.2.6 )  Molybdenum ABC transporter2C periplasmic molybdenum-binding protein ModA (TC 3.A.1.8.1)  Molybdenum transport ATP-binding protein ModC (TC 3.A.1.8.1)  Molybdenum transport system permease protein ModB (TC 3.A.1.8.1)  Proton/glutamate symport protein @ Sodium/glutamate symport protein  TPR domain protein in aerotolerance operon  TonB-dependent receptor  Transcriptional regulator2C ArsR family  Transketolase (EC 2.2.1.1)  conserved hypothetical protein  hypothetical protein  hypothetical protein PA3071  putative membrane protein  transcriptional regulator blaI family |
| 28476 | AGDB01000255.1:1..28476 | ATP-dependent DNA helicase UvrD/PcrA  Cardiolipin synthetase (EC 2.7.8.-)  Cardiolipin synthetase (EC 2.7.8.-)  Cobalt/zinc/cadmium efflux RND transporter2C membrane fusion protein2C CzcB family  Coproporphyrinogen III oxidase2C aerobic (EC 1.3.3.3)  DNA polymerase I (EC 2.7.7.7)  Endonuclease/Exonuclease/phosphatase family protein  FIG01111767: hypothetical protein  FIG01111872: hypothetical protein  FIG01210192: hypothetical protein  FIG01210827: hypothetical protein  Hydroxyacylglutathione hydrolase (EC 3.1.2.6)  LSU ribosomal protein L28p  LSU ribosomal protein L33p  Maltose O-acetyltransferase (EC 2.3.1.79)  PDZ domain family protein  Permeases of the major facilitator superfamily  Transcriptional regulator2C LysR family  Universal stress protein family  hypothetical protein  hypothetical protein  hypothetical protein  hypothetical protein  hypothetical protein  hypothetical protein  membrane protein2C putative  putative secreted protein |
| 28242 | AGDB01000003.1:1..28242 | 1-hydroxy-2-methyl-2-(E)-butenyl 4-diphosphate synthase (EC 1.17.7.1)  Aldehyde dehydrogenase B (EC 1.2.1.22)  Aspartate racemase (EC 5.1.1.13)  Aspartokinase (EC 2.7.2.4) / Homoserine dehydrogenase (EC 1.1.1.3)  Beta-propeller domains of methanol dehydrogenase type  Chaperone protein hscC (Hsc62)  Dihydroorotate dehydrogenase (EC 1.3.3.1)  Dna binding response regulator PrrA (RegA)  FIG01210486: hypothetical protein  Homoserine kinase (EC 2.7.1.39)  Membrane-associated phospholipid phosphatase  Methyltransferase (EC 2.1.1.-)  Periplasmic beta-glucosidase (EC 3.2.1.21)  Predicted sodium-dependent galactose transporter  Sensor histidine kinase PrrB (RegB) (EC 2.7.3.-)  UDP-N-acetylenolpyruvoylglucosamine reductase (EC 1.1.1.158)  diguanylate cyclase/phosphodiesterase (GGDEF  hypothetical protein  hypothetical protein  hypothetical protein  hypothetical protein  hypothetical protein  hypothetical protein  hypothetical protein  integral membrane protein  metallophosphoesterase  tRNA-Arg-CCT |
| 28154 | AGDB01000162.1:1..28154 | 3-oxoacyl-[acyl-carrier protein] reductase (EC 1.1.1.100)  4-carboxymuconolactone decarboxylase (EC 4.1.1.44)  4-hydroxybenzoate polyprenyltransferase (EC 2.5.1.-)  4-oxalocrotonate tautomerase  8-amino-7-oxononanoate synthase (EC 2.3.1.47)  Biosynthetic arginine decarboxylase (EC 4.1.1.19)  Biotin synthase (EC 2.8.1.6)  Biotin synthesis protein bioC  Biotin synthesis protein bioH  Cell division protein FtsN  Competence protein F homolog2C phosphoribosyltransferase domain; protein YhgH required for utilization of DNA as sole source of carbon and energy  FIG01211043: hypothetical protein  GCN5-related N-acetyltransferase  Isopropylmalate/homocitrate/citramalate synthases  MG(2 ) CHELATASE FAMILY PROTEIN / ComM-related protein  Nitrogen regulatory protein P-II  O-antigen acetylase  Oxidoreductase  Polymyxin resistance protein ArnT2C undecaprenyl phosphate-alpha-L-Ara4N transferase; Melittin resistance protein PqaB  Probable transcriptional regulator lrhA  Protein of unknown function DUF218  Spermidine synthase (EC 2.5.1.16)  Threonine dehydratase2C catabolic (EC 4.3.1.19)  TonB-dependent receptor  alpha/beta hydrolase fold  hypothetical protein  hypothetical protein  hypothetical protein  hypothetical protein  hypothetical protein  hypothetical protein  pyridoxamine 5'-phosphate oxidase-related2C FMN-binding  tRNA-Arg-CCG |
| 27983 | AGDB01000238.1:1..27983 | 3-oxoacyl-[acyl-carrier protein] reductase (EC 1.1.1.100)  Acyl-CoA-binding protein  Broad-specificity glycerol dehydrogenase (EC 1.1.99.22)2C subunit SldA  DNA gyrase subunit A (EC 5.99.1.3)  DNA ligase (EC 6.5.1.2)  FIG01210333: hypothetical protein  FIG01210541: hypothetical protein  FIG01211108: hypothetical protein  Formiminoglutamic iminohydrolase (EC 3.5.3.13)  GCN5-related N-acetyltransferase  Histidine ammonia-lyase (EC 4.3.1.3)  Histidine utilization repressor  Imidazolonepropionase (EC 3.5.2.7)  Methylthioribose-1-phosphate isomerase (EC 5.3.1.23)  N-formylglutamate deformylase (EC 3.5.1.68)  Transcriptional regulator2C TetR family  Translation elongation factor P Lys34:lysine transferase  Urocanate hydratase (EC 4.2.1.49)  acetyltransferase2C GNAT family  hypothetical protein  hypothetical protein  hypothetical protein  putative tetratricopeptide repeat family protein |
| 27744 | AGDB01000078.1:1..27744 | ATP-dependent Clp protease ATP-binding subunit ClpX  ATP-dependent Clp protease proteolytic subunit (EC 3.4.21.92)  ATP-dependent protease La (EC 3.4.21.53) Type I  Amidophosphoribosyltransferase (EC 2.4.2.14)  COG2833: uncharacterized protein  Cell division trigger factor (EC 5.2.1.8)  Colicin V production protein  DNA-binding protein HU-alpha  DedD protein  Exopolyphosphatase (EC 3.6.1.11)  FIG01209733: hypothetical protein  FIG01210385: hypothetical protein  FIG01210804: hypothetical protein  FIG01211525: hypothetical protein  Glutaredoxin 3  Glycosyltransferase  Isocitrate dehydrogenase [NAD] (EC 1.1.1.41)  Phosphate regulon sensor protein PhoR (SphS) (EC 2.7.13.3)  Phosphate regulon transcriptional regulatory protein PhoB (SphR)  Polyphosphate kinase (EC 2.7.4.1)  Protease  UDP-22C3-diacylglucosamine hydrolase (EC 3.6.1.-)  hypothetical protein  hypothetical protein  peptidyl-Asp metalloendopeptidase  tRNA-Arg-TCT  tRNA-His-GTG  tRNA-Leu-TAG  tRNA-Lys-CTT  tRNA-Pro-TGG  tRNA-Val-TAC |
| 27381 | AGDB01000034.1:1..27381 | DNA repair protein RadC  FIG01210885: hypothetical protein  FIG01211142: hypothetical protein  FIG01213221: hypothetical protein  FIG01213768: hypothetical protein  Integrase  Succinoglycan biosynthesis protein  endonuclease precursor  hypothetical protein  hypothetical protein  hypothetical protein  hypothetical protein  phage-related integrase  plasmid mobilization protein  virulence regulator |
| 27292 | AGDB01000225.1:1..27292 | 1-acyl-sn-glycerol-3-phosphate acyltransferase (EC 2.3.1.51)  2-keto-3-deoxy-L-fuconate dehydrogenase  22C4-diketo-3-deoxy-L-fuconate hydrolase  Aerobic glycerol-3-phosphate dehydrogenase (EC 1.1.5.3)  Conserved domain protein  ECF sigma factor  Exodeoxyribonuclease III (EC 3.1.11.2)  Fucose permease  Glycerol kinase (EC 2.7.1.30)  Glycerol uptake facilitator protein  Glycerol-3-phosphate regulon repressor2C DeoR family  L-fuco-beta-pyranose dehydrogenase  L-fuconate dehydratase (EC 4.2.1.68)  L-fuconolactone hydrolase  PUTATIVE HYDROLASE PHOSPHATASE PROTEIN( EC:3.1.3.- )  Pirin2C N-terminal:Pirin2C C-terminal  Possible alternative L-fucose mutarotase  Transcriptional regulator2C IclR family  cytochrome C biogenesis protein  hypothetical protein  hypothetical protein  hypothetical protein  hypothetical protein  hypothetical protein  hypothetical protein  hypothetical protein  putative secreted protein |
| 27003 | AGDB01000263.1:1..27003 | 5-formyltetrahydrofolate cyclo-ligase (EC 6.3.3.2)  Azurin  FIG001590: Putative conserved exported protein precursor  FIG01210021: hypothetical protein  FIG01210448: hypothetical protein  FIG017670: hypothetical protein  FIG019278: hypothetical protein  Glutamate-1-semialdehyde aminotransferase (EC 5.4.3.8)  Periplasmic aromatic amino acid aminotransferase beta precursor (EC 2.6.1.57)  Protein of unknown function DUF55  Protein sirB1  Ribose 5-phosphate isomerase A (EC 5.3.1.6)  Rubredoxin  TdcF protein  TesB-like acyl-CoA thioesterase 1  Thiamin-phosphate pyrophosphorylase (EC 2.5.1.3)  Thymidine phosphorylase (EC 2.4.2.4)  TonB-dependent receptor  Transcriptional regulator  Xaa-Pro aminopeptidase (EC 3.4.11.9)  Xaa-Pro dipeptidase PepQ (EC 3.4.13.9)  cytochrome C6  diguanylate cyclase/phosphodiesterase (GGDEF  flavin monoamine oxidase-related protein  hypothetical protein  hypothetical protein  hypothetical protein |
| 26778 | AGDB01000435.1:1..26778 | 3-oxoacyl-[acyl-carrier protein] reductase (EC 1.1.1.100)  ABC transporter ATP-binding protein  Alpha/beta hydrolase fold  Chaperone protein DnaJ  Chaperone protein DnaK  Cyclohexadienyl dehydrogenase (EC 1.3.1.12)(EC 1.3.1.43)  DNA repair protein RecN  Dehydrogenases with different specificities (related to short-chain alcohol dehydrogenases)  FIG000605: protein co-occurring with transport systems (COG1739)  Ferric uptake regulation protein FUR  Glucoamylase (EC 3.2.1.3)  Glucose-methanol-choline (GMC) oxidoreductase:NAD binding site  Heat shock protein GrpE  Heat-inducible transcription repressor HrcA  Outer membrane lipoprotein SmpA2C a component of the essential YaeT outer-membrane protein assembly complex  Putative oligoketide cyclase/dehydratase or lipid transport protein YfjG  Pyridoxal kinase (EC 2.7.1.35)  Serine peptidase  hypothetical protein  hypothetical protein  hypothetical protein  hypothetical protein  tmRNA-binding protein SmpB |
| 26651 | AGDB01000087.1:1..26651 | ATP-dependent hsl protease ATP-binding subunit HslU  ATP-dependent protease HslV (EC 3.4.25.-)  Branched-chain amino acid aminotransferase (EC 2.6.1.42)  Diaminopimelate epimerase (EC 5.1.1.7)  FIG01209776: hypothetical protein  FIG01210375: hypothetical protein  FIG01210600: hypothetical protein  FIG01211065: hypothetical protein  L-asparaginase (EC 3.5.1.1)  NAD(P) transhydrogenase alpha subunit (EC 1.6.1.2)  NAD(P) transhydrogenase alpha subunit (EC 1.6.1.2)  NAD(P) transhydrogenase subunit beta (EC 1.6.1.2)  Prolyl endopeptidase (EC 3.4.21.26)  Protease II (EC 3.4.21.83)  Protein of unknown function DUF484  RNA polymerase sigma-54 factor RpoN  Ribonuclease BN (EC 3.1.-.-)  Site-specific tyrosine recombinase  Trp repressor binding protein  YbaK family protein  extracellular protease  hypothetical protein  hypothetical protein  hypothetical protein  hypothetical protein  hypothetical protein  hypothetical protein  hypothetical protein( EC:2.6.1.1 )  probably aromatic ring hydroxylating enzyme2C evidenced by COGnitor; PaaD-like protein (DUF59) involved in Fe-S cluster assembly |
| 26612 | AGDB01000244.1:1..26612 | 2-hydroxy-3-keto-5-methylthiopentenyl-1-phosphate phosphatase related protein  3-oxoacyl-[acyl-carrier-protein] synthase2C KASII (EC 2.3.1.41)  Acyl carrier protein  Alcohol dehydrogenase (EC 1.1.1.1)  Aminotransferase2C class III  Coproporphyrinogen III oxidase2C oxygen-independent (EC 1.3.99.22)  DegT/DnrJ/EryC1/StrS aminotransferase  FIG01210093: hypothetical protein  FIG01212309: hypothetical protein  FIG01212404: hypothetical protein  FIG01212902: hypothetical protein  Permeases of the drug/metabolite transporter (DMT) superfamily  Permeases of the drug/metabolite transporter (DMT) superfamily  Putative exported protein precursor  Transcriptional regulator2C TetR family  Twin-arginine translocation pathway signal( EC:1.11.1.10 )  Type cbb3 cytochrome oxidase biogenesis protein CcoG2C involved in Cu oxidation  hypothetical protein  hypothetical protein  hypothetical protein  hypothetical protein  hypothetical protein  hypothetical protein  hypothetical protein  putative sec-independent protein translocase protein TatC  putative transmembrane protein  secretion protein HlyD family protein  tRNA-Gly-GCC  tRNA-Leu-TAA  two-component system regulatory protein |
| 26142 | AGDB01000207.1:1..26142 | ATP-dependent DNA ligase (EC 6.5.1.1)  Cellulase  Cellulase  Cellulase  DNA topoisomerase IB (poxvirus type) (EC 5.99.1.2)  Glutamate synthase [NADPH] large chain (EC 1.4.1.13)  Glutamate synthase [NADPH] small chain (EC 1.4.1.13)  Methylglyoxal synthase (EC 4.2.3.3)  Phosphate-binding protein  Putative short-chain dehydrogenase  TonB-dependent receptor  Transcriptional repressor2C BlaI/MecI family  hypothetical protein  hypothetical protein  hypothetical protein  hypothetical protein  hypothetical protein  hypothetical protein  hypothetical protein  putative exported protein |
| 25997 | AGDB01000451.1:1..25997 | 4-carboxymuconolactone decarboxylase domain/alkylhydroperoxidase AhpD family core domain protein  Cold shock protein CspG  Deoxyribodipyrimidine photolyase (EC 4.1.99.3)  FIG01212380: hypothetical protein  Fumarate hydratase class I2C aerobic (EC 4.2.1.2)  GCN5-related N-acetyltransferase  Glutaryl-CoA dehydrogenase (EC 1.3.99.7)  Glutathione S-transferase (EC 2.5.1.18)  Glutathione S-transferase2C unnamed subgroup (EC 2.5.1.18)  HesA/MoeB/ThiF family protein  Outer membrane protein A precursor  Outer membrane receptor proteins2C mostly Fe transport  Peptidoglycan-associated outer membrane lipoprotein  Phospholipase A1 precursor (EC 3.1.1.322C EC 3.1.1.4); Outer membrane phospholipase A  Putative deoxyribonuclease YjjV  Putative oxidoreductase  RNA polymerase ECF-type sigma factor  Replicative DNA helicase (EC 3.6.1.-)  Wax ester synthase/acyl-CoA:diacylglycerol acyltransferase  diguanylate cyclase/phosphodiesterase (GGDEF  hypothetical protein  hypothetical protein  hypothetical protein  probable exported protein YPO1624  probable membrane protein NMA1128  putative secreted protein |
| 25796 | AGDB01000302.1:1..25796 | 2-amino-4-hydroxy-6-hydroxymethyldihydropteridine pyrophosphokinase (EC 2.7.6.3)  6-phosphogluconate dehydrogenase2C decarboxylating (EC 1.1.1.44)  COG1565: Uncharacterized conserved protein  Cell division inhibitor  FIG01111359: hypothetical protein  FIG01209778: hypothetical protein  FIG01210026: hypothetical protein  FIG01210236: hypothetical protein  FIG01210252: hypothetical protein  FolM Alternative dihydrofolate reductase 1  Histidine kinase/response regulator hybrid protein  N-acetylglucosamine-regulated TonB-dependent outer membrane receptor  N-acetylglucosamine-regulated TonB-dependent outer membrane receptor  Osmotically inducible protein OsmY  Phytochrome2C two-component sensor histidine kinase (EC 2.7.3.-)  Putative exported protein  cellulase  hypothetical protein  hypothetical protein  hypothetical protein  hypothetical protein  oxidoreductase  two-component system regulatory protein |
| 25553 | AGDB01000384.1:1..25553 | ABC transporter ATP-binding protein  ABC transporter ATP-binding protein  ABC transporter ATP-binding protein  ABC transporter ATP-binding protein  ABC transporter permease  ABC transporter permease  ABC transporter permease  ABC transporter permease  ABC transporter permease  ABC transporter permease  Cell division protein FtsX  FIG01210028: hypothetical protein  FIG01211682: hypothetical protein  GCN5-related N-acetyltransferase  Glucosamine--fructose-6-phosphate aminotransferase [isomerizing] (EC 2.6.1.16)  Histidine kinase/response regulator hybrid protein  Lipoprotein releasing system ATP-binding protein LolD  N-acetylglucosamine-1-phosphate uridyltransferase (EC 2.7.7.23) / Glucosamine-1-phosphate N-acetyltransferase (EC 2.3.1.157)  Sensor histidine kinase  Transcriptional regulator2C TetR family  hypothetical protein  putative monooxygenase. |
| 25490 | AGDB01000333.1:1..25490 | FIG01209769: hypothetical protein  FIG01212237: hypothetical protein  Os01g0879400  Putative glycosyl hydrolase of unknown function (DUF1680)  TonB-dependent receptor  TonB-dependent receptor  Ureidoglycolate/malate/sulfolactate dehydrogenase family (EC 1.1.1.-)  Xylosidase/arabinosidase ass w/ COG3533  avirulence protein  hypothetical protein  hypothetical protein  hypothetical protein |
| 25378 | AGDB01000006.1:1..25378 | Adenylosuccinate synthetase (EC 6.3.4.4)  Alcohol dehydrogenase (EC 1.1.1.1)  Death on curing protein2C Doc toxin  DnaJ-class molecular chaperone CbpA  Excinuclease ABC subunit A paralog of unknown function  FIG01211223: hypothetical protein  GCN5-related N-acetyltransferase  GGDEF domain protein  Guanosine polyphosphate pyrophosphohydrolases/synthetases  HflC protein  HflK protein  Mrr restriction endonuclease  Type I restriction-modification system2C DNA-methyltransferase subunit M (EC 2.1.1.72)  Type I restriction-modification system2C restriction subunit R (EC 3.1.21.3)  Type I restriction-modification system2C specificity subunit S (EC 3.1.21.3)  hypothetical protein  hypothetical protein  hypothetical protein  hypothetical protein  hypothetical protein  hypothetical protein  twitching motility protein PilH |
| 25373 | AGDB01000218.1:1..25373 | Acid phosphatase (EC 3.1.3.2)  FIG01112077: hypothetical protein  Hemolysin activator protein precursor  Long-chain-fatty-acid--CoA ligase (EC 6.2.1.3)  OmpA-related protein  Orotidine 5'-phosphate decarboxylase (EC 4.1.1.23)  PE-PGRS family protein  Transcriptional regulator2C LacI family  Tryptophan halogenase  hypothetical protein |
| 25081 | AGDB01000165.1:1..25081 | ABC transporter ATP-binding protein  ABC transporter permease  ATPase associated with various cellular activities2C AAA\_5  COG4615: ABC-type siderophore export system2C fused ATPase and permease components  Long-chain-fatty-acid--CoA ligase (EC 6.2.1.3)  Mg-chelatase subunit ChlD  Protein related to penicillin acylase  RND efflux system2C outer membrane lipoprotein CmeC  Two-component response regulator  hypothetical protein  hypothetical protein  hypothetical protein  hypothetical protein  hypothetical protein  hypothetical protein  two-component system sensor protein |
| 24781 | AGDB01000008.1:1..24781 | FIG01210126: hypothetical protein  FIG01210947: hypothetical protein  Polysaccharide deacetylase  Pyruvate dehydrogenase E1 component (EC 1.2.4.1)  RNA:NAD 2'-phosphotransferase  Serine phosphatase RsbU2C regulator of sigma subunit  anti-sigma F factor antagonist  carboxyl-terminal protease( EC:3.4.21.102 )  hypothetical protein  hypothetical protein  hypothetical protein  hypothetical protein  hypothetical protein  hypothetical protein  hypothetical protein  hypothetical protein  putative secreted protein  putative; ORF located using Glimmer/Genemark |
| 24736 | AGDB01000002.1:1..24736 | 2-octaprenyl-3-methyl-6-methoxy-12C4-benzoquinol hydroxylase (EC 1.14.13.-)  2-octaprenyl-6-methoxyphenol hydroxylase (EC 1.14.13.-)  4-hydroxythreonine-4-phosphate dehydrogenase (EC 1.1.1.262)  ApaG protein  Bis(5'-nucleosyl)-tetraphosphatase2C symmetrical (EC 3.6.1.41)  Cob(I)alamin adenosyltransferase PduO (EC 2.5.1.17)  Dihydrofolate reductase (EC 1.5.1.3)  Dimethyladenosine transferase (EC 2.1.1.-)  FIG004694: Hypothetical protein  FIG01212910: hypothetical protein  FIG01213330: hypothetical protein  Gluconolactonase  Optional hypothetical component of the B12 transporter BtuM  Outer membrane protein Imp2C required for envelope biogenesis / Organic solvent tolerance protein precursor  Prolipoprotein diacylglyceryl transferase (EC 2.4.99.-)  Putative RNA 2'-O-ribose methyltransferase mtfA (EC 2.1.1.-)  Survival protein SurA precursor (Peptidyl-prolyl cis-trans isomerase SurA) (EC 5.2.1.8)  Thymidylate synthase (EC 2.1.1.45)  acetoin utilization family protein  cytidine and deoxycytidylate deaminase family protein  glucose-fructose oxidoreductase  hypothetical protein  hypothetical protein  hypothetical protein  hypothetical protein  hypothetical protein  integral membrane protein  transcriptional regulator |
| 23940 | AGDB01000405.1:1..23940 | 23S rRNA (guanine-N-2-) -methyltransferase rlmL EC 2.1.1.-)  ABC transporter ATP-binding protein  Carbonic anhydrase (EC 4.2.1.1)  Endonuclease III (EC 4.2.99.18)  Enoyl-CoA hydratase (EC 4.2.1.17)  FIG00506329: hypothetical protein  FIG01111044: hypothetical protein  FIG01209703: hypothetical protein  FIG01209728: hypothetical protein  FIG01209903: hypothetical protein  Hypothetical nudix hydrolase YeaB  Macrophage infectivity potentiator  Negative regulator of beta-lactamase expression  Phosphate ABC transporter2C periplasmic phosphate-binding protein PstS (TC 3.A.1.7.1)  Phosphate ABC transporter2C periplasmic phosphate-binding protein PstS (TC 3.A.1.7.1)  Sulfate transporter family protein in cluster with carbonic anhydrase  Thiosulfate sulfurtransferase2C rhodanese (EC 2.8.1.1)  acetyltransferase  hypothetical protein  hypothetical protein  hypothetical protein  hypothetical protein  two-component system regulatory protein |
| 23671 | AGDB01000208.1:1..23671 | 2-hydroxychromene-2-carboxylate isomerase/DsbA-like thioredoxin domain  23S rRNA (Uracil-5-) -methyltransferase RumA (EC 2.1.1.-)  3-ketoacyl-CoA thiolase (EC 2.3.1.16) @ Acetyl-CoA acetyltransferase (EC 2.3.1.9)  5'-methylthioadenosine phosphorylase (EC 2.4.2.28)  ATP-dependent DNA ligase (EC 6.5.1.1) LigC  Acyl-CoA dehydrogenase (EC 1.3.99.3)  Beta N-acetyl-glucosaminidase (EC 3.2.1.52)  Cold shock protein CspD  DNA recombination and repair protein RecO  FIG003033: Helicase domain protein  FIG006285: hypothetical protein  FIG01210304: hypothetical protein  FIG01211136: hypothetical protein  FIG01212761: hypothetical protein  Hypoxanthine-guanine phosphoribosyltransferase (EC 2.4.2.8)  Oxidoreductase  Response regulator  hypothetical protein  hypothetical protein  mRNA 3-end processing factor  outer membrane protein  sensor histidine kinase |
| 23401 | AGDB01000033.1:1..23401 | 2-dehydropantoate 2-reductase (EC 1.1.1.169)  ABC-type multidrug transport system2C ATPase component  Deoxyribonuclease TatD  FIG00457326: hypothetical protein  FIG01209941: hypothetical protein  FIG01211213: hypothetical protein  Flp pilus assembly protein RcpC/CpaB  Flp pilus assembly protein TadB  Outer membrane protein  Transcriptional regulator2C Cro/CI family  Type II/IV secretion system ATP hydrolase TadA/VirB11/CpaF2C TadA subfamily  Type II/IV secretion system protein TadC2C associated with Flp pilus assembly  hypothetical protein  hypothetical protein  hypothetical protein  hypothetical protein  hypothetical protein  hypothetical protein  hypothetical protein  hypothetical protein  permease  phenol hydroxylase  type II and III secretion system protein |
| 23313 | AGDB01000188.1:1..23313 | Decarboxylase family protein  Excinuclease ABC subunit B  FIG01211666: hypothetical protein  Forms the bulk of type IV secretion complex that spans outer membrane and periplasm (VirB9)  Inner membrane protein forms channel for type IV secretion of T-DNA complex (VirB8)  TonB-dependent receptor  Type IV fimbrial biogenesis protein FimT  Type IV fimbrial biogenesis protein PilV  Type IV fimbrial biogenesis protein PilW  Type IV fimbrial biogenesis protein PilX  Type IV fimbrial biogenesis protein PilY1  Type IV pilus biogenesis protein PilE  Type IV secretion system protein VirD4  alginate biosynthesis protein  fimbrial biogenesis protein  hypothetical protein  hypothetical protein  hypothetical protein  tRNA-Asn-GTT  tRNA-Val-GAC |
| 23300 | AGDB01000175.1:1..23300 | Acetyltransferase  Aspartokinase (EC 2.7.2.4) / Diaminopimelate decarboxylase (EC 4.1.1.20)  Cysteine desulfurase (EC 2.8.1.7)2C SufS subfamily  Dienelactone hydrolase family protein  FIG01209781: hypothetical protein  FIG01210076: hypothetical protein  FIG01210339: hypothetical protein  Ferredoxin2C 2Fe-2S  Histone-like protein  Iron-sulfur cluster assembly ATPase protein SufC  Iron-sulfur cluster assembly protein SufB  Iron-sulfur cluster assembly protein SufD  Iron-sulfur cluster regulator IscR  Octaprenyl-diphosphate synthase (EC 2.5.1.-) / Dimethylallyltransferase (EC 2.5.1.1) / Geranyltranstransferase (farnesyldiphosphate synthase) (EC 2.5.1.10) / Geranylgeranyl pyrophosphate synthetase (EC 2.5.1.29)  Organic hydroperoxide resistance protein  Phenazine biosynthesis protein PhzF like  Proteins containing SET domain  ThiJ/PfpI family protein  UDP-N-acetylmuramoylalanine--D-glutamate ligase (EC 6.3.2.9)  hypothetical protein  hypothetical protein  hypothetical protein  peptidase2C M23/M37 family  putative membrane protein |
| 22435 | AGDB01000100.1:1..22435 | Long-chain-fatty-acid--CoA ligase (EC 6.2.1.3)  Long-chain-fatty-acid--CoA ligase (EC 6.2.1.3) |
| 22405 | AGDB01000204.1:1..22405 | 16S rRNA processing protein RimM  Aminodeoxychorismate lyase (EC 4.1.3.38)  Dephospho-CoA kinase (EC 2.7.1.24)  Enoyl-[acyl-carrier-protein] reductase [FMN] (EC 1.3.1.9)  Glucose dehydrogenase2C PQQ-dependent (EC 1.1.5.2)  LSU ribosomal protein L19p  Leader peptidase (Prepilin peptidase) (EC 3.4.23.43) / N-methyltransferase (EC 2.1.1.-)  NAD synthetase (EC 6.3.1.5) / Glutamine amidotransferase chain of NAD synthetase  Na -driven multidrug efflux pump  Ribosome-associated heat shock protein implicated in the recycling of the 50S subunit (S4 paralog)  SSU ribosomal protein S16p  Succinyl-CoA ligase [ADP-forming] alpha chain (EC 6.2.1.5)  Succinyl-CoA ligase [ADP-forming] beta chain (EC 6.2.1.5)  Two-component sensor PilS  Type IV fimbriae expression regulatory protein PilR  Type IV fimbrial assembly protein PilC  Type IV fimbrial assembly2C ATPase PilB  Type IV pilin PilA  hypothetical protein  hypothetical protein  tRNA (Guanine37-N1) -methyltransferase (EC 2.1.1.31) |
| 22321 | AGDB01000428.1:1..22321 | Acyl-CoA dehydrogenase2C short-chain specific (EC 1.3.99.2)  Aminomethyltransferase (glycine cleavage system T protein) (EC 2.1.2.10)  Aspartyl/asparaginyl beta-hydroxylase and related dioxygenase  Beta-lactamase (EC 3.5.2.6)  COG0457: FOG: TPR repeat  Choline dehydrogenase (EC 1.1.99.1)  FIG01113126: hypothetical protein  FIG01209855: hypothetical protein  FIG01211173: hypothetical protein  FIG01211374: hypothetical protein  Glycine cleavage system H protein  TonB-dependent receptor  Transcriptional regulator2C TetR family  histone H1  hypothetical protein  hypothetical protein |
| 22011 | AGDB01000254.1:1..22011 | (3R)-hydroxymyristoyl-[ACP] dehydratase (EC 4.2.1.-)  3-hydroxydecanoyl-[ACP] dehydratase (EC 4.2.1.60)  3-oxoacyl-[ACP] reductase (EC 1.1.1.100)  Acyl carrier protein (ACP1)  Chlorogenate esterase  Dipeptidyl peptidase IV  FIG017861: hypothetical protein  FIG018329: 1-acyl-sn-glycerol-3-phosphate acyltransferase  FIG021862: membrane protein2C exporter  FIG022199: FAD-binding protein  FIG027190: Putative transmembrane protein  FIG143263: Glycosyl transferase @ Dolichyl-phosphate mannose synthase related protein  FIGfam138462: Acyl-CoA synthetase2C AMP-(fatty) acid ligase  Halogenase  Lysophospholipid acyltransferase  glucokinase  pteridine-dependent deoxygenase like protein  putative Cytochrome bd22C subunit I  putative Cytochrome bd22C subunit II |
| 21897 | AGDB01000341.1:1..21897 | ABC transporter2C transmembrane region:ABC transporter:Peptidase C392C bacteriocin processing  Cytochrome d ubiquinol oxidase subunit I (EC 1.10.3.-)  Cytochrome d ubiquinol oxidase subunit II (EC 1.10.3.-)  FIG00957062: hypothetical protein  FIG01211915: hypothetical protein  Glutaryl-7-ACA acylase  HlyD family secretion protein  Transport ATP-binding protein CydD  hypothetical protein  hypothetical protein  hypothetical protein  hypothetical protein  hypothetical protein  tRNA-Pro-CGG |
| 21808 | AGDB01000338.1:1..21808 | 2-C-methyl-D-erythritol 22C4-cyclodiphosphate synthase (EC 4.6.1.12)  2-C-methyl-D-erythritol 4-phosphate cytidylyltransferase (EC 2.7.7.60)  2-Keto-3-deoxy-D-manno-octulosonate-8-phosphate synthase (EC 2.5.1.55)  CDP-diacylglycerol pyrophosphatase (EC 3.6.1.26)  CTP synthase (EC 6.3.4.2)  Carboxypeptidase C (cathepsin A)  Cell division protein FtsB  Enolase (EC 4.2.1.11)  ExoD protein  Hemolysins and related proteins containing CBS domains  Luciferase-like  Probable low-affinity inorganic phosphate transporter  Putative FecR  TonB-dependent ferrichrome-iron receptor  Topoisomerase IV subunit B (EC 5.99.1.-)  hypothetical protein  hypothetical protein  hypothetical protein  hypothetical protein  hypothetical protein  nodulation protein L  tRNA pseudouridine 13 synthase (EC 4.2.1.-) |
| 21759 | AGDB01000292.1:1..21759 | CoA tranferase  DUF1022 domain-containing protein  Glutamate-ammonia-ligase adenylyltransferase (EC 2.7.7.42)  Glycerophosphoryl diester phosphodiesterase (EC 3.1.4.46)  L-Proline/Glycine betaine transporter ProP  Nitroreductase  Protein yceI precursor  Putative preQ0 transporter  TonB-dependent receptor  hypothetical protein  hypothetical protein  hypothetical protein  hypothetical protein  hypothetical protein  proteinase  putative RecF protein |
| 21604 | AGDB01000107.1:1..21604 | 1-acyl-sn-glycerol-3-phosphate acyltransferase (EC 2.3.1.51)  CDP-diacylglycerol--glycerol-3-phosphate 3-phosphatidyltransferase (EC 2.7.8.5)  FIG00509706: hypothetical protein  FIG01111853: hypothetical protein  FIG01209718: hypothetical protein  FIG01211385: hypothetical protein  IMP cyclohydrolase (EC 3.5.4.10) / Phosphoribosylaminoimidazolecarboxamide formyltransferase (EC 2.1.2.3)  Interferon-induced transmembrane protein  Interferon-induced transmembrane protein  NADH pyrophosphatase (EC 3.6.1.22)  Phosphoribosylamine--glycine ligase (EC 6.3.4.13)  Putative lipase in cluster with Phosphatidate cytidylyltransferase  Ser/Thr and Tyr protein phosphatase (dual specificity)  Transmembrane protein  conserved hypothetical protein  hypothetical protein  hypothetical protein  transcriptional regulator lysR family  transmembrane protein |
| 21402 | AGDB01000105.1:1..21402 | ABC transporter related  ABC-2 type transporter  Acriflavin resistance protein  Catalase (EC 1.11.1.6)  FIG01111989: hypothetical protein  Isovaleryl-CoA dehydrogenase (EC 1.3.99.10)  Methylcrotonyl-CoA carboxylase carboxyl transferase subunit (EC 6.4.1.4)  Na( ) H( ) antiporter subunit E  Na( ) H( ) antiporter subunit F  Na( ) H( ) antiporter subunit G  Nuclease  Predicted transcriptional regulator LiuX of leucine degradation pathway2C AcrR family  Putative OMR family iron-siderophore receptor precursor  Sulfite dehydrogenase cytochrome subunit SoxD  cytochrome B561  diguanylate cyclase  hypothetical protein  hypothetical protein  hypothetical protein  two-component system regulatory protein  two-component system sensor protein |
| 21161 | AGDB01000028.1:1..21161 | Alpha-L-fucosidase (EC 3.2.1.51)  Beta-galactosidase (EC 3.2.1.23)  Beta-galactosidase (EC 3.2.1.23)  Cobalt-zinc-cadmium resistance protein CzcD  DNA mismatch repair protein MutS  Enoyl-CoA hydratase (EC 4.2.1.17) / 3-hydroxyacyl-CoA dehydrogenase (EC 1.1.1.35) / 3-hydroxybutyryl-CoA epimerase (EC 5.1.2.3)  LysR family transcriptional regulator YeiE  Putative membrane protein YeiH  RNA polymerase sigma factor RpoE  Threonine dehydrogenase and related Zn-dependent dehydrogenases  TonB-dependent receptor  hypothetical protein  hypothetical protein  hypothetical protein |
| 21072 | AGDB01000385.1:1..21072 | 3-hydroxydecanoyl-[acyl-carrier-protein] dehydratase (EC 4.2.1.60)  3-oxoacyl-[acyl-carrier-protein] synthase2C KASI (EC 2.3.1.41)  Copper resistance protein B  Copper resistance protein CopZ  Cysteine synthase B (EC 2.5.1.47)  DNA polymerase IV (EC 2.7.7.7)  Dethiobiotin synthetase (EC 6.3.3.3)  FIG01210979: hypothetical protein  Free methionine-(R)-sulfoxide reductase2C contains GAF domain  Lead2C cadmium2C zinc and mercury transporting ATPase (EC 3.6.3.3) (EC 3.6.3.5); Copper-translocating P-type ATPase (EC 3.6.3.4)  Multicopper oxidase  Oligopeptidase A (EC 3.4.24.70)  Outer membrane vitamin B12 receptor BtuB  Phosphoglycolate phosphatase (EC 3.1.3.18)  PxORF73 peptide  Queuosine biosynthesis QueD2C PTPS-I  TfoX C-terminal domain superfamily  hypothetical protein  hypothetical protein  transcriptional regulator2C MerR family |
| 20807 | AGDB01000330.1:1..20807 | 6-phosphofructokinase (EC 2.7.1.11)  Acetylornithine aminotransferase (EC 2.6.1.11)  Adenylate kinase (EC 2.7.4.3)  DedA protein  FIG01210548: hypothetical protein  FIG01210920: hypothetical protein  FIG01211770: hypothetical protein  Inorganic pyrophosphatase (EC 3.6.1.1)  Lactoylglutathione lyase (EC 4.4.1.5)  Potassium voltage-gated channel subfamily KQT; possible potassium channel2C VIC family  Pyrophosphate-energized proton pump (EC 3.6.1.1)  Short chain dehydrogenase  UDP-N-acetylmuramate:L-alanyl-gamma-D-glutamyl-meso-diaminopimelate ligase (EC 6.3.2.-)  Uncharacterized protein2C similar to the N-terminal domain of Lon protease  hypothetical protein  hypothetical protein  hypothetical protein  hypothetical protein  response regulator  transcriptional regulator |
| 20289 | AGDB01000423.1:1..20289 | Aspartate carbamoyltransferase (EC 2.1.3.2)  COG1801: Uncharacterized conserved protein  Cytochrome c family protein  DNA-3-methyladenine glycosylase (EC 3.2.2.20)  Dehydrogenase flavoprotein LodB  FIG01209760: hypothetical protein  Hypothetical protein YggS2C proline synthase co-transcribed bacterial homolog PROSC  Lysine-epsilon oxidase (EC 1.4.3.20) antimicrobial protein LodA  Putative Holliday junction resolvase (EC 3.1.-.-)  Response regulator containing a CheY-like receiver domain and a GGDEF domain  Transporter  Twitching motility protein PilT  Twitching motility protein PilT  UPF0301 protein YqgE  hypothetical protein  hypothetical protein  putative secreted protein |
| 20005 | AGDB01000440.1:1..20005 | Beta-lactamase (EC 3.5.2.6)  FIG01211686: hypothetical protein  Glutathione-regulated potassium-efflux system ATP-binding protein  Glycine cleavage system transcriptional activator  Na /H exchange protein  Phospholipase C  TonB-dependent receptor  TonB-dependent receptor  TonB-dependent receptor  Type I antifreeze protein  Xylanase  hypothetical protein  hypothetical protein  hypothetical protein |
| 19864 | AGDB01000434.1:1..19864 | 2-oxoglutarate dehydrogenase E1 component (EC 1.2.4.2)  Adenylosuccinate lyase (EC 4.3.2.2)  Dihydrolipoamide dehydrogenase of 2-oxoglutarate dehydrogenase (EC 1.8.1.4)  Dihydrolipoamide succinyltransferase component (E2) of 2-oxoglutarate dehydrogenase complex (EC 2.3.1.61)  FIG002776: hypothetical protein  FIG01111324: hypothetical protein  FIG01111779: hypothetical protein  FIG01211089: hypothetical protein  Fumarate hydratase class II (EC 4.2.1.2)  Lysine decarboxylase family  RNA polymerase sigma-70 factor  hypothetical protein  hypothetical protein  hypothetical protein |
| 19837 | AGDB01000193.1:1..19837 | FIG000233: metal-dependent hydrolase  FIG01209757: hypothetical protein  FIG01209779: hypothetical protein  FIG01210646: hypothetical protein  Glutathione S-transferase (EC 2.5.1.18)  Inner membrane component of tripartite multidrug resistance system  Magnesium and cobalt efflux protein CorC  Magnesium and cobalt transport protein CorA  OsmC/Ohr family protein  Outer membrane component of tripartite multidrug resistance system  Phosphate starvation-inducible ATPase PhoH with RNA binding motif  Putrescine transport ATP-binding protein PotG (TC 3.A.1.11.2)  Putrescine transport system permease protein PotH (TC 3.A.1.11.2)  Putrescine transport system permease protein PotI (TC 3.A.1.11.2)  Succinate-semialdehyde dehydrogenase [NAD] (EC 1.2.1.24); Succinate-semialdehyde dehydrogenase [NADP ] (EC 1.2.1.16)  Transcriptional regulator2C ArsR family  hypothetical protein  hypothetical protein  tRNA-i(6)A37 methylthiotransferase |
| 19810 | AGDB01000346.1:1..19810 | 2-octaprenyl-3-methyl-6-methoxy-12C4-benzoquinol hydroxylase (EC 1.14.13.-)  ADA regulatory protein / Methylated-DNA--protein-cysteine methyltransferase (EC 2.1.1.63)  Ethidium bromide-methyl viologen resistance protein EmrE  FIG01210006: hypothetical protein  FIG01210022: hypothetical protein  FIG01210449: hypothetical protein  FIG01211728: hypothetical protein  FIG01217340: hypothetical protein  Inner membrane protein  Methylated-DNA--protein-cysteine methyltransferase (EC 2.1.1.63)  Periplasmic aromatic aldehyde oxidoreductase2C FAD binding subunit YagS  Periplasmic aromatic aldehyde oxidoreductase2C iron-sulfur subunit YagT  TonB-dependent receptor  Xanthine dehydrogenase2C molybdenum binding subunit (EC 1.17.1.4)  Zinc-regulated outer membrane receptor  chloride channel  hypothetical protein  hypothetical protein  hypothetical protein  hypothetical protein |
| 19035 | AGDB01000177.1:1..19035 | 4Fe-4S ferredoxin2C iron-sulfur binding  Acriflavin resistance protein  Chaperone protein HtpG  DNA topology modulation protein FLAR-related protein  DNA-binding heavy metal response regulator  FIG01212077: hypothetical protein  Gamma-glutamyltranspeptidase (EC 2.3.2.2)  Heavy metal sensor histidine kinase  Phosphopantetheine adenylyltransferase (EC 2.7.7.3)  Probable Co/Zn/Cd efflux system membrane fusion protein  RND efflux system2C outer membrane lipoprotein2C NodT family  Ribosomal RNA small subunit methyltransferase D (EC 2.1.1.-)  Zn-dependent hydrolases2C including glyoxylases  nucleoside-diphosphate-sugar epimerases |
| 18888 | AGDB01000441.1:1..18888 | 4-hydroxy-2-oxovalerate aldolase  MASE1 domain protein  Outer membrane receptor proteins2C mostly Fe transport  PsiF  Vibrioferrin amide bond forming protein PvsB @ Siderophore synthetase superfamily2C group B  Vibrioferrin amide bond forming protein PvsD @ Siderophore synthetase superfamily2C group A  Vibrioferrin decarboxylase protein PvsE  Vibrioferrin ligase/carboxylase protein PvsA  Vibrioferrin membrane-spanning transport protein PvsC  Vibrioferrin receptor PvuA  hypothetical protein  hypothetical protein  hypothetical protein  hypothetical protein  hypothetical protein  hypothetical protein  hypothetical protein  putative Glutathione-regulated potassium-efflux system protein KefB |
| 18773 | AGDB01000047.1:1..18773 | CDP-glycerol: N-acetyl-beta-D-mannosaminyl-12C4-N-acetyl-D-glucosaminyldiphosphoundecaprenyl glycerophosphotransferase  DNA topoisomerase I (EC 5.99.1.2)  FIG01210152: hypothetical protein  Fimbrial protein precursor  Lipid A core - O-antigen ligase and related enzymes  Methionyl-tRNA formyltransferase (EC 2.1.2.9)  Peptide deformylase (EC 3.5.1.88)  Polymyxin resistance protein ArnT2C undecaprenyl phosphate-alpha-L-Ara4N transferase; Melittin resistance protein PqaB  Protein of unknown function Smg  Ribosomal RNA small subunit methyltransferase B (EC 2.1.1.-)  Rossmann fold nucleotide-binding protein Smf possibly involved in DNA uptake  Sua5 YciO YrdC YwlC family protein  Uncharacterized protein with LysM domain2C COG1652  beta 12C4 glucosyltransferase  conserved hypothetical protein |
| 18535 | AGDB01000116.1:1..18535 | Asp-tRNAAsn/Glu-tRNAGln amidotransferase A subunit and related amidases  COG21102C Macro domain2C possibly ADP-ribose binding module  Gamma-glutamyltranspeptidase (EC 2.3.2.2)  Hemolysin activation/secretion protein associated with VreARI signalling system  Inosine-uridine preferring nucleoside hydrolase (EC 3.2.2.1)  Large exoproteins involved in heme utilization or adhesion  hypothetical protein  hypothetical protein  hypothetical protein  putative signal transduction histidine kinase  transcriptional regulator lysR family |
| 18527 | AGDB01000074.1:1..18527 | 12C4-alpha-glucan (glycogen) branching enzyme2C GH-13-type (EC 2.4.1.18)  Autolysis response regulater LytR  FIG01211032: hypothetical protein  Glucans biosynthesis glucosyltransferase H (EC 2.4.1.-)  Glycogen synthase2C ADP-glucose transglucosylase (EC 2.4.1.21)  Isochorismatase (EC 3.3.2.1)  Malto-oligosyltrehalose trehalohydrolase (EC 3.2.1.141)  Phospholipase/Carboxylesterase  Porphobilinogen deaminase (EC 2.5.1.61)  Redox-sensing transcriptional regulator QorR  Signal transduction histidine kinase CheA (EC 2.7.3.-)  hypothetical protein  hypothetical protein  hypothetical protein  hypothetical protein  outer membrane lipoprotein  protein of unknown function DUF1486  putative salt-induced outer membrane protein  single-domain response regulator |
| 18513 | AGDB01000053.1:1..18513 | ATPase involved in DNA repair  FIG01112335: hypothetical protein  TonB-dependent receptor  hypothetical protein  hypothetical protein  hypothetical protein  hypothetical protein  hypothetical protein  hypothetical protein  hypothetical protein  hypothetical protein  hypothetical protein  hypothetical protein  hypothetical protein  hypothetical protein  hypothetical protein  hypothetical protein  hypothetical protein  hypothetical protein |
| 18305 | AGDB01000138.1:1..18305 | Aminotransferase  Cytochrome c-type biogenesis protein CcmC2C putative heme lyase for CcmE  Cytochrome c-type biogenesis protein CcmE2C heme chaperone  Cytochrome c2  FIG01209798: hypothetical protein  FIG01210230: hypothetical protein  FIG01210267: hypothetical protein  FIG01210424: hypothetical protein  FIG01211823: hypothetical protein  FIG01213081: hypothetical protein  FIG01214411: hypothetical protein  RNA polymerase sigma-E factor  cytochrome C  cytochrome like B561  glycosyl transferase  hypothetical protein  lipopolysaccharide biosynthesis protein  serine protease  transferase2C putative |
| 18251 | AGDB01000018.1:1..18251 | Alpha/beta hydrolase fold (EC 3.8.1.5)  Beta-galactosidase (EC 3.2.1.23)  Cyclohexadienyl dehydratase (EC 4.2.1.51)(EC 4.2.1.91)  D-serine/D-alanine/glycine transporter  M. jannaschii predicted coding region MJ1233  Magnesium and cobalt transport protein  Membrane protein2C putative  NAD(P)H oxidoreductase YRKL (EC 1.6.99.-) @ Putative NADPH-quinone reductase (modulator of drug activity B) @ Flavodoxin 2  Transcriptional regulator  Transcriptional regulator2C AraC family  Xylanase  hypothetical protein  hypothetical protein  hypothetical protein  hypothetical protein  hypothetical protein  hypothetical protein  hypothetical protein  inner membrane protein  putative lipoprotein |
| 18107 | AGDB01000039.1:1..18107 | DNA recombination-dependent growth factor C  FIG01210461: hypothetical protein  FIG01212167: hypothetical protein  Ferrochelatase2C protoheme ferro-lyase (EC 4.99.1.1)  Methyl-accepting chemotaxis protein I (serine chemoreceptor protein)  Twin-arginine translocation protein TatA  Zinc metalloprotease (EC 3.4.24.-)  conserved hypothetical protein protein  hydrolase2C alpha/beta fold family  hypothetical protein  hypothetical protein  hypothetical protein  hypothetical protein  hypothetical protein  hypothetical protein  hypothetical protein  photolyase protein family  tRNA(Cytosine32)-2-thiocytidine synthetase |
| 18044 | AGDB01000366.1:1..18044 | ABC-type multidrug transport system2C ATPase component  ABC-type multidrug transport system2C permease component  Biotin synthase related domain containing protein  Domain often clustered or fused with uracil-DNA glycosylase / Uracil-DNA glycosylase2C putative family 6  FIG01210488: hypothetical protein  FIG01211407: hypothetical protein  Leucine-responsive regulatory protein2C regulator for leucine (or lrp) regulon and high-affinity branched-chain amino acid transport system  Na /H antiporter  Oxidoreductase  Permease of the drug/metabolite transporter (DMT) superfamily  Putative cytoplasmic protein  Sensory subunit of low CO2-induced protein complex2C putative  Transcriptional regulator2C LysR family  Transcriptional regulator2C LysR family  carboxymuconolactone decarboxylase family protein  hypothetical protein  tryptophan repressor binding protein  unknown |
| 17959 | AGDB01000206.1:1..17959 | Aldose 1-epimerase (EC 5.1.3.3)  Alpha-N-arabinofuranosidase 2 (EC 3.2.1.55)  Cation:proton antiporter  FIG01132299: hypothetical protein  Ribose ABC transport system2C permease protein RbsC (TC 3.A.1.2.1)  Sugar transport ATP-binding protein  Translation elongation factor G-related protein  Uncharacterized glutathione S-transferase-like protein  hypothetical protein  hypothetical protein  hypothetical protein  hypothetical protein  inner-membrane translocator  peptidase M192C renal dipeptidase |
| 17868 | AGDB01000317.1:1..17868 | Aromatic hydrocarbon utilization transcriptional regulator CatR (LysR family)  Catechol 12C2-dioxygenase (EC 1.13.11.1)  FIG01210350: hypothetical protein  FIG01210755: hypothetical protein  FIG01211451: hypothetical protein  G:T/U mismatch-specific uracil/thymine DNA-glycosylase  Membrane protein involved in aromatic hydrocarbon degradation  Methionine ABC transporter ATP-binding protein  Methionine ABC transporter permease protein  Methionine ABC transporter substrate-binding protein  Muconate cycloisomerase (EC 5.5.1.1)  Muconolactone isomerase (EC 5.3.3.4)  Putative n-hydroxybenzoate hydroxylase  Ribosomal small subunit pseudouridine synthase A (EC 4.2.1.70)  UPF0234 protein YajQ  hypothetical protein  hypothetical protein  hypothetical protein  outer membrane protein  ribosomal RNA small subunit methyltransferase C  salicylate esterase |
| 17672 | AGDB01000026.1:1..17672 | Cytoplasmic axial filament protein CafA and Ribonuclease G (EC 3.1.4.-)  DNA polymerase III delta subunit (EC 2.7.7.7)  DnaJ domain protein  Ferric siderophore transport system2C periplasmic binding protein TonB  Iojap protein  LPS-assembly lipoprotein RlpB precursor (Rare lipoprotein B)  LSU m3Psi1915 methyltransferase RlmH  Leucyl-tRNA synthetase (EC 6.1.1.4)  Nicotinate-nucleotide adenylyltransferase (EC 2.7.7.18)  Protein of unknown function DUF541  Septum formation protein Maf  TonB-dependent receptor  hypothetical protein  hypothetical protein  hypothetical protein |
| 17670 | AGDB01000135.1:1..17670 | 3-oxoacyl-[acyl-carrier-protein] synthase2C KASIII (EC 2.3.1.41)  Acetyltransferase  Acetyltransferase (isoleucine patch superfamily)  Acyl carrier protein  Aminotransferase2C DegT/DnrJ/EryC1/StrS family  DNA-binding response regulator2C LuxR family  FIG00959623: hypothetical protein  FIG01211124: hypothetical protein  Flagellar biosynthesis protein FliC  Flagellar biosynthesis protein FliS  Flagellar hook-associated protein FliD  Flagellar regulatory protein FleQ  GbcA Glycine betaine demethylase subunit A  Oxidoreductase2C short chain dehydrogenase/reductase family  RNA polymerase sigma-54 factor RpoN  hypothetical protein  hypothetical protein  response regulator  short chain dehydrogenase |
| 17320 | AGDB01000303.1:1..17320 | Alpha-N-acetylglucosaminidase (EC 3.2.1.50)  Beta-galactosidase (EC 3.2.1.23)  GTPase and tRNA-U34 5-formylation enzyme TrmE  Glucosamine-6-phosphate deaminase [isomerizing]2C alternative (EC 3.5.99.6)  N-acetyl glucosamine transporter2C NagP  N-acetylglucosamine related transporter2C NagX  N-acetylglucosamine-6-phosphate deacetylase (EC 3.5.1.25)  Predicted transcriptional regulator of N-Acetylglucosamine utilization2C GntR family  Predicted transcriptional regulator of N-Acetylglucosamine utilization2C LacI family  Soluble lytic murein transglycosylase precursor (EC 3.2.1.-)  hypothetical protein  hypothetical protein  tRNA nucleotidyltransferase (EC 2.7.7.21) (EC 2.7.7.25) |
| 16639 | AGDB01000242.1:1..16639 | BarA-associated response regulator UvrY ( GacA SirA)  Beta-ketoadipate enol-lactone hydrolase (EC 3.1.1.24)  FIG00964901: hypothetical protein  GCN5-related N-acetyltransferase  Transcriptional regulator2C LysR family  Uncharacterized protein conserved in bacteria  alginate regulatory protein AlgP  hypothetical protein  hypothetical protein  hypothetical protein  hypothetical protein  hypothetical protein  hypothetical protein  hypothetical protein  hypothetical protein  hypothetical protein  hypothetical protein  tRNA-Ser-TGA |
| 16466 | AGDB01000042.1:1..16466 | 3-oxoacyl-[acyl-carrier protein] reductase (EC 1.1.1.100)  Aromatic-amino-acid aminotransferase (EC 2.6.1.57)  Biopolymer transport protein ExbD/TolR  Biopolymer transport protein ExbD/TolR  Biopolymer transport protein ExbD/TolR  Cardiolipin synthetase (EC 2.7.8.-)  FIG01212315: hypothetical protein  Fructose-12C6-bisphosphatase2C type I (EC 3.1.3.11)  MotA/TolQ/ExbB proton channel family protein  Pyridoxine 5'-phosphate synthase (EC 2.6.99.2)  Radical SAM domain protein  TonB-dependent receptor  hypothetical protein  hypothetical protein  hypothetical protein  hypothetical protein  hypothetical protein  hypothetical protein |
| 16354 | AGDB01000102.1:1..16354 | DNA-directed RNA polymerase beta subunit (EC 2.7.7.6)  DNA-directed RNA polymerase beta' subunit (EC 2.7.7.6)  LSU ribosomal protein L10p (P0)  LSU ribosomal protein L11p (L12e)  LSU ribosomal protein L1p (L10Ae)  LSU ribosomal protein L7/L12 (P1/P2)  Preprotein translocase subunit SecE (TC 3.A.5.1.1)  SSU ribosomal protein S12p (S23e)  SSU ribosomal protein S7p (S5e)  Transcription antitermination protein NusG  Translation elongation factor G  tRNA-Trp-CCA |
| 16291 | AGDB01000345.1:1..16291 | Carbon-nitrogen hydrolase  Glutamyl-tRNA synthetase (EC 6.1.1.17)  Histone acetyltransferase HPA2 and related acetyltransferases  Inner membrane protein CreD  Protein RtcB  RNA 3'-terminal phosphate cyclase (EC 6.5.1.4)  TROVE domain protein  Transcriptional regulatory protein RtcR  Zinc uptake regulation protein ZUR  hypothetical protein  hypothetical protein  hypothetical protein  hypothetical protein  putative cytoplasmic protein  tRNA-Gly-TCC |
| 16288 | AGDB01000040.1:1..16288 | ABC-type Na efflux pump2C permease component  ABC-type Na transport system2C ATPase component  Dipeptidyl peptidase IV  FIG01112087: hypothetical protein  FIG01210241: hypothetical protein  FIG01211797: hypothetical protein  FIG01211910: hypothetical protein  FIG032621: Hydrolase2C alpha/beta hydrolase fold family  Ferric siderophore transport system2C periplasmic binding protein TonB  Glutathione S-transferase (EC 2.5.1.18)  Porphobilinogen synthase (EC 4.2.1.24)  Transcriptional regulator lysR family  hypothetical protein  protease2C putative  protein |
| 16264 | AGDB01000082.1:1..16264 | ABC-type phosphate/phosphonate transport system periplasmic component  Aerotaxis sensor receptor protein  COG2363  D-alanyl-D-alanine carboxypeptidase (EC 3.4.16.4)  FIG01209965: hypothetical protein  FIG01212003: hypothetical protein  Membrane protease family protein BA0301  Phosphoribosylglycinamide formyltransferase 2 (EC 2.1.2.-)  Roadblock/LC7 family protein  aminoglycoside 3'-phosphotransferase  hypothetical protein  hypothetical protein  hypothetical protein  hypothetical protein  hypothetical protein  hypothetical protein  hypothetical protein  hypothetical protein  hypothetical protein  hypothetical protein  hypothetical protein  ring hydroxylating dioxygenase alpha-subunit  two-component system regulatory protein |
| 16257 | AGDB01000123.1:1..16257 | Glucoamylase (EC 3.2.1.3)  Glucose-6-phosphate 1-dehydrogenase (EC 1.1.1.49)  Glutathione-regulated potassium-efflux system protein KefB  Outer membrane protein  ankyrin-like protein  hypothetical protein  probable two-component response regulator  protease  sensory box histidine kinase/response regulator |
| 16237 | AGDB01000198.1:1..16237 | 52C10-methylenetetrahydrofolate reductase (EC 1.5.1.20)  Adenosylhomocysteinase (EC 3.3.1.1)  Alpha-amylase  Conserved hypothetical protein 2001  FIG01211153: hypothetical protein  FIG01212366: hypothetical protein  FIG01212745: hypothetical protein  Methyl-accepting chemotaxis protein I (serine chemoreceptor protein)  cytochrome P450 hydroxylase  hypothetical protein  hypothetical protein  hypothetical protein  hypothetical protein  hypothetical protein  methyltransferase  sulfotransferase |
| 16046 | AGDB01000060.1:1..16046 | Alcohol dehydrogenase (EC 1.1.1.1)  Carboxyl-terminal protease (EC 3.4.21.102)  D-3-phosphoglycerate dehydrogenase (EC 1.1.1.95)  FIG01209735: hypothetical protein  FIG01211097: hypothetical protein  FIG01212518: hypothetical protein  INTEGRAL MEMBRANE PROTEIN (Rhomboid family)  Long-chain fatty acid transport protein  Membrane-bound metallopeptidase  Proline dehydrogenase (EC 1.5.99.8) (Proline oxidase) / Delta-1-pyrroline-5-carboxylate dehydrogenase (EC 1.5.1.12)  hypothetical protein  peptidase M28 |
| 15946 | AGDB01000024.1:1..15946 | Chromosomal replication initiator protein DnaA  DNA gyrase subunit B (EC 5.99.1.3)  DNA polymerase III beta subunit (EC 2.7.7.7)  DNA recombination and repair protein RecF  FIG01210301: hypothetical protein  Ferric siderophore transport system2C periplasmic binding protein TonB  Inner membrane protein translocase component YidC2C long form  LSU ribosomal protein L34p  Ribonuclease P protein component (EC 3.1.26.5)  TPR domain protein2C putative component of TonB system  Zn-dependent protease with chaperone function PA4632 |
| 15942 | AGDB01000151.1:1..15942 | FIG01122584: hypothetical protein  FIG01210269: hypothetical protein  Ferredoxin2C 2Fe-2S  Fumarylacetoacetate hydrolase family protein  Large-conductance mechanosensitive channel  Phospholipase C  Phospholipase/lecithinase/hemolysin  Sulfur carrier protein ThiS  Thiazole biosynthesis protein ThiG  aminopeptidase  hypothetical tRNA/rRNA methyltransferase yfiF [EC:2.1.1.-]  sulfur deprivation response regulator  tRNA (guanine46-N7-)-methyltransferase (EC 2.1.1.33)  tRNA-Gly-CCC  transcriptional regulator lacI family |
| 15862 | AGDB01000391.1:1..15862 | 1-acyl-sn-glycerol-3-phosphate acyltransferase (EC 2.3.1.51)  3-dehydroquinate dehydratase II (EC 4.2.1.10)  Biotin carboxyl carrier protein of acetyl-CoA carboxylase  Biotin carboxylase of acetyl-CoA carboxylase (EC 6.3.4.14)  CDP-diacylglycerol-glycerol-3-phosphate 3-phosphatidyltransferase-related protein  Cytochrome c-type biogenesis protein ResA  DNA-binding protein Fis  Hyaluronidase  Hydroxyacylglutathione hydrolase (EC 3.1.2.6)  Phosphatidate cytidylyltransferase (EC 2.7.7.41)  Ribosomal protein L11 methyltransferase (EC 2.1.1.-)  Transcriptional regulator pbsX family  hypothetical protein  hypothetical protein  hypothetical protein  hypothetical protein  hypothetical protein |
| 15807 | AGDB01000370.1:1..15807 | 3-dehydroquinate dehydratase  Autolysin sensor kinase (EC 2.7.3.-)  Endoglucanase (EC 3.2.1.4)  FIG01210787: hypothetical protein  TonB-dependent siderophore receptor  Transcriptional regulator protein  amino acid transporter  aminopeptidase  hypothetical protein  hypothetical protein  hypothetical protein  hypothetical protein  hypothetical protein  putative secreted protein |
| 15724 | AGDB01000246.1:1..15724 | 3-deoxy-manno-octulosonate cytidylyltransferase (EC 2.7.7.38)  Alr1013 protein  CDP-diacylglycerol--glycerol-3-phosphate 3-phosphatidyltransferase (EC 2.7.8.5)  Excinuclease ABC subunit C  Ferrichrome-iron receptor  Low molecular weight protein tyrosine phosphatase (EC 3.1.3.48)  Outer membrane receptor for ferric coprogen and ferric-rhodotorulic acid  hypothetical protein  hypothetical protein  hypothetical protein  hypothetical protein  hypothetical protein  hypothetical protein  hypothetical protein  putative secreted protein  tRNA-Cys-GCA  tRNA-Gly-GCC |
| 15642 | AGDB01000080.1:1..15642 | 3-deoxy-D-manno-octulosonic acid kinase (EC 2.7.1.-)  ADP-heptose--lipooligosaccharide heptosyltransferase II (EC 2.4.1.-)  DNA polymerase III epsilon subunit (EC 2.7.7.7)  FIG005121: SAM-dependent methyltransferase (EC 2.1.1.-)  FIG01209666: hypothetical protein  FIG01209934: hypothetical protein  FIG01210053: hypothetical protein  Hydroxyacylglutathione hydrolase (EC 3.1.2.6)  Molybdenum cofactor biosynthesis protein MoaA  Molybdenum cofactor biosynthesis protein MoaC  Molybdenum cofactor biosynthesis protein MoaD  Molybdenum cofactor biosynthesis protein MoaE  Ribonuclease HI (EC 3.1.26.4)  hypothetical protein  protein phosphatase  putative rhamnogalacturonase B precursor  tRNA-Ser-CGA  tRNA-Ser-GGA |
| 15574 | AGDB01000077.1:1..15574 | Acetyltransferase2C GNAT family (EC 2.3.1.-)  FIG01210523: hypothetical protein  FIG01210868: hypothetical protein  Fibronectin type III domain protein  Lipoprotein  Microcystin dependent protein  Microcystin dependent protein  Microcystin dependent protein  Uncharacterized ABC transporter2C ATP-binding protein YrbF  Uncharacterized ABC transporter2C auxiliary component YrbC  Uncharacterized ABC transporter2C periplasmic component YrbD  Uncharacterized ABC transporter2C permease component YrbE  hypothetical protein  hypothetical protein |
| 15496 | AGDB01000374.1:1..15496 | FIG01209691: hypothetical protein  Penicillin-binding protein 2 (PBP-2)  Rod shape-determining protein MreB  Rod shape-determining protein MreC  Rod shape-determining protein MreD  Rod shape-determining protein RodA  S-(hydroxymethyl)glutathione dehydrogenase (EC 1.1.1.284)  Sugar kinase  TonB-dependent receptor  surface antigen gene  transcriptional regulator |
| 15434 | AGDB01000229.1:1..15434 | Alpha-2-macroglobulin  Glycerophosphoryl diester phosphodiesterase (EC 3.1.4.46)  Hydrolase2C haloacid dehalogenase-like family  Inosine-uridine preferring nucleoside hydrolase (EC 3.2.2.1)  PrpF protein involved in 2-methylcitrate cycle  TonB-dependent receptor  TonB-dependent receptor  hypothetical protein  hypothetical protein  hypothetical protein |
| 15296 | AGDB01000096.1:1..15296 | COGs COG3737  Cell division protein FtsH (EC 3.4.24.-)  Cell division protein FtsJ / Ribosomal RNA large subunit methyltransferase E (EC 2.1.1.-)  Dihydropteroate synthase (EC 2.5.1.15)  FIG004454: RNA binding protein  GTP-binding protein HflX  Protein Implicated in DNA repair function with RecA and MutS  RNA-binding protein Hfq  RecA protein  Regulatory protein RecX  SOS-response repressor and protease LexA (EC 3.4.21.88)  Ubiquinone biosynthesis monooxygenase UbiB  hypothetical protein  tRNA delta(2)-isopentenylpyrophosphate transferase (EC 2.5.1.8) |
| 15167 | AGDB01000280.1:1..15167 | Acetoacetyl-CoA reductase (EC 1.1.1.36)  DNA mismatch repair protein MutL  Esterase/lipase  FIG01209920: hypothetical protein  FIG01210620: hypothetical protein  FIG01211838: hypothetical protein  FOG: PAS/PAC domain  Glutathione S-transferase (EC 2.5.1.18)  PhbF  Probable protease htpX homolog (EC 3.4.24.-)  Selenoprotein O and cysteine-containing homologs  YaeQ protein  glutamyl-Q-tRNA synthetase  major cold shock protein  transcriptional regulator2C TetR family |
| 15129 | AGDB01000143.1:1..15129 | ABC-type anion transport system2C duplicated permease component  ABC-type nitrate/sulfonate/bicarbonate transport system2C ATPase component  C-di-GMP phosphodiesterase A  Chemotaxis protein CheV (EC 2.7.3.-)  FIG01210950: hypothetical protein  Flagellar basal-body P-ring formation protein FlgA  Histidine kinase  HrpX related protein  Negative regulator of flagellin synthesis FlgM  Sensory box/GGDEF family protein  hypothetical protein |
| 14994 | AGDB01000418.1:1..14994 | 3-deoxy-D-manno-octulosonate 8-phosphate phosphatase (EC 3.1.3.45)  Arabinose 5-phosphate isomerase (EC 5.3.1.13)  FIG000506: Predicted P-loop-containing kinase  FIG01211114: hypothetical protein  FIG01211150: hypothetical protein  FIG01211650: hypothetical protein  HPr kinase/phosphorylase (EC 2.7.1.-) (EC 2.7.4.-)  Lipopolysaccharide ABC transporter2C ATP-binding protein LptB  LptA2C protein essential for LPS transport across the periplasm  PTS system nitrogen-specific IIA component2C PtsN  PTS system2C mannose-specific IIA component (EC 2.7.1.69)  Phosphocarrier protein2C nitrogen regulation associated  Phosphoenolpyruvate-protein phosphotransferase of PTS system (EC 2.7.3.9)  RNA polymerase sigma-54 factor RpoN  Ribosome hibernation protein YhbH  UDP-N-acetylglucosamine 1-carboxyvinyltransferase (EC 2.5.1.7)  Uncharacterized protein YrbK clustered with lipopolysaccharide transporters  YrbA protein  hypothetical protein |
| 14778 | AGDB01000413.1:1..14778 | 22C32C42C5-tetrahydropyridine-22C6-dicarboxylate N-succinyltransferase (EC 2.3.1.117)  Asparagine synthetase [glutamine-hydrolyzing] (EC 6.3.5.4)  FIG138056: a glutathione-dependent thiol reductase  GAF domain/sensory box/EAL domain protein  Histone acetyltransferase HPA2 and related acetyltransferases  Methionine aminopeptidase (EC 3.4.11.18)  N-succinyl-L2CL-diaminopimelate desuccinylase (EC 3.5.1.18)  Sigma-fimbriae chaperone protein  Sigma-fimbriae tip adhesin  Threonine efflux protein  [Protein-PII] uridylyltransferase (EC 2.7.7.59)  hypothetical protein |
| 14757 | AGDB01000301.1:1..14757 | D-alanyl-D-alanine carboxypeptidase (EC 3.4.16.4)  FIG01209954: hypothetical protein  Lipoate synthase  Nicotinamide phosphoribosyltransferase (EC 2.4.2.12)  Nicotinamide-nucleotide adenylyltransferase2C NadM family (EC 2.7.7.1) / ADP-ribose pyrophosphatase (EC 3.6.1.13)  Octanoate-[acyl-carrier-protein]-protein-N-octanoyltransferase  Proposed lipoate regulatory protein YbeD  Transcriptional regulator2C LysR family  arabinogalactan endo-12C4-beta-galactosidase  membrane protein2C putative  probable outer membrane protein  tail-specific protease |
| 14731 | AGDB01000142.1:1..14731 | ATP-dependent Clp protease ATP-binding subunit ClpA  ATP-dependent Clp protease adaptor protein ClpS  COGs COG3146  Cell division protein FtsK  FIG002903: a protein of unknown function perhaps involved in purine metabolism  Leucyl/phenylalanyl-tRNA--protein transferase (EC 2.3.2.6)  Nudix-like NDP and NTP phosphohydrolase YmfB  Thioredoxin reductase (EC 1.8.1.9)  Translation initiation factor 1  conserved hypothetical protein  hypothetical protein  methyl-accepting chemotaxis sensory transducer  tRNA (5-methylaminomethyl-2-thiouridylate)-methyltransferase (EC 2.1.1.61) |
| 14653 | AGDB01000468.1:1..14653 | FOG: Ankyrin repeat  FrmR: Negative transcriptional regulator of formaldehyde detoxification operon  Glutamate--cysteine ligase (EC 6.3.2.2)  Glutathione-dependent formaldehyde-activating enzyme (EC 4.4.1.22)  PlcB2C ORFX2C ORFP2C ORFB2C ORFA2C ldh gene  S-(hydroxymethyl)glutathione dehydrogenase (EC 1.1.1.284)  S-formylglutathione hydrolase (EC 3.1.2.12)  Transcriptional regulator2C GntR family domain / Aspartate aminotransferase (EC 2.6.1.1)  Zn-dependent hydrolases2C including glyoxylases  hypothetical protein  hypothetical protein  hypothetical protein  nodulin 21-related protein  transcriptional regulator  two-component system regulatory protein  two-component system sensor protein |
| 14604 | AGDB01000025.1:1..14604 | Abortive infection protein  Dehydrogenases with different specificities (related to short-chain alcohol dehydrogenases)  Phosphohydrolase (MutT/nudix family protein)  Ribonucleotide reductase of class II (coenzyme B12-dependent) (EC 1.17.4.1)  Ribonucleotide reductase of class II (coenzyme B12-dependent)2C alpha subunit (EC 1.17.4.1)  Transcriptional regulator2C LysR family  Trypsin-like serine proteases2C typically periplasmic2C contain C-terminal PDZ domain  diguanylate phosphodiesterase with GAF sensor  histone protein  hypothetical protein |
| 14576 | AGDB01000085.1:1..14576 | Chemotaxis protein methyltransferase CheR (EC 2.1.1.80)  Chemotaxis response regulator protein-glutamate methylesterase CheB (EC 3.1.1.61)  Methyl-accepting chemotaxis protein I (serine chemoreceptor protein)  Methyl-accepting chemotaxis protein I (serine chemoreceptor protein)  Methyl-accepting chemotaxis protein I (serine chemoreceptor protein)  Positive regulator of CheA protein activity (CheW)  Putative glycosyl hydrolase of unknown function (DUF1680)  Putative permease  Putative regulatory protein  diguanylate cyclase/phosphodiesterase (GGDEF |
| 14517 | AGDB01000399.1:1..14517 | Beta-galactosidase (EC 3.2.1.23)  Maltodextrin glucosidase (EC 3.2.1.20)  TonB-dependent receptor  Xylulose kinase (EC 2.7.1.17)  galactose-binding protein regulator  hypothetical protein  sialic acid-specific 9-O-acetylesterase |
| 14462 | AGDB01000093.1:1..14462 | Acyl-CoA thioester hydrolase  Alkaline phosphatase (EC 3.1.3.1)  Cell wall-associated hydrolases (invasion-associated proteins)  FIG01111849: hypothetical protein  FIG01210044: hypothetical protein  FKBP-type peptidyl-prolyl cis-trans isomerase SlyD (EC 5.2.1.8)  Glutathione reductase (EC 1.8.1.7)  NLP/P60 family protein  Permease of the drug/metabolite transporter (DMT) superfamily  Proton/glutamate symport protein @ Sodium/glutamate symport protein  Transport protein  hypothetical protein  methylated-DNA-protein-cysteine S-methyltransferase related protein  putative nucleotidyltransferase |
| 14377 | AGDB01000054.1:1..14377 | Benzoate transport protein  FIG007317: hypothetical protein  FIG011065: hypothetical protein  FIG01211439: hypothetical protein  FIG039767: hypothetical protein  Membrane-bound lytic murein transglycosylase B precursor (EC 3.2.1.-)  Phytochrome2C two-component sensor histidine kinase (EC 2.7.3.-)  UPF0225 protein YchJ  hypothetical protein  tetracycline-efflux transporter |
| 14318 | AGDB01000277.1:1..14318 | ATP/GTP-binding protein  CDP-diacylglycerol--serine O-phosphatidyltransferase (EC 2.7.8.8)  D-beta-hydroxybutyrate dehydrogenase (EC 1.1.1.30)  FIG01210457: hypothetical protein  FIG01213735: hypothetical protein  Mutator mutT protein (72C8-dihydro-8-oxoguanine-triphosphatase) (EC 3.6.1.-)  Oxidoreductase (EC 1.1.1.-)  PHA synthase subunit  Polyhydroxyalkanoic acid synthase  Putative stress-responsive transcriptional regulator  hypothetical protein  hypothetical protein  sensor histidine kinase |
| 14174 | AGDB01000465.1:1..14174 | Acetoacetyl-CoA reductase (EC 1.1.1.36)  Phosphate-specific outer membrane porin OprP ; Pyrophosphate-specific outer membrane porin OprO  Predicted sucrose-specific TonB-dependent receptor  Sugar transporter  Tricarboxylate transport transcriptional regulator TctD  Two-component system sensor protein  Uncharacterized transporter2C similarity to citrate transporter  alpha-amylase (EC 3.2.1.1)  hypothetical protein  transcriptional regulator2C LacI family |
| 14164 | AGDB01000409.1:1..14164 | Aldehyde dehydrogenase (EC 1.2.1.3)  Membrane fusion component of tripartite multidrug resistance system  Nucleoside-diphosphate-sugar epimerases  Outer membrane component of tripartite multidrug resistance system  Topoisomerase IV subunit A (EC 5.99.1.-)  Transcriptional regulator2C AraC family  Transcriptional regulator2C MarR family  Transmembrane protein  hypothetical protein  hypothetical protein  hypothetical protein  hypothetical protein  probable DNA-binding protein  transcriptional regulator2C TetR family |
| 14061 | AGDB01000048.1:1..14061 | 32C4-dihydroxy-2-butanone 4-phosphate synthase / GTP cyclohydrolase II (EC 3.5.4.25)  Alpha-12C2-mannosidase  D-mannose isomerase (EC 5.3.1.7)  D-tyrosyl-tRNA(Tyr) deacylase  Lipid A biosynthesis lauroyl acyltransferase (EC 2.3.1.-)  Phosphinothricin N-acetyltransferase (EC 2.3.1.-)  Predicted mannose transporter2C GGP family  RNA polymerase sigma factor RpoD  hypothetical protein  transcriptional regulator |
| 13727 | AGDB01000173.1:1..13727 | DNA-binding response regulator KdpE  Osmosensitive K channel histidine kinase KdpD (EC 2.7.3.-)  Potassium-transporting ATPase A chain (EC 3.6.3.12) (TC 3.A.3.7.1)  Potassium-transporting ATPase B chain (EC 3.6.3.12) (TC 3.A.3.7.1)  Potassium-transporting ATPase C chain (EC 3.6.3.12) (TC 3.A.3.7.1)  Protein of unknown function DUF1597  Thiamine-monophosphate kinase (EC 2.7.4.16)  hypothetical protein  putative ABC transporter ATP-binding protein |
| 13576 | AGDB01000164.1:1..13576 | 3-dehydroquinate dehydratase I (EC 4.2.1.10)  4-hydroxybenzoate transporter  FIG01209899: hypothetical protein  Quinate/shikimate dehydrogenase [Pyrroloquinoline-quinone] (EC 1.1.99.25)  RND efflux system2C inner membrane transporter CmeB  RND efflux system2C membrane fusion protein CmeA  RND efflux system2C outer membrane lipoprotein CmeC  hypothetical protein  lipase |
| 13507 | AGDB01000350.1:1..13507 | 2-keto-3-deoxygluconate permease (KDG permease)  ABC transporter2C ATP-binding/permease protein  Beta-lactamase (EC 3.5.2.6)  COG1801: Uncharacterized conserved protein  Permease of the drug/metabolite transporter (DMT) superfamily  Preprotein translocase subunit YajC (TC 3.A.5.1.1)  Protein-export membrane protein SecD (TC 3.A.5.1.1)  Protein-export membrane protein SecF (TC 3.A.5.1.1)  hypothetical protein  sensor histidine kinase  tRNA-guanine transglycosylase (EC 2.4.2.29) |
| 13475 | AGDB01000149.1:1..13475 | Acetyltransferase  Glycosyltransferase  Membrane alanine aminopeptidase N (EC 3.4.11.2)  Membrane fusion protein of RND family multidrug efflux pump  Polysaccharide deacetylase  RND multidrug efflux transporter; Acriflavin resistance protein  Sulfate adenylyltransferase subunit 1 (EC 2.7.7.4) / Adenylylsulfate kinase (EC 2.7.1.25)  Sulfate adenylyltransferase subunit 2 (EC 2.7.7.4)  hypothetical protein  hypothetical protein |
| 13391 | AGDB01000358.1:1..13391 | General secretion pathway protein E / Type II secretion cytoplasmic ATP binding protein (PulE2C ATPase)  General secretion pathway protein F  General secretion pathway protein G  General secretion pathway protein H  General secretion pathway protein I  General secretion pathway protein J  General secretion pathway protein K  Outer membrane protein  Protease  hypothetical protein  hypothetical protein |
| 13363 | AGDB01000332.1:1..13363 | Acetolactate synthase large subunit (EC 2.2.1.6)  Acetolactate synthase small subunit (EC 2.2.1.6)2C Xanthomonadales type  FIG01111142: hypothetical protein  Gamma-glutamyltranspeptidase (EC 2.3.2.2)  Ketol-acid reductoisomerase (EC 1.1.1.86)  Thiamin biosynthesis protein ThiC  chemotaxis protein  hypothetical protein  hypothetical protein |
| 13321 | AGDB01000104.1:1..13321 | 2-amino-3-ketobutyrate coenzyme A ligase (EC 2.3.1.29)  FIG01209976: hypothetical protein  FIG01211355: hypothetical protein  Hypothetical2C related to broad specificity phosphatases COG0406  L-threonine 3-dehydrogenase (EC 1.1.1.103)  Pseudouridylate synthase  Sulfate and thiosulfate binding protein CysP  Sulfate and thiosulfate import ATP-binding protein CysA (EC 3.6.3.25)  Sulfate transport system permease protein CysT  Sulfate transport system permease protein CysW  hypothetical protein |
| 13124 | AGDB01000335.1:1..13124 | 1-pyrroline-4-hydroxy-2-carboxylate deaminase (EC 3.5.4.22)  Amino acid permease in 4-hydroxyproline catabolic gene cluster  Dipeptidyl peptidase IV in 4-hydroxyproline catabolic gene cluster  FIG01211086: hypothetical protein  FIG01217180: hypothetical protein  Ketoglutarate semialdehyde dehydrogenase (EC 1.2.1.26)  Predicted 4-hydroxyproline dipeptidase  Putative oxidoreductase in 4-hydroxyproline catabolic gene cluster  hypothetical protein  hypothetical protein |
| 13063 | AGDB01000460.1:1..13063 | FIG01211048: hypothetical protein  Long-chain-fatty-acid--CoA ligase (EC 6.2.1.3)  Oxidoreductase2C short-chain dehydrogenase/reductase family  Protein-L-isoaspartate O-methyltransferase (EC 2.1.1.77)  Sensory histidine kinase QseC  Transcriptional regulator2C TetR family  Two-component system response regulator QseB  Type I secretion outer membrane protein2C TolC precursor  hypothetical protein  hypothetical protein  long-chain acyl-CoA synthetase  putative thermostable hemolysin |
| 13014 | AGDB01000396.1:1..13014 | Aspartate 1-decarboxylase (EC 4.1.1.11)  Cytochrome c551 peroxidase (EC 1.11.1.5)  Glucose-6-phosphate isomerase (EC 5.3.1.9)  IPT/TIG domain protein2C putative  hypothetical protein  hypothetical protein |
| 12926 | AGDB01000286.1:1..12926 | DNA recombination protein RmuC  FIG01211801: hypothetical protein  Glutathione peroxidase (EC 1.11.1.9)  Glycerate kinase (EC 2.7.1.31)  LSU m5C1962 methyltransferase RlmI  Mg(2 ) transport ATPase protein C  Nitrogen regulation protein NR(I)  amino acid transporter  export protein  hypothetical protein  hypothetical protein  hypothetical protein  periplasmic sensor signal transduction histidine kinase |
| 12865 | AGDB01000041.1:1..12865 | Alcohol dehydrogenase (EC 1.1.1.1)  Aldehyde dehydrogenase (EC 1.2.1.3)  FIG01210906: hypothetical protein  Ku domain protein  L-lactate dehydrogenase (EC 1.1.2.3)  Lactate-responsive regulator LldR in Enterobacteria2C GntR family  Putative protein-S-isoprenylcysteine methyltransferase  Two-component system regulatory protein  hypothetical protein  hypothetical protein  hypothetical protein  putative inner membrane protein  putative sigma-54-dependent transcriptional regulator  two-component system sensor protein |
| 12742 | AGDB01000051.1:1..12742 | ABC transporter2C ATP-binding protein  ATP-dependent DNA helicase Rep  FIG01210778: hypothetical protein  Fatty acid desaturase (EC 1.14.19.1); Delta-9 fatty acid desaturase (EC 1.14.19.1)  Formamidopyrimidine-DNA glycosylase (EC 3.2.2.23)  GTPase and tRNA-U34 5-formylation enzyme TrmE  Glucans biosynthesis protein D precursor  NADPH:quinone oxidoreductase 2  Polysaccharide deacetylase2C caspase activity  Redox-sensing transcriptional regulator QorR2C putative  Thymidine kinase (EC 2.7.1.21)  hypothetical protein  hypothetical protein  hypothetical protein |
| 12593 | AGDB01000445.1:1..12593 | Alpha2Calpha-trehalose-phosphate synthase [UDP-forming] (EC 2.4.1.15)  GCN5-related N-acetyltransferase  Glucoamylase (EC 3.2.1.3)  Glucose dehydrogenase2C PQQ-dependent (EC 1.1.5.2)  Trehalose-6-phosphate phosphatase (EC 3.1.3.12)  ferric enterobactin receptor  hypothetical protein  hypothetical protein |
| 12579 | AGDB01000007.1:1..12579 | Cell division protein FtsA  Cell division protein FtsQ  Cell division protein FtsZ (EC 3.4.24.-)  D-alanine--D-alanine ligase (EC 6.3.2.4)  Protein export cytoplasm protein SecA ATPase RNA helicase (TC 3.A.5.1.1)  UDP-3-O-[3-hydroxymyristoyl] N-acetylglucosamine deacetylase (EC 3.5.1.-)  UDP-N-acetylglucosamine--N-acetylmuramyl-(pentapeptide) pyrophosphoryl-undecaprenol N-acetylglucosamine transferase (EC 2.4.1.227)  UDP-N-acetylmuramate--alanine ligase (EC 6.3.2.8)  peptidase |
| 12442 | AGDB01000429.1:1..12442 | Polyribonucleotide nucleotidyltransferase (EC 2.7.7.8)  Ribosome-binding factor A  SSU ribosomal protein S15p (S13e)  Translation initiation factor 2  diguanylate cyclase with GAF sensor  hypothetical protein  hypothetical protein  tRNA pseudouridine synthase B (EC 4.2.1.70) |
| 12412 | AGDB01000393.1:1..12412 | Flagellar biosynthesis protein FlhA  Flagellar biosynthesis protein FlhB  Flagellar biosynthesis protein FliL  Flagellar biosynthesis protein FliP  Flagellar biosynthesis protein FliQ  Flagellar biosynthesis protein FliQ  Flagellar biosynthesis protein FliR  Flagellar hook-length control protein FliK  Flagellar motor switch protein FliM  Flagellar motor switch protein FliN  diguanylate cyclase (GGDEF domain)  hypothetical protein |
| 12361 | AGDB01000020.1:1..12361 | Hemolysin  Large exoproteins involved in heme utilization or adhesion  Putative large exoprotein involved in heme utilization or adhesion of ShlA/HecA/FhaA family  Putative large exoprotein involved in heme utilization or adhesion of ShlA/HecA/FhaA family  probable hemagglutinin |
| 12333 | AGDB01000108.1:1..12333 | 5-methyltetrahydrofolate--homocysteine methyltransferase (EC 2.1.1.13)  5-methyltetrahydrofolate--homocysteine methyltransferase (EC 2.1.1.13)  Butyryl-CoA dehydrogenase (EC 1.3.99.2)  FIG01212492: hypothetical protein  RND efflux system2C inner membrane transporter CmeB  Transcriptional regulator2C ArsR family / Methyltransferase fusion  UDP-glucose 4-epimerase (EC 5.1.3.2) |
| 12298 | AGDB01000181.1:1..12298 | AmpG permease  Anhydro-N-acetylmuramic acid kinase (EC 2.7.1.-)  Arginyl-tRNA synthetase (EC 6.1.1.19)  DNA repair protein RadC  Deoxyuridine 5'-triphosphate nucleotidohydrolase (EC 3.6.1.23)  Exodeoxyribonuclease III (EC 3.1.11.2)  Exonuclease SbcC  Orotate phosphoribosyltransferase (EC 2.4.2.10)  Phosphomannomutase (EC 5.4.2.8) / Phosphoglucomutase (EC 5.4.2.2)  Phosphopantothenoylcysteine decarboxylase (EC 4.1.1.36) / Phosphopantothenoylcysteine synthetase (EC 6.3.2.5)  hypothetical protein |
| 12230 | AGDB01000012.1:1..12230 | ATP-dependent RNA helicase DbpA  Adenine phosphoribosyltransferase (EC 2.4.2.7)  COG0488: ATPase components of ABC transporters with duplicated ATPase domains  FIG01211504: hypothetical protein  Manganese superoxide dismutase (EC 1.15.1.1)  RND efflux system2C inner membrane transporter CmeB  RND efflux system2C membrane fusion protein CmeA  bioH protein2C putative  hypothetical protein  hypothetical protein  ribonuclease |
| 11938 | AGDB01000356.1:1..11938 | Cytosol aminopeptidase PepA (EC 3.4.11.1)  DNA polymerase III chi subunit (EC 2.7.7.7)  Distant similarity with leukotriene C4 synthase (microsomal glutathione S-transferase)  FIG000906: Predicted Permease  FIG000988: Predicted permease  FIG01212138: hypothetical protein  Pectate lyase precursor (EC 4.2.2.2)  Ribosomal-protein-S18p-alanine acetyltransferase (EC 2.3.1.-)  Valyl-tRNA synthetase (EC 6.1.1.9) |
| 11732 | AGDB01000360.1:1..11732 | Ferrichrome-iron receptor  General secretion pathway protein D / Type II secretion outermembrane pore forming protein (PulD)  General secretion pathway protein N  Glycosyl transferase  Glycosyltransferase (EC 2.4.1.-)  PnuC protein  Putative homoserine kinase type II2C PnuC-associated2C THI-regulated branch  Transcriptional regulator2C GntR family |
| 11732 | AGDB01000265.1:1..11732 | ATPase YjeE2C predicted to have essential role in cell wall biosynthesis  Epoxyqueuosine (oQ) reductase QueG  Exodeoxyribonuclease VII large subunit (EC 3.1.11.6)  FIG01210123: hypothetical protein  Outer membrane lipoprotein Blc  YjeF protein2C function unknown  hypothetical protein  hypothetical protein  hypothetical protein  hypothetical protein  secretion protein HlyD family  tRNA-Phe-GAA  transcriptional regulator marR family |
| 11615 | AGDB01000019.1:1..11615 | Acetyltransferase  Outer membrane protein  Protein of unknown function YceH  hypothetical protein  hypothetical protein  putative exported protein |
| 11571 | AGDB01000035.1:1..11571 | FIG01210576: hypothetical protein  TonB-dependent receptor  hypothetical protein |
| 11503 | AGDB01000457.1:1..11503 | Beta-xylosidase (EC 3.2.1.37)  Exodeoxyribonuclease VII small subunit (EC 3.1.11.6)  FIG01210697: hypothetical protein  FIG138315: Putative alpha helix protein  Octaprenyl-diphosphate synthase (EC 2.5.1.-) / Dimethylallyltransferase (EC 2.5.1.1) / Geranyltranstransferase (farnesyldiphosphate synthase) (EC 2.5.1.10) / Geranylgeranyl pyrophosphate synthetase (EC 2.5.1.29)  TldD protein2C part of proposed TldE/TldD proteolytic complex (PMID 12029038)  TldE/PmbA protein2C part of proposed TldE/TldD proteolytic complex (PMID 12029038)  conserved hypothetical protein  extracellular protease( EC:3.4.24.20 )  hypothetical protein  hypothetical protein  tRNA(Ile)-lysidine synthetase |
| 11473 | AGDB01000323.1:1..11473 | FIG01210399: hypothetical protein  Methyl-accepting chemotaxis protein I (serine chemoreceptor protein)  Methyl-accepting chemotaxis protein I (serine chemoreceptor protein)  Methyl-accepting chemotaxis protein I (serine chemoreceptor protein)  Positive regulator of CheA protein activity (CheW)  hypothetical protein  hypothetical protein  hypothetical protein  hypothetical protein |
| 11453 | AGDB01000403.1:1..11453 | 3-oxoacyl-[acyl-carrier protein] reductase (EC 1.1.1.100)  FIG01111127: hypothetical protein  Glycogen debranching enzyme (EC 3.2.1.-)  Pirin  Rhodanese-related sulfurtransferase  Transcriptional regulator  hypothetical protein  hypothetical protein  putative; ORF located using Glimmer/Genemark  tRNA-Arg-ACG  tRNA-Arg-ACG |
| 11442 | AGDB01000144.1:1..11442 | Flagellar L-ring protein FlgH  Flagellar P-ring protein FlgI  Flagellar basal-body rod modification protein FlgD  Flagellar basal-body rod protein FlgB  Flagellar basal-body rod protein FlgC  Flagellar basal-body rod protein FlgF  Flagellar basal-body rod protein FlgG  Flagellar hook protein FlgE  Flagellar hook-associated protein FlgK  Flagellar hook-associated protein FlgL  Flagellar protein FlgJ [peptidoglycan hydrolase] (EC 3.2.1.-) |
| 11326 | AGDB01000310.1:1..11326 | ATP-dependent RNA helicase RhlB  Cell division protein FtsX  Cell division transporter2C ATP-binding protein FtsE (TC 3.A.5.1.1)  FIG01211391: hypothetical protein  Helicase PriA essential for oriC/DnaA-independent DNA replication  NADH dehydrogenase (EC 1.6.99.3)  RNA polymerase sigma factor RpoH  Response regulator protein  Uracil-DNA glycosylase2C family 1  Vancomycin B-type resistance protein VanW |
| 11267 | AGDB01000071.1:1..11267 | Acetyltransferase2C GNAT family  FIG01211014: hypothetical protein  FKBP-type peptidyl-prolyl cis-trans isomerase  Ferredoxin-dependent glutamate synthase (EC 1.4.7.1)  NAD(FAD)-utilizing dehydrogenases  Oxidoreductase2C short chain dehydrogenase/reductase family  Rhodanese domain protein UPF01762C Betaproteobacterial subgroup  beta-lactamase  polysaccharide biosynthetic protein  sensory box histidine kinase/response regulator |
| 11196 | AGDB01000063.1:1..11196 | Dihydroxyacetone kinase2C ATP-dependent (EC 2.7.1.29)  FIG00960671: hypothetical protein  Membrane protein2C putative  Potassium efflux system KefA protein / Small-conductance mechanosensitive channel  Putative diheme cytochrome c-553  Ubiquinone biosynthesis monooxygenase UbiB  hypothetical protein  hypothetical protein |
| 11180 | AGDB01000132.1:1..11180 | Cell division protein FtsH (EC 3.4.24.-)  DNA-3-methyladenine glycosylase II (EC 3.2.2.21)  FIG01111551: hypothetical protein  Gluconokinase (EC 2.7.1.12)  wall-associated protein |
| 11109 | AGDB01000357.1:1..11109 | FIG01210521: hypothetical protein  LigA  PE\_PGRS family protein  Phosphoribosylformylglycinamidine synthase2C synthetase subunit (EC 6.3.5.3) / Phosphoribosylformylglycinamidine synthase2C glutamine amidotransferase subunit (EC 6.3.5.3)  Thiol:disulfide interchange protein DsbC  Tyrosine recombinase XerD  hypothetical protein |
| 11012 | AGDB01000365.1:1..11012 | Flavodoxin reductases (ferredoxin-NADPH reductases) family 1  Nitrogen regulation protein NR(I)  Oxidoreductase  POSSIBLE LINOLEOYL-CoA DESATURASE (DELTA(6)-DESATURASE)  Pyruvate oxidase [ubiquinone2C cytochrome] (EC 1.2.2.2)  Unsaturated fatty acid biosythesis repressor FabR2C TetR family  hypothetical protein  hypothetical protein  protein of unknown function DUF1452  two-component system sensor protein |
| 10688 | AGDB01000032.1:1..10688 | Catalase (EC 1.11.1.6) / Peroxidase (EC 1.11.1.7)  Glutathione S-transferase (EC 2.5.1.18)  Ribosomal protein S6 glutaminyl transferase  TonB-dependent receptor  Transcriptional regulator2C DeoR family  Two-component system regulatory protein  conserved hypothetical protein  hypothetical protein  hypothetical protein  two-component system sensor protein |
| 10625 | AGDB01000443.1:1..10625 | 3-oxoacyl-[acyl-carrier protein] reductase (EC 1.1.1.100)  ClpB protein  Fusaric acid resistance protein conserved region  Glutathione S-transferase  O-acetylhomoserine sulfhydrylase (EC 2.5.1.49) / O-succinylhomoserine sulfhydrylase (EC 2.5.1.48)  Transcriptional regulator2C AraC family  Transcriptional regulator2C HxlR family  Transcriptional regulator2C LysR family  hypothetical protein |
| 10509 | AGDB01000433.1:1..10509 | ABC transporter2C permease protein2C putative  ABC-type multidrug transport system2C ATPase component  FIG01210246: hypothetical protein  FIG01211096: hypothetical protein  FKBP-type peptidyl-prolyl cis-trans isomerase FkpA precursor (EC 5.2.1.8)  Glutathione peroxidase (EC 1.11.1.9)  Permease of the drug/metabolite transporter (DMT) superfamily  Protein slyX homolog  Transcriptional regulator2C GntR family domain / Aspartate aminotransferase (EC 2.6.1.1)  UDP-glucose dehydrogenase (EC 1.1.1.22)  hypothetical protein  hypothetical protein  transcriptional regulator gntR family |
| 10402 | AGDB01000111.1:1..10402 | 3-oxoacyl-[acyl-carrier protein] reductase (EC 1.1.1.100)  Exodeoxyribonuclease V alpha chain (EC 3.1.11.5)  FIG01209846: hypothetical protein  FIG01210492: hypothetical protein  Lysophospholipase (EC 3.1.1.5)  Transcriptional regulator2C TetR family  hypothetical protein  hypothetical protein  hypothetical protein  hypothetical protein |
| 10361 | AGDB01000056.1:1..10361 | FIG01211164: hypothetical protein  GMP synthase [glutamine-hydrolyzing] (EC 6.3.5.2)  General secretion pathway protein E  Glycyl-tRNA synthetase alpha chain (EC 6.1.1.14)  Glycyl-tRNA synthetase beta chain (EC 6.1.1.14)  Twin-arginine translocation protein TatC  glutamyl endopeptidase  hypothetical protein  hypothetical protein |
| 10241 | AGDB01000122.1:1..10241 | Acetyl-CoA synthetase (ADP-forming) alpha and beta chains2C putative  Cold shock domain family protein  FIG01211261: hypothetical protein  FIG01212194: hypothetical protein  Glyoxalase family protein  Methylcrotonyl-CoA carboxylase biotin-containing subunit (EC 6.4.1.4)  hypothetical protein  hypothetical protein  hypothetical protein  hypothetical protein |
| 10197 | AGDB01000166.1:1..10197 | Acriflavin resistance protein  hypothetical protein  hypothetical protein  hypothetical protein  membrane fusion protein  tRNA-Ala-GGC  tRNA-Ala-GGC  tRNA-Glu-TTC  tRNA-Glu-TTC  tRNA-Leu-CAA  two-component system regulatory protein |
| 10064 | AGDB01000343.1:1..10064 | Histone acetyltransferase HPA2 and related acetyltransferases  Protein involved in catabolism of external DNA  Transcription-repair coupling factor  YwqG  hypothetical protein |
| 10046 | AGDB01000463.1:1..10046 | Aerobic C4-dicarboxylate transporter for fumarate2C L-malate2C D-malate2C succunate2C aspartate  FIG01211600: hypothetical protein  NADP-dependent malic enzyme (EC 1.1.1.40)  Phosphate-specific outer membrane porin OprP ; Pyrophosphate-specific outer membrane porin OprO  Sensor histidine kinase  hypothetical protein  hypothetical protein  hypothetical protein  hypothetical protein |
| 9896 | AGDB01000408.1:1..9896 | 23S rRNA (guanosine-2'-O-) -methyltransferase rlmB (EC 2.1.1.-)  3'-to-5' exoribonuclease RNase R  Beta-glucosidase (EC 3.2.1.21)  Gfa-like protein  VirK family protein  hypothetical protein  hypothetical protein  hypothetical protein  hypothetical protein  tRNA-Leu-CAG  tRNA-Leu-CAG |
| 9688 | AGDB01000059.1:1..9688 | 32C4-dihydroxy-2-butanone 4-phosphate synthase / GTP cyclohydrolase II (EC 3.5.4.25)  62C7-dimethyl-8-ribityllumazine synthase (EC 2.5.1.9)  Diaminohydroxyphosphoribosylaminopyrimidine deaminase (EC 3.5.4.26) / 5-amino-6-(5-phosphoribosylamino)uracil reductase (EC 1.1.1.193)  FIG01209811: hypothetical protein  FIG01209995: hypothetical protein  Riboflavin synthase alpha chain (EC 2.5.1.9)  Ribonucleotide reductase transcriptional regulator NrdR  Serine hydroxymethyltransferase (EC 2.1.2.1)  Transcription termination protein NusB  acetyltransferase  hypothetical protein  hypothetical protein |
| 9630 | AGDB01000455.1:1..9630 | COG3332  Cell division protein FtsL  Cell division protein MraZ  LppC putative lipoprotein  Predicted endonuclease distantly related to archaeal Holliday junction resolvase  Putative membrane-bound metal-dependent hydrolases  rRNA small subunit methyltransferase H  rRNA small subunit methyltransferase I |
| 9601 | AGDB01000446.1:1..9601 | COG1496: Uncharacterized conserved protein  FIG01112524: hypothetical protein  FIG01209970: hypothetical protein  FIG01211029: hypothetical protein  L-proline glycine betaine ABC transport system permease protein ProV (TC 3.A.1.12.1)  L-proline glycine betaine binding ABC transporter protein ProX (TC 3.A.1.12.1) / Osmotic adaptation  Methyl-accepting chemotaxis protein I (serine chemoreceptor protein)  Probable component of the lipoprotein assembly complex (forms a complex with YaeT2C YfgL2C and NlpB)  Ribosomal large subunit pseudouridine synthase D (EC 4.2.1.70)  hypothetical protein |
| 9420 | AGDB01000090.1:1..9420 | 2-ketoglutaric semialdehyde dehydrogenase (EC 1.2.1.26)  Galactose-binding protein regulator  L-arabonate dehydratase (EC 4.2.1.25)  Putative sugar ABC transport system2C periplasmic binding protein YtfQ precursor  RNA polymerase sigma-54 factor RpoN  SUGAR TRANSPORTER  predicted L-arabinose 1-dehydrogenase (EC 1.1.1.46) |
| 9397 | AGDB01000291.1:1..9397 | 2-keto-3-deoxy-D-arabino-heptulosonate-7-phosphate synthase I alpha (EC 2.5.1.54)  TonB-dependent receptor  TonB-dependent receptor  hypothetical protein  related to competence proteins |
| 9395 | AGDB01000316.1:1..9395 | Alanine racemase (EC 5.1.1.1)  D-amino acid dehydrogenase small subunit (EC 1.4.99.1)  FIG01111765: hypothetical protein  L-sorbosone dehydrogenase  Leucine-responsive regulatory protein2C regulator for leucine (or lrp) regulon and high-affinity branched-chain amino acid transport system  Sensor histidine kinase  hypothetical protein  hypothetical protein  hypothetical protein  putative AtsE |
| 9371 | AGDB01000155.1:1..9371 | Adenosylhomocysteinase (EC 3.3.1.1)  Amino acid permease  Amino acid permease  D-2-hydroxyglutarate dehydrogenase  D-3-phosphoglycerate dehydrogenase (EC 1.1.1.95)  FIG01210489: hypothetical protein  hypothetical protein  hypothetical protein |
| 9306 | AGDB01000127.1:1..9306 | Cytochrome c oxidase polypeptide I (EC 1.9.3.1)  Cytochrome c oxidase polypeptide III (EC 1.9.3.1)  Cytochrome oxidase biogenesis protein Cox11-CtaG2C copper delivery to Cox1  Cytochrome oxidase biogenesis protein Surf12C facilitates heme A insertion  FIG01210764: hypothetical protein  FIG01211359: hypothetical protein  FIG01212439: hypothetical protein  Heme A synthase2C cytochrome oxidase biogenesis protein Cox15-CtaA  Heme O synthase2C protoheme IX farnesyltransferase (EC 2.5.1.-) COX10-CtaB  methyl-accepting chemotaxis protein2C putative |
| 9178 | AGDB01000467.1:1..9178 | 2-hydroxychromene-2-carboxylate isomerase  FIG01095481: hypothetical protein  FIG01210796: hypothetical protein  FIG01213217: hypothetical protein  Hypothetical protein DUF1942C DegV family  Phenazine biosynthesis protein PhzF like  hypothetical protein  hypothetical protein  hypothetical protein  hypothetical protein  truncated cellulase S |
| 9047 | AGDB01000064.1:1..9047 | 3-oxoacyl-[acyl-carrier protein] reductase (EC 1.1.1.100)  ATP-dependent RNA helicase RhlE  Dihydrolipoamide acyltransferase component of branched-chain alpha-keto acid dehydrogenase complex (EC 2.3.1.168)  FIG00446866: hypothetical protein  Fructokinase (EC 2.7.1.4)  MgtC/SapB transporter  Oxidoreductase (EC 1.1.1.-)  Vibriolysin2C extracellular zinc protease (EC 3.4.24.25) @ Pseudolysin2C extracellular zinc protease (EC 3.4.24.26)  hypothetical protein |
| 8974 | AGDB01000212.1:1..8974 | Cyclopropane-fatty-acyl-phospholipid synthase-like protein2C clusters with FIG005069  Endoribonuclease L-PSP  FIG001571: Hypothetical protein  FIG005069: Hypothetical protein  S-adenosyl-L-methionine dependent methyltransferase2C similar to cyclopropane-fatty-acyl-phospholipid synthase  hypothetical protein  hypothetical protein  hypothetical protein |
| 8871 | AGDB01000275.1:1..8871 | (Acyl-carrier-protein) phosphodiesterase( EC:3.1.4.14 )  Arsenic resistance protein ArsH  GMP synthase [glutamine-hydrolyzing] (EC 6.3.5.2)  Hypothetical transmembrane protein coupled to NADH-ubiquinone oxidoreductase chain 5 homolog  Inosine-5'-monophosphate dehydrogenase (EC 1.1.1.205)  Methylenetetrahydrofolate dehydrogenase (NADP ) (EC 1.5.1.5) / Methenyltetrahydrofolate cyclohydrolase (EC 3.5.4.9)  Redox-sensing transcriptional regulator QorR2C putative  Transcriptional regulator2C ArsR family  hypothetical protein |
| 8841 | AGDB01000072.1:1..8841 | FIG00537880: hypothetical protein  FIG01211960: hypothetical protein  Glycogen debranching enzyme (EC 3.2.1.-)  MFS transporter  Malto-oligosyltrehalose synthase (EC 5.4.99.15)  Siroheme synthase / Precorrin-2 oxidase (EC 1.3.1.76) / Sirohydrochlorin ferrochelatase (EC 4.99.1.4) / Uroporphyrinogen-III methyltransferase (EC 2.1.1.107)  hypothetical protein  hypothetical protein |
| 8772 | AGDB01000387.1:1..8772 | Beta-glucosidase (EC 3.2.1.21)  Di-/tripeptide transporter  FIG01211502: hypothetical protein  FIG01211598: hypothetical protein  Gll2474 protein  hypothetical protein  hypothetical protein  putative secreted protein |
| 8762 | AGDB01000022.1:1..8762 | ATPase provides energy for both assembly of type IV secretion complex and secretion of T-DNA complex (VirB11)  ATPase provides energy for both assembly of type IV secretion complex and secretion of T-DNA complex (VirB4)  Bores hole in peptidoglycan layer allowing type IV secretion complex assembly to occur (VirB1)  FIG01213967: hypothetical protein  Inner membrane protein forms channel for type IV secretion of T-DNA complex (VirB10)  Inner membrane protein forms channel for type IV secretion of T-DNA complex (VirB3)  Major pilus subunit of type IV secretion complex (VirB2) |
| 8732 | AGDB01000129.1:1..8732 | Dihydroneopterin aldolase (EC 4.1.2.25)  Protein containing domains DUF403  Protein containing domains DUF4042C DUF407  Ribonuclease BN (EC 3.1.-.-)  SSU ribosomal protein S21p  Small-conductance mechanosensitive channel  Transamidase GatB domain protein  YgjD/Kae1/Qri7 family2C required for threonylcarbamoyladenosine (t(6)A) formation in tRNA |
| 8690 | AGDB01000245.1:1..8690 | FIG00582408: hypothetical protein  TonB-dependent receptor  hypothetical protein  two-component system sensor protein |
| 8669 | AGDB01000466.1:1..8669 | Aspartate aminotransferase (EC 2.6.1.1)  FIG01210031: hypothetical protein  FIG01211317: hypothetical protein  Glutaredoxin-like protein  Lysophospholipase L1 and related esterases  Probable GTPase related to EngC  ThiJ/PfpI family protein  acetylxylan esterase  hypothetical protein |
| 8610 | AGDB01000422.1:1..8610 | Methyl-accepting chemotaxis protein I (serine chemoreceptor protein)  Pectate lyase precursor (EC 4.2.2.2)  hypothetical protein  probable aminopeptidase |
| 8532 | AGDB01000363.1:1..8532 | Cellulose synthase catalytic subunit [UDP-forming] (EC 2.4.1.12)  Cyclic di-GMP binding protein precursor  Endoglucanase precursor (EC 3.2.1.4) |
| 8429 | AGDB01000119.1:1..8429 | Citrate synthase (si) (EC 2.3.3.1)  FIG01209925: hypothetical protein  Inosine-uridine preferring nucleoside hydrolase (EC 3.2.2.1)  LSU ribosomal protein L31p  Multimodular transpeptidase-transglycosylase (EC 2.4.1.129) (EC 3.4.-.-)  Type IV pilus biogenesis protein PilM  hypothetical protein |
| 8378 | AGDB01000005.1:1..8378 | ABC-type antimicrobial peptide transport system2C permease component  ABC-type antimicrobial peptide transport system2C permease component  FIG01210546: hypothetical protein  Methionine ABC transporter ATP-binding protein  Permease of the drug/metabolite transporter (DMT) superfamily  RarD protein2C chloamphenicol sensitive  hypothetical protein  hypothetical protein |
| 8288 | AGDB01000294.1:1..8288 | DNA-directed RNA polymerase omega subunit (EC 2.7.7.6)  GTP pyrophosphokinase (EC 2.7.6.5)2C (p)ppGpp synthetase II / Guanosine-3'2C5'-bis(diphosphate) 3'-pyrophosphohydrolase (EC 3.1.7.2)  Glyoxalase family protein  Guanylate kinase (EC 2.7.4.8)  Nucleoside 5-triphosphatase RdgB (dHAPTP2C dITP2C XTP-specific) (EC 3.6.1.15)  Protein YicC  Radical SAM family enzyme2C similar to coproporphyrinogen III oxidase2C oxygen-independent2C clustered with nucleoside-triphosphatase RdgB  Ribonuclease PH (EC 2.7.7.56) |
| 8243 | AGDB01000192.1:1..8243 | FIG01111295: hypothetical protein  FIG01210056: hypothetical protein  Quinolinate phosphoribosyltransferase [decarboxylating] (EC 2.4.2.19)  Soluble lytic murein transglycosylase and related regulatory proteins (some contain LysM/invasin domains)  Stringent starvation protein A  Stringent starvation protein B  Ubiquinol--cytochrome c reductase2C cytochrome B subunit (EC 1.10.2.2)  Ubiquinol-cytochrome C reductase iron-sulfur subunit (EC 1.10.2.2)  hypothetical protein  ubiquinol cytochrome C oxidoreductase2C cytochrome C1 subunit |
| 8218 | AGDB01000089.1:1..8218 | 4-diphosphocytidyl-2-C-methyl-D-erythritol kinase (EC 2.7.1.148)  FIG140336: TPR domain protein  GTP-binding and nucleic acid-binding protein YchF  LSU ribosomal protein L25p  Outer membrane lipoprotein LolB precursor  Peptidyl-tRNA hydrolase (EC 3.1.1.29)  Ribose-phosphate pyrophosphokinase (EC 2.7.6.1)  tRNA-Gln-TTG  tRNA-Gly-TCC  tRNA-Thr-GGT  tRNA-Tyr-GTA |
| 8146 | AGDB01000094.1:1..8146 | 4-hydroxyphenylpyruvate dioxygenase (EC 1.13.11.27)  Di-/tripeptide transporter  FIG01209887: hypothetical protein  FIG01210281: hypothetical protein  Homogentisate 12C2-dioxygenase (EC 1.13.11.5)  Transcriptional regulator2C MarR family  Tryptophan 22C3-dioxygenase (EC 1.13.11.11) |
| 8089 | AGDB01000424.1:1..8089 | 3-dehydroquinate synthase (EC 4.2.3.4)  MFS transporter  Periplasmic binding protein  Pyridoxamine 5'-phosphate oxidase (EC 1.4.3.5)  Pyrroline-5-carboxylate reductase (EC 1.5.1.2)  Redox-sensitive transcriptional activator SoxR  Shikimate kinase I (EC 2.7.1.71)  hypothetical protein  hypothetical protein  transcriptional regulator2C AraC family |
| 8027 | AGDB01000240.1:1..8027 | 5-Enolpyruvylshikimate-3-phosphate synthase (EC 2.5.1.19)  Chorismate mutase I (EC 5.4.99.5) / Prephenate dehydratase (EC 4.2.1.51)  FIG001196: putative membrane protein  FIG01210275: hypothetical protein  FIG01210359: hypothetical protein  Phosphoserine aminotransferase (EC 2.6.1.52)  Putative sulfite oxidase subunit YedY  hypothetical protein |
| 7944 | AGDB01000367.1:1..7944 | 3-oxoacyl-(acyl carrier protein) synthase (EC 2.3.1.41)  FIG003671: Metal-dependent hydrolase  FIG036672: Nucleoside-diphosphate-sugar epimerase  Glycosyl transferase2C family 2  hypothetical protein  hypothetical protein  periplasmic/7TM domain sensor diguanylate cyclase  putative acyltransferase (PhnO) |
| 7811 | AGDB01000436.1:1..7811 | 3-oxoacyl-[acyl-carrier protein] reductase (EC 1.1.1.100)  FIG01211551: hypothetical protein  RND efflux system2C inner membrane transporter CmeB  RND efflux system2C outer membrane lipoprotein CmeC  hypothetical protein  hypothetical protein |
| 7737 | AGDB01000259.1:1..7737 | Arsenate reductase (EC 1.20.4.1)  Cysteine synthase (EC 2.5.1.47)  Sensor histidine kinase  Siroheme synthase / Precorrin-2 oxidase (EC 1.3.1.76) / Sirohydrochlorin ferrochelatase (EC 4.99.1.4) / Uroporphyrinogen-III methyltransferase (EC 2.1.1.107)  Transcriptional regulator  XdhC protein (assists in molybdopterin insertion into xanthine dehydrogenase)  hypothetical protein |
| 7734 | AGDB01000430.1:1..7734 | Lhr-like helicases  Methyl-accepting chemotaxis protein I (serine chemoreceptor protein)  Methyl-accepting chemotaxis protein I (serine chemoreceptor protein)  Positive regulator of CheA protein activity (CheW)  Ribonuclease D (EC 3.1.26.3)  hypothetical protein  hypothetical protein  tRNA-Ala-CGC  virulence regulator |
| 7651 | AGDB01000407.1:1..7651 | FIG005107: hypothetical protein  FIG023677: hypothetical protein  GGDEF family protein  Ribonuclease T (EC 3.1.13.-)  hypothetical protein  hypothetical protein  hypothetical protein |
| 7651 | AGDB01000322.1:1..7651 | FIG01209962: hypothetical protein  Methyl-accepting chemotaxis protein I (serine chemoreceptor protein)  Methyl-accepting chemotaxis protein I (serine chemoreceptor protein)  Methyl-accepting chemotaxis protein I (serine chemoreceptor protein) |
| 7512 | AGDB01000069.1:1..7512 | Arginase (EC 3.5.3.1)  Beta-lactamase related protein  Entericidin EcnAB  FIG01111084: hypothetical protein  Protein yjbJ  Tryptophanyl-tRNA synthetase (EC 6.1.1.2)  entericidin A  hypothetical protein  hypothetical protein  hypothetical protein  hypothetical protein  tRNA-Thr-TGT |
| 7496 | AGDB01000353.1:1..7496 | 3-demethylubiquinone-9 3-methyltransferase (EC 2.1.1.64)  Lysyl-lysine 22C3-aminomutase  S-adenosylhomocysteine deaminase (EC 3.5.4.28); Methylthioadenosine deaminase  Similar to phosphoglycolate phosphatase2C clustered with ubiquinone biosynthesis SAM-dependent O-methyltransferase  Translation elongation factor P  diguanylate cyclase/phosphodiesterase (GGDEF  hypothetical protein |
| 7403 | AGDB01000450.1:1..7403 | Dipeptidyl carboxypeptidase Dcp (EC 3.4.15.5)  Ferredoxin--NADP( ) reductase (EC 1.18.1.2)  Glutathione peroxidase (EC 1.11.1.9)  Lipid A export ATP-binding/permease protein MsbA  hypothetical protein  hypothetical protein  hypothetical protein  hypothetical protein |
| 7361 | AGDB01000230.1:1..7361 | 2-methylcitrate dehydratase FeS dependent (EC 4.2.1.79)  2-methylcitrate synthase (EC 2.3.3.5)  Acid-resistant locus arl7 (Fragment)  Methylisocitrate lyase (EC 4.1.3.30)  hypothetical protein |
| 7360 | AGDB01000088.1:1..7360 | Cytochrome c-type biogenesis protein ResA  GCN5-related N-acetyltransferase  Glutamyl-tRNA reductase (EC 1.2.1.70)  Molybdenum cofactor biosynthesis protein MoaB  Peptide chain release factor 1  TPR domain protein2C putative component of TonB system  Transcriptional regulator  hypothetical protein  hypothetical protein |
| 7343 | AGDB01000126.1:1..7343 | LSU ribosomal protein L14p (L23e)  LSU ribosomal protein L16p (L10e)  LSU ribosomal protein L22p (L17e)  LSU ribosomal protein L23p (L23Ae)  LSU ribosomal protein L24p (L26e)  LSU ribosomal protein L29p (L35e)  LSU ribosomal protein L2p (L8e)  LSU ribosomal protein L3p (L3e)  LSU ribosomal protein L4p (L1e)  LSU ribosomal protein L5p (L11e)  SSU ribosomal protein S10p (S20e)  SSU ribosomal protein S14p (S29e)  SSU ribosomal protein S17p (S11e)  SSU ribosomal protein S19p (S15e)  SSU ribosomal protein S3p (S3e) |
| 7238 | AGDB01000066.1:1..7238 | Extracellular ribonuclease precursor (EC 3.1.-.-)  Heat shock protein 60 family chaperone GroEL  Heat shock protein 60 family co-chaperone GroES  Periplasmic divalent cation tolerance protein cutA  RNA polymerase sigma-70 factor2C ECF subfamily  transcriptional regulator2C putative |
| 7104 | AGDB01000402.1:1..7104 | Dihydrodipicolinate synthase (EC 4.2.1.52)  FIG01209675: hypothetical protein  FIG01210237: hypothetical protein  Glycine cleavage system transcriptional antiactivator GcvR  Phosphomethylpyrimidine kinase (EC 2.7.4.7)  Possible transmembrane protein  Predicted ATPase related to phosphate starvation-inducible protein PhoH  Thiol peroxidase2C Bcp-type (EC 1.11.1.15) |
| 7080 | AGDB01000044.1:1..7080 | ATP synthase B chain (EC 3.6.3.14)  ATP synthase alpha chain (EC 3.6.3.14)  ATP synthase beta chain (EC 3.6.3.14)  ATP synthase delta chain (EC 3.6.3.14)  ATP synthase epsilon chain (EC 3.6.3.14)  ATP synthase gamma chain (EC 3.6.3.14)  Periplasmic chorismate mutase I precursor (EC 5.4.99.5) |
| 7019 | AGDB01000036.1:1..7019 | FIG01209931: hypothetical protein  GTP-binding protein Era  Ribonuclease III (EC 3.1.26.3)  Serine protease precursor MucD/AlgY associated with sigma factor RpoE  Sigma factor RpoE negative regulatory protein RseA  Signal peptidase I (EC 3.4.21.89)  Translation elongation factor LepA |
| 6994 | AGDB01000117.1:1..6994 | 5-Hydroxyisourate Hydrolase (HIUase) (EC 3.5.2.17)  FIG00483075: hypothetical protein  FIG01210112: hypothetical protein  FIG01212343: hypothetical protein  N-carbamoyl-L-amino acid hydrolase (EC 3.5.1.87)  Oxidoreductase (EC 1.1.1.-)  Serine--pyruvate aminotransferase (EC 2.6.1.51) / L-alanine:glyoxylate aminotransferase (EC 2.6.1.44) |
| 6973 | AGDB01000410.1:1..6973 | FIG01211244: hypothetical protein  Glutamate-1-semialdehyde aminotransferase (EC 5.4.3.8)  Ribulose-5-phosphate 4-epimerase and related epimerases and aldolases  Xylulose kinase (EC 2.7.1.17)  hypothetical protein  putative; ORF located using Glimmer/Genemark  putative; ORF located using Glimmer/Genemark |
| 6879 | AGDB01000214.1:1..6879 | FIG01210495: hypothetical protein  FIG01210706: hypothetical protein  FIG111991: hypothetical protein  Fatty acid desaturase (EC 1.14.19.1); Delta-9 fatty acid desaturase (EC 1.14.19.1)  Ferredoxin II  Phosphoserine phosphatase (EC 3.1.3.3)  RNA polymerase sigma-70 factor  hypothetical protein |
| 6651 | AGDB01000312.1:1..6651 | 3'(2')2C5'-bisphosphate nucleotidase (EC 3.1.3.7)  ADP compounds hydrolase NudE (EC 3.6.1.-)  Adenosylmethionine-8-amino-7-oxononanoate aminotransferase (EC 2.6.1.62)  FIG027190: Putative transmembrane protein  Nucleoside triphosphate pyrophosphohydrolase MazG  Protein of unknown function UPF0060  Putative activity regulator of membrane protease YbbK  Putative stomatin/prohibitin-family membrane protease subunit YbbK  hypothetical protein |
| 6633 | AGDB01000215.1:1..6633 | FIG01210277: hypothetical protein  FIG01211069: hypothetical protein  Homocysteine S-methyltransferase (EC 2.1.1.10)  Hypothetical protein YaeR with similarity to glyoxylase family  Methionyl-tRNA synthetase (EC 6.1.1.10) |
| 6529 | AGDB01000383.1:1..6529 | ABC transporter involved in cytochrome c biogenesis2C ATPase component CcmA  FIG01209836: hypothetical protein  Methionine aminotransferase2C PLP-dependent  Possible hydrolase  hypothetical protein  hypothetical protein  hypothetical protein |
| 6363 | AGDB01000182.1:1..6363 | ATPase2C AFG1 family  Organic hydroperoxide resistance protein  Organic hydroperoxide resistance transcriptional regulator  Putative metal chaperone2C involved in Zn homeostasis2C GTPase of COG0523 family  hypothetical protein  hypothetical protein  hypothetical protein  integral membrane protein |
| 6324 | AGDB01000113.1:1..6324 | LigA  Methyl-accepting chemotaxis protein  NAD-dependent glyceraldehyde-3-phosphate dehydrogenase (EC 1.2.1.12)  Outer membrane protein W precursor  hypothetical protein |
| 6129 | AGDB01000251.1:1..6129 | DNA-binding response regulator  PTS system2C fructose-specific IIB component (EC 2.7.1.69) / PTS system2C fructose-specific IIC component (EC 2.7.1.69)  periplasmic sensor signal transduction histidine kinase  regulator of pathogenicity factors |
| 6012 | AGDB01000219.1:1..6012 | FIG01209964: hypothetical protein  Glycerol-3-phosphate acyltransferase (EC 2.3.1.15)  hypothetical protein  hypothetical protein  hypothetical protein  protein of unknown function DUF1130 |
| 5955 | AGDB01000220.1:1..5955 | hypothetical protein |
| 5950 | AGDB01000319.1:1..5950 | Methyl-accepting chemotaxis protein I (serine chemoreceptor protein)  Signal transduction histidine kinase CheA (EC 2.7.3.-) |
| 5941 | AGDB01000199.1:1..5941 | Dipeptidyl aminopeptidases/acylaminoacyl-peptidases  Phosphoenolpyruvate carboxylase (EC 4.1.1.31)  Transcriptional regulator2C TetR family |
| 5905 | AGDB01000269.1:1..5905 | Maltodextrin glucosidase (EC 3.2.1.20)  TonB-dependent receptor  hypothetical protein |
| 5895 | AGDB01000137.1:1..5895 | Glycosyltransferase  Radical SAM domain protein  biotin synthesis protein  chlorohydrolase family protein  hexosyltransferase |
| 5722 | AGDB01000114.1:1..5722 | FIG01210396: hypothetical protein  Phosphoglycerate kinase (EC 2.7.2.3)  Phosphoglycolate phosphatase (EC 3.1.3.18)  Probable transmembrane protein  Pyruvate kinase (EC 2.7.1.40) |
| 5704 | AGDB01000055.1:1..5704 | CBSS-498211.3.peg.1514: hypothetical protein  FIG01209759: hypothetical protein  GTP cyclohydrolase I (EC 3.5.4.16) type 1  dicarboxylate transport protein |
| 5691 | AGDB01000431.1:1..5691 | A/G-specific adenine glycosylase (EC 3.2.2.-)  FIG001341: Probable Fe(2 )-trafficking protein YggX  FIG01111754: hypothetical protein  Zn-dependent protease with chaperone function PA4632  hypothetical protein  hypothetical protein  serine/threonine kinase |
| 5670 | AGDB01000013.1:1..5670 | 5S RNA  Large Subunit Ribosomal RNA; lsuRNA; LSU rRNA  Small Subunit Ribosomal RNA; ssuRNA; SSU rRNA  tRNA-Ala-TGC  tRNA-Ile-GAT |
| 5551 | AGDB01000139.1:1..5551 | Cytochrome c heme lyase subunit CcmF  Cytochrome c heme lyase subunit CcmL  Cytochrome c-type biogenesis protein CcmG/DsbE2C thiol:disulfide oxidoreductase  FIG024006: iron uptake protein  hypothetical protein  putative C-type cytochrome biogenesis protein |
| 5521 | AGDB01000070.1:1..5521 | Phosphoenolpyruvate-protein phosphotransferase of PTS system (EC 2.7.3.9)  Transcriptional regulator lacI family  hypothetical protein  hypothetical protein |
| 5514 | AGDB01000076.1:1..5514 | Exodeoxyribonuclease V beta chain (EC 3.1.11.5)  Exodeoxyribonuclease V gamma chain (EC 3.1.11.5) |
| 5470 | AGDB01000186.1:1..5470 | 4'-phosphopantetheinyl transferase (EC 2.7.8.-) [enterobactin] siderophore  Endonuclease V (EC 3.1.21.7)  FIG01210964: hypothetical protein  Phosphoglycerate mutase (EC 5.4.2.1)  Pirin-related protein  hypothetical protein  hypothetical protein |
| 5421 | AGDB01000092.1:1..5421 | Metallopeptidase  Metallopeptidase  hypothetical protein  integral membrane rhomboid family serine protease MJ0610.1 |
| 5368 | AGDB01000043.1:1..5368 | FIG01210050: hypothetical protein  Glutaminyl-tRNA synthetase (EC 6.1.1.18)  two-component system regulatory protein  two-component system sensor protein  two-component system sensor protein |
| 5354 | AGDB01000352.1:1..5354 | FIG01214082: hypothetical protein  Inositol-1-monophosphatase (EC 3.1.3.25)  Phosphate-binding protein  tRNA:Cm32/Um32 methyltransferase |
| 5335 | AGDB01000131.1:1..5335 | FIG01209726: hypothetical protein  FIG01209890: hypothetical protein  FIG01211334: hypothetical protein  PQQ-dependent oxidoreductase2C gdhB family  cationic amino acid transporter  hypothetical protein |
| 5329 | AGDB01000148.1:1..5329 | Phosphoadenylyl-sulfate reductase [thioredoxin] (EC 1.8.4.8)  Sulfite reductase [NADPH] flavoprotein alpha-component (EC 1.8.1.2)  Sulfite reductase [NADPH] hemoprotein beta-component (EC 1.8.1.2) |
| 5275 | AGDB01000373.1:1..5275 | FIG01211260: hypothetical protein  Lytic transglycosylase  Trehalase (EC 3.2.1.28); Periplasmic trehalase precursor (EC 3.2.1.28)  hypothetical protein |
| 5239 | AGDB01000371.1:1..5239 | Predicted Zn-dependent peptidases  hypothetical protein |
| 5227 | AGDB01000233.1:1..5227 | Bis(5'-nucleosyl)-tetraphosphatase (asymmetrical) (EC 3.6.1.17)  DNA polymerase III subunits gamma and tau (EC 2.7.7.7)  FIG000557: hypothetical protein co-occurring with RecR  FIG001454: Transglutaminase-like enzymes2C putative cysteine proteases  FIG002343: hypothetical protein  Recombination protein RecR  Starvation lipoprotein Slp paralog |
| 5221 | AGDB01000268.1:1..5221 | Alpha-glucosidase (EC 3.2.1.20)  Six-hairpin glycosidase-like protein |
| 5143 | AGDB01000296.1:1..5143 | AcrA/AcrE family putative membrane-fusion protein  Acriflavin resistance protein  Thioredoxin  hypothetical protein |
| 5052 | AGDB01000331.1:1..5052 | TonB-dependent receptor  Transcriptional regulator  putative regulatory protein |
| 5001 | AGDB01000098.1:1..5001 | FecR protein  TonB-dependent receptor |
| 4999 | AGDB01000075.1:1..4999 | Ammonium transporter  Glutamine synthetase type I (EC 6.3.1.2)  Nitrogen regulatory protein P-II  Undecaprenyl-diphosphatase (EC 3.6.1.27)  hypothetical protein |
| 4994 | AGDB01000459.1:1..4994 | 2-isopropylmalate synthase (EC 2.3.3.13)  3-isopropylmalate dehydratase large subunit (EC 4.2.1.33)  3-isopropylmalate dehydratase small subunit (EC 4.2.1.33)  3-isopropylmalate dehydrogenase (EC 1.1.1.85)  hypothetical protein |
| 4952 | AGDB01000308.1:1..4952 | Dehydrogenases with different specificities (related to short-chain alcohol dehydrogenases)  Na -driven multidrug efflux pump  hypothetical protein  protease IV |
| 4896 | AGDB01000030.1:1..4896 | ATP synthase A chain (EC 3.6.3.14)  ATP synthase C chain (EC 3.6.3.14)  Dihydrolipoamide dehydrogenase of pyruvate dehydrogenase complex (EC 1.8.1.4)  FIG01210264: hypothetical protein  hypothetical protein  hypothetical protein  hypothetical protein |
| 4886 | AGDB01000062.1:1..4886 | FIG01212128: hypothetical protein  Quinone oxidoreductase (EC 1.6.5.5)  TonB-dependent receptor |
| 4814 | AGDB01000304.1:1..4814 | Endonuclease/exonuclease/phosphatase family protein  Periplasmic thiol:disulfide interchange protein DsbA  TonB-dependent receptor |
| 4788 | AGDB01000057.1:1..4788 | MoxR-like ATPase in aerotolerance operon  Type IV pilus biogenesis protein PilO  Type IV pilus biogenesis protein PilP  Type IV pilus biogenesis protein PilQ |
| 4770 | AGDB01000128.1:1..4770 | DNA primase (EC 2.7.7.-)  Sodium - Bile acid symporter  hypothetical protein |
| 4741 | AGDB01000133.1:1..4741 | Flagellar M-ring protein FliF  Flagellar assembly protein FliH  Flagellar motor switch protein FliG  Flagellar protein FliJ  Flagellum-specific ATP synthase FliI |
| 4651 | AGDB01000398.1:1..4651 | D-xylose proton-symporter XylE  GTP cyclohydrolase I (EC 3.5.4.16) type 2  Poly(A) polymerase (EC 2.7.7.19)  hypothetical protein  tRNA-Glu-CTC |
| 4609 | AGDB01000141.1:1..4609 | Cobalt-zinc-cadmium resistance protein CzcD  FIG065221: Holliday junction DNA helicase  Outer membrane lipoprotein carrier protein LolA  Protein crcB homolog  Transglutaminase-like enzymes2C putative cysteine proteases |
| 4576 | AGDB01000464.1:1..4576 | FIG00537784: hypothetical protein  Transcriptional regulator2C LuxR family  response regulator receiver domain protein (CheY-like) |
| 4463 | AGDB01000209.1:1..4463 | Leucine dehydrogenase (EC 1.4.1.9)  Serine protease |
| 4433 | AGDB01000118.1:1..4433 | 5-nucleotidase SurE (EC 3.1.3.5)  FIG139438: lipoprotein B  Lipoprotein NlpD  Protein-L-isoaspartate O-methyltransferase (EC 2.1.1.77)  ortholog of Bordetella pertussis (BX470248) BP2475 |
| 4399 | AGDB01000401.1:1..4399 | 12C4-lactonase (EC 3.1.1.25)  2-dehydro-3-deoxygalactonokinase (EC 2.7.1.58)  2-dehydro-3-deoxyphosphogalactonate aldolase (EC 4.1.2.21)  4Fe-4S ferredoxin2C iron-sulfur binding  Galactonate dehydratase (EC 4.2.1.6) |
| 4345 | AGDB01000228.1:1..4345 | ABC transporter2C ATP-binding protein  FIG01211383: hypothetical protein  hypothetical protein |
| 4332 | AGDB01000382.1:1..4332 | Cytochrome c heme lyase subunit CcmF  Cytochrome c-type biogenesis protein CcmC2C putative heme lyase for CcmE  Cytochrome c-type biogenesis protein CcmD2C interacts with CcmCE  Cytochrome c-type biogenesis protein CcmE2C heme chaperone  hypothetical protein |
| 4296 | AGDB01000216.1:1..4296 | COG3118: Thioredoxin domain-containing protein EC-YbbN  FIG01210157: hypothetical protein  FIG01211262: hypothetical protein  Putative preQ0 transporter  Transporter |
| 4248 | AGDB01000419.1:1..4248 | FIG01210004: hypothetical protein  Magnesium transporter  hypothetical protein |
| 4210 | AGDB01000461.1:1..4210 | 3-deoxy-D-manno-octulosonic-acid transferase (EC 2.-.-.-)  Glycosyltransferase  Lipid A biosynthesis lauroyl acyltransferase (EC 2.3.1.-)  membrane protein |
| 4159 | AGDB01000406.1:1..4159 | Phosphate transport ATP-binding protein PstB (TC 3.A.1.7.1)  Phosphate transport system permease protein PstA (TC 3.A.1.7.1)  Phosphate transport system permease protein PstC (TC 3.A.1.7.1)  Phosphate transport system regulatory protein PhoU |
| 4090 | AGDB01000394.1:1..4090 | Ferric siderophore transport system2C periplasmic binding protein TonB  NfuA Fe-S protein maturation  Pterin-4-alpha-carbinolamine dehydratase (EC 4.2.1.96)  Zinc transporter ZupT |
| 4089 | AGDB01000247.1:1..4089 | Biopolymer transport protein ExbD/TolR  Ferric siderophore transport system2C biopolymer transport protein ExbB  Lipid A export ATP-binding/permease protein MsbA (EC 3.6.3.25) |
| 4069 | AGDB01000158.1:1..4069 | Bifunctional protein: zinc-containing alcohol dehydrogenase; quinone oxidoreductase ( NADPH:quinone reductase) (EC 1.1.1.-); Similar to arginate lyase  COG2755: Lysophospholipase L1 and related esterases  Oxidoreductase  hypothetical protein |
| 4051 | AGDB01000120.1:1..4051 | Chemotaxis regulator - transmits chemoreceptor signals to flagelllar motor components CheY  Chemotaxis response - phosphatase CheZ  Flagellar biosynthesis protein FlhF  Flagellar synthesis regulator FleN  RNA polymerase sigma factor for flagellar operon |
| 4009 | AGDB01000205.1:1..4009 | FIG001154: CcsA-related protein  Signal recognition particle2C subunit Ffh SRP54 (TC 3.A.5.1.1)  putative; ORF located using Glimmer/Genemark |
| 4009 | AGDB01000163.1:1..4009 | hypothetical protein  tRNA uridine 5-carboxymethylaminomethyl modification enzyme GidA |
| 4003 | AGDB01000395.1:1..4003 | Ribosomal large subunit pseudouridine synthase C (EC 4.2.1.70)  hypothetical protein  hypothetical protein |
| 3949 | AGDB01000189.1:1..3949 | FIG01210190: hypothetical protein  TonB-dependent receptor  short chain dehydrogenase |
| 3898 | AGDB01000362.1:1..3898 | FIG01211368: hypothetical protein  FIG01213672: hypothetical protein  Nicotinate phosphoribosyltransferase (EC 2.4.2.11)  hypothetical protein |
| 3889 | AGDB01000203.1:1..3889 | Cysteine desulfurase (EC 2.8.1.7)  FIG01210717: hypothetical protein  FIG01211456: hypothetical protein |
| 3887 | AGDB01000235.1:1..3887 | Nuclease |
| 3883 | AGDB01000386.1:1..3883 | Fumarylacetoacetate hydrolase family protein  Maleylacetoacetate isomerase (EC 5.2.1.2) @ Glutathione S-transferase2C zeta (EC 2.5.1.18) |
| 3873 | AGDB01000376.1:1..3873 | Membrane-bound lytic murein transglycosylase B precursor (EC 3.2.1.-)  Polygalacturonase (EC 3.2.1.15) |
| 3703 | AGDB01000147.1:1..3703 | Chemotaxis regulator - transmits chemoreceptor signals to flagelllar motor components CheY  Chromosome (plasmid) partitioning protein ParA / Sporulation initiation inhibitor protein Soj  FIG01209829: hypothetical protein  Positive regulator of CheA protein activity (CheW) |
| 3678 | AGDB01000337.1:1..3678 | 4-hydroxyproline epimerase (EC 5.1.1.8)  D-amino-acid oxidase (EC 1.4.3.3)  Transcriptional regulator2C AraC family  sarcosine oxidase alpha subunit |
| 3651 | AGDB01000274.1:1..3651 | hypothetical protein  hypothetical protein  protein of unknown function DUF1244  putative Glutathione-regulated potassium-efflux system protein KefB |
| 3617 | AGDB01000361.1:1..3617 | FIG01211880: hypothetical protein  hypothetical protein |
| 3617 | AGDB01000172.1:1..3617 | ABC transporter2C ATP-binding protein  Probable MFS transporter |
| 3616 | AGDB01000197.1:1..3616 | 2-polyprenyl-6-methoxyphenol hydroxylase and related FAD-dependent oxidoreductases  FIG01210371: hypothetical protein  Mutator mutT protein (72C8-dihydro-8-oxoguanine-triphosphatase) (EC 3.6.1.-) / Thiamin-phosphate pyrophosphorylase-like protein  Transcriptional regulator2C TetR family |
| 3590 | AGDB01000248.1:1..3590 | Nucleoside-diphosphate-sugar epimerases  hypothetical protein |
| 3567 | AGDB01000017.1:1..3567 | FIG01210108: hypothetical protein  FIG01210386: hypothetical protein  FIG01211220: hypothetical protein  Flagellar motor rotation protein MotA  Flagellar motor rotation protein MotB |
| 3541 | AGDB01000258.1:1..3541 | Xanthine dehydrogenase2C iron-sulfur cluster and FAD-binding subunit A (1.17.1.4)  Xanthine dehydrogenase2C molybdenum binding subunit (EC 1.17.1.4) |
| 3536 | AGDB01000065.1:1..3536 | Branched-chain alpha-keto acid dehydrogenase2C E1 component2C alpha subunit (EC 1.2.4.4)  Branched-chain alpha-keto acid dehydrogenase2C E1 component2C beta subunit (EC 1.2.4.4)  FIG01209918: hypothetical protein |
| 3520 | AGDB01000176.1:1..3520 | Cellulase  Methyl-accepting chemotaxis protein I (serine chemoreceptor protein)  Uracil phosphoribosyltransferase (EC 2.4.2.9)  hypothetical protein |
| 3492 | AGDB01000134.1:1..3492 | Flagellar hook-basal body complex protein FliE  O-antigen biosynthesis protein |
| 3439 | AGDB01000211.1:1..3439 | hypothetical protein  hypothetical protein  hypothetical protein  virulence protein |
| 3431 | AGDB01000073.1:1..3431 | 4-alpha-glucanotransferase (amylomaltase) (EC 2.4.1.25) |
| 3261 | AGDB01000453.1:1..3261 | Cell division protein FtsW  Phospho-N-acetylmuramoyl-pentapeptide-transferase (EC 2.7.8.13)  UDP-N-acetylmuramoylalanyl-D-glutamyl-22C6-diaminopimelate--D-alanyl-D-alanine ligase (EC 6.3.2.10) |
| 3224 | AGDB01000086.1:1..3224 | Ribonucleotide reductase of class Ia (aerobic)2C alpha subunit (EC 1.17.4.1)  hypothetical protein |
| 3214 | AGDB01000369.1:1..3214 | hypothetical protein  hypothetical protein |
| 3210 | AGDB01000146.1:1..3210 | Flagellar motor rotation protein MotA  Signal transduction histidine kinase CheA (EC 2.7.3.-) |
| 3204 | AGDB01000266.1:1..3204 | SprB |
| 3137 | AGDB01000260.1:1..3137 | TonB-dependent receptor |
| 3109 | AGDB01000249.1:1..3109 | FIG01209842: hypothetical protein  Lipoprotein releasing system ATP-binding protein LolD  Lipoprotein releasing system transmembrane protein LolC  YgfY COG2938 |
| 3105 | AGDB01000414.1:1..3105 | Sigma-fimbriae tip adhesin  Sigma-fimbriae usher protein  hypothetical protein |
| 3102 | AGDB01000420.1:1..3102 | Arsenic efflux pump protein  amino acid transporter |
| 3060 | AGDB01000115.1:1..3060 | Fructose-bisphosphate aldolase class I (EC 4.1.2.13)  Metal-dependent phosphohydrolase2C HD subdomain  two component transcriptional regulator2C winged helix family |
| 3057 | AGDB01000159.1:1..3057 | Transcriptional regulator2C LysR family |
| 3051 | AGDB01000442.1:1..3051 | FIG00537023: hypothetical protein  Fosmidomycin resistance protein |
| 3039 | AGDB01000168.1:1..3039 | TonB-dependent receptor |
| 3032 | AGDB01000381.1:1..3032 | Cytochrome c heme lyase subunit CcmH  Cytochrome c heme lyase subunit CcmL  Homoserine O-acetyltransferase (EC 2.3.1.31)  hypothetical protein |
| 2991 | AGDB01000320.1:1..2991 | FIG01214082: hypothetical protein  Methyl-accepting chemotaxis protein I (serine chemoreceptor protein) |
| 2988 | AGDB01000321.1:1..2988 | Methyl-accepting chemotaxis protein I (serine chemoreceptor protein)  hypothetical protein |
| 2980 | AGDB01000239.1:1..2980 | Poly(Hydroxyalcanoate) granule associated protein  hypothetical protein  hypothetical protein |
| 2968 | AGDB01000195.1:1..2968 | Adenosylmethionine-8-amino-7-oxononanoate aminotransferase (EC 2.6.1.62)  Putrescine ABC transporter putrescine-binding protein PotF (TC 3.A.1.11.2) |
| 2964 | AGDB01000344.1:1..2964 | Two-component response regulator CreB  Two-component response regulator CreC |
| 2952 | AGDB01000079.1:1..2952 | Peptidyl-prolyl cis-trans isomerase ppiD (EC 5.2.1.8)  hypothetical protein  tRNA-Asp-GTC |
| 2947 | AGDB01000180.1:1..2947 | Membrane proteins related to metalloendopeptidases  Tyrosyl-tRNA synthetase (EC 6.1.1.1)  hypothetical protein |
| 2922 | AGDB01000262.1:1..2922 | TonB-dependent receptor  hypothetical protein |
| 2907 | AGDB01000227.1:1..2907 | putative sensory box histidine kinase  sigma-54 dependent DNA-binding response regulator |
| 2895 | AGDB01000256.1:1..2895 | Cobalt-zinc-cadmium resistance protein CzcA; Cation efflux system protein CusA  FIG01113058: hypothetical protein |
| 2882 | AGDB01000226.1:1..2882 | Penicillin-insensitive transglycosylase (EC 2.4.2.-) |
| 2842 | AGDB01000253.1:1..2842 | 3-oxoacyl-[ACP] synthase  3-oxoacyl-[ACP] synthase (EC 2.3.1.41) FabV like  FIG01210860: hypothetical protein |
| 2797 | AGDB01000171.1:1..2797 | Cellulose synthase operon protein C  Peptide deformylase (EC 3.5.1.88) |
| 2763 | AGDB01000156.1:1..2763 | ATP-dependent DNA helicase RecG (EC 3.6.1.-)  Endoribonuclease L-PSP  hypothetical protein |
| 2739 | AGDB01000454.1:1..2739 | Cell division protein FtsI [Peptidoglycan synthetase] (EC 2.4.1.129)  UDP-N-acetylmuramoylalanyl-D-glutamate--22C6-diaminopimelate ligase (EC 6.3.2.13) |
| 2706 | AGDB01000324.1:1..2706 | Methyl-accepting chemotaxis protein I (serine chemoreceptor protein)  chemotaxis protein |
| 2689 | AGDB01000469.1:1..2689 | GTP-binding protein EngB  Periplasmic thiol:disulfide interchange protein DsbA  Probable cytochrome c2 |
| 2673 | AGDB01000449.1:1..2673 | RNA polymerase sigma-54 factor RpoN  peptidase S8 and S532C subtilisin2C kexin2C sedolisin |
| 2673 | AGDB01000130.1:1..2673 | Beta-glucosidase (EC 3.2.1.21) |
| 2658 | AGDB01000183.1:1..2658 | Esterase/lipase  NAD-dependent protein deacetylase of SIR2 family  integral membrane sensor signal transduction histidine kinase |
| 2657 | AGDB01000243.1:1..2657 | ABC transporter2C ATP-binding protein  Cysteine desulfurase (EC 2.8.1.7)  RND efflux system2C outer membrane lipoprotein2C NodT family |
| 2567 | AGDB01000327.1:1..2567 | Chemotaxis protein CheD  Chemotaxis protein methyltransferase CheR (EC 2.1.1.80)  Methyl-accepting chemotaxis protein I (serine chemoreceptor protein) |
| 2556 | AGDB01000191.1:1..2556 | Phosphoribosylaminoimidazole carboxylase ATPase subunit (EC 4.1.1.21)  Phosphoribosylaminoimidazole carboxylase catalytic subunit (EC 4.1.1.21)  UPF0434 protein YcaR |
| 2535 | AGDB01000109.1:1..2535 | hypothetical protein |
| 2477 | AGDB01000179.1:1..2477 | FIG01211155: hypothetical protein  Methyl-accepting chemotaxis protein I (serine chemoreceptor protein) |
| 2436 | AGDB01000110.1:1..2436 | 2-dehydro-3-deoxygluconate kinase (EC 2.7.1.45)  Short-chain alcohol dehydrogenase family |
| 2432 | AGDB01000196.1:1..2432 | FIG01085402: hypothetical protein  Mlr6856 protein |
| 2424 | AGDB01000103.1:1..2424 | Bacterioferritin  Peroxiredoxin  low molecular weight heat shock protein |
| 2405 | AGDB01000347.1:1..2405 | hypothetical protein  hypothetical protein |
| 2331 | AGDB01000178.1:1..2331 | ISHne32C transposase  Transcriptional regulator2C PadR family  iron-chelator utilization protein |
| 2292 | AGDB01000009.1:1..2292 | Flavodoxins  Ribonucleotide reductase of class Ia (aerobic)2C beta subunit (EC 1.17.4.1)  Thioredoxin |
| 2281 | AGDB01000167.1:1..2281 | Chemotaxis protein methyltransferase CheR (EC 2.1.1.80) |
| 2232 | AGDB01000425.1:1..2232 | Lipase  RND efflux system2C outer membrane lipoprotein CmeC |
| 2226 | AGDB01000083.1:1..2226 | FIG01210504: hypothetical protein |
| 2225 | AGDB01000264.1:1..2225 | N-acetylmuramoyl-L-alanine amidase (EC 3.5.1.28) |
| 2216 | AGDB01000124.1:1..2216 | LigA  Thioredoxin |
| 2183 | AGDB01000412.1:1..2183 | FIG01210913: hypothetical protein  Penicillin acylase II |
| 2129 | AGDB01000397.1:1..2129 | 2-amino-4-hydroxy-6-hydroxymethyldihydropteridine pyrophosphokinase (EC 2.7.6.3)  3-methyl-2-oxobutanoate hydroxymethyltransferase (EC 2.1.2.11) |
| 2040 | AGDB01000150.1:1..2040 |  |
| 2026 | AGDB01000411.1:1..2026 |  |
| 1988 | AGDB01000169.1:1..1988 | MoxJ protein |
| 1952 | AGDB01000241.1:1..1952 | Seryl-tRNA synthetase (EC 6.1.1.11) |
| 1946 | AGDB01000185.1:1..1946 |  |
| 1867 | AGDB01000427.1:1..1867 | 33 kDa chaperonin (Heat shock protein 33) (HSP33)  Monofunctional biosynthetic peptidoglycan transglycosylase (EC 2.4.2.-) |
| 1844 | AGDB01000295.1:1..1844 | endonuclease  outer membrane protein |
| 1834 | AGDB01000221.1:1..1834 | hypothetical protein |
| 1832 | AGDB01000326.1:1..1832 |  |
| 1800 | AGDB01000157.1:1..1800 | Dihydrolipoamide acetyltransferase component of pyruvate dehydrogenase complex (EC 2.3.1.12) |
| 1760 | AGDB01000339.1:1..1760 | COG2990: Uncharacterized protein conserved in bacteria  RNA polymerase sigma-70 factor |
| 1760 | AGDB01000097.1:1..1760 |  |
| 1744 | AGDB01000437.1:1..1744 | RND efflux system2C membrane fusion protein CmeA  aklaviketone reductase |
| 1737 | AGDB01000452.1:1..1737 | transcriptional regulator |
| 1710 | AGDB01000194.1:1..1710 | Membrane fusion component of tripartite multidrug resistance system |
| 1692 | AGDB01000145.1:1..1692 | Flagellar biosynthesis protein FliC |
| 1590 | AGDB01000068.1:1..1590 | Biotin-protein ligase (EC 6.3.4.15) / Biotin operon repressor  Pantothenate kinase type III2C CoaX-like (EC 2.7.1.33) |
| 1497 | AGDB01000298.1:1..1497 | BlaR1 peptidase M56 family membrane protein  FIG01210969: hypothetical protein |
| 1486 | AGDB01000421.1:1..1486 | Methyl-accepting chemotaxis protein I (serine chemoreceptor protein)  hypothetical protein |
| 1433 | AGDB01000282.1:1..1433 | Probable transmembrane protein |
| 1423 | AGDB01000415.1:1..1423 | SSU ribosomal protein S2p (SAe)  Sigma-fimbriae chaperone protein |
| 1379 | AGDB01000084.1:1..1379 | Signal transduction histidine kinase CheA (EC 2.7.3.-) |
| 1360 | AGDB01000458.1:1..1360 | FIG005080: Possible exported protein |
| 1329 | AGDB01000184.1:1..1329 | Two-component system response regulator OmpR |
| 1325 | AGDB01000359.1:1..1325 | General secretion pathway protein L |
| 1212 | AGDB01000456.1:1..1212 | D-2-hydroxyglutarate dehydrogenase |
| 1185 | AGDB01000389.1:1..1185 | FIG01211421: hypothetical protein  Transcription repressor |
| 1152 | AGDB01000287.1:1..1152 | PROBABLE INTEGRAL MEMBRANE PROTEIN |
| 1123 | AGDB01000432.1:1..1123 | Signal recognition particle receptor protein FtsY (alpha subunit) (TC 3.A.5.1.1) |
| 1123 | AGDB01000190.1:1..1123 | Glutaredoxin-related protein  Superoxide dismutase [Fe] (EC 1.15.1.1) |
| 1010 | AGDB01000309.1:1..1010 | FIG01210453: hypothetical protein  FIG01211156: hypothetical protein |
| 1008 | AGDB01000305.1:1..1008 | Dihydrofolate synthase (EC 6.3.2.12) / Folylpolyglutamate synthase (EC 6.3.2.17) |
| 1006 | AGDB01000202.1:1..1006 |  |
