# Supplementary material for: Draft Genome Sequences of Xanthomonas sacchari and Two Banana-Associated Xanthomonads Reveal Insights into the Xanthomonas Group 1 Clade
Source: Genes (Basel). 2011 Dec 2;2(4):1050–65. doi: 10.3390/genes2041050 (PMC3927605; doi:10.3390/genes2041050)
Supplement: Supplementary File 1 — ZIP-Document (ZIP, 7075 KB) [file genes-02-01050-s001.zip › genes-11371-supplementary/phylogenetic_trees.pdf]

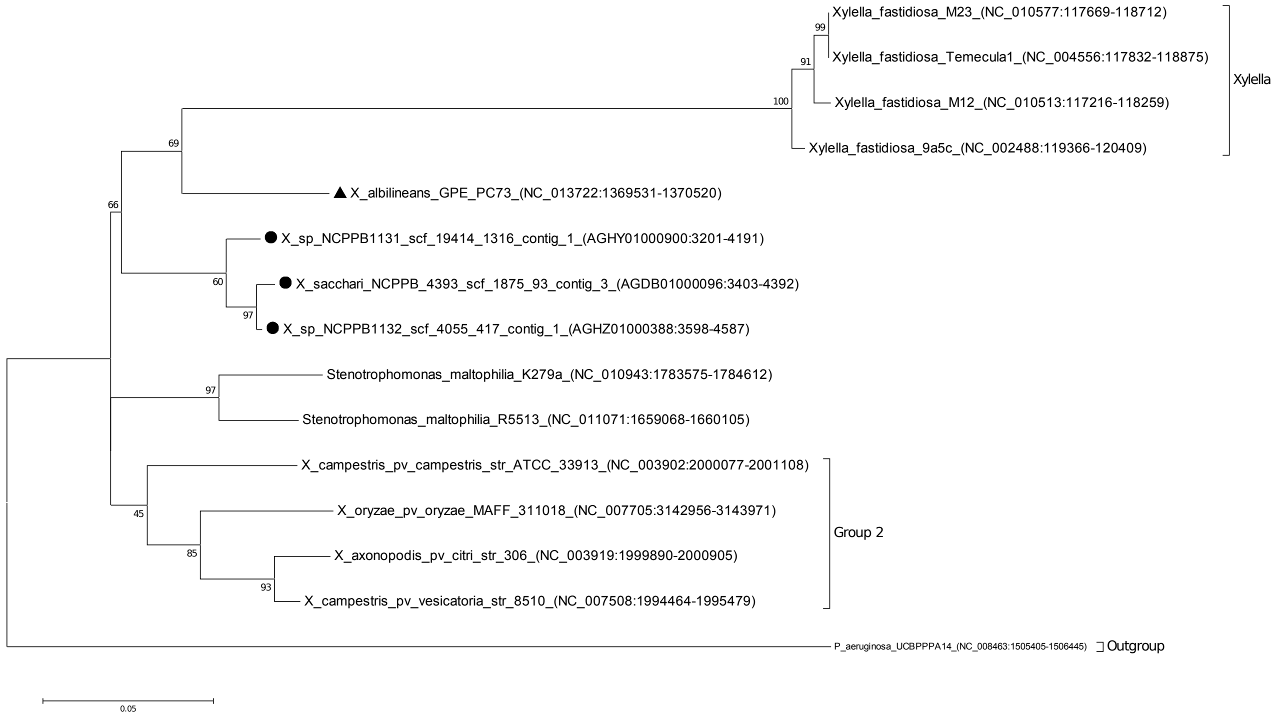


**Figure S1. Molecular Phylogenetic anaylsis of *recA* by Maximum Likelihood method.**
The evolutionary history was inferred by using the Maximum Likelihood method based on the Tamura-Nei model [1]. The bootstrap consensus tree inferred from 500 replicates [2] is taken to represent the evolutionary history of the taxa analyzed [2]. Branches corresponding to partitions reproduced in less than 50% bootstrap replicates are collapsed. The percentage of replicate trees in which the associated taxa clustered together in the bootstrap test (500 replicates) are shown next to the branches [2]. Initial tree(s) for the heuristic search were obtained automatically as follows. When the number of common sites was < 100 or less than one fourth of the total number of sites, the maximum parsimony method was used; otherwise BIONJ method with MCL distance matrix was used. The tree is drawn to scale, with branch lengths measured in the number of substitutions per site. The analysis involved 15 nucleotide sequences. Codon positions included were 1st+2nd+3rd+Noncoding. All positions containing gaps and missing data were eliminated. There were a total of 981 positions in the final dataset. Evolutionary analyses were conducted in MEGA5 [3]. New sequences generated in the present study are indicated with black circles (●). The previously sequenced *Xanthomonas albilineans* is indicated with a black triangle (▲). GenBank and RefSeq accession numbers and coordinates are given in parentheses.

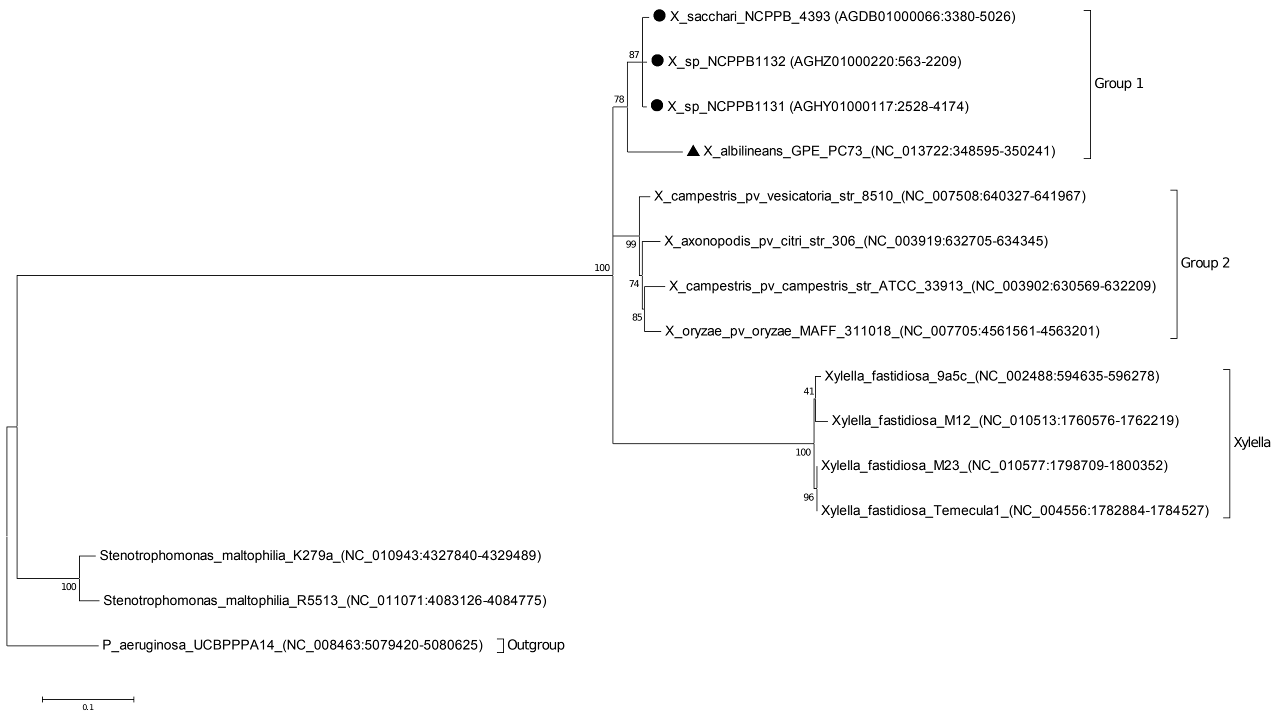


**Figure S2. Molecular Phylogenetic anaylsis of *groEL* by Maximum Likelihood method.** The evolutionary history was inferred by using the Maximum Likelihood method based on the Tamura-Nei model [1]. The bootstrap consensus tree inferred from 500 replicates [2] is taken to represent the evolutionary history of the taxa analyzed [2]. Branches corresponding to partitions reproduced in less than 50% bootstrap replicates are collapsed. The percentage of replicate trees in which the associated taxa clustered together in the bootstrap test (500 replicates) are shown next to the branches [2]. Initial tree(s) for the heuristic search were obtained automatically as follows. When the number of common sites was < 100 or less than one fourth of the total number of sites, the maximum parsimony method was used; otherwise BIONJ method with MCL distance matrix was used. The tree is drawn to scale, with branch lengths measured in the number of substitutions per site. The analysis involved 15 nucleotide sequences. Codon positions included were 1st+2nd+3rd+Noncoding. All positions containing gaps and missing data were eliminated. There were a total of 1052 positions in the final dataset. Evolutionary analyses were conducted in MEGA5 [3]. New sequences generated in the present study are indicated with black circles (●). The previously sequenced *Xanthomonas albilineans* is indicated with a black triangle (▲). GenBank and RefSeq accession numbers and coordinates are given in parentheses.

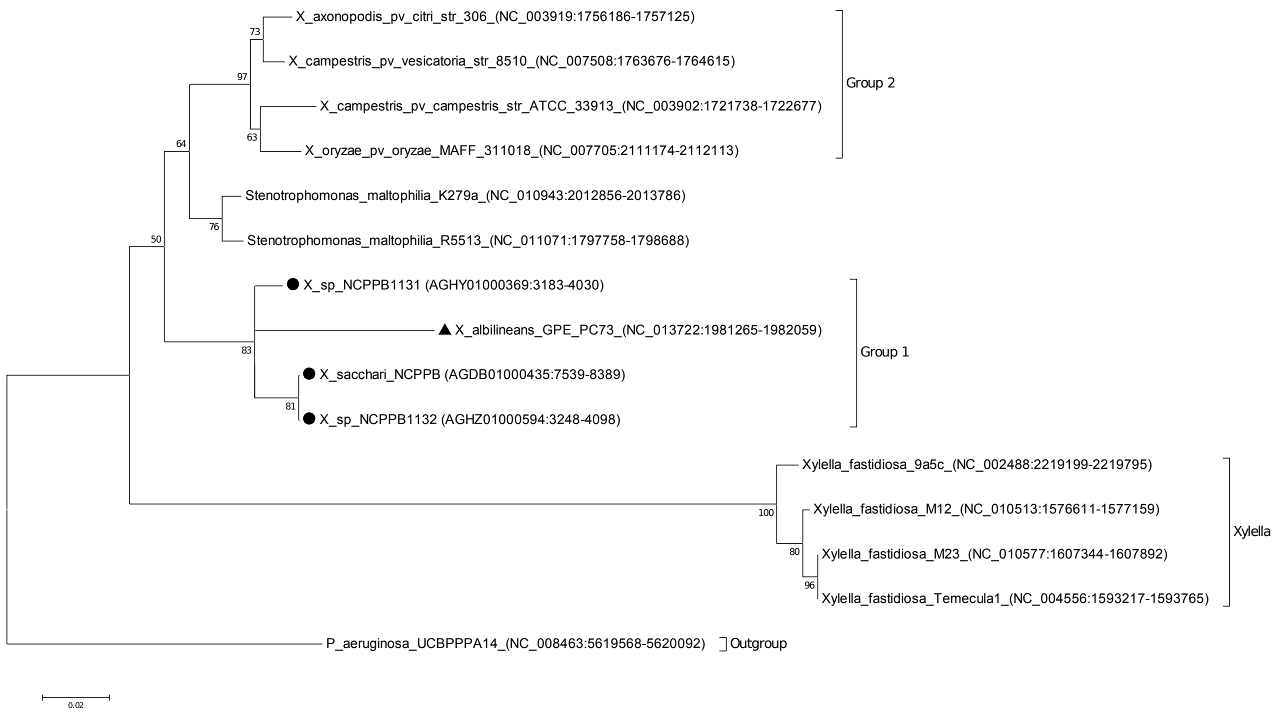


**Figure S3. Molecular Phylogenetic anaylsis of *dnaK* by Maximum Likelihood method.**
The evolutionary history was inferred by using the Maximum Likelihood method based on the Tamura-Nei model [1]. The bootstrap consensus tree inferred from 500 replicates [2] is taken to represent the evolutionary history of the taxa analyzed [2]. Branches corresponding to partitions reproduced in less than 50% bootstrap replicates are collapsed. The percentage of replicate trees in which the associated taxa clustered together in the bootstrap test (500 replicates) are shown next to the branches [2]. Initial tree(s) for the heuristic search were obtained automatically as follows. When the number of common sites was < 100 or less than one fourth of the total number of sites, the maximum parsimony method was used; otherwise BIONJ method with MCL distance matrix was used. The tree is drawn to scale, with branch lengths measured in the number of substitutions per site. The analysis involved 15 nucleotide sequences. Codon positions included were 1st+2nd+3rd+Noncoding. All positions containing gaps and missing data were eliminated. There were a total of 468 positions in the final dataset. Evolutionary analyses were conducted in MEGA5 [3]. New sequences generated in the present study are indicated with black circles (●). The previously sequenced *Xanthomonas albilineans* is indicated with a black triangle (▲). GenBank and RefSeq accession numbers and coordinates are given in parentheses.

## References

1. Tamura K. and Nei M. (**1993**). Estimation of the number of nucleotide substitutions in the control region of mitochondrial DNA in humans and chimpanzees. *Molecular Biology and Evolution* **10**:512-526.

2. Felsenstein J. (**1985**). Confidence limits on phylogenies: An approach using the bootstrap. *Evolution* **39**:783-791.

3. Tamura K., Peterson D., Peterson N., Stecher G., Nei M., and Kumar S. (**2011**). MEGA5: Molecular Evolutionary Genetics Analysis using Maximum Likelihood, Evolutionary Distance, and Maximum Parsimony Methods. *Molecular Biology and Evolution* **(In Press)**..
