# Supplementary material for: Draft Genome Sequences of Xanthomonas sacchari and Two Banana-Associated Xanthomonads Reveal Insights into the Xanthomonas Group 1 Clade
Source: Genes (Basel). 2011 Dec 2;2(4):1050–65. doi: 10.3390/genes2041050 (PMC3927605; doi:10.3390/genes2041050)
Supplement: Supplementary File 1 — ZIP-Document (ZIP, 7075 KB) [file genes-02-01050-s001.zip › genes-11371-supplementary/Supplementary data files.pdf]

Supplementary data files.

File name	File type	Description
GyraseA_alignment.pdf	PDF (Adobe Acrobat)	Multiple sequence alignment of partial GyrA proteins from *Xanthomonas albilineans* and other gamma-Proteobacteria
phylogenetic_trees.pdf	PDF (Adobe Acrobat)	Phylogenetic analysis of fully sequenced members of the genera *Xanthomonas*, *Xylella* and *Steonotrophomonas*. Maximum likelihood trees are based on nucleotide sequences of the house-keeping genes *dnaK*, *groEL* and *recA*
NCPPB1131-sequences-not-in-X_albilineans.html	HTML (web browser)	A list of regions in the genome of NCPPB1131 that share no detectable nucleotide sequence similarity with *X. albilineans*
NCPPB1132-sequences-not-in-X_albilineans.html	HTML (web browser)	A list of regions in the genome of NCPPB1132 that share no detectable nucleotide sequence similarity with *X. albilineans*
NCPPB4393-sequences-not-in-X_albilineans.html	HTML (web browser)	A list of regions in the genome of NCPPB4393 that share no detectable nucleotide sequence similarity with *X. albilineans*
NCPPB1131-RAST.xls	Microsoft Excel spreadsheet	Automated annotation results for NCPPB1131 draft genome sequence generated by the RAST server
NCPPB1132-RAST.xls	Microsoft Excel spreadsheet	Automated annotation results for NCPPB1132 draft genome sequence generated by the RAST server
NCPPB4393-RAST.xls	Microsoft Excel spreadsheet	Automated annotation results for NCPPB4393 draft genome sequence generated by the RAST server
NCPPB1131_genes_comparison.pdf	PDF (Adobe Acrobat)	A list of predicted genes in NCPPB1131 that are absent from NCPPB1132 and/or NCPPB4393 as judged by BWA alignments of raw Illumina sequence reads against the NCPPB1131 genome. Genomic coordinates are given on GenBank accession numbers
NCPPB1132_genes_comparison.pdf	PDF (Adobe Acrobat)	A list of predicted genes in NCPPB1132 that are absent from NCPPB1131 and/or NCPPB4393 as judged by BWA alignments of raw Illumina sequence reads against the NCPPB1132 genome. Genomic coordinates are given on GenBank accession numbers
NCPPB4393_genes_comparison.pdf	PDF (Adobe Acrobat)	A list of predicted genes in NCPPB4393 that are absent from NCPPB1131 and/or NCPPB1132 as judged by BWA alignments of raw Illumina sequence reads against the NCPPB4393 genome. Genomic coordinates are given on GenBank accession numbers
atpD_aligned_fasta.txt	Gapped FastA / plain text	Multiple sequence alignment of *atpD* used to generate the phylogenetic tree in Figure 1 in the main text
gyrB_aligned_fasta.txt	Gapped FastA / plain text	Multiple sequence alignment of *gyrB* used to generate the phylogenetic tree in Figure 1 in the main text
